# Pros and Cons of Aspirin for the Primary Prevention of Cardiovascular Events: A Secondary Study of Trial Sequential Analysis

**DOI:** 10.3389/fphar.2020.592116

**Published:** 2021-01-14

**Authors:** Binghao Zhao, Qian Wu, Li Wang, Chen Liao, Yifei Dong, Jingsong Xu, Yiping Wei, Wenxiong Zhang

**Affiliations:** ^1^ Department of Cardio-Thoracic Surgery, The Second Affiliated Hospital of Nanchang University, Nanchang, China; ^2^ Departments of Neurosurgery, Peking Union Medical College Hospital, Chinese Academy of Medical Sciences and Peking Union Medical College, Beijing, China; ^3^ Jiangxi Medical College, Nanchang University, Nanchang, China; ^4^ Department of Cardiology, The Second Affiliated Hospital of Nanchang University, Nanchang, China

**Keywords:** aspirin, primary prevention, cardiovascular disease, secondary study, trial sequential analysis

## Abstract

**Background and Aims:** Aspirin leads to substantial benefits for the secondary prevention of cardiovascular disease (CVD). We aimed to cast more light on aspirin’s role for the primary prevention of CVD.

**Methods:** Databases were searched for clinical trials comparing aspirin vs. no aspirin use in this meta-analysis. Efficacy and safety profiles were rigorously investigated. Trial sequential analysis (TSA) was used to determine the robustness of the results.

**Results:** Fourteen studies with 163,840 participants were eligible (mean follow-up 6.2 y). Aspirin intake was found to be associated with 9, 13, and 12% reductions in the risk of cardiovascular events (CV events) (relative risk [RR]: 0.91, 95% confidence intervals [CI]: 0.87–0.96; risk difference (RD): 0.29%; absolute risk percentage (AR%): 7.61%; number needed to treat (NNT): 345), myocardial infarction (RR: 0.87, 95% CI: 0.77–0.97; RD: 0.21%; AR%: 11.11%; NNT: 488) and ischemic stroke (RR: 0.88, 95% CI: 0.80–0.96; RD: 0.21%; AR%: 16.14%; NNT: 476), respectively; aspirin intake was also associated with 40%, 30%, and 57% increases in the risk of major bleeding (RR: 1.40, 95% CI: 1.29–1.53; RD: 0.47%; AR%: 27.85; NNT: 214), intracranial bleeding (RR: 1.30, 95% CI: 1.11–1.52; RD: 0.10%; AR%: 22.99%; NNT: 1,000) and major gastrointestinal bleeding (RR: 1.57, 95% CI: 1.38–1.78; RD: 0.32%; AR%: 36.70%; NNT: 315), respectively. Further, populations with low doses of aspirin intake (≤100 mg), populations <65 y old or populations with body mass index (BMI) ≧ 25 experienced more advantages; high-risk (10-y cardiovascular risk ≧10%) and full diabetic individuals reported hardly clinical benefits.

**Conclusion:** Aspirin intake was associated with a reduced risk of CV events and an increased incidence of bleeding profiles in primary prevention. It is necessary to identify individual’s CVD risk using clear examinations or assessments before aspirin intake, and truly realize individualized prescription.

## Introduction

Currently, many patients are at high risk because their health is influenced by occlusive vascular disease; indeed, a long-term antiplatelet regimen (e.g., aspirin therapy) reduces the yearly risk of worse vascular events (such as nonfatal myocardial infarction, nonfatal stroke and vessel-related death) by almost one-quarter (Antiplatelet Trialists’ Collaboration, 1994). Distinct benefits are observed with respect to the incidence of non-fatal cardiovascular events (CV events), with a small but definitive absolute risk reduction of approximately 10–20 CV events per 1,000 per year. Despite the benefits of aspirin, the absolute risk of major gastrointestinal or other major extracranial bleeding is also increased by an order of magnitude, so in secondary prevention, the benefits exceed the risks (Antithrombotic Trialists’ Collaboration, 2002).

For primary prevention in patients without prior cardiovascular disease (CVD), both the risk without aspirin and absolute benefits of aspirin are smaller than those in secondary prevention. Although rates of death from coronary heart disease (CHD) and stroke in America have significantly decreased, CVD and cerebrovascular disease remain a large health and economic burden (Bibbins-Domingo, 2016). New guidelines suggest that regardless of bleeding risk, the wide use of aspirin is recommended for patients with a moderate risk of CHD, and a low dosage of aspirin (75–100 mg daily) may be reasonably recommended to 40- to 70-year-old adults at high risk of CVD without increasing major bleeding (IIb grade). New guidelines also recommended that age should be considered as a key determinant of the CVD risk, as a daily dose aspirin (alone or in combination with other drugs) has been recommended for all people above a specific age. Low doses of aspirin should not be recommended as primary prevention for 70-year-olds or for individuals with a high risk of bleeding (Pearson et al., 2002; Wald and Law, 2003; Elwood et al., 2005; Bulugahapitiya et al., 2008; Fox et al., 2015a; Bibbins-Domingo, 2016; Piepoli et al., 2016; Grundy et al., 2019; Mortensen and Nordestgaard, 2020). However, a moderate risk of CVD is hard to define, and whether the high CVD risk populations as well as the diabetic populations can get real benefits from aspirin or not.

Deferring the start of long-term aspirin use for primary prevention is a noted alternative that has the main advantage of avoiding an increased risk of slight or major bleeding events but has the disadvantage that the initial manifestation may be a disabling or fatal event. In previous primary prevention trials (Peto et al., 1988; Steering Committee of the Physicians’ Health Study Research Group, 1989; The Medical Research Council’s General Practice Research Framework, 1998; Hansson et al., 1998; de Gaetano, 2001; Ridker et al., 2005; Belch et al., 2008; Ogawa et al., 2008; Fowkes et al., 2010; Ikeda et al., 2014; Saito et al., 2017; Bowman et al., 2018; Gaziano et al., 2018; McNeil et al., 2018), control populations with non-fatal CVD (non-fatal CHD or non-fatal occlusive stroke) would probably be prescribed long-term aspirin use to avoid recurrence, hence helping to compare the efficacy of immediate vs. deferred aspirin use.

A previous meta-analysis (Whitlock et al., 2016) noted that aspirin reduced all-cause mortality, myocardial infarction (MI), and ischemic stroke while increasing the risk of major bleeding; another pooled study (Zheng and Roddick, 2019) showed that aspirin reduced nonfatal MI but did not significantly influence all-cause mortality. Above mentioned studies had heterogeneous results on all-cause mortality because they had involved different number of trials conducted in different time. Another key controversial point was on individuals’ CVD risk classification that whether the higher risk individuals or the lower risk individuals could derive real prevention benefits from aspirin discussed by various guidelines or researchers. Actually, there are a lot of meta-analysis discussing this topic emerging yearly, not so many addressed their “cost-effectiveness”, which is to say if the conclusions are statistically sufficient and robust, no repetitive meta-analyses or further evidence are needed to some extent so that saving the cost on public health.

Given the large number of individuals affected by current studies and guidelines, and less helpful of the impact from no-innovative work on global health policy making, we conducted a comprehensive meta-analysis with the aim to resolve clinical controversial points under intention-to-treat principles and to evaluate the sufficiency of current synthesized evidence using trial sequential method.

## Methods

The current study was conducted in accordance with the Preferred Reporting Items for Systematic Reviews and Meta-Analysis (PRISMA) guidelines, the PRISMA Checklist was shown in Supplementary Table S1. The protocol is available in PROSPERO (CRD42019127570).

### Data Source and Study Selection

A rigorous search was performed in the PubMed, EMBASE, Cochrane Library, Web of Science and ClinicalTrials.gov databases from inception to February 1, 2020, to retrieve randomized controlled trials (RCTs) relating to aspirin use in patients without prior CVD. The search had no language restrictions. The main key words used were “aspirin”, “cardiovascular disease”, “cardiovascular events”, “coronary heart disease”, and “randomized controlled trials”. Reference lists of the eligible studies and identified meta-analyses were also reviewed (Supplementary Material S1).

The inclusion criteria were as follows: 1) enrolled adult participants (≥18 y) without preexisting CV events [CV events here include peripheral arterial disease, CHD, prior myocardial infarction (MI), ischemic stroke, prior percutaneous coronary intervention, prior coronary artery bypass grafting]; 2) compared aspirin use to no aspirin use (placebo included); 3) had a follow-up no less than 1 year to confirm the high quality of primary studies; 4) provided reliable and available outcome data (at least one primary efficacy outcome of interest was reported); and 5) was an RCT.

Studies with the most comprehensive outcomes were included to avoid duplications; studies that assessed patients with diabetes but without atherosclerosis were also considered. JPAD (Ogawa et al., 2008) and JPAD2 (Saito et al., 2017) trials were both included for they had different characteristics and proportion of the incorporated individuals as well as the differed follow-up. We excluded pure basic studies, reviews, and animal experiments.

### Data Extraction and Outcome Definition

Two authors (Binghao Zhao, Yiping Wei) independently performed the study screening and extracted the baseline characteristics of each eligible trial. The baseline characteristics included demographic characteristics of included populations, clinical information about the intervention/control arms, and essential outcome data as well as the study design. Fully adjusted models for adjusted hazard ratio (HR), odd ratio (OR) and relative risk (RR) of analyzed outcomes were used if the models were available in included studies. Fully adjusted variables were varied, however, mostly included sex, age, country, hypertension, diabetes and smoking status. If some studies used intention-to-treat principles, we extracted the intention-to-treat data. Any discrepancies between the reviewers were resolved by a third author. If there were any missing data, the original authors were contacted.

The primary efficacy outcomes were CV events, all-cause mortality and cardiovascular mortality due to their universal definitions and balance of efficacy and safety, which reduce heterogeneity among eligible studies. The secondary efficacy outcomes were all MI, total stroke, ischemic stroke, cancer incidence and cancer mortality. The safety profile outcomes were major bleeding, intracranial bleeding and major gastrointestinal bleeding, as defined by each eligible trial. Intracranial bleeding was treated as a potential outcome of aspirin use in addition to CV events. All these definitions follow per included study’s definition (Grundy et al., 2019).

Some studies even noted that aspirin increased the probability of cancer mortality, therefore, cancer outcomes were also appointed as exploratory outcome for robust evidence. The 10-y major adverse cardiovascular event rate (10-y MACE%) was extracted and calculated by multiplying the annualized event rate for cardiovascular mortality, nonfatal MI, and nonfatal stroke. A 10-y MACE% ≥ 10% was regarded as high risk; the others were regarded as low risk (Supplementary Material S1).

### Study Quality Assessment

Methodological quality assessment was performed by three co-authors (Binghao Zhao, Li Wang, Wenxiong Zhang). We used the Cochrane Risk and Bias Tool (Higgins et al., 2011) recommended by the Cochrane handbook to evaluate the quality of each eligible study. There were several terms regarding the methodological quality of RCTs, and each study could be categorized as low, high or unclear quality; low-quality studies and those with unclear quality had a high risk of bias. Details are provided in the Supplementary Material S1.

### Statistical Analysis

For descriptive purposes and statistical convenience, weighted frequencies were calculated for categorical variables using the provided sample size of each trial. Multivariable RRs and 95% confidence intervals (95% CIs) (De Lima Taga and Singer, 2018) for primary/secondary efficacy outcomes of interest and primary safety outcomes were estimated using the DerSimonian-Laird (D-L) random effects model considering the existence of within- and between-study variability. To further illustrate these outcome estimations, risk difference (RD), absolute risk percentage (AR%) and number needed to treat (NNT) were also analyzed. For further statistical purposes, HRs and ORs were considered RRs in this study. Fully adjusted effect sizes (ESs) were logarithmically transformed to stabilize the variance; hence, the data distribution could be normalized.

Between-study heterogeneity and variability were quantified by Cochran’s Q test and *I* ^2^, whereby an *I* ^2^ > 50% or a *p*-value for the Q test <0.10 was considered to represent significant heterogeneity (Higgins et al., 2003). To provide more clinical implications, we conducted comprehensive subgroup analyses mainly focusing on several significant variables, including region (North America vs. Europe vs. Asia vs. multiple nations), individuals’ main age (<65 vs. ≧ 65 y), mean body mass index (BMI) (<25 vs. ≧ 25), aspirin dose taken (≤100 vs. > 100 mg) and 10-y MACE% (low risk vs. high risk). For 10-y MACE%, the computed value of 10-y MACE% < 10% was defined as low risk, but the other populations were high risk. To provide more useful clinical data as well as to investigate the influence of individual studies on final results, we carried out sensitivity analyses by omitting one study each turn.

Publication bias was assessed by funnel plots and Egger’s test (Egger et al., 1997), with *p* < 0.05 indicating significant bias. All analyses were performed using *R* project software (version 3.5.3, https://www.r-project.org/, United States) with forest, ggplot2, survminer etc. public packages; a two-sided *p* < 0.05 was considered statistically significant except where otherwise specified. More details are provided in the Supplementary Material S1.

### Trial Sequential Analysis

Previous studies have confirmed that the risk of type 1 error from interim analyses can be reasonably reduced through monitoring boundaries and modifying the *p*-value. Similar in meta-analyses, random errors caused by sparse data and repetitive testing also enhance the risk of type 1 error. Such a method setting analogous trial sequential monitoring boundaries to meta-analyses is called trial sequential analysis (TSA), is used to determine whether evidence is reliable or conclusive (Wetterslev et al., 2008; Brok et al., 2009). Actually, random errors can be rectified and reduced using TSA software [version 0.9 beta (http://www.ctu.dk/tsa)] because it combines the estimation of the required information size (RIS) with an adjusted threshold for statistical significance. We assumed that if the Z-curve crossed the TSA boundary or entered the futility area, a sufficient effect was obtained, and further studies were not required; otherwise, the amount of evidence was considered insufficient. TSA was performed for a 10% relative risk reduction, conservatively, according to the TSA manual; there was also a 5% (*α* = 0.05; two-sided) risk of a type 1 error and 80% statistical power. Other parameters were set empirically following default settings.

## Results

### Study Selection and Characteristics

**FIGURE 1:**
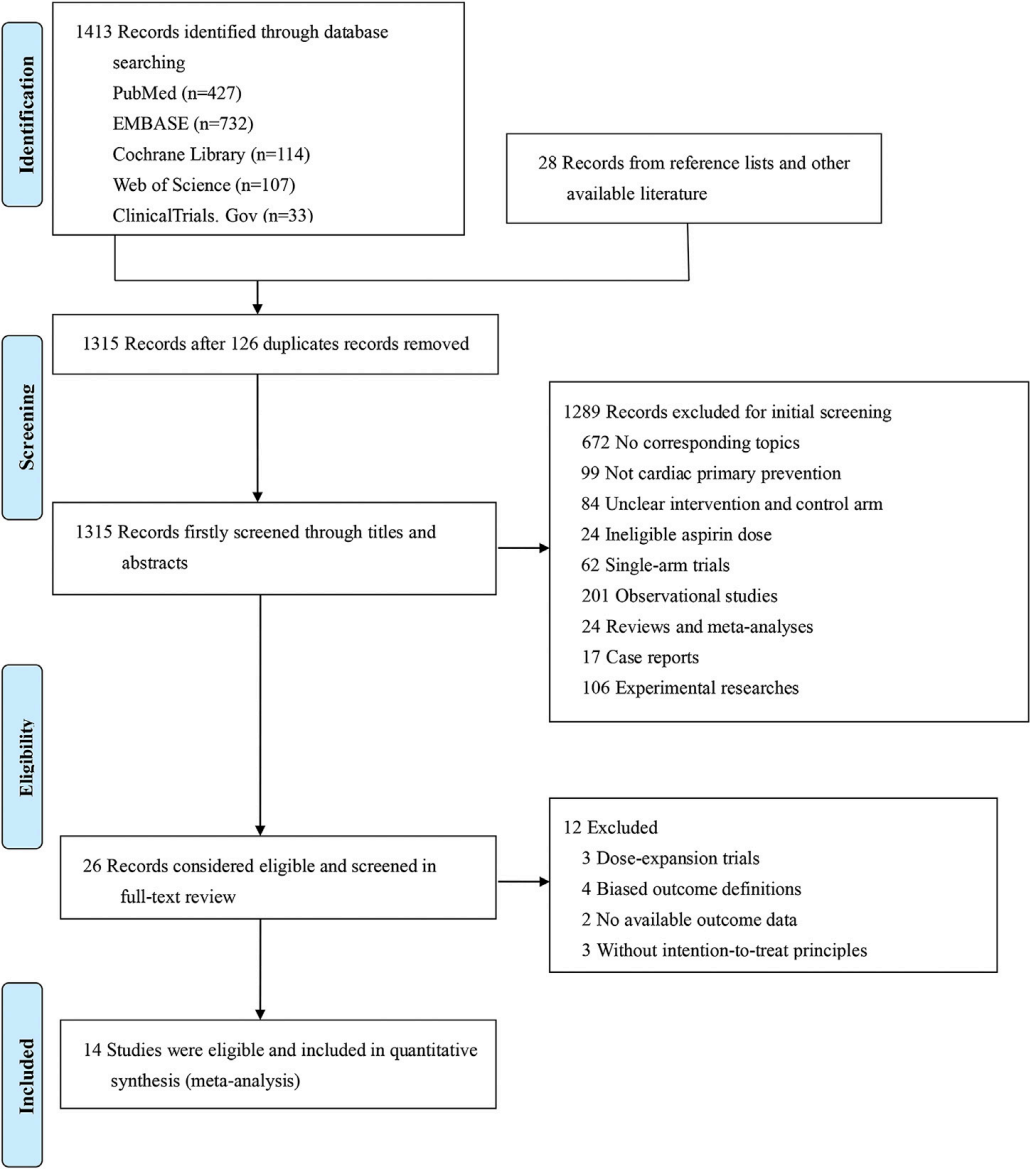
Flow chart for literature search.

Among 1,441 searched articles (1,423 from database searching and 28 from other available source), we identified 26 studies for full-text review, of which 14 studies were eligible for qualitative and quantitative analyses (Figure 1). The 14 included studies (Peto et al., 1988; Steering Committee of the Physicians’ Health Study Research Group, 1989; The Medical Research Council’s General Practice Research Framework, 1998; Hansson et al., 1998; de Gaetano, 2001; Ridker et al., 2005; Belch et al., 2008; Ogawa et al., 2008; Fowkes et al., 2010; Ikeda et al., 2014; Saito et al., 2017; Bowman et al., 2018; Gaziano et al., 2018; McNeil et al., 2018) encompassed a total of 163,840 patients and used intention-to-treat principles. The detailed study characteristics are summarized in Table 1.

**TABLE 1:** Characteristics of included studies and participants.

Publication	Study population	Number of population	Mean age y/Male (%)	Aspirin use (mg/day)	Control group	Diabetes No. (%)	Current smokers NO. (%)	Hypertension NO. (%)	Mean SBP (mean ± SD) mmHg	Total Cholesterol (mean ± SD) mmol/L	BMI	Outcomes	Study period (follow-up y)	Quality assessment^b^
Peto 1988; United Kingdom, (BDS) (Peto et al., 1988)	Male physicians	5,139 (3,429/1710)	61/5,139 (100)	300 or 500	No aspirin	101 (2)	661 (13)	508 (10)	136 ± 17	NA	24.4 ± 2.5	②③④⑤⑥⑦⑧⑨⑩	1978–1984 (NA)	High risk
Steering 1989; America, (PHS) (Steering Committee of the Physicians’ Health Study Research Group, 1989)	Male physicians	22,071 (11,037/11,034)	53/22,071 (100)	325	Placebo	533 (2)	2,438 (11)	5,297 (24)	126 ± 12	5.5 ± 1.2	24.9 ± 3.0	②③④⑤⑥⑧⑨⑩⑪	1982–1988 (5)	High risk
Meade 1998; United Kingdom, (TPT) (The Medical Research Council’s General Practice Research Framework, 1998)	Males in the top 20–25% risk of CV events	2,540 (1,268/1,272)^c^	57/2,540 (100)	75	Placebo	51 (2)	83 (3)	278 (11)	139 ± 18	6.4 ± 1.0	27.4 ± 3.6	②③④⑤⑥⑧⑨⑩⑪	1984–1997 (NA)	High risk
Hansson 1998; multi-nations, (HOT) (Hansson et al., 1998)	Hypertensive populations	18,790 (9,399/9,391)	61/9,959 (53)	75	Placebo	1,503 (8)	2,988 (16)	18,790 (100)	170 ± 14	6.0 ± 1.1	28.4 ± 4.7	①②③④⑤⑦⑧⑨⑩⑪	1992–1997 (3.8)	Low risk
De Gaetano 2001; Italy, (PPP) (de Gaetano, 2001 **)**	Populations with ≥1 CV risk factor	4,495 (2,226/2,269)	64/1912 (42)	100	No aspirin	742 (17)	667 (15)	3,065 (68)	145 ± 16	6.1 ± 1.2	27.6 ± 4.7	①②③④⑤⑥⑦⑧⑨⑩⑪	1994–1998 (3.6)	High risk
Ridker 2005; America, (WHS) (Ridker et al., 2005)	Healthy females	39,876 (19,934/19,942)	54/0 (0)	100	Placebo	1,037 (3)	5,224 (13)	10,328 (26)	NA	5.2 ± 1.0	26.1 ± 5.2	①②③④⑤⑥⑦⑧⑨⑩⑪	1992–2004 (10.1)	Low risk
Belch 2008; United Kingdom, (POPADAD) (Belch et al., 2008)	Diabetic populations (ABPI ≤0.99)	1,276 (638/638)	60/563 (44)	100	Placebo	1,276 (100)	NA	NA	145 ± 21	5.5	29.2	②③④⑤⑦⑧	1997–2006 (6.7) (ISRCTN53295293)	Low risk
Ogawa et al, 2008; Japan, (JPAD) (Ogawa et al., 2008)	Diabetic populations	2,539 (1,262/1,277)	65/1,387 (55)	81 or 100	No aspirin	2,539 (100)	537 (21)	1,473 (58)	135 ± 15	5.2 ± 0.9	24.0 ± 4.0	①②③④⑤⑥⑦⑧⑨⑩⑪	2002–2008 (4.37) (NCT00110448)	High risk
Fowkes 2010; United Kingdom, (AAA) (Fowkes et al., 2010)	Populations with ≤0.95 ABPI	3,350 (1,675/1,675)	62/954 (28)	100	Placebo	88 (3)	1,085 (32)	NA	148 ± 22	6.2 ± 1.1	NA	①②③④⑤⑥⑦⑧⑨⑩⑪	1998–2008 (8.2) (ISRCTN66587262)	Low risk
Ikeda 2014; Japan. (JPPP) (Ikeda et al., 2014)	Hypertensive, hyperlipidemic or diabetic populations	14,464 (7,220/7,244)	71/6,123 (42)	100	No aspirin	4,903 (34)	1893 (13)	12,278 (85)	137 ± 16	5.3 ± 0.8	24.2 ± 3.5	①②③④⑤⑥⑦⑧⑨⑩	2005–2012 (5.02) (NCT00225849)	High risk
Saito et al, 2017; Japan, (JPAD2) (Saito et al., 2017)	Diabetic populations	2,160 (992/1,168)	65/1,195 (55)	81 or 100	No aspirin	2,160 (100)	459 (21)	2,142 (58)	135 ± 15	5.2 ± 0.9	24.0 ± 4.0	①③④⑤⑥⑨⑩⑪	2002–2015 (10.3) (NCT00110448)	High risk
Bowman 2018; United Kingdom, (ASCEND) (Bowman et al., 2018)	Diabetic populations	15,480 (7,740/7,740)	63/9,684 (63)	100	Placebo	15,480 (100)	1,279 (8)	9,533 (62)	136 ± 15	4.2 ± 0.9	30.7 ± 6.3	①②③④⑤⑥⑦⑧⑨⑩⑪	2007–2016 (7.4) (NCT00135226)	Low risk
Gaziano 2018; multi-nations, (ARRIVE) (Gaziano et al., 2018)	Males with ≥2 and females with ≥3 CV risk factors, with 10–20% 10-y MACE risk	12,546 (6,270/6,276)	64/8,838 (70)	100	Placebo	0 (0)	3,594 (29)	7,866 (63)	144 (90–199)^e^	NA	28.4 ± 4.3	①②③④⑤⑩⑪	2007–2016 (5) (NCT00501059)	Low risk
McNeil 2018; multi-nations, (ASPREE) (McNeil et al., 2018)	≥65 y populations	19,114 (9,525/9,589)	74/8,331 (44)	100	Placebo	2057 (11)	735 (4)	14,283 (74)	140 ± 17	5.3 ± 1.0	28.1 ± 4.7	①②③④⑤⑥⑦⑧⑨⑩⑪	2010–2014 (4.7) (NCT01038583)	Low risk

Abbreviations: SBP, systolic blood pressure; BMI, body mass index; MACE, major adverse cardiovascular events; CV risk, cardiovascular risk; ABPI, ankle-brachial pressure index; SD, standard deviation; MI, myocardial infraction; NA, not available.

Outcome classification: ①, CV events; ②, All-cause mortality; ③, Cardiovascular mortality; ④, All MI; ⑤, Total stroke; ⑥, Ischemic stroke; ⑦, Cancer incidence; ⑧, Cancer mortality; ⑨, Major bleeding; ⑩, Intracranial bleeding; ⑪, Major gastrointestinal bleeding.

^a^ 10-y MACE% was calculated by multiplying the annualized event rate for cardiovascular outcomes in the control group by 10 years. MACE was defined as composite of cardiovascular mortality, non-fatal myocardial infraction and non-fatal stroke etc.

^b^ Methodology quality was assessed by Cochrane risk and Bias tool.

^c^ There were 5,085 participants randomized in a 2*2 factorial design with warfarin, aspirin, warfarin and aspirin or placebo, we excluded 2,545 populations with warfarin or warfarin and aspirin. 2,540 were randomized to aspirin and placebo.

Two studies (Steering Committee of the Physicians’ Health Study Research Group, 1989; Ridker et al., 2005) were conducted in America, six studies were conducted in Europe (5 (Peto et al., 1988; The Medical Research Council’s General Practice Research Framework, 1998; Belch et al., 2008; Saito et al., 2017; Bowman et al., 2018) in the United Kingdom and 1 (de Gaetano, 2001) in Italy), three studies (Ogawa et al., 2008; Ikeda et al., 2014; Saito et al., 2017) were performed in Japan, and three studies (Hansson et al., 1998; Gaziano et al., 2018; McNeil et al., 2018) were performed in multiple nations. The comparator treatment was a placebo group in nine studies (Steering Committee of the Physicians’ Health Study Research Group, 1989; The Medical Research Council’s General Practice Research Framework, 1998; Hansson et al., 1998; Ridker et al., 2005; Belch et al., 2008; Fowkes et al., 2010; Bowman et al., 2018; Gaziano et al., 2018; McNeil et al., 2018) and was a no aspirin group in five studies. Of note, in addition to aspirin and placebo, six studies used a factorial design, in which 1 (The Medical Research Council’s General Practice Research Framework, 1998) study used warfarin, 2 (de Gaetano, 2001); (Ridker et al., 2005) used vitamin E, 1 (Bowman et al., 2018) prescribed n-3 fatty acid, 1 (Belch et al., 2008) used antioxidants, and 1 (Peto et al., 1988) supplied anti-hypertension drugs. Three studies (Peto et al., 1988; Steering Committee of the Physicians’ Health Study Research Group, 1989; The Medical Research Council’s General Practice Research Framework, 1998) exclusively enrolled male individuals (29,750 males), and one study (Ridker et al., 2005) specially enrolled female individuals (39,876 females). Across the included studies, 78,696 (48%) patients were males. Four studies (Belch et al., 2008; Ogawa et al., 2008; Saito et al., 2017; Bowman et al., 2018) exclusively enrolled diabetic patients (including type I and type II diabetes). The mean BMI of eligible participants was 28.5, and the mean 10-y MACE% was 7.24. The median duration was 8.1 y (4 (de Gaetano, 2001) to 13 (The Medical Research Council’s General Practice Research Framework, 1998; Saito et al., 2017)), and the mean follow-up was 6.2 y. The studies were published between 1988 (Peto et al., 1988) and 2018 (Bowman et al., 2018; Gaziano et al., 2018; McNeil et al., 2018). All studies were written in English, and there was no attempt to ask the primary authors for raw data.

## Methodological Quality Assessment

Of the 14 included studies, nine studies used double-blind methods and five studies (Peto et al., 1988; de Gaetano, 2001; Ogawa et al., 2008; Ikeda et al., 2014; Saito et al., 2017) used open-label settings. Three studies (Steering Committee of the Physicians’ Health Study Research Group, 1989; The Medical Research Council’s General Practice Research Framework, 1998; de Gaetano, 2001) had selective reporting or other bias. Of the included studies, 7 (Hansson et al., 1998; Ridker et al., 2005; Belch et al., 2008; Fowkes et al., 2010; Bowman et al., 2018; Gaziano et al., 2018; McNeil et al., 2018) were of low risk and 7 (Peto et al., 1988; Steering Committee of the Physicians’ Health Study Research Group, 1989; The Medical Research Council’s General Practice Research Framework, 1998; de Gaetano, 2001; Ogawa et al., 2008; Ikeda et al., 2014; Saito et al., 2017) were of high risk (Supplementary Figure S1; Supplementary Table S2).

### The Primary Efficacy Outcomes

**FIGURE 2:**
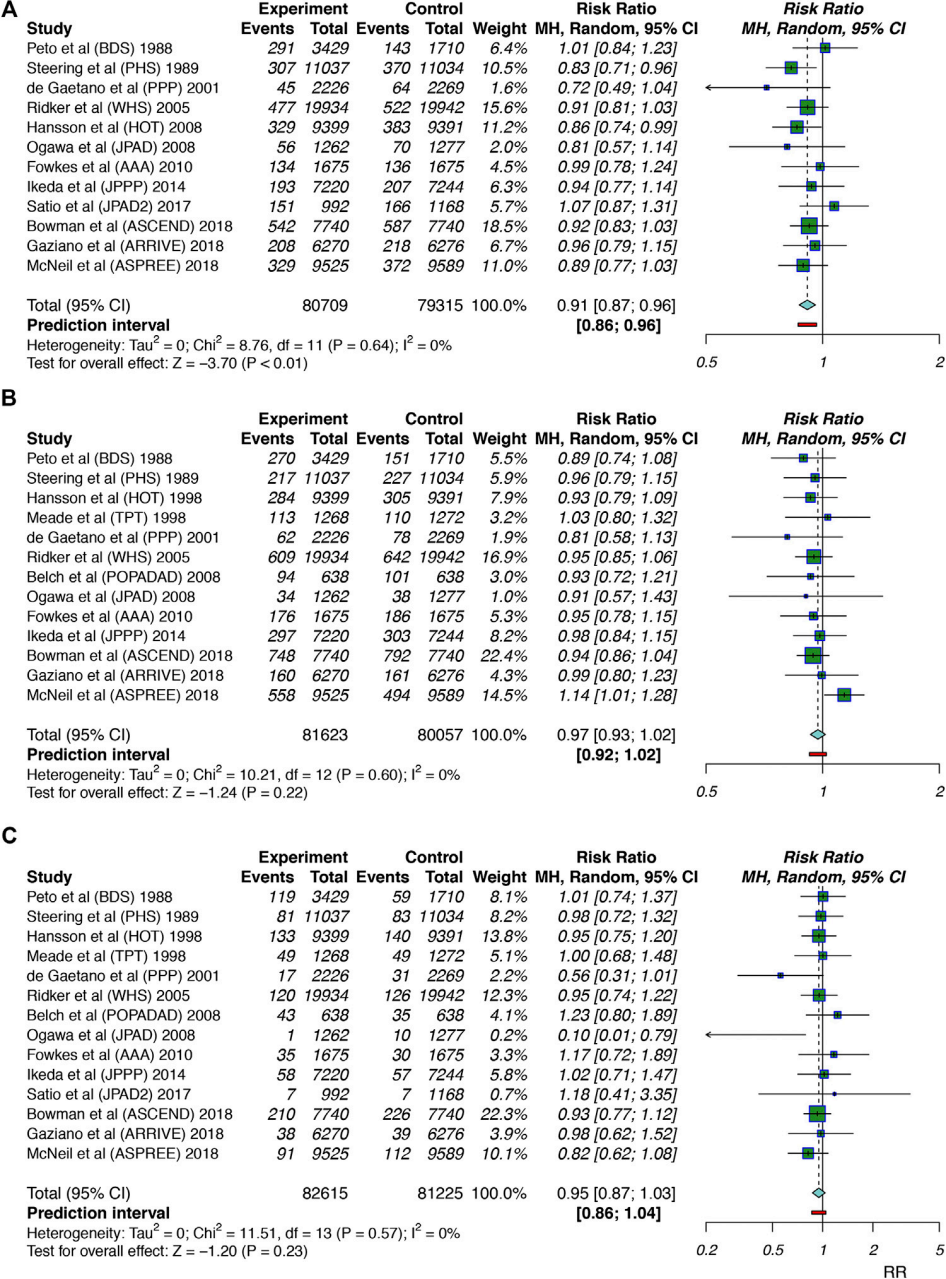
Summary forest plots for the primary efficacy outcomes. **(A)** Forest plot for CV events. **(B)** Forest plot for all-cause mortality. **(C)** Forest plot for cardiovascular mortality.

For the primary efficacy outcomes, twelve studies (Peto et al., 1988; Steering Committee of the Physicians’ Health Study Research Group, 1989; Hansson et al., 1998; de Gaetano, 2001; Ridker et al., 2005; Ogawa et al., 2008; Fowkes et al., 2010; Ikeda et al., 2014; Saito et al., 2017; Bowman et al., 2018; Gaziano et al., 2018; McNeil et al., 2018) involving 160,024 individuals reported CV event outcomes, and we found that the use of aspirin was associated with a 9% reduction in CV events (RR: 0.91, 95% CI: 0.87–0.96; *p* < 0.001; RD: 0.29%; AR%: 7.61%; NNT = 345) compared to no aspirin use, and there was no significant heterogeneity (I^2^ = 0; *p* = 0.64). Thirteen studies (Peto et al., 1988; Steering Committee of the Physicians’ Health Study Research Group, 1989; The Medical Research Council’s General Practice Research Framework, 1998; Hansson et al., 1998; de Gaetano, 2001; Ridker et al., 2005; Belch et al., 2008; Ogawa et al., 2008; Fowkes et al., 2010; Ikeda et al., 2014; Bowman et al., 2018; Gaziano et al., 2018; McNeil et al., 2018) including 161,680 individuals examined all-cause mortality outcomes; aspirin use did not lead to a significant reduction in all-cause mortality (RR: 0.97, 95% CI: 0.93–1.02; *p* = 0.22; RD: 0.04%; AR%: 0.99%; NNT = 2,273), and there was no heterogeneity (I^2^ = 0; *p* = 0.60). Fourteen studies (Peto et al., 1988; Steering Committee of the Physicians’ Health Study Research Group, 1989; The Medical Research Council’s General Practice Research Framework, 1998; Hansson et al., 1998; de Gaetano, 2001; Ridker et al., 2005; Belch et al., 2008; Ogawa et al., 2008; Fowkes et al., 2010; Ikeda et al., 2014; Saito et al., 2017; Bowman et al., 2018; Gaziano et al., 2018; McNeil et al., 2018) (163,840 participants) examined cardiovascular mortality; aspirin use was not significantly associated with cardiovascular mortality reduction (RR: 0.95, 95% CI: 0.87–1.03; *p* = 0.23; RD: 0.02%; AR%: 1.91%; NNT = 4,348), and there was no significant heterogeneity (I^2^ = 0; *p* = 0.57) (Figure 2).

### The Secondary Efficacy Outcomes

Regarding the secondary efficacy outcomes, fourteen studies (Peto et al., 1988; Steering Committee of the Physicians’ Health Study Research Group, 1989; The Medical Research Council’s General Practice Research Framework, 1998; Hansson et al., 1998; de Gaetano, 2001; Ridker et al., 2005; Belch et al., 2008; Ogawa et al., 2008; Fowkes et al., 2010; Ikeda et al., 2014; Saito et al., 2017; Bowman et al., 2018; Gaziano et al., 2018; McNeil et al., 2018) with 163,840 individuals revealed that aspirin intake was associated with a 13% reduction in all MIs (RR: 0.87, 95% CI: 0.77–0.97; *p* = 0.02; RD: 0.21%; AR%: 11.11%; NNT = 488), and there was significant heterogeneity (I^2^ = 58%; *p* < 0.01). Eleven studies (Peto et al., 1988; Steering Committee of the Physicians’ Health Study Research Group, 1989; The Medical Research Council’s General Practice Research Framework, 1998; de Gaetano, 2001; Ridker et al., 2005; Ogawa et al., 2008; Fowkes et al., 2010; Ikeda et al., 2014; Saito et al., 2017; Bowman et al., 2018; McNeil et al., 2018) (131,228 individuals) revealed that aspirin intake was associated with a 12% risk reduction in ischemic stroke (RR: 0.88, 95% CI: 0.80–0.96; *p* < 0.01; RD: 0.21%; AR%: 16.14%; NNT = 476), and there was no significant heterogeneity (I^2^ = 0; *p* = 0.62). Fourteen studies (Peto et al., 1988; Steering Committee of the Physicians’ Health Study Research Group, 1989; The Medical Research Council’s General Practice Research Framework, 1998; Hansson et al., 1998; de Gaetano, 2001; Ridker et al., 2005; Belch et al., 2008; Ogawa et al., 2008; Fowkes et al., 2010; Ikeda et al., 2014; Saito et al., 2017; Bowman et al., 2018; Gaziano et al., 2018; McNeil et al., 2018) (163,840 individuals) revealed that aspirin use was not significantly associated with total stroke (RR: 0.94, 95% CI: 0.88–1.02; *p* = 0.13; RD: 0.09%; AR%: 5.30%; NNT = 1,111), and there was no significant heterogeneity (I^2^ = 0; *p* = 0.59).

Furthermore, we explored the cancer outcomes. Ten studies (Peto et al., 1988; Hansson et al., 1998; de Gaetano, 2001; Ridker et al., 2005; Belch et al., 2008; Ogawa et al., 2008; Fowkes et al., 2010; Ikeda et al., 2014; Bowman et al., 2018; McNeil et al., 2018) including 124,523 participants and 12 studies (Peto et al., 1988; Steering Committee of the Physicians’ Health Study Research Group, 1989; The Medical Research Council’s General Practice Research Framework, 1998; Hansson et al., 1998; de Gaetano, 2001; Ridker et al., 2005; Belch et al., 2008; Ogawa et al., 2008; Fowkes et al., 2010; Ikeda et al., 2014; Bowman et al., 2018; McNeil et al., 2018) including 149,134 participants reported cancer incidence and cancer mortality, respectively. There was no significant difference in cancer incidence (RR: 1.00, 95% CI: 0.95–1.06; *p* = 0.87; RD: 0.02%; AR%: 0.28%; NNT = 5,000) or cancer mortality (RR: 1.03, 95% CI: 0.94–1.12; *p* = 0.87; RD: 0.07%; AR%: 3.41%; NNT = 1,449) between the aspirin use and no aspirin use groups, and there was no significant heterogeneity (I^2^ = 36%, *p* = 0.12; I^2^ = 21%, *p* = 0.24, respectively). Aspirin showed the potential to increase the risk of cancer mortality (Supplementary Figure S2).

### The Safety Profile Outcomes

**FIGURE 3:**
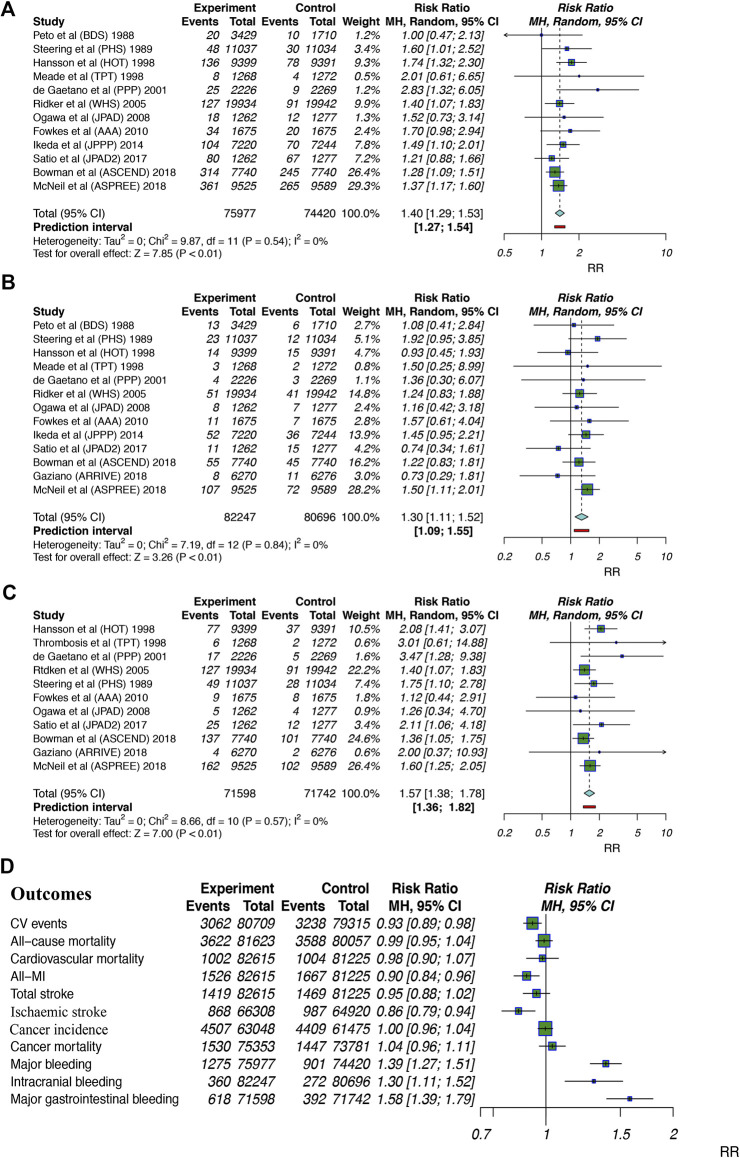
Summary forest plots for the outcomes of bleeding. **(A)** Forest plot for major bleeding. **(B)** Forest plot for intracranial bleeding. **(C)** Forest plot for major gastrointestinal bleeding. **(D)** Forest plot for summarized outcomes analyzed in the current study. MI, myocardial infarction; 95% CI, 95% confidence interval.

Safety profiles outcomes included major bleeding, intracranial bleeding and major gastrointestinal bleeding. Twelve studies (Peto et al., 1988; Steering Committee of the Physicians’ Health Study Research Group, 1989; The Medical Research Council’s General Practice Research Framework, 1998; Hansson et al., 1998; de Gaetano, 2001; Ridker et al., 2005; Ogawa et al., 2008; Fowkes et al., 2010; Ikeda et al., 2014; Saito et al., 2017; Bowman et al., 2018; McNeil et al., 2018) including 150,397 patients examined major bleeding events; aspirin use was found to significantly increase the risk of major bleeding by 40% (RR: 1.40, 95% CI: 1.29–1.53; *p* < 0.01; RD: 0.47%; AR%: 27.85%; NNT = 214), and there was no significant heterogeneity (I^2^ = 0%; *p* = 0.54). Thirteen studies (Peto et al., 1988; Steering Committee of the Physicians’ Health Study Research Group, 1989; The Medical Research Council’s General Practice Research Framework, 1998; Hansson et al., 1998; de Gaetano, 2001; Ridker et al., 2005; Ogawa et al., 2008; Fowkes et al., 2010; Ikeda et al., 2014; Saito et al., 2017; Bowman et al., 2018; Gaziano et al., 2018; McNeil et al., 2018) (162,934 participants) examined intracranial bleeding; aspirin use was associated with a 30% increase in intracranial bleeding (RR: 1.30, 95% CI: 1.11–1.52; *p* < 0.01; RD: 0.10%; AR%: 22.99%; NNT = 1,000), and there was no heterogeneity (I^2^ = 0%; *p* = 0.84). Eleven trials (Steering Committee of the Physicians’ Health Study Research Group, 1989; The Medical Research Council’s General Practice Research Framework, 1998; Hansson et al., 1998; de Gaetano, 2001; Ridker et al., 2005; Ogawa et al., 2008; Fowkes et al., 2010; Saito et al., 2017; Bowman et al., 2018; Gaziano et al., 2018; McNeil et al., 2018) (143,340 participants) examined major gastrointestinal bleeding; aspirin intake was associated with a 57% increase in major gastrointestinal bleeding (RR: 1.57, 95% CI: 1.38–1.78; *p* < 0.01; RD: 0.32%; AR%: 36.70%; NNT = 315), and there was no heterogeneity (I^2^ = 0%; *p* = 0.57). The finding that aspirin use significantly increased the risk of bleeding events led us to identify the proper indicators for balancing the benefits and harm of clinical routines (Figure 3).

### Subgroup Analysis for Further Clinical Implications

Subgroups involving region, mean age, mean BMI, aspirin dosage in the intervention arm and 10-y MACE% were constructed, and subgroup analyses were performed (Table 2). We observed that populations with a dosage of ≤100 mg/day experienced more benefits with respect to CV events, MI, total stroke and ischemic stroke than those with a dosage >100 mg/day. Individuals with a BMI ≧ 25 seemed experience more aspirin-induced benefits with respect to cardiovascular and cerebrovascular outcomes (CV events, RR: 0.91, 95% CI: 0.86–0.98; total stroke, RR: 0.90, 95% CI: 0.82–0.99; ischemic stroke, RR: 0.85, 95% CI: 0.76–0.95) than individuals with a BMI < 25 with similar bleeding events. Aspirin-induced cardiovascular benefits were consistently found in participants with a mean age < 65 y; however, they were not as robust in the patients with a mean age ≥ 65 y, with only one statistically significant outcome for CV events (RR: 0.90, 95% CI: 0.81–1.00). Participants with a low 10-y MACE% risk had the potential to obtain more cardiovascular advantages from aspirin use than those with a high 10-y MACE% risk. There was no significant difference in cardiovascular outcomes and bleeding events between patients from different regions. Across the subgroup analyses, aspirin still had no statistically significant effects on cancer incidence or mortality. All of the above results are presented in Table 2.

**TABLE 2:** Summarized results of total and subgroup analyses.

Items/Outcomes^b^	Total	By region				By mean age (y)		By mean BMI		By aspirin dose (mg)		By 10y-MACE%^a^	
		North America	Europe	Asia	Multiple nations	<65	≧65	<25	≧25	≤100	>100	Low risk	High risk
CV events	0.91 (0.87–0.96)	0.88 (0.80–0.97)	0.94 (0.86–1.03)	0.97 (0.85–1.10)	0.90 (0.82–0.98)	0.92 (0.87–0.97)	0.90 (0.81–1.00)	0.91 (0.84–0.99)	0.91 (0.86–0.98)	0.92 (0.87–0.97)	0.91 (0.75–1.10)	0.89 (0.84–0.96)	0.94 (0.87–1.01)
All-cause mortality	0.97 (0.93–1.02)	0.95 (0.87–1.05)	0.94 (0.88–1.01)	0.98 (0.84–1.13)	1.03 (0.91–1.17)	0.95 (0.90–1.00)	1.06 (0.95–1.18)	0.94 (0.87–1.03)	0.99 (0.92–1.06)	0.98 (0.93–1.03)	0.93 (0.81–1.06)	1.00 (0.92–1.08)	0.94 (0.88–1.01)
Cardiovascular mortality	0.95 (0.87–1.03)	0.96 (0.79–1.17)	0.97 (0.85–1.11)	0.76 (0.31–1.90)	0.90 (0.77–1.07)	0.96 (0.88–1.06)	0.82 (0.53–1.29)	0.97 (0.84–1.12)	0.92 (0.83–1.03)	0.94 (0.85–1.03)	0.99 (0.80–1.23)	0.91 (0.79–1.04)	0.96 (0.85–1.08)
All MI	0.87 (0.77–0.97)	0.78 (0.45–1.34)	0.95 (0.86–1.05)	0.89 (0.69–1.16)	0.81 (0.66–1.01)	0.87 (0.76–1.00)	0.90 (0.75–1.08)	0.78 (0.61–0.99)	0.93 (0.86–1.02)	0.91 (0.83–0.99)	0.78 (0.44–1.38)	0.81 (0.66–1.00)	0.90 (0.79–1.02)
Total stroke	0.94 (0.88–1.02)	0.99 (0.69–1.43)	0.89 (0.78–1.01)	0.99 (0.82–1.18)	1.00 (0.87–1.14)	0.94 (0.86–1.02)	0.97 (0.84–1.13)	1.04 (0.92–1.17)	0.90 (0.82–0.99)	0.92 (0.85–1.00)	1.16 (0.94–1.44)	0.97 (0.86–1.11)	0.94 (0.84–1.05)
Ischemic stroke	0.88 (0.80–0.96)	0.91 (0.64–1.29)	0.89 (0.76–1.03)	0.88 (0.71–1.10)	0.89 (0.72–1.11)	0.88 (0.78–1.00)	0.88 (0.74–1.04)	0.98 (0.82–1.16)	0.85 (0.76–0.95)	0.85 (0.78–0.94)	1.14 (0.86–1.52)	0.87 (0.76–0.98)	0.91 (0.79–1.05)
Cancer incidence	1.00 (0.95–1.06)	1.01 (0.94–1.08)	0.98 (0.91–1.06)	1.06 (0.79–1.42)	1.01 (0.94–1.09)	0.99 (0.94–1.04)	1.05 (0.92–1.21)	1.02 (0.88–1.19)	1.01 (0.97–1.06)	1.02 (0.96–1.07)	0.91 (0.77–1.08)	1.05 (0.98–1.13)	0.97 (0.91–1.04)
Cancer mortality	1.03 (0.94–1.12)	1.00 (0.84–1.18)	0.94 (0.84–1.05)	1.07 (0.88–1.30)	1.18 (0.94–1.48)	0.97 (0.89–1.05)	1.19 (1.04–1.36)	1.03 (0.90–1.18)	1.04 (0.91–1.19)	1.03 (0.95–1.12)	0.97 (0.68–1.40)	1.11 (0.96–1.27)	0.96 (0.87–1.07)
Major bleeding	1.40 (1.29–1.53)	1.44 (1.15–1.82)	1.46 (1.10–1.95	1.35 (1.10–1.67)	1.49 (1.18–1.88)	1.39 (1.21–1.59)	1.42 (1.25–1.62)	1.47 (1.26–1.71)	1.36 (1.21–1.53)	1.39 (1.28–1.52)	1.40 (0.92–2.12)	1.42 (1.27–1.60)	1.36 (1.20–1.54)
Intracranial bleeding	1.30 (1.11–1.52)	1.40 (0.96–2.05)	1.26 (0.91–1.74)	1.21 (0.82–1.77)	1.18 (0.77–1.80)	1.18 (0.96–1.47)	1.46 (1.15–1.84)	1.25 (0.95–1.65)	1.31 (1.08–1.60)	1.28 (1.08–1.51)	1.57 (0.89–2.77	1.40 (1.15–1.70)	1.12 (0.85–1.47)
Major gastrointestinal bleeding	1.57 (1.38–1.78)	1.47 (1.17–1.86)	1.61 (1.02–2.54)	1.87 (1.02–3.44)	1.72 (1.40–2.11)	1.58 (1.35–1.85)	1.58 (1.24–2.01)	1.92 (1.47–2.51)	1.49 (1.28–1.72)	1.55 (1.36–1.77)	1.75 (1.10–2.78)	1.57 (1.33–1.85)	1.57 (1.28–1.93)

Abbreviations: BMI, body mass index; MACE, major adverse cardiovascular event rate; CV event, cardiovascular event; MI, myocardial infraction.

^a^ A 10-y MACE% of at least 10% was regarded as high CV risk and less than 10% was low.

^b^ All the outcomes were shown in RR and 95% CI form.

### Sensitivity Analysis

In sensitivity analyses, many variables were classified into different subgroups. To better eliminate bias and heterogeneous interactions (TPT (The Medical Research Council’s General Practice Research Framework, 1998) trial was excluded for warfarin use), we used the inverse variance (IV) statistical method. Most of the results were consistent with the primary results and remained robust through sensitivity analyses. Interestingly, we observed increased aspirin-induced benefits for cardiac outcomes (CV events, RR: 0.90, 95% CI: 0.85–0.95; all MI, RR: 0.83, 95% CI: 0.72–0.96; ischemic stroke, RR: 0.86, 95% CI: 0.76–0.97) among trials with diabetic and nondiabetic patients compared to the trials involving only diabetic patients. We also observed aspirin-induced benefits when excluding patients with asymptomatic peripheral artery disease (PAD). Furthermore, after excluding trials published before 2000, the cardiovascular benefits were still obvious. No effects on cancer were found across sensitivity analyses (Table 3). The omission process as well as the results of the heterogeneity analyses can be found in Table 3 and Supplementary Material S2–S12.

**TABLE 3:** Summarized results of the sensitivity analysis.

Outcomes (RR, 95% CI)	Excluding before 2000 trials^a^	Excluding open-label trials^b^	Excluding high risk trials^c^	Excluding asymptomatic PAD trials^d^	Excluding 100% male individual trials^e^	Excluding 100% diabetic individuals trials^f^	Restricting on 100% diabetic individuals trials^g^	Excluding placebo use trials^h^	Excluding TPT study^i^
Primary efficacy outcomes									
CV Events	0.91 (0.87–0.96)	0.90 (0.85–0.95)	0.91 (0.86–0.97)	0.91 (0.87–0.96)	0.92 (0.87–0.97)	0.90 (0.85–0.95)	0.95 (0.84–1.06)	0.92 (0.84–1.02)	NA
All-cause mortality	0.98 (0.93–1.04)	0.98 (0.93–1.03)	0.98 (0.92–1.04)	0.97 (0.93–1.02)	0.98 (0.93–1.03)	0.98 (0.93–1.04)	0.94 (0.86–1.03)	0.93 (0.83–1.03)	0.97 (0.93–1.02)
Cardiovascular mortality	0.93 (0.82–1.07)	0.95 (0.87–1.05)	0.95 (0.85–1.05)	0.93 (0.85–1.02)	0.94 (0.84–1.04)	0.94 (0.85–1.05)	0.97 (0.65–1.45)	0.85 (0.59–1.22)	0.95 (0.86–1.03)
Secondary efficacy outcomes									
All MI	0.95 (0.88–1.03)	0.86 (0.74–0.99)	0.93 (0.83–1.04)	0.84 (0.74–0.95)	0.92 (0.84–1.00)	0.83 (0.72–0.96)	0.97 (0.85–1.10)	0.94 (0.79–1.12)	0.88 (0.78–0.99)
Total stroke	0.92 (0.84–1.00)	0.94 (0.86–1.03)	0.92 (0.84–1.00)	0.95 (0.89–1.03)	0.92 (0.85–1.00)	0.96 (0.88–1.05)	0.90 (0.88–1.02)	0.98 (0.84–1.15)	0.95 (0.88–1.02)
Ischemic stroke	0.86 (0.78–0.94)	0.88 (0.78–0.98)	0.85 (0.76–0.95)	0.88 (0.80–0.97)	0.86 (0.78–0.98)	0.86 (0.76–0.97)	0.92 (0.79–1.07)	0.89 (0.72–1.09)	0.88 (0.81–0.97)
Cancer incidence	1.01 (0.94–1.08)	0.99 (0.95–1.05)	0.99 (0.95–1.05)	1.02 (0.97–1.07)	1.00 (0.94–1.07)	1.02 (0.95–1.10)	0.94 (0.82–1.08)	1.06 (0.90–1.25)	NA
Cancer mortality	1.03 (0.93–1.15)	1.03 (0.92–1.15)	1.02 (0.89–1.16)	1.05 (0.96–1.14)	1.03 (0.94–1.12)	1.04 (0.93–1.16)	0.98 (0.86–1.12)	1,01 (0.86–1.19)	1.03 (0.94–1.12)
Safety outcomes									
Major bleeding	1.37 (1.12–1.50)	1.40 (1.28–1.54)	1.39 (1.26–1.53)	1.40 (1.28–1.52)	1.40 (1.28–1.54)	1.48 (1.33–1.64)	1.27 (1.11–1.47)	1.42 (1.11–1.80)	1.40 (1.29–1.52)
Intracranial bleeding	1.30 (1.10–1.54)	1.33 (1.11–1.59)	1.29 (1.07–1.56)	1.29 (1.10–1.52)	1.28 (1.08–1.51)	1.36 (1.14–1.63)	1.11 (0.80–1.54)	1.22 (0.89–1.68)	1.30 (1.11–1.52)
Gastrointestinal bleeding	1.49 (1.30–1.72)	1.52 (1.33–1.74)	1.51 (1.38–1.78)	1.58 (1.39–1.80)	1.55 (1.36–1.77)	1.63 (1.41–1.90)	1.43 (1.13–1.80)	2.23 (1.33–3.74)	1.56 (1.38–1.78)

Note: Sensitivity analysis was conducted by omitting one/several study/studies each turn to show more clinical useful data.

Abbreviations: MI, myocardial infraction; PAD, peripheral artery disease; NA, Not available; RR, Relative risk; CI, Confidence interval.

^a^ Total 10 trials (de Gaetano, 2001; Ridker et al., 2005; Belch et al., 2008; Ogawa et al., 2008; Fowkes et al., 2010; Ikeda et al., 2014; Saito et al., 2017; Bowman et al., 2018; Gaziano et al., 2018; McNeil et al., 2018), N = 115,300.

^b^ Total nine trials (Steering Committee of the Physicians’ Health Study Research Group, 1989; The Medical Research Council’s General Practice Research Framework, 1998; Hansson et al., 1998, Ridker et al., 2005; Belch et al., 2008; Fowkes et al., 2010; Bowman et al., 2018; Gaziano et al., 2018; McNeil et al., 2018), N = 135,042.

^c^ Total seven trials (Hansson et al., 1998; Ridker et al., 2005; Belch et al., 2008; Fowkes et al., 2010; Bowman et al., 2018; Gaziano et al., 2018; McNeil et al., 2018), N = 110,432.

^d^ Total 12 trials (Peto et al., 1988; Steering Committee of the Physicians’ Health Study Research Group, 1989; The Medical Research Council’s General Practice Research Framework, 1998; Hansson et al., 1998; de Gaetano, 2001; Ridker et al., 2005; Ogawa et al., 2008; Ikeda et al., 2014; Saito et al., 2017; Bowman et al., 2018; Gaziano et al., 2018; McNeil et al., 2018); N = 159,214.

^e^ Total 11 trials (Hansson et al., 1998; de Gaetano, 2001; Ridker et al., 2005; Belch et al., 2008; Ogawa et al., 2008; Fowkes et al., 2010; Ikeda et al., 2014; Saito et al., 2017; Bowman et al., 2018; Gaziano et al., 2018; McNeil et al., 2018), N = 134,090.

^f^ Total 10 trials (Peto et al., 1988; Steering Committee of the Physicians’ Health Study Research Group, 1989; The Medical Research Council’s General Practice Research Framework, 1998; Hansson et al., 1998; de Gaetano, 2001; Ridker et al., 2005; Fowkes et al., 2010; Ikeda et al., 2014; Gaziano et al., 2018; McNeil et al., 2018), N = 142,385.

^g^ Total four trials (Belch et al., 2008; Ogawa et al., 2008; Saito et al., 2017; Bowman et al., 2018), N = 21,455.

^h^ Total five trials (Peto et al., 1988; de Gaetano, 2001; Ogawa et al., 2008; Ikeda et al., 2014; Saito et al., 2017), N = 28,797.

^i^ Total 13 trials (Peto et al., 1988; Steering Committee of the Physicians’ Health Study Research Group, 1989; Hansson et al., 1998; de Gaetano, 2001; Ridker et al., 2005; Belch et al., 2008; Ogawa et al., 2008; Fowkes et al., 2010; Ikeda et al., 2014; Saito et al., 2017; Bowman et al., 2018; Gaziano et al., 2018; McNeil et al., 2018), N = 161,300.

These findings implied that aspirin use among diabetic individuals may not lead to the primary prevention of CVD because diabetes, which is known as a risk factor for CVD, might indirectly enhance the CV risk estimated by the MACE; similarly, the efficacy of aspirin use in studies including both diabetic and nondiabetic patients was excellent. Second, diagnosis technology is developing over time, which means that more patients with potential or asymptomatic CVD could be properly diagnosed and excluded before entering clinical trials or taking aspirin for “primary prevention”. Therefore, the preferable role of aspirin in the primary prevention of CVD would be highlighted, especially in recently published studies (after 2000). Finally, early screening for PAD was equally important to help identify individuals who may not benefit from aspirin.

### Trial Sequential Analysis

**FIGURE 4:**
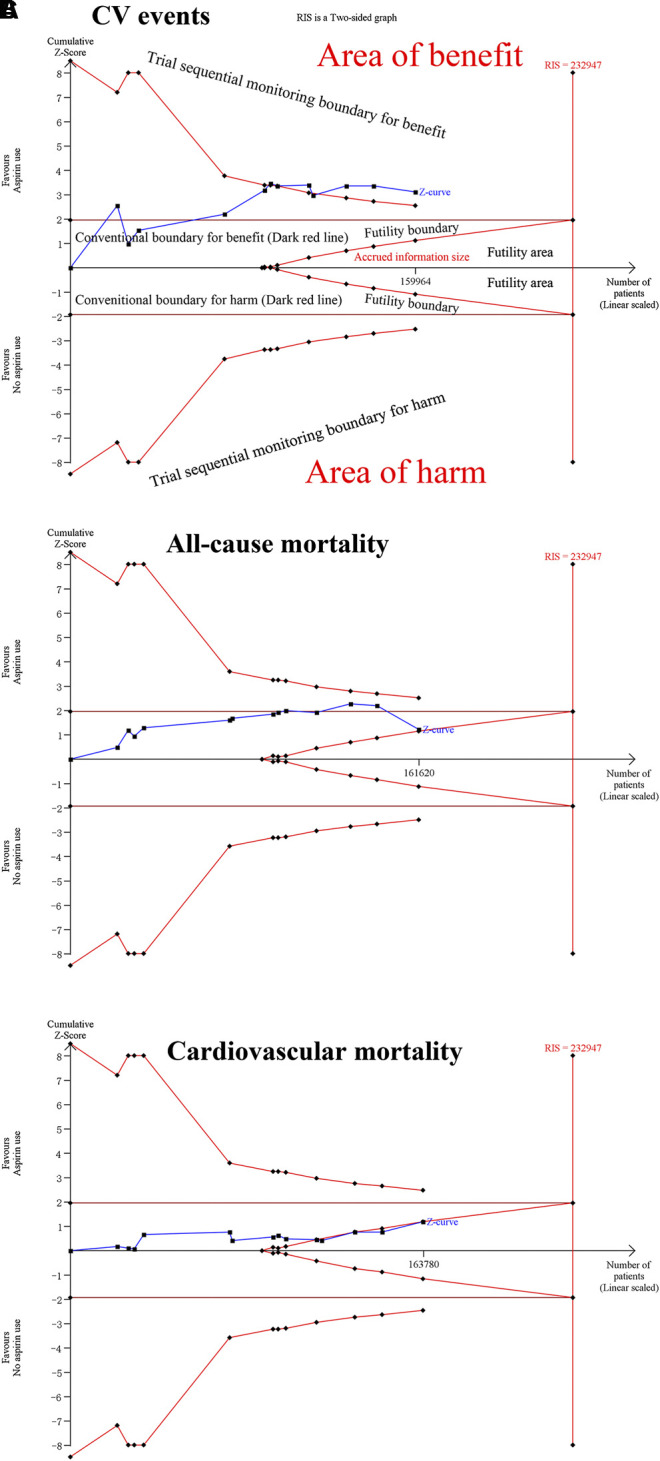
Trial sequential analysis of CV events, all-cause mortality, and cardiovascular mortality under 5% relative risk reduction, 5% for two-sided type 1 error risk, 80% statistical power and 5% control event incidence conditions. **(A)** For CV events. **(B)** For all-cause mortality. **(C)** For cardiovascular mortality.

In TSA, we observed the Z-curve cross the trial sequential analysis boundary (TSA boundary) for CV events, all MI, ischemic stroke, major bleeding, intracranial bleeding and major gastrointestinal bleeding outcomes under conditions of 5% relative risk reduction, 5% for two-sided type 1 error risk, 80% statistical power and 5% control event incidence. The Z-curve did not cross the traditional boundary or the TSA boundary but crossed the futility boundary for cardiovascular mortality. The Z-curve crossed the traditional and futility boundaries but did not cross the TSA boundary for all-cause mortality. These findings showed that conclusions on the abovementioned outcomes were robust and were hardly modified with additional related trials. However, the Z-curve did not cross the TSA boundary or the futility boundary for total stroke, cancer incidence and cancer mortality, which suggested that additional studies should be conducted to evaluate those effects (Figure 4; Supplementary Figure S3).

Egger’s test revealed no significant publication bias for CV events (*p* = 0.882), all-cause mortality (*p* = 0.362), CV mortality (*p* = 0.390), major bleeding (*p* = 0.126), intracranial bleeding (*p* = 0.236), or major gastrointestinal bleeding (*p* = 0.152) (Supplementary Figure S4).

## Discussion

As one of the most widely used drugs worldwide, aspirin celebrated its 121st birthday in 2020 and the remarkable store is still going on (Vranckx et al., 2018). In this study, aspirin was observed to be significantly associated with a 9, 13, and 12% reduction in the risk of CV events, all-MI and ischemic stroke, respectively; however, aspirin was associated with a 40, 30, and 57% increase in the risk of bleeding profiles, including major bleeding, intracranial bleeding and major gastrointestinal bleeding, respectively. No causal outcomes were found in all-cause mortality, cardiovascular mortality, total stroke, cancer incidence or cancer mortality. Low doses of aspirin (≤100 mg) might offer more clinical benefits than high doses of aspirin; individuals who are <65 y old and have a BMI ≥ 25 demonstrated stronger effects of aspirin on the primary prevention of CVD; the data indicated that aspirin did not confer benefits in the high 10-y MACE% risk group. The results were not significantly modified after excluding asymptomatic PAD trials and trials with only diabetic individuals. Besides recommendations from contemporary guidelines, we hypothesized that aspirin might be prescribed depending on body size (BMI), that is, individuals with varied BMI should take different dose of aspirin, for we observing significant differences between <25 and ≧25 BMI, ≤100 and >100 aspirin intake groups on few intended CV outcomes (Rothwell et al., 2018). It is still crucial to perform complete screening and examinations on large populations to evaluate populations’ CVD risk, hence quantifying their probability of obtaining real benefits from aspirin. This study provides further insights through updated data on comprehensive subgroup and sensitivity analyses to display potential utility on CVD primary prevention. Indeed, the one-dose-fits-all intake strategy is unlikely optimal, and a more tailored and wise dosing approach is called for to maximize substantial benefits and reduce potential risk.

The endorsed role of aspirin in the primary prevention of ischemic events (all-MI, ischemic stroke) has been supported by several studies (Fox et al., 2015b). The potential mechanism for preventing ischemic events is based on the inhibition of thrombus propagation and plaque rupture (Cleland, 2013). This study also suggested a beneficial role of aspirin in all-MI and ischemic stroke outcomes. Notably, only two eligible trials (HOT and PHS) (Steering Committee of the Physicians’ Health Study Research Group, 1989; Hansson et al., 1998) exhibited significant risk reduction in all-MI; however, their conducting time was rather early, and no significant risk reduction was observed in cardiovascular mortality and all-cause mortality under the long follow-up period. Because the two trials were conducted early, researchers could not properly emphasize the biases from risk factors such as smoking status, blood glucose, blood cholesterol level or blood pressure. Another concern is that almost 50% of MIs are considered to be clinically silent; accordingly, it is not easy to ascertain the clinical benefit from long-term aspirin use through this endpoint (Zhang et al., 2016). It may be that all CV events are assessed to be proper endpoints to evaluate all these cases. Some studies have suggested that populations with substantially increased CVD risk may benefit from preventive aspirin use, and guidelines from the US Preventive Services Task Force also suggested prescribing low doses of aspirin in adults aged 50–59 years with a CVD risk of at least 10% (Guirguis-Blake et al., 2016), which was in contrast to our findings that low-risk individuals seemed to obtain more clinical benefits. We used the 10-y MACE% to reflect participants’ CVD risk and hypothesized that the CVD risk of participants tended to be overestimated due to the lack of agreement on unified risk calculators in primary trials (Rana et al., 2016). For example, the ARRIVE trial (Gaziano et al., 2018) mixed predicted and observed CVD risk, such that the enrolled moderate risk populations had a standard risk of 17.3% as estimated by American Heart Association (AHA)/American College of Cardiology (ACC) 10-y CV risk estimated criteria (Allan et al., 2013; Rana et al., 2016) but had an observed CVD risk rate of 6.9%. Similarly, the ASPREE trial (McNeil et al., 2018) enrolled patients who were older than 65 or 70 y old; the CVD risk of these older patients was hard to evaluate, and the reported 10-y MACE% of 7.8% differed from the 8.3% figure found herein, although both 10-y MACE% were less than 10%. The reason for this discrepancy was that MACE in the ASPREE trial was defined as a composite of fatal coronary heart disease, nonfatal MI and fatal or nonfatal ischemic stroke, which differed from the unified definition. In this study, CV event risk was reduced by 11% in the low 10-y MACE% risk group.

Guidelines driven by the AHA/American Diabetes Association (ADA) recommend aspirin use in diabetic populations with intermediate risk (5–10% 10-y MACE%) for primary prevention (Fox et al., 2015b). JPAD (Ogawa et al., 2008) and ASCEND (Bowman et al., 2018) trials specifically incorporated diabetic populations, but the cardiovascular benefits seemed to be higher in the ASCEND trial. The total proportion of statin use was 75% in the ASCEND trial vs. 25% in the JPAD trial, which might have resulted in higher benefits seen in the ASCEND trial. Additionally, this study indicated fewer CVD benefits among populations with diabetes, which was supported by recent European Society of Cardiology guidelines recommending against aspirin use in diabetic populations who have no history of CVD (Piepoli et al., 2016). Routine aspirin use was not enough for primary prevention among individuals with a high risk of CVD; at that time, blood pressure and blood glucose were controlled, cholesterol levels were reduced with statins, and physical activity and healthy eating were reduced are also necessary. Aspirin use increased the risk of bleeding profiles but was not associated with cardiovascular mortality considering that deaths caused by bleeding were rare. Since the strategy to reduce harm of long-term aspirin use is not understood from current evidence, prescribing proton pump inhibitors (PPIs) might limit the risk of major gastrointestinal bleeding and enhance the benefit-risk ratio toward intended populations (Fowkes et al., 2010). Aspirin appears to be not associated with all-cause mortality; however, several trials revealed that aspirin reduced the risk of colorectal cancer (RR: 0.73, 95% CI: 0.69–0.78), squamous-cell oesophageal cancer (RR: 0.67, 95% CI: 0.57–0.79), gastric cancer (RR: 0.64, 95% CI: 0.51–0.82) and pancreatic cancer (RR: 0.78, 95% CI: 0.68–0.89) (Bosetti et al., 2020). At this time, the reduction in cancer mortality appeared after 5 y of follow-up, and this result was not duplicated in the ASCEND trial (Bowman et al., 2018). Current findings suggest a neutral role of aspirin in cancer outcomes; therefore, no suggestions could be made regarding benefit-risk balance from current evidence.

### Added Value and Limitations

Contrast to prior similar studies, current study has several innovations. Mahmoud et al. (Mahmoud et al., 2019) conducted a TSA meta-analysis, the authors mainly focused on CVD-related outcomes including all-cause mortality, all MI, bleeding events. Comparing to Mahmoud et al. (Mahmoud et al., 2019), current study is more comprehensive because we also investigated cancer outcomes. Study from Mahmoud et al. (Mahmoud et al., 2019) included 11 RCTs, in our prospective, it was not enough, trials like POPADAD (Belch et al., 2008), AAA (Fowkes et al., 2010) were not reasonably included. Also, several 10y-MACE% values presented in that study were not in consistent with current study, for example ASCEND (Bowman et al., 2018), ARRIVE (Gaziano et al., 2018) and ASPREE (McNeil et al., 2018). 10y-MACE% for BDS (Peto et al., 1988) and TPT (The Medical Research Council’s General Practice Research Framework, 1998) was also absent in Mahmoud et al. (Mahmoud et al., 2019) study. Lin et al. (Lin et al., 2019) investigated the role of low-dose of aspirin on CVD primary prevention, they demonstrated low-dose aspirin had no role in all MI, but did reduce stroke incidence, which was in contrast to findings from current paper (that aspirin might significantly reduce all MI incidence instead of total stroke, ischemic stroke could be reasonably reduced). Current study had included more comprehensive RCTs than Lin et al. (Lin et al., 2019), subgroup analyses aiming to low-dose of aspirin (<100 mg/d) were also conducted. This study clearly pinpointed low CVD risk individuals might get more clinical benefits than the high risk from aspirin. Only one TSA for MACE outcome in Lin et al. (Lin et al., 2019) was far enough to draw robust conclusions. Major controversial issues from current study and Gelbenegger et al. (Gelbenegger et al., 2019) were the outcomes on diabetic populations, this study supported there were no substantial benefits of aspirin on diabetic populations primary prevention. POPADAD (Belch et al., 2008), JPAD (Ogawa et al., 2008), JPAD2 (Saito et al., 2017) and ASCEND (Bowman et al., 2018) were special trials conducted on full diabetic populations (100% diabetic individuals), to our great knowledge, it was more proper to investigate the intended results on the four trials, data stem from calculation on other small diabetic-proportion trials (Ridker et al., 2005; Ikeda et al., 2014) would add extra reporting bias. Zheng et al. (Zheng and Roddick, 2019) also performed a similar research, however, no TSA results were revealed and merits from network meta-analysis methods seemed not so obvious. Overall, current study with particular subgroup and sensitivity analyses clearly addressed the less priority of aspirin on high 10y-MACE% risk and diabetic populations, such populations may need more aggressive therapy or combined pharmaceutical intervention. We believe these results add new evidence to the discussion on aspirin primary prevention in CVD and may arouse new disputes.

Limitations were also detected. First, definitions of reported outcomes were different, reflecting advances in CVD diagnosis and treatment. To best overcome this heterogeneity, we defined unified primary and secondary efficacy outcomes and safety profiles and then properly extracted the required data in eligible studies. Second, aspirin use in the included studies was not consistent with the major dose of 75–100 mg. Importantly, more clinical benefits with bleeding risk were found in trials restricted to ≤100 mg/d intake. Third, several trials (BDS (1998), PHS (1989), TPT (1998), HOT (1998)) were published rather early, and thus, some examinations and screening methods may not have been as accurate as expected. This contributed to an overestimated 10-y MACE%. Long-term follow-up studies are welcomed to better characterize individuals who may benefit from aspirin for primary prevention outweighing the unexpected bleeding events. Objective influence on all-cause mortality and cancer incidence should be re-evaluated. Considering no individual-patients-data was involved, therefore, a more precise study based on individual data is quite encouraged.

## Conclusion

Aspirin intake was associated with reduced risk of CV events, all MI, and ischemic stroke, and was associated with increased incidences of major bleeding, intracranial bleeding, and major gastrointestinal bleeding in the primary prevention of CVD. The use was not associated with an increased risk of all-cause mortality, cardiovascular mortality, total stroke, cancer incidence or cancer mortality. No substantial benefits with respect to CVD were observed in the diabetic and high 10-y MACE% risk group populations. A one-dose-fits-all strategy is not optimal, and BMI may be a potential indicator to guide aspirin prescription. It is also necessary to identify individuals who may benefit from aspirin by more accurate cardiovascular-relating examinations. Overall, the benefits and harm of aspirin for primary prevention should be re-evaluated. Based on these findings, we believe it is not yet the time to quit the aspirin era.

## Data Availability Statement

The original contributions presented in the study are included in the article/Supplementary Material, further inquiries can be directed to the corresponding author.

## Author Contributions

All authors designed and conducted this review. BZ wrote the paper. QW, LW, CL, YD, JX, YW, and WZ helped the study design. BZ, YW, and WZ revised the statistical methodology. BZ and WZ had primary responsibility for the final content. All authors read and approved the final manuscript. Notably, BZ and WZ equally share the corresponding authorship.

## Funding

This study was supported by National Natural Science Foundation of China (NSFC), with no commercial entity involved (grant no 81560345). The NSFC had no role in the design and conduct of the study; collection, management, analysis, and interpretation of the data; preparation, review, or approval of the manuscript; and decision to submit the manuscript for publication.

## Conflict of Interest

The authors have completed and submitted the ICMJE Form for Disclosure of Potential Conflicts of Interest. No other disclosures were reported.
