# Supplementary material for: Pros and Cons of Aspirin for the Primary Prevention of Cardiovascular Events: A Secondary Study of Trial Sequential Analysis
Source: Front Pharmacol. 2021 Jan 14;11:592116. doi: 10.3389/fphar.2020.592116 (PMC7845480; doi:10.3389/fphar.2020.592116)
Supplement: Supplementary file 1 [file table1.docx]

**Appendix File S1. Appendix Methods**

**Material and Methods**

This article was according to the Preferred Reporting Items for Systematic Reviews and Meta-Analyses. The protocol was registered with PROSPERO (**CRD42019127570**).

**Search strategy**

Relating published trials were identified after a rigorous literature search on PubMed, EMBASE, Cochrane Library, Web of Science and Clinical Trials.gov from inception to Feb 1 2020. The key items used were “aspirin”, “antiplatelet drug”, “cardiovascular disease”, “cardiovascular event”, “coronary heart disease”, “primary prevention”, “randomized controlled trials”. No restrictions were applied on language. Reference lists were searched manually for additional records.

# Comprehensive searches were conducted in four electronic databases:

(1) PubMed/Medline (NLM)

(2) EMBASE (Elsevier)

(3) Cochrane Library (CENTRAL/Wiley)

(4) Web of Science (Medline)

(5) Clinical Trials.gov (NIH)

The literature search strategy was developed first in PubMed and then translated to the other databases. A combination of relevant keywords and controlled vocabulary (MeSH - Medical Subject Headings in PubMed and Emtree in EMBASE) were used in the PubMed and EMBASE searches. Comparable keyword search strategies were used in Cochrane Central Register of Controlled Trials (CENTRAL), Web of Science and Clinical Trials.gov.

No date or language restrictions were applied. Results were limited to Human clinical trials. MEDLINE records were excluded from EMBASE results sets.

# Four component concepts made up the search strategy:

(1) Aspirin

(2) Cardiovascular disease

(3) Primary prevention

(4) RCTs

For search set #4, we used Cochrane Handbook recommended search filters for finding RCTs.

Search filters were used for finding RCTs in PubMed and EMBASE. Available database limiters were used in Cochrane CENTRAL (Trials)

<http://work.cochrane.org/pubmed>
**Sensitivity- and precision-maximizing version (2008 revision); PubMed format**

(randomized controlled trial[pt] OR controlled clinical trial[pt] OR randomized[tiab] OR placebo[tiab] OR clinical trials as topic[mesh:noexp] OR randomly[tiab] OR trial[ti] NOT (animals[mh] NOT humans [mh])

<http://work.cochrane.org/embase>

**Embase search strategy for finding RCTs in Embase**

('crossover procedure':de OR 'double-blind procedure':de OR 'randomized controlled trial':de OR  'single-blind procedure':de OR (random* OR  factorial* OR crossover* OR cross NEXT/1 over* OR placebo* OR doubl* NEAR/1 blind* OR singl* NEAR/1 blind* OR assign* OR allocat* OR volunteer*):de,ab,ti)

Each of the four components of the search strategy was first searched upon individually, combining synonyms describing that concept with the Boolean operator OR. The four individual component search sets were then combined together using the Boolean operator AND.

Resulting citations were managed and duplicates removed using the Endnote citation management software program X8 (Thomson Reuters).

|  | PubMed/MEDLINE Search Strategy |
| --- | --- |
| 1 Aspirin | ("Aspirin"[Mesh] OR " acetylsalicylic acid" OR "acid, acetylsalicylic" OR "2-(Acetyloxy)benzoic Acid" OR "acylpyrin" OR "aloxiprimum" OR "colfarit" OR "dispril" OR "easprin" OR "ecotrin" OR "endosprin" OR "magnecyl" OR "micristin" OR "polopirin" OR "polopiryna" OR "solprin" OR "solupsan" OR "zorprin" OR "acetysal") |
| 2 Cardiovascular disease | ("Cardiovascular disease"[Mesh] OR "disease, cardiovascular" OR "diseases, cardiovascular" OR "cardiovascular events" OR "coronary heart disease" OR "disease, coronary heart" OR "heart disease, coronary" OR "strokes", OR "cerebrovascular accident", OR "CVA (cerebrovascular accident)", OR "cerebrovascular apoplexy", OR "brain vascular accident", OR "acute cerebrovascular accident", OR " acute stroke", OR " cerebral stroke") |
| 3 primary prevention | ("Primary prevention"[Mesh] OR "disease prevention, primary" OR "primary disease prevention" OR " prevention, primary" OR "preventions, primordial" OR "prevention, primordial" OR "primordial prevention" OR "disease preventions, Primary" |
| 4 RCT | (randomized controlled trial[pt] OR controlled clinical trial[pt] OR randomized[tiab] OR placebo[tiab] OR clinical trials as topic[mesh:noexp] OR randomly[tiab] OR trial[ti]) |
| 5 | (animals[mh] NOT humans [mh]) |
| 6 | **1** AND **2** AND **3** AND **4** |
| 7 | **6** NOT **5** |

**Abbreviations:** Mesh: Medical Subject Heading, pt: Publication Type, tiab: Title/Abstract, ti: Title, mh: MeSH Terms.

**Selection Criteria**

Eligible studies had to (1) enroll adult participants (≥ 18 y) without preexisting CV events (CV events here include peripheral arterial disease, CHD, prior myocardial infraction (MI), ischaemic stroke, prior percutaneous coronary intervention, prior coronary artery bypass grafting); (2) compare aspirin to no aspirin use (placebo included); (3) the follow-up is no less than 1 year to confirm the high quality of primary studies; (4) provide reliable and available outcome data (at least one interested primary efficacy outcome was reported); (5) be in RCT form.

Studies with the most comprehensive outcomes were considered for avoiding duplications; the incorporated patients with diabetes while without atherosclerosis were also considered. JPAD^8^ and JPAD2^11^ trials were both included for they having different baseline characteristics and proportion of the involved individuals. We excluded pure basic researches, reviews, and animal experiments.

**Data extraction and outcome definition**

Two authors (Binghao Zhao and Yiping Wei) independently performed study screening and extracted the baseline characteristics of each eligible trial^1-14^. The baseline characteristics include: study populations, number of populations, mean age, male proportion, aspirin use dose, control arm set, proportion of diabetes, current smoking and hypertension, mean systolic blood pressure (SBP), total cholesterol, body mass index (BMI), 10-y major adverse cardiovascular events rate (10-y MACE%) and study follow-up period. The adjusted hazard ratio (HR), odd ratio (OR) and relative risk (RR) of analyzed outcomes were adjusted for fully adjusted models. If some studies used intention-to-treat principles or provided complete intention-to-treat principles data, we would extract and use the intention-to-treat data. Any discrepancies were resolved by the author panel with a third author (Wenbin Ma). If there was any data not available, the original authors would be kindly contacted.

The primary efficacy outcomes were CV events, all-cause mortality and cardiovascular mortality given their universal definitions and balance of efficacy and safety, which would reduce inconsistency among eligible studies. The secondary efficacy outcomes were all MI, total stroke and ischaemic stroke, cancer incidence and cancer mortality. The safety profile outcomes were major bleeding, intracranial bleeding and major gastrointestinal bleeding just as defined by each eligible trial. Intracranial bleeding was treated as a potential outcome of aspirin use other than caused by CV events. All these definitions follow per study’s definition^15^.

Dramatically, the effect of aspirin on cancer incidence and cancer mortality was still controversial with contrasting results yielded from meta-analyses. Some studies even addressed that aspirin added the probability of cancer mortality. Therefore, we prescribed cancer outcomes as exploratory outcomes to generate robust evidence. Major adverse cardiovascular events (MACE) were defined as composite of cardiovascular death, non-fatal MI and non-fatal stroke etc. 10-y MACE% was extracted and calculated by multiplying the annualized event rate for cardiovascular mortality, non-fatal MI, and non-fatal stroke etc. The 10-y MACE% ≥ 10% was regarded as high risk; < 10% was regarded as low risk.

Totally, main definitions of each interested outcome were listed below:

**CV events**: involving all cardiovascular events or disease;

**All-cause mortality**: Any death;

**Cardiovascular mortality**: Death caused by coronary heart disease, stroke, vascular death etc;

**All-MI**: Not specified MI according to WHO criteria;

**Total stroke**: Total stroke includes ischaemic stroke, hemorrhagic stroke and unknown types of stroke;

**Ischaemic stroke**：Not specified ischaemic stroke diagnosed by CT or MRI findings;

**Cancer incidence:** All types of cancer incidence;

**Cancer mortality**: Death caused by all types of cancer;

**Major bleeding**: Major hemorrhage (hemorrhagic stroke, subarachnoid hemorrhage), gastrointestinal or other requiring admission to hospital for intervention to control bleeding events;

**Intracranial bleeding**: Intracranial hemorrhage or subarachnoid hemorrhage;

**Major gastrointestinal bleeding**: Gastrointestinal bleeding (upper or lower gastrointestinal bleeding) requiring admission to hospital for intervention to control bleeding.

**Study Quality Assessment**

Methodological quality assessment was performed by three co-authors. We used Cochrane Risk and Bias Tool recommended by the Cochrane handbook to evaluate the quality of each eligible study^16^. Seven items were used to evaluate heterogeneity in each trial: randomization sequence generation, allocation concealment, blinding of participants and personnel, blinding of outcome assessment, incomplete outcome data, selective reporting, and other biases. The quality of each study was categorized as high, low, or unclear. In case of disagreement, the two authors would recheck the original articles and a consensus would be achieved after a discussion.

**Statistical Analysis**

For descriptive purpose and statistical convenience, weighted frequencies were calculated for categorical variables using the provided sample size of each trial. Multivariable RRs and 95% confidence intervals (95% CIs)^17^ for interested primary/secondary efficacy outcomes, primary safety outcomes were specially estimated using the DerSimonian-Laird (D-L) random-effects model considering the existence of within- and between-study variability. To further illustrate these estimations, risk difference (RD), absolute risk percentage (AR%) and number needed to treat (NNT) were also analyzed. For further statistical purpose, HRs, ORs were considered as RRs in this study. Fully adjusted effect sizes (ESs) were logarithmically transformed to stabilize the variance, hence the data distribution could be normalized.

Between-study heterogeneity and variability was quantified by Cochran’s Q chi-squared test and I^2^, whereby an I^2^ > 50% or a P-value for the Q test < 0.10 was considered to represent significant heterogeneity^18^. For providing more clinical implications, we conducted comprehensive subgroup analyses mainly focusing on several significant variables including: region, individuals’ main age, mean body mass index (BMI), aspirin taken dose and 10-y MACE%; for investigating the influence of individual studies on final results, we carried out sensitivity analyses by omitting one study each turn to see the robustness if there were significant heterogeneity.

Publication bias was assessed by funnel plots and Egger’s test^19^, with P < 0.05 indicating significant bias. All analyses were did using R project software (version 3.5.3, <https://www.r-project.org/>, USA) and other public packages (forest, ggplot2, survminer etc.); two-sided P < 0.05 was considered statistically significant except where otherwise specified.

**Trial sequential analysis**

Previous studies have confirmed the risk of type 1 error from interim analyses can be reasonably reduced through monitoring boundaries and modifying the P-value. Similar in meta-analyses, random errors caused by sparse data and repetitive testing also enhance the risk of type 1 error. Such method setting analogous trial sequential monitoring boundaries to meta-analyses is called trial sequential analysis (TSA), which assist to determine whether evidence is reliable or conclusive^20,21^. Actually, random errors can be rectified and reduced via TSA using TSA software (version 0.9 beta (<http://www.ctu.dk/tsa)>) because it combines estimation of the required information size (RIS) with an adjusted threshold for statistical significance. We assumed that if the Z-curve crossed the TSA boundary or entered the futility area, a sufficient effect was obtained, while further studies were not required; if not, evidence was insufficient. TSA was performed for a 10% relative risk reduction, conservatively, according to the TSA manual, a total of a 5% (α = 0.05; two sided) risk of a type 1 error and 80% statistical power. Other parameters were set empirically following default settings.

**References**

1. Peto R, Gray R, Collins R, Wheatley K, Hennekens C, Jamrozik K, et al. Randomised trial of prophylactic daily aspirin in British male doctors. BMJ. 1988;296(6618):313-316.

2. Steering Committee of the Physicians' Health Study Research Group. Final Report on the Aspirin Component of the Ongoing Physicians' Health Study. N Engl J Med. 1989;321(3):129-135.

3. The Medical Research Council's General Practice Research Framework. Thrombosis prevention trial: randomised trial of low-intensity oral anticoagulation with warfarin and low-dose aspirin in the primary prevention of ischaemic heart disease in men at increased risk. Lancet. 1998;351(9098):233-241.

4. Hansson L, Zanchetti A, Carruthers SG, Dahlof B, Elmfeldt D, Julius S, et al. Effects of intensive blood-pressure lowering and low-dose aspirin in patients with hypertension: principal results of the Hypertension Optimal Treatment (HOT) randomised trial. HOT Study Group. Lancet. 1998;351(9118):1755-1762.

5. De Gaetano G. Low-dose aspirin and vitamin E in people at cardiovascular risk: a randomised trial in general practice. Collaborative Group of the Primary Prevention Project. Lancet. 2001;357(9250):89-95.

6. Ridker PM, Cook NR, Lee IM, Gordon D, Gaziano JM, Manson JE, et al. A randomized trial of low-dose aspirin in the primary prevention of cardiovascular disease in women. N Engl J Med. 2005;352(13):1293-1304.

7. Belch J, MacCuish A, Campbell I, Cobbe S, Taylor R, Prescott R, et al. The prevention of progression of arterial disease and diabetes (POPADAD) trial: factorial randomised placebo controlled trial of aspirin and antioxidants in patients with diabetes and asymptomatic peripheral arterial disease. BMJ. 2008;337:a1840.

8. Ogawa H, Nakayama M, Morimoto T, Uemura S, Kanauchi M, Doi N, et al. Low-dose aspirin for primary prevention of atherosclerotic events in patients with type 2 diabetes: a randomized controlled trial. JAMA. 2008;300(18):2134-2141.

9. Fowkes FG, Price JF, Stewart MC, Butcher I, Leng GC, Pell AC, et al, Lowe GD, Murray GD. Aspirin for prevention of cardiovascular events in a general population screened for a low ankle brachial index: a randomized controlled trial. JAMA. 2010;303(9):841-848.

10. Ikeda Y, Shimada K, Teramoto T, Uchiyama S, Yamazaki T, Oikawa S, et al. Low-dose aspirin for primary prevention of cardiovascular events in Japanese patients 60 years or older with atherosclerotic risk factors: a randomized clinical trial. JAMA. 2014;312(23):2510-2520.

11. Saito Y, Okada S, Ogawa H, Soejima H, Sakuma M, Nakayama M, et al. Low-Dose Aspirin for Primary Prevention of Cardiovascular Events in Patients With Type 2 Diabetes Mellitus: 10-Year Follow-Up of a Randomized Controlled Trial. Circulation. 2017;135(7):659-670.

12. Bowman L, Mafham M, Wallendszus K, Stevens W, Buck G, Barton J, et al. Effects of Aspirin for Primary Prevention in Persons with Diabetes Mellitus. N Engl J Med. 2018;379(16):1529-1539.

13. Gaziano JM, Brotons C, Coppolecchia R, Cricelli C, Darius H, Gorelick PB, et al. Use of aspirin to reduce risk of initial vascular events in patients at moderate risk of cardiovascular disease (ARRIVE): a randomised, double-blind, placebo-controlled trial. Lancet. 2018;392(10152):1036-1046.

14. McNeil JJ, Wolfe R, Woods RL, Tonkin AM, Donnan GA, Nelson MR, et al. Effect of Aspirin on Cardiovascular Events and Bleeding in the Healthy Elderly. N Engl J Med. 2018;379(16):1509-1518.

15. Grundy SM, Stone NJ, Bailey AL, Beam C, Birtcher KK, Blumenthal RS, et al. 2018 AHA/ACC/AACVPR/AAPA/ABC/ACPM/ADA/AGS/APhA/ASPC/NLA/PCNA Guideline on the Management of Blood Cholesterol: A Report of the American College of Cardiology/American Heart Association Task Force on Clinical Practice Guidelines. Circulation. 2019;139(25):e1082-e1143.

16. Higgins JP, Altman DG, Gotzsche PC, Juni P, Moher D, Oxman AD, et al. The Cochrane Collaboration's tool for assessing risk of bias in randomised trials. BMJ. 2011;343:d5928.

17. De Lima Taga MF, Singer JM. Simple linear regression with interval censored dependent and independent variables. Stat Methods Med Res. 2018;27(1):198-207.

18. Higgins JP, Thompson SG, Deeks JJ, Altman DG. Measuring inconsistency in meta-analyses. BMJ. 2003;327(7414):557-560.

19. Egger M, Davey Smith G, Schneider M, Minder C. Bias in meta-analysis detected by a simple, graphical test. BMJ. 1997;315(7109):629-634.

20. Brok J, Thorlund K, Wetterslev J, Gluud C. Apparently conclusive meta-analyses may be inconclusive--Trial sequential analysis adjustment of random error risk due to repetitive testing of accumulating data in apparently conclusive neonatal meta-analyses. Int J Epidemiol. 2009;38(1):287-298.

21. Wetterslev J, Thorlund K, Brok J, Gluud C. Trial sequential analysis may establish when firm evidence is reached in cumulative meta-analysis. J Clin Epidemiol. 2008;61(1):64-75.
