# Supplementary material for: Pros and Cons of Aspirin for the Primary Prevention of Cardiovascular Events: A Secondary Study of Trial Sequential Analysis
Source: Front Pharmacol. 2021 Jan 14;11:592116. doi: 10.3389/fphar.2020.592116 (PMC7845480; doi:10.3389/fphar.2020.592116)
Supplement: Supplementary file 3 [file table3.docx]

**Appendix Table S2.** Cochrane Risk of Bias for the Included Studies.

|  | Randomization sequence generation (selection bias) | Allocation concealment (selection bias) | Blinding of participants and personnel (performance bias) | Blinding of outcome assessment (detection bias) | Incomplete outcome data (attrition bias) | Selective reporting (reporting bias) | Other bias |
| --- | --- | --- | --- | --- | --- | --- | --- |
| Peto, 1988^12^ | + | - | - | - | + | ? | ? |
| Steering, 1989^13^ | + | ? | + | + | + | + | - |
| Meade , 1998^14^ | + | + | + | + | + | - | + |
| Hansson, 1998^15^ | + | + | + | + | + | + | + |
| de Gaetano, 2001^16^ | + | ? | - | - | + | - | ? |
| Ridker, 2005^17^ | + | + | + | + | + | + | + |
| Belch, 2008^18^ | + | + | + | + | + | ? | + |
| Ogawa, 2008^19^ | + | + | - | - | + | + | ? |
| Fowkes, 2010^20^ | + | + | + | + | + | + | + |
| Ikeda, 2014^21^ | + | + | - | - | + | + | ? |
| Saito, 2017^22^ | + | + | - | - | + | + | ? |
| Bowman, 2018^23^ | + | + | + | + | + | + | + |
| Gaziano, 2018^24^ | + | + | + | + | + | + | + |
| McNeil, 2018^25^ | + | + | + | + | + | + | + |
