# Supplementary material for: Pros and Cons of Aspirin for the Primary Prevention of Cardiovascular Events: A Secondary Study of Trial Sequential Analysis
Source: Front Pharmacol. 2021 Jan 14;11:592116. doi: 10.3389/fphar.2020.592116 (PMC7845480; doi:10.3389/fphar.2020.592116)

Appendix File S2. Sensitivity analyses on CV events.

Figure A. Summary forest plot for sensitivity analysis on CV events by year (excluding trials published before 2000)

Study or Subgroup	Experiment Events	Experiment Total	Control Events	Control Total	Weight	Risk Ratio IV, Random, 95% CI
**< 2000**						
Peto et al (BDS) 1988	*291*	*3429*	*143*	*1710*	*6.4%*	*1.01 [0.84; 1.23]*
Steering et al (PHS) 1989	*307*	*11037*	*370*	*11034*	*10.5%*	*0.83 [0.71; 0.96]*
Total (95% CI)		14466		12744	16.9%	0.91 [0.75; 1.11]
Heterogeneity: $Tau^2 = 0.0127$; $Chi^2 = 2.65$, df = 1 (P = 0.10); $I^2 = 62\%$						
**> = 2000**						
de Gaetano et al (PPP) 2001	*45*	*2226*	*64*	*2269*	*1.6%*	*0.72 [0.49; 1.04]*
Ridker et al (WHS) 2005	*477*	*19934*	*522*	*19942*	*15.6%*	*0.91 [0.81; 1.03]*
Hansson et al (HOT) 2008	*329*	*9399*	*383*	*9391*	*11.2%*	*0.86 [0.74; 0.99]*
Ogawa et al (JPAD) 2008	*56*	*1262*	*70*	*1277*	*2.0%*	*0.81 [0.57; 1.14]*
Fowkes et al (AAA) 2010	*134*	*1675*	*136*	*1675*	*4.5%*	*0.99 [0.78; 1.24]*
Ikeda et al (JPPP) 2014	*193*	*7220*	*207*	*7244*	*6.3%*	*0.94 [0.77; 1.14]*
Satio et al (JPAD2) 2017	*151*	*992*	*166*	*1168*	*5.7%*	*1.07 [0.87; 1.31]*
Bowman et al (ASCEND) 2018	*542*	*7740*	*587*	*7740*	*18.5%*	*0.92 [0.83; 1.03]*
Gaziano et al (ARRIVE) 2018	*208*	*6270*	*218*	*6276*	*6.7%*	*0.96 [0.79; 1.15]*
McNeil et al (ASPREE) 2018	*329*	*9525*	*372*	*9589*	*11.0%*	*0.89 [0.77; 1.03]*
Total (95% CI)		66243		66571	83.1%	0.92 [0.87; 0.97]
Heterogeneity: $Tau^2 = 0$; $Chi^2 = 5.97$, df = 9 (P = 0.74); $I^2 = 0\%$						
Total (95% CI)		80709		79315	100.0%	0.91 [0.87; 0.96]
**Prediction interval**						**[0.86; 0.96]**

Heterogeneity: $Tau^2 = 0$; $Chi^2 = 8.75$, df = 11 (P = 0.64); $I^2 = 0\%$
Residual heterogeneity: Tau^2 = NA; $Chi^2 = 8.62$, df = 10 (P = 0.57); $I^2 = 0\%$
Test for overall effect: Z = -3.70 (P < 0.01)

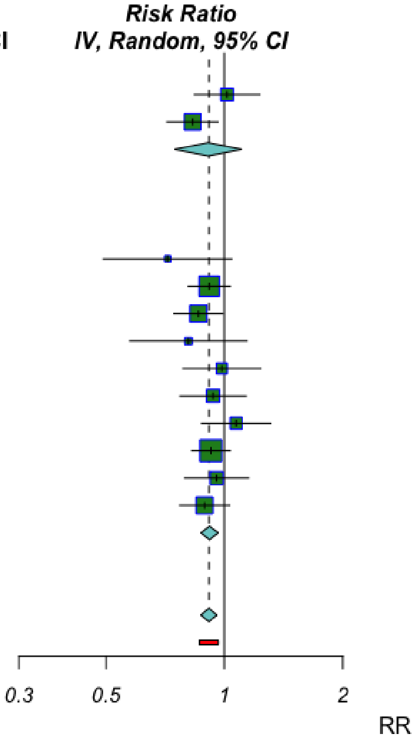

Figure B. Summary forest plot for sensitivity analysis on CV events by blinding (excluding open-label trials)

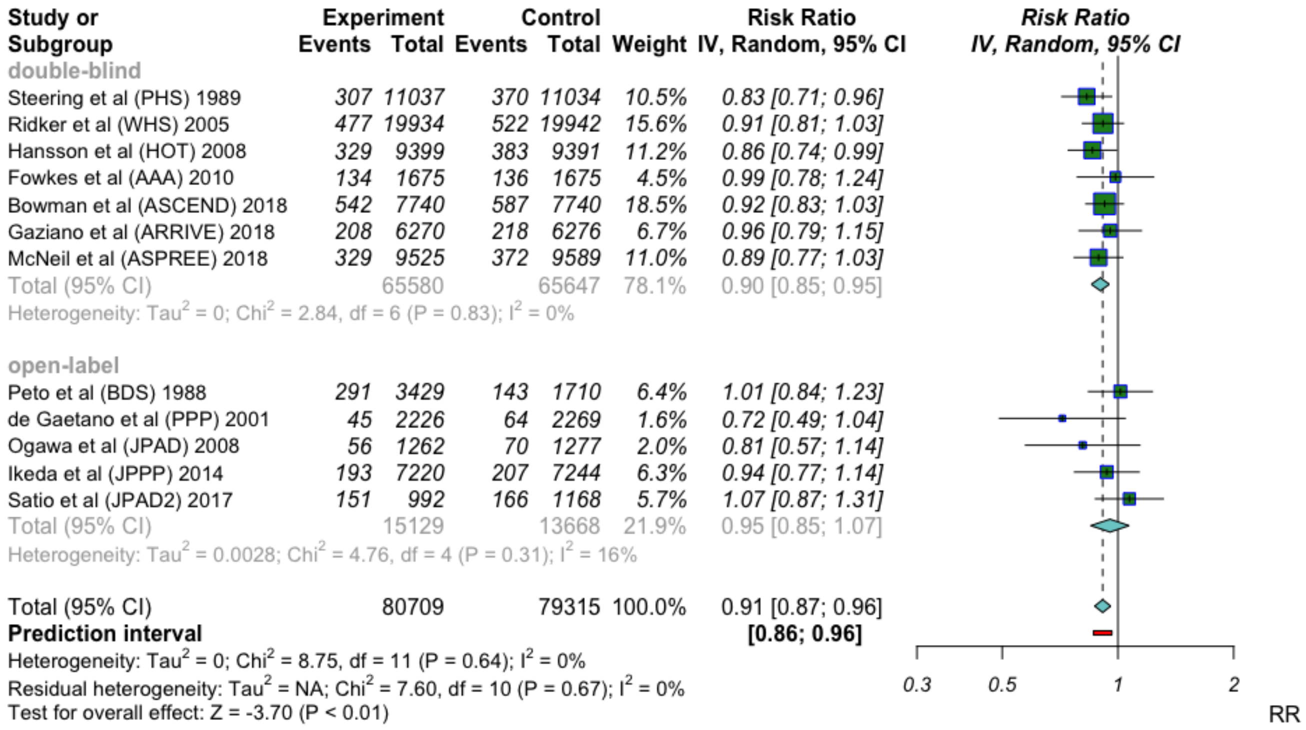

Figure C. Summary forest plot for sensitivity analysis on CV events by study quality (excluding high risk trials)

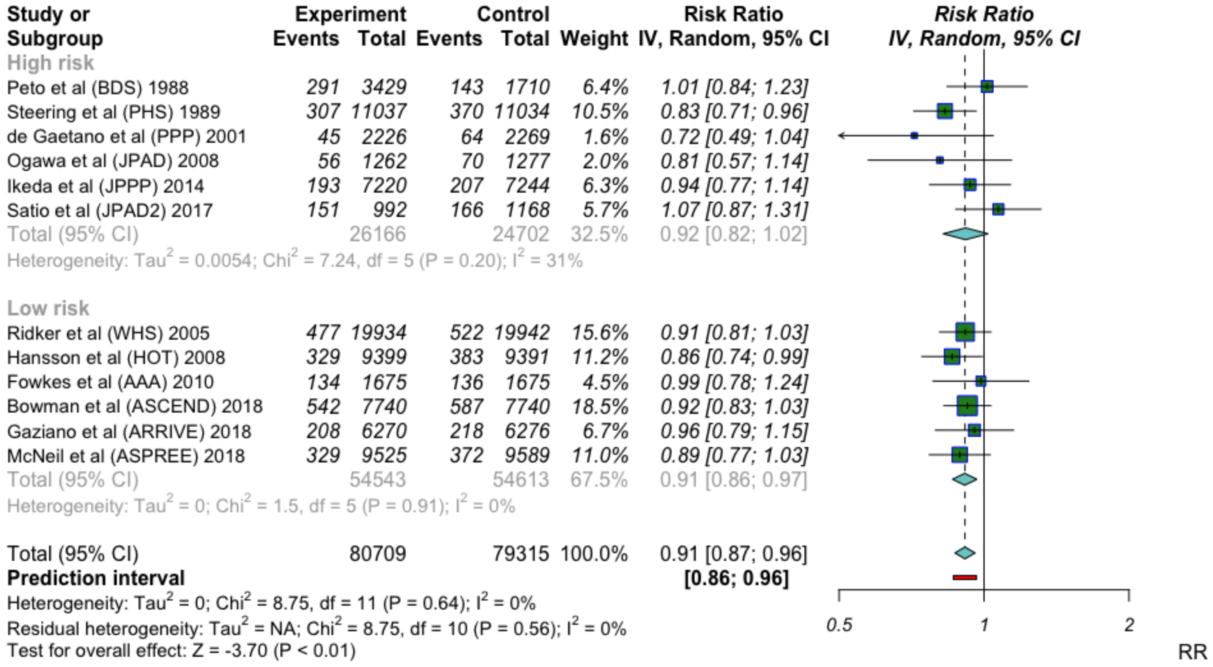

Figure D. Summary forest plot for sensitivity analysis on CV events by asymptomatic PAD (excluding asymptomatic PAD trials)

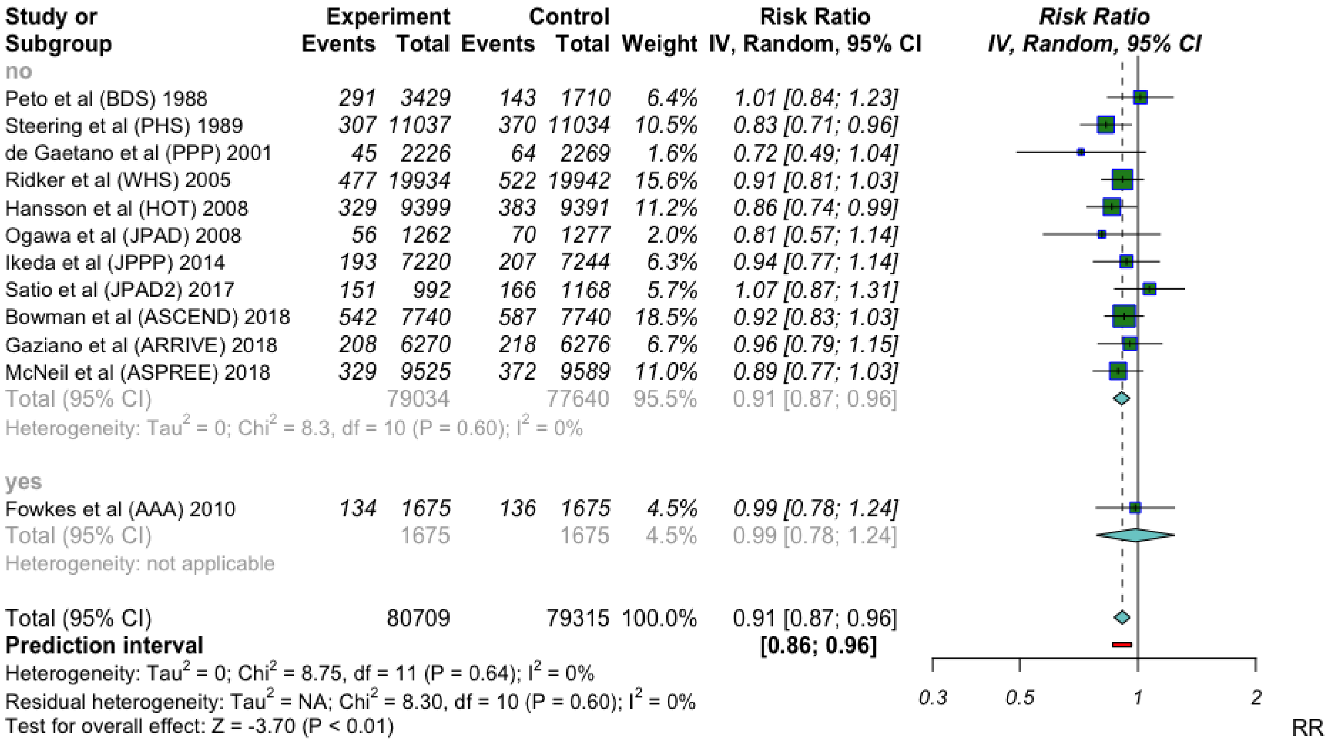

Figure E. Summary forest plot for sensitivity analysis on CV events by male populations (excluding 100% male individual trials)

Study or Subgroup	Experiment Events	Experiment Total	Control Events	Control Total	Weight	Risk Ratio IV, Random, 95% CI
**male**						
Peto et al (BDS) 1988	*291*	*3429*	*143*	*1710*	*6.4%*	*1.01 [0.84; 1.23]*
Steering et al (PHS) 1989	*307*	*11037*	*370*	*11034*	*10.5%*	*0.83 [0.71; 0.96]*
Total (95% CI)		14466		12744	16.9%	0.91 [0.75; 1.11]
Heterogeneity: $Tau^2 = 0.0127$; $Chi^2 = 2.65$, df = 1 (P = 0.10); $I^2 = 62\%$						
**not-male**						
de Gaetano et al (PPP) 2001	*45*	*2226*	*64*	*2269*	*1.6%*	*0.72 [0.49; 1.04]*
Ridker et al (WHS) 2005	*477*	*19934*	*522*	*19942*	*15.6%*	*0.91 [0.81; 1.03]*
Hansson et al (HOT) 2008	*329*	*9399*	*383*	*9391*	*11.2%*	*0.86 [0.74; 0.99]*
Ogawa et al (JPAD) 2008	*56*	*1262*	*70*	*1277*	*2.0%*	*0.81 [0.57; 1.14]*
Fowkes et al (AAA) 2010	*134*	*1675*	*136*	*1675*	*4.5%*	*0.99 [0.78; 1.24]*
Ikeda et al (JPPP) 2014	*193*	*7220*	*207*	*7244*	*6.3%*	*0.94 [0.77; 1.14]*
Satio et al (JPAD2) 2017	*151*	*992*	*166*	*1168*	*5.7%*	*1.07 [0.87; 1.31]*
Bowman et al (ASCEND) 2018	*542*	*7740*	*587*	*7740*	*18.5%*	*0.92 [0.83; 1.03]*
Gaziano et al (ARRIVE) 2018	*208*	*6270*	*218*	*6276*	*6.7%*	*0.96 [0.79; 1.15]*
McNeil et al (ASPREE) 2018	*329*	*9525*	*372*	*9589*	*11.0%*	*0.89 [0.77; 1.03]*
Total (95% CI)		66243		66571	83.1%	0.92 [0.87; 0.97]
Heterogeneity: $Tau^2 = 0$; $Chi^2 = 5.97$, df = 9 (P = 0.74); $I^2 = 0\%$						
Total (95% CI)		80709		79315	100.0%	0.91 [0.87; 0.96]
**Prediction interval**						**[0.86; 0.96]**

Heterogeneity: $Tau^2 = 0$; $Chi^2 = 8.75$, df = 11 (P = 0.64); $I^2 = 0\%$
Residual heterogeneity: $Tau^2 = NA$; $Chi^2 = 8.62$, df = 10 (P = 0.57); $I^2 = 0\%$
Test for overall effect: Z = -3.70 (P < 0.01)

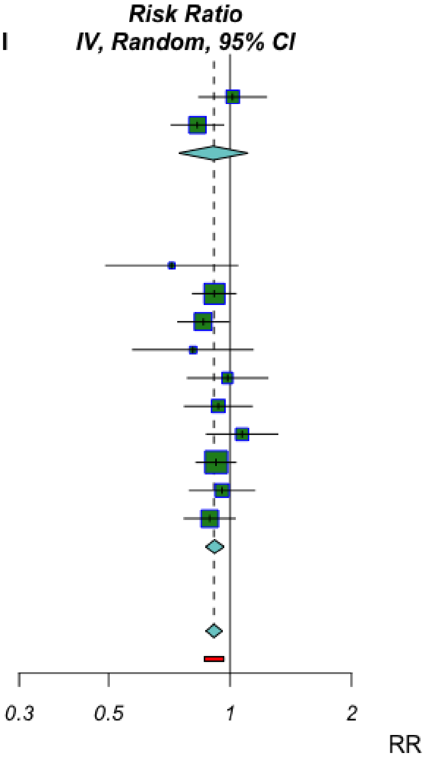

Figure F. Summary forest plot for sensitivity analysis on CV events by diabetes status (excluding 100% diabetic individual trials and restricted on 100% diabetic individual trials)

Study or Subgroup	Experiment Events	Experiment Total	Control Events	Control Total	Weight	Risk Ratio IV, Random, 95% CI
no						
Peto et al (BDS) 1988	291	3429	143	1710	6.4%	1.01 [0.84; 1.23]
Steering et al (PHS) 1989	307	11037	370	11034	10.5%	0.83 [0.71; 0.96]
de Gaetano et al (PPP) 2001	45	2226	64	2269	1.6%	0.72 [0.49; 1.04]
Ridker et al (WHS) 2005	477	19934	522	19942	15.6%	0.91 [0.81; 1.03]
Hansson et al (HOT) 2008	329	9399	383	9391	11.2%	0.86 [0.74; 0.99]
Fowkes et al (AAA) 2010	134	1675	136	1675	4.5%	0.99 [0.78; 1.24]
Ikeda et al (JPPP) 2014	193	7220	207	7244	6.3%	0.94 [0.77; 1.14]
Gaziano et al (ARRIVE) 2018	208	6270	218	6276	6.7%	0.96 [0.79; 1.15]
McNeil et al (ASPREE) 2018	329	9525	372	9589	11.0%	0.89 [0.77; 1.03]
Total (95% CI)		70715		69130	73.8%	0.90 [0.85; 0.95]
Heterogeneity: $Tau^2 = 0$; $Chi^2 = 5.7$, df = 8 (P = 0.68); $I^2 = 0\%$						
yes						
Ogawa et al (JPAD) 2008	56	1262	70	1277	2.0%	0.81 [0.57; 1.14]
Satio et al (JPAD2) 2017	151	992	166	1168	5.7%	1.07 [0.87; 1.31]
Bowman et al (ASCEND) 2018	542	7740	587	7740	18.5%	0.92 [0.83; 1.03]
Total (95% CI)		9994		10185	26.2%	0.95 [0.84; 1.06]
Heterogeneity: $Tau^2 = 0.0021$; $Chi^2 = 2.4$, df = 2 (P = 0.30); $I^2 = 17\%$						
Total (95% CI)		80709		79315	100.0%	0.91 [0.87; 0.96]
**Prediction interval**						**[0.86; 0.96]**

Heterogeneity: $Tau^2 = 0$; $Chi^2 = 8.75$, df = 11 (P = 0.64); $I^2 = 0\%$
Residual heterogeneity: Tau^2 = NA; $Chi^2 = 8.10$, df = 10 (P = 0.62); $I^2 = 0\%$
Test for overall effect: Z = -3.70 (P < 0.01)

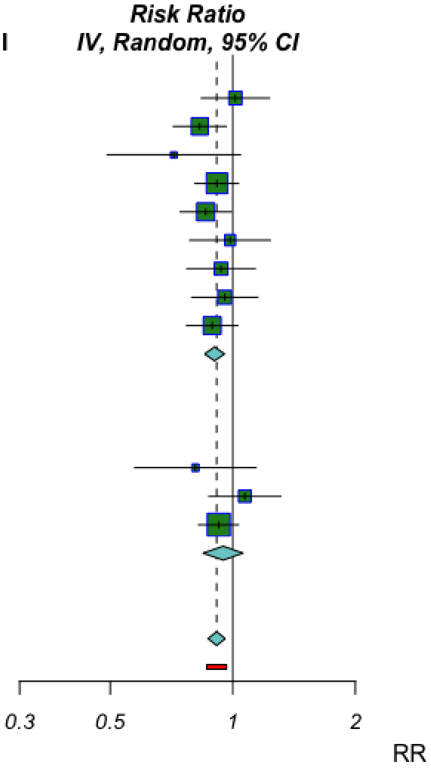

Figure G. Summary forest plot for sensitivity analysis on CV events by placebo use (excluding placebo use trials)

Study or Subgroup	Experiment Events	Experiment Total	Control Events	Control Total	Weight	Risk Ratio IV, Random, 95% CI
no						
Peto et al (BDS) 1988	291	3429	143	1710	6.4%	1.01 [0.84; 1.23]
de Gaetano et al (PPP) 2001	45	2226	64	2269	1.6%	0.72 [0.49; 1.04]
Hansson et al (HOT) 2008	329	9399	383	9391	11.2%	0.86 [0.74; 0.99]
Ogawa et al (JPAD) 2008	56	1262	70	1277	2.0%	0.81 [0.57; 1.14]
Ikeda et al (JPPP) 2014	193	7220	207	7244	6.3%	0.94 [0.77; 1.14]
Satio et al (JPAD2) 2017	151	992	166	1168	5.7%	1.07 [0.87; 1.31]
Total (95% CI)		24528		23059	33.1%	0.92 [0.84; 1.02]
Heterogeneity: $Tau^2 = 0.0030$; $Chi^2 = 6.28$, df = 5 (P = 0.28); $I^2 = 20\%$						
yes						
Steering et al (PHS) 1989	307	11037	370	11034	10.5%	0.83 [0.71; 0.96]
Ridker et al (WHS) 2005	477	19934	522	19942	15.6%	0.91 [0.81; 1.03]
Fowkes et al (AAA) 2010	134	1675	136	1675	4.5%	0.99 [0.78; 1.24]
Bowman et al (ASCEND) 2018	542	7740	587	7740	18.5%	0.92 [0.83; 1.03]
Gaziano et al (ARRIVE) 2018	208	6270	218	6276	6.7%	0.96 [0.79; 1.15]
McNeil et al (ASPREE) 2018	329	9525	372	9589	11.0%	0.89 [0.77; 1.03]
Total (95% CI)		56181		56256	66.9%	0.91 [0.86; 0.96]
Heterogeneity: $Tau^2 = 0$; $Chi^2 = 2.35$, df = 5 (P = 0.80); $I^2 = 0\%$						
Total (95% CI)		80709		79315	100.0%	0.91 [0.87; 0.96]
**Prediction interval**						**[0.86; 0.96]**

Heterogeneity: $Tau^2 = 0$; $Chi^2 = 8.75$, df = 11 (P = 0.64); $I^2 = 0\%$
Residual heterogeneity: Tau^2 = NA; $Chi^2 = 8.63$, df = 10 (P = 0.57); $I^2 = 0\%$
Test for overall effect: Z = -3.70 (P < 0.01)

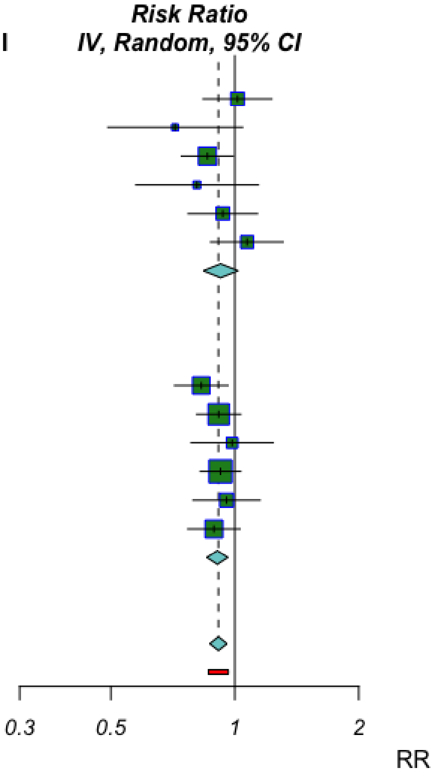

Supplement: Supplementary file 4 [file datasheet1.pdf]
