# Supplementary material for: Pros and Cons of Aspirin for the Primary Prevention of Cardiovascular Events: A Secondary Study of Trial Sequential Analysis
Source: Front Pharmacol. 2021 Jan 14;11:592116. doi: 10.3389/fphar.2020.592116 (PMC7845480; doi:10.3389/fphar.2020.592116)

Appendix File S3. Sensitivity analyses on all-cause mortality.

Figure A. Summary forest plot for sensitivity analysis on all-cause mortality by year (excluding trials published before 2000)

Study or Subgroup	experiment Events	experiment Total	control Events	control Total	Weight	Risk Ratio IV, Random, 95% CI
**< 2000**						
Peto et al (BDS) 1988	*270*	*3429*	*151*	*1710*	*5.5%*	*0.89 [0.74; 1.08]*
Steering et al (PHS) 1989	*217*	*11037*	*227*	*11034*	*5.9%*	*0.96 [0.79; 1.15]*
Hansson et al (HOT) 1998	*284*	*9399*	*305*	*9391*	*7.9%*	*0.93 [0.79; 1.09]*
Meade et al (TPT) 1998	*113*	*1268*	*110*	*1272*	*3.2%*	*1.03 [0.80; 1.32]*
Total (95% CI)		25133		23407	22.6%	0.94 [0.86; 1.03]
Heterogeneity: $Tau^2 = 0$; $Chi^2 = 0.86$, df = 3 (P = 0.84); $I^2 = 0\%$						
**>= 2000**						
de Gaetano et al (PPP) 2001	*62*	*2226*	*78*	*2269*	*1.9%*	*0.81 [0.58; 1.13]*
Ridker et al (WHS) 2005	*609*	*19934*	*642*	*19942*	*16.9%*	*0.95 [0.85; 1.06]*
Belch et al (POPADAD) 2008	*94*	*638*	*101*	*638*	*3.0%*	*0.93 [0.72; 1.21]*
Ogawa et al (JPAD) 2008	*34*	*1262*	*38*	*1277*	*1.0%*	*0.91 [0.57; 1.43]*
Fowkes et al (AAA) 2010	*176*	*1675*	*186*	*1675*	*5.3%*	*0.95 [0.78; 1.15]*
Ikeda et al (JPPP) 2014	*297*	*7220*	*303*	*7244*	*8.2%*	*0.98 [0.84; 1.15]*
Bowman et al (ASCEND) 2018	*748*	*7740*	*792*	*7740*	*22.4%*	*0.94 [0.86; 1.04]*
Gaziano et al (ARRIVE) 2018	*160*	*6270*	*161*	*6276*	*4.3%*	*0.99 [0.80; 1.23]*
McNeil et al (ASPREE) 2018	*558*	*9525*	*494*	*9589*	*14.5%*	*1.14 [1.01; 1.28]*
Total (95% CI)		56490		56650	77.4%	0.98 [0.93; 1.04]
Heterogeneity: $Tau^2 = 0.0006$; $Chi^2 = 8.75$, df = 8 (P = 0.36); $I^2 = 9\%$						
Total (95% CI)		81623		80057	100.0%	0.97 [0.93; 1.02]
**Prediction interval**						**[0.92; 1.02]**

Heterogeneity: $Tau^2 = 0$; $Chi^2 = 10.21$, df = 12 (P = 0.60); $I^2 = 0\%$
Residual heterogeneity: $Tau^2 = NA$; $Chi^2 = 9.61$, df = 11 (P = 0.57); $I^2 = 0\%$
Test for overall effect: Z = -1.24 (P = 0.22)

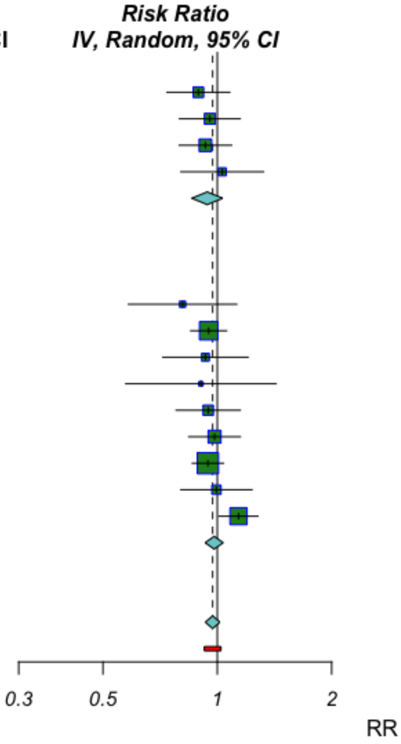

Figure B. Summary forest plot for sensitivity analysis on all-cause mortality by blinding (excluding open-label trials)

Study or Subgroup	Experiment Events	Experiment Total	Control Events	Control Total	Weight	Risk Ratio IV, Random, 95% CI
**double-blind**						
Steering et al (PHS) 1989	*217*	*11037*	*227*	*11034*	*5.9%*	*0.96 [0.79; 1.15]*
Hansson et al (HOT) 1998	*284*	*9399*	*305*	*9391*	*7.9%*	*0.93 [0.79; 1.09]*
Meade et al (TPT) 1998	*113*	*1268*	*110*	*1272*	*3.2%*	*1.03 [0.80; 1.32]*
Ridker et al (WHS) 2005	*609*	*19934*	*642*	*19942*	*16.9%*	*0.95 [0.85; 1.06]*
Belch et al (POPADAD) 2008	*94*	*638*	*101*	*638*	*3.0%*	*0.93 [0.72; 1.21]*
Fowkes et al (AAA) 2010	*176*	*1675*	*186*	*1675*	*5.3%*	*0.95 [0.78; 1.15]*
Bowman et al (ASCEND) 2018	*748*	*7740*	*792*	*7740*	*22.4%*	*0.94 [0.86; 1.04]*
Gaziano et al (ARRIVE) 2018	*160*	*6270*	*161*	*6276*	*4.3%*	*0.99 [0.80; 1.23]*
McNeil et al (ASPREE) 2018	*558*	*9525*	*494*	*9589*	*14.5%*	*1.14 [1.01; 1.28]*
Total (95% CI)		67486		67557	83.4%	0.98 [0.93; 1.03]
Heterogeneity: $Tau^2 = 0$; $Chi^2 = 7.98$, df = 8 (P = 0.44); $I^2 = 0\%$						
**open-label**						
Peto et al (BDS) 1988	*270*	*3429*	*151*	*1710*	*5.5%*	*0.89 [0.74; 1.08]*
de Gaetano et al (PPP) 2001	*62*	*2226*	*78*	*2269*	*1.9%*	*0.81 [0.58; 1.13]*
Ogawa et al (JPAD) 2008	*34*	*1262*	*38*	*1277*	*1.0%*	*0.91 [0.57; 1.43]*
Ikeda et al (JPPP) 2014	*297*	*7220*	*303*	*7244*	*8.2%*	*0.98 [0.84; 1.15]*
Total (95% CI)		14137		12500	16.6%	0.93 [0.83; 1.03]
Heterogeneity: $Tau^2 = 0$; $Chi^2 = 1.36$, df = 3 (P = 0.71); $I^2 = 0\%$						
Total (95% CI)		81623		80057	100.0%	0.97 [0.93; 1.02]
**Prediction interval**						**[0.92; 1.02]**

Heterogeneity: $Tau^2 = 0$; $Chi^2 = 10.21$, df = 12 (P = 0.60); $I^2 = 0\%$
Residual heterogeneity: $Tau^2 = NA$; $Chi^2 = 9.34$, df = 11 (P = 0.59); $I^2 = 0\%$
Test for overall effect: Z = -1.24 (P = 0.22)

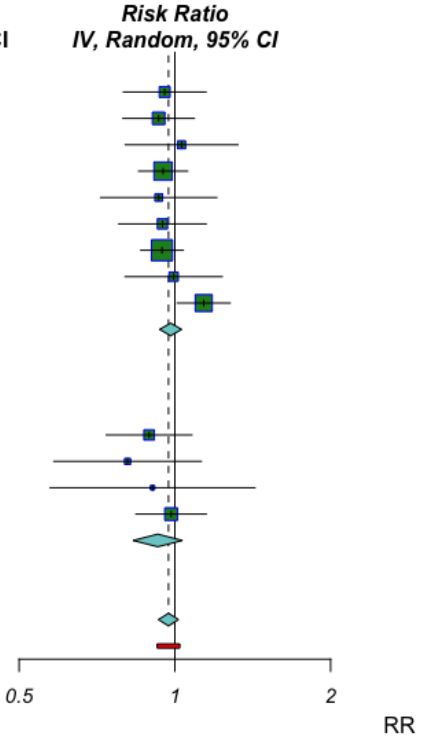

Figure C. Summary forest plot for sensitivity analysis on all-cause mortality by study quality (excluding high risk trials)

Study or Subgroup	Experiment Events	Experiment Total	Control Events	Control Total	Weight	Risk Ratio IV, Random, 95% CI
**High risk**						
Peto et al (BDS) 1988	*270*	*3429*	*151*	*1710*	*5.5%*	*0.89 [0.74; 1.08]*
Steering et al (PHS) 1989	*217*	*11037*	*227*	*11034*	*5.9%*	*0.96 [0.79; 1.15]*
Meade et al (TPT) 1998	*113*	*1268*	*110*	*1272*	*3.2%*	*1.03 [0.80; 1.32]*
de Gaetano et al (PPP) 2001	*62*	*2226*	*78*	*2269*	*1.9%*	*0.81 [0.58; 1.13]*
Ogawa et al (JPAD) 2008	*34*	*1262*	*38*	*1277*	*1.0%*	*0.91 [0.57; 1.43]*
Ikeda et al (JPPP) 2014	*297*	*7220*	*303*	*7244*	*8.2%*	*0.98 [0.84; 1.15]*
Total (95% CI)		26442		24806	25.7%	0.95 [0.87; 1.03]
Heterogeneity: $Tau^2 = 0$; $Chi^2 = 1.96$, df = 5 (P = 0.86); $I^2 = 0\%$						
**Low risk**						
Hansson et al (HOT) 1998	*284*	*9399*	*305*	*9391*	*7.9%*	*0.93 [0.79; 1.09]*
Ridker et al (WHS) 2005	*609*	*19934*	*642*	*19942*	*16.9%*	*0.95 [0.85; 1.06]*
Belch et al (POPADAD) 2008	*94*	*638*	*101*	*638*	*3.0%*	*0.93 [0.72; 1.21]*
Fowkes et al (AAA) 2010	*176*	*1675*	*186*	*1675*	*5.3%*	*0.95 [0.78; 1.15]*
Bowman et al (ASCEND) 2018	*748*	*7740*	*792*	*7740*	*22.4%*	*0.94 [0.86; 1.04]*
Gaziano et al (ARRIVE) 2018	*160*	*6270*	*161*	*6276*	*4.3%*	*0.99 [0.80; 1.23]*
McNeil et al (ASPREE) 2018	*558*	*9525*	*494*	*9589*	*14.5%*	*1.14 [1.01; 1.28]*
Total (95% CI)		55181		55251	74.3%	0.98 [0.92; 1.04]
Heterogeneity: $Tau^2 = 0.0015$; $Chi^2 = 7.75$, df = 6 (P = 0.26); $I^2 = 23\%$						
Total (95% CI)		81623		80057	100.0%	0.97 [0.93; 1.02]
**Prediction interval**						**[0.92; 1.02]**
Heterogeneity: $Tau^2 = 0$; $Chi^2 = 10.21$, df = 12 (P = 0.60); $I^2 = 0\%$						
Residual heterogeneity: Tau^2 = NA; $Chi^2 = 9.71$, df = 11 (P = 0.56); $I^2 = 0\%$						
Test for overall effect: Z = -1.24 (P = 0.22)						

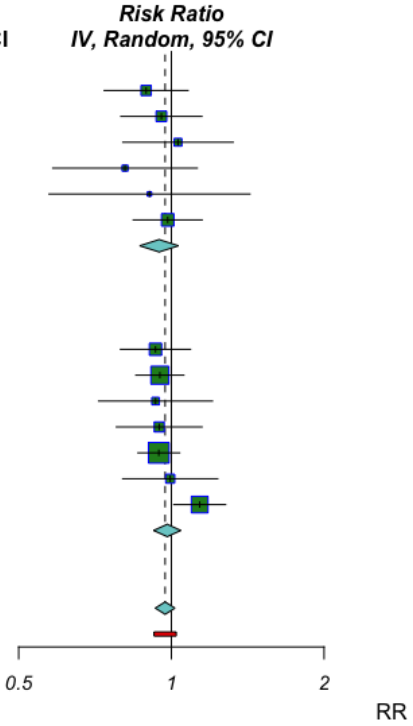

Figure D. Summary forest plot for sensitivity analysis on all-cause mortality by asymptomatic PAD (excluding asymptomatic PAD trials)

Study or Subgroup	experiment Events	experiment Total	control Events	control Total	Weight	Risk Ratio IV, Random, 95% CI
no						
Peto et al (BDS) 1988	*270*	*3429*	*151*	*1710*	*5.5%*	*0.89 [0.74; 1.08]*
Steering et al (PHS) 1989	*217*	*11037*	*227*	*11034*	*5.9%*	*0.96 [0.79; 1.15]*
Hansson et al (HOT) 1998	*284*	*9399*	*305*	*9391*	*7.9%*	*0.93 [0.79; 1.09]*
Meade et al (TPT) 1998	*113*	*1268*	*110*	*1272*	*3.2%*	*1.03 [0.80; 1.32]*
de Gaetano et al (PPP) 2001	*62*	*2226*	*78*	*2269*	*1.9%*	*0.81 [0.58; 1.13]*
Ridker et al (WHS) 2005	*609*	*19934*	*642*	*19942*	*16.9%*	*0.95 [0.85; 1.06]*
Ogawa et al (JPAD) 2008	*34*	*1262*	*38*	*1277*	*1.0%*	*0.91 [0.57; 1.43]*
Ikeda et al (JPPP) 2014	*297*	*7220*	*303*	*7244*	*8.2%*	*0.98 [0.84; 1.15]*
Bowman et al (ASCEND) 2018	*748*	*7740*	*792*	*7740*	*22.4%*	*0.94 [0.86; 1.04]*
Gaziano et al (ARRIVE) 2018	*160*	*6270*	*161*	*6276*	*4.3%*	*0.99 [0.80; 1.23]*
McNeil et al (ASPREE) 2018	*558*	*9525*	*494*	*9589*	*14.5%*	*1.14 [1.01; 1.28]*
Total (95% CI)		79310		77744	91.7%	0.97 [0.93; 1.02]
Heterogeneity: Tau^2 = < 0.0001; Chi^2 = 10.01, df = 10 (P = 0.44); I^2 = 0%						
yes						
Belch et al (POPADAD) 2008	*94*	*638*	*101*	*638*	*3.0%*	*0.93 [0.72; 1.21]*
Fowkes et al (AAA) 2010	*176*	*1675*	*186*	*1675*	*5.3%*	*0.95 [0.78; 1.15]*
Total (95% CI)		2313		2313	8.3%	0.94 [0.81; 1.10]
Heterogeneity: Tau^2 = 0; Chi^2 = 0.01, df = 1 (P = 0.92); I^2 = 0%						
Total (95% CI)		81623		80057	100.0%	0.97 [0.93; 1.02]
**Prediction interval**						**[0.92; 1.02]**

Heterogeneity: Tau^2 = 0; Chi^2 = 10.21, df = 12 (P = 0.60); I^2 = 0%
Residual heterogeneity: Tau^2 = NA; Chi^2 = 10.02, df = 11 (P = 0.53); I^2 = 0%
Test for overall effect: Z = -1.24 (P = 0.22)

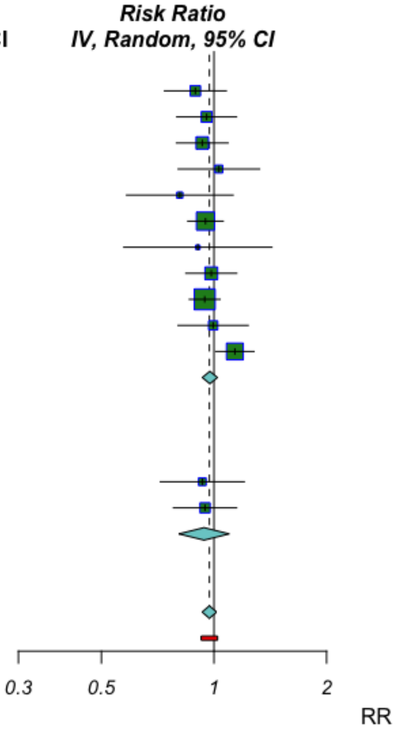

Figure E. Summary forest plot for sensitivity analysis on all-cause mortality by male populations (excluding 100% male individual trials)

Study or Subgroup	Experiment Events	Experiment Total	Control Events	Control Total	Weight	Risk Ratio IV, Random, 95% CI
**male**						
Peto et al (BDS) 1988	270	3429	151	1710	5.5%	0.89 [0.74; 1.08]
Steering et al (PHS) 1989	217	11037	227	11034	5.9%	0.96 [0.79; 1.15]
Meade et al (TPT) 1998	113	1268	110	1272	3.2%	1.03 [0.80; 1.32]
Total (95% CI)		15734		14016	14.7%	0.95 [0.84; 1.06]
Heterogeneity: $Tau^2 = 0$; $Chi^2 = 0.83$, df = 2 (P = 0.66); $I^2 = 0\%$						
**not-male**						
Hansson et al (HOT) 1998	284	9399	305	9391	7.9%	0.93 [0.79; 1.09]
de Gaetano et al (PPP) 2001	62	2226	78	2269	1.9%	0.81 [0.58; 1.13]
Ridker et al (WHS) 2005	609	19934	642	19942	16.9%	0.95 [0.85; 1.06]
Belch et al (POPADAD) 2008	94	638	101	638	3.0%	0.93 [0.72; 1.21]
Ogawa et al (JPAD) 2008	34	1262	38	1277	1.0%	0.91 [0.57; 1.43]
Fowkes et al (AAA) 2010	176	1675	186	1675	5.3%	0.95 [0.78; 1.15]
Ikeda et al (JPPP) 2014	297	7220	303	7244	8.2%	0.98 [0.84; 1.15]
Bowman et al (ASCEND) 2018	748	7740	792	7740	22.4%	0.94 [0.86; 1.04]
Gaziano et al (ARRIVE) 2018	160	6270	161	6276	4.3%	0.99 [0.80; 1.23]
McNeil et al (ASPREE) 2018	558	9525	494	9589	14.5%	1.14 [1.01; 1.28]
Total (95% CI)		65889		66041	85.3%	0.98 [0.93; 1.03]
Heterogeneity: $Tau^2 = 0.0001$; $Chi^2 = 9.14$, df = 9 (P = 0.42); $I^2 = 2\%$						
Total (95% CI)		81623		80057	100.0%	0.97 [0.93; 1.02]
**Prediction interval**						**[0.92; 1.02]**

Heterogeneity: $Tau^2 = 0$; $Chi^2 = 10.21$, df = 12 (P = 0.60); $I^2 = 0\%$
Residual heterogeneity: Tau^2 = NA; $Chi^2 = 9.97$, df = 11 (P = 0.53); $I^2 = 0\%$
Test for overall effect: Z = -1.24 (P = 0.22)

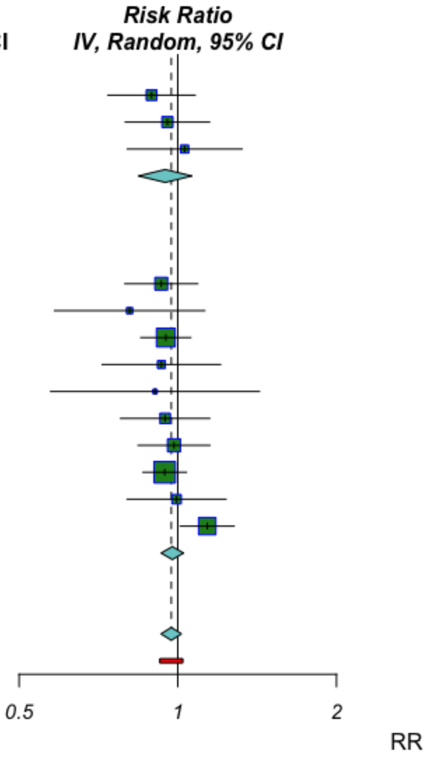

Figure F. Summary forest plot for sensitivity analysis on all-cause mortality by diabetes status (excluding 100% diabetic individual trials and restricted on 100% diabetic individual trials)

Study or Subgroup	Experiment Events	Experiment Total	Control Events	Control Total	Weight	Risk Ratio IV, Random, 95% CI
no						
Peto et al (BDS) 1988	270	3429	151	1710	5.5%	0.89 [0.74; 1.08]
Steering et al (PHS) 1989	217	11037	227	11034	5.9%	0.96 [0.79; 1.15]
Hansson et al (HOT) 1998	284	9399	305	9391	7.9%	0.93 [0.79; 1.09]
Meade et al (TPT) 1998	113	1268	110	1272	3.2%	1.03 [0.80; 1.32]
de Gaetano et al (PPP) 2001	62	2226	78	2269	1.9%	0.81 [0.58; 1.13]
Ridker et al (WHS) 2005	609	19934	642	19942	16.9%	0.95 [0.85; 1.06]
Fowkes et al (AAA) 2010	176	1675	186	1675	5.3%	0.95 [0.78; 1.15]
Ikeda et al (JPPP) 2014	297	7220	303	7244	8.2%	0.98 [0.84; 1.15]
Gaziano et al (ARRIVE) 2018	160	6270	161	6276	4.3%	0.99 [0.80; 1.23]
McNeil et al (ASPREE) 2018	558	9525	494	9589	14.5%	1.14 [1.01; 1.28]
Total (95% CI)		71983		70402	73.7%	0.98 [0.93; 1.04]
Heterogeneity: $Tau^2 = 0.0004$; $Chi^2 = 9.46$, df = 9 (P = 0.40); $I^2 = 5\%$						
yes						
Belch et al (POPADAD) 2008	94	638	101	638	3.0%	0.93 [0.72; 1.21]
Ogawa et al (JPAD) 2008	34	1262	38	1277	1.0%	0.91 [0.57; 1.43]
Bowman et al (ASCEND) 2018	748	7740	792	7740	22.4%	0.94 [0.86; 1.04]
Total (95% CI)		9640		9655	26.3%	0.94 [0.86; 1.03]
Heterogeneity: $Tau^2 = 0$; $Chi^2 = 0.04$, df = 2 (P = 0.98); $I^2 = 0\%$						
Total (95% CI)		81623		80057	100.0%	0.97 [0.93; 1.02]
**Prediction interval**						**[0.92; 1.02]**

Heterogeneity: $Tau^2 = 0$; $Chi^2 = 10.21$, df = 12 (P = 0.60); $I^2 = 0\%$
Residual heterogeneity: $Tau^2 = NA$; $Chi^2 = 9.50$, df = 11 (P = 0.58); $I^2 = 0\%$
Test for overall effect: Z = -1.24 (P = 0.22)

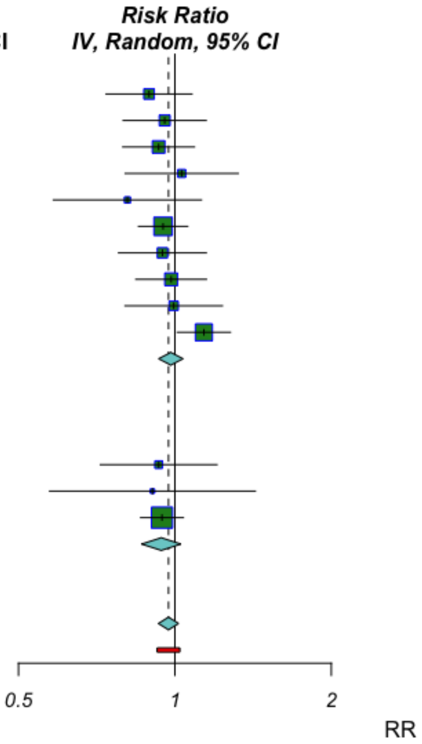

Figure G. Summary forest plot for sensitivity analysis on all-cause mortality by placebo use (excluding placebo use trials)

Study or Subgroup	Experiment Events	Experiment Total	Control Events	Control Total	Weight	Risk Ratio IV, Random, 95% CI
**no**						
Peto et al (BDS) 1988	270	3429	151	1710	5.5%	0.89 [0.74; 1.08]
de Gaetano et al (PPP) 2001	62	2226	78	2269	1.9%	0.81 [0.58; 1.13]
Ogawa et al (JPAD) 2008	34	1262	38	1277	1.0%	0.91 [0.57; 1.43]
Ikeda et al (JPPP) 2014	297	7220	303	7244	8.2%	0.98 [0.84; 1.15]
Total (95% CI)		14137		12500	16.6%	0.93 [0.83; 1.03]
Heterogeneity: $Tau^2 = 0$; $Chi^2 = 1.36$, df = 3 (P = 0.71); $I^2 = 0\%$						
**yes**						
Steering et al (PHS) 1989	217	11037	227	11034	5.9%	0.96 [0.79; 1.15]
Hansson et al (HOT) 1998	284	9399	305	9391	7.9%	0.93 [0.79; 1.09]
Meade et al (TPT) 1998	113	1268	110	1272	3.2%	1.03 [0.80; 1.32]
Ridker et al (WHS) 2005	609	19934	642	19942	16.9%	0.95 [0.85; 1.06]
Belch et al (POPADAD) 2008	94	638	101	638	3.0%	0.93 [0.72; 1.21]
Fowkes et al (AAA) 2010	176	1675	186	1675	5.3%	0.95 [0.78; 1.15]
Bowman et al (ASCEND) 2018	748	7740	792	7740	22.4%	0.94 [0.86; 1.04]
Gaziano et al (ARRIVE) 2018	160	6270	161	6276	4.3%	0.99 [0.80; 1.23]
McNeil et al (ASPREE) 2018	558	9525	494	9589	14.5%	1.14 [1.01; 1.28]
Total (95% CI)		67486		67557	83.4%	0.98 [0.93; 1.03]
Heterogeneity: $Tau^2 = 0$; $Chi^2 = 7.98$, df = 8 (P = 0.44); $I^2 = 0\%$						
Total (95% CI)		81623		80057	100.0%	0.97 [0.93; 1.02]
**Prediction interval**						**[0.92; 1.02]**

Heterogeneity: $Tau^2 = 0$; $Chi^2 = 10.21$, df = 12 (P = 0.60); $I^2 = 0\%$
Residual heterogeneity: Tau^2 = NA; $Chi^2 = 9.34$, df = 11 (P = 0.59); $I^2 = 0\%$
Test for overall effect: Z = -1.24 (P = 0.22)

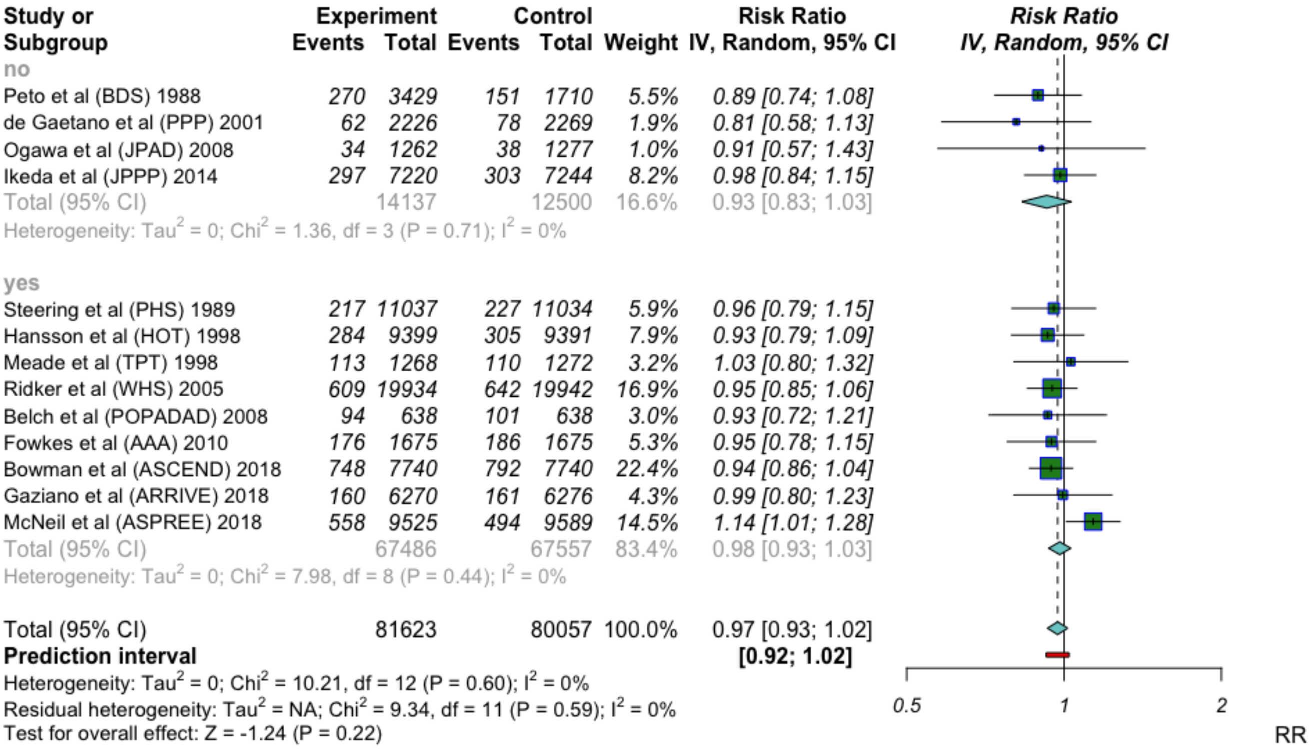

Figure H. Summary forest plot for sensitivity analysis on all-cause mortality by excluding TPT trial

Study or Subgroup	Experiment Events	Experiment Total	Control Events	Control Total	Weight	Risk Ratio IV, Random, 95% CI
no						
Peto et al (BDS) 1988	*270*	*3429*	*151*	*1710*	*5.5%*	*0.89 [0.74; 1.08]*
Steering et al (PHS) 1989	*217*	*11037*	*227*	*11034*	*5.9%*	*0.96 [0.79; 1.15]*
Hansson et al (HOT) 1998	*284*	*9399*	*305*	*9391*	*7.9%*	*0.93 [0.79; 1.09]*
de Gaetano et al (PPP) 2001	*62*	*2226*	*78*	*2269*	*1.9%*	*0.81 [0.58; 1.13]*
Ridker et al (WHS) 2005	*609*	*19934*	*642*	*19942*	*16.9%*	*0.95 [0.85; 1.06]*
Belch et al (POPADAD) 2008	*94*	*638*	*101*	*638*	*3.0%*	*0.93 [0.72; 1.21]*
Ogawa et al (JPAD) 2008	*34*	*1262*	*38*	*1277*	*1.0%*	*0.91 [0.57; 1.43]*
Fowkes et al (AAA) 2010	*176*	*1675*	*186*	*1675*	*5.3%*	*0.95 [0.78; 1.15]*
Ikeda et al (JPPP) 2014	*297*	*7220*	*303*	*7244*	*8.2%*	*0.98 [0.84; 1.15]*
Bowman et al (ASCEND) 2018	*748*	*7740*	*792*	*7740*	*22.4%*	*0.94 [0.86; 1.04]*
Gaziano et al (ARRIVE) 2018	*160*	*6270*	*161*	*6276*	*4.3%*	*0.99 [0.80; 1.23]*
McNeil et al (ASPREE) 2018	*558*	*9525*	*494*	*9589*	*14.5%*	*1.14 [1.01; 1.28]*
Total (95% CI)		80355		78785	96.8%	0.97 [0.93; 1.02]
Heterogeneity: $Tau^2 = 0$; $Chi^2 = 9.99$, df = 11 (P = 0.53); $I^2 = 0\%$						
yes						
Meade et al (TPT) 1998	*113*	*1268*	*110*	*1272*	*3.2%*	*1.03 [0.80; 1.32]*
Total (95% CI)		1268		1272	3.2%	1.03 [0.80; 1.32]
Heterogeneity: not applicable						
Total (95% CI)		81623		80057	100.0%	0.97 [0.93; 1.02]
**Prediction interval**						**[0.92; 1.02]**

Heterogeneity: $Tau^2 = 0$; $Chi^2 = 10.21$, df = 12 (P = 0.60); $I^2 = 0\%$
Residual heterogeneity: $Tau^2 = NA$; $Chi^2 = 9.99$, df = 11 (P = 0.53); $I^2 = 0\%$
Test for overall effect: Z = -1.24 (P = 0.22)

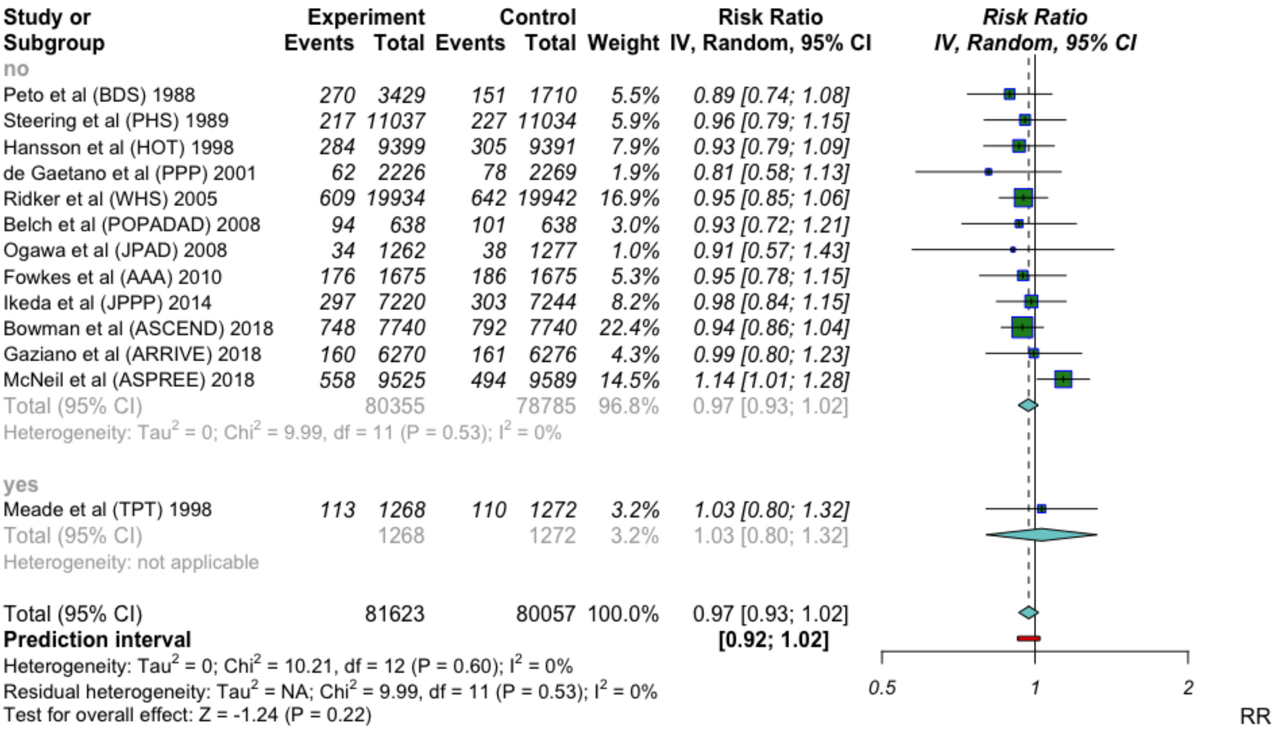

Supplement: Supplementary file 5 [file datasheet2.pdf]
