# Supplementary material for: Pros and Cons of Aspirin for the Primary Prevention of Cardiovascular Events: A Secondary Study of Trial Sequential Analysis
Source: Front Pharmacol. 2021 Jan 14;11:592116. doi: 10.3389/fphar.2020.592116 (PMC7845480; doi:10.3389/fphar.2020.592116)

Appendix File S4. Sensitivity analyses on cardiovascular mortality.

Figure A. Summary forest plot for sensitivity analysis on cardiovascular mortality by year (excluding trials published before 2000)

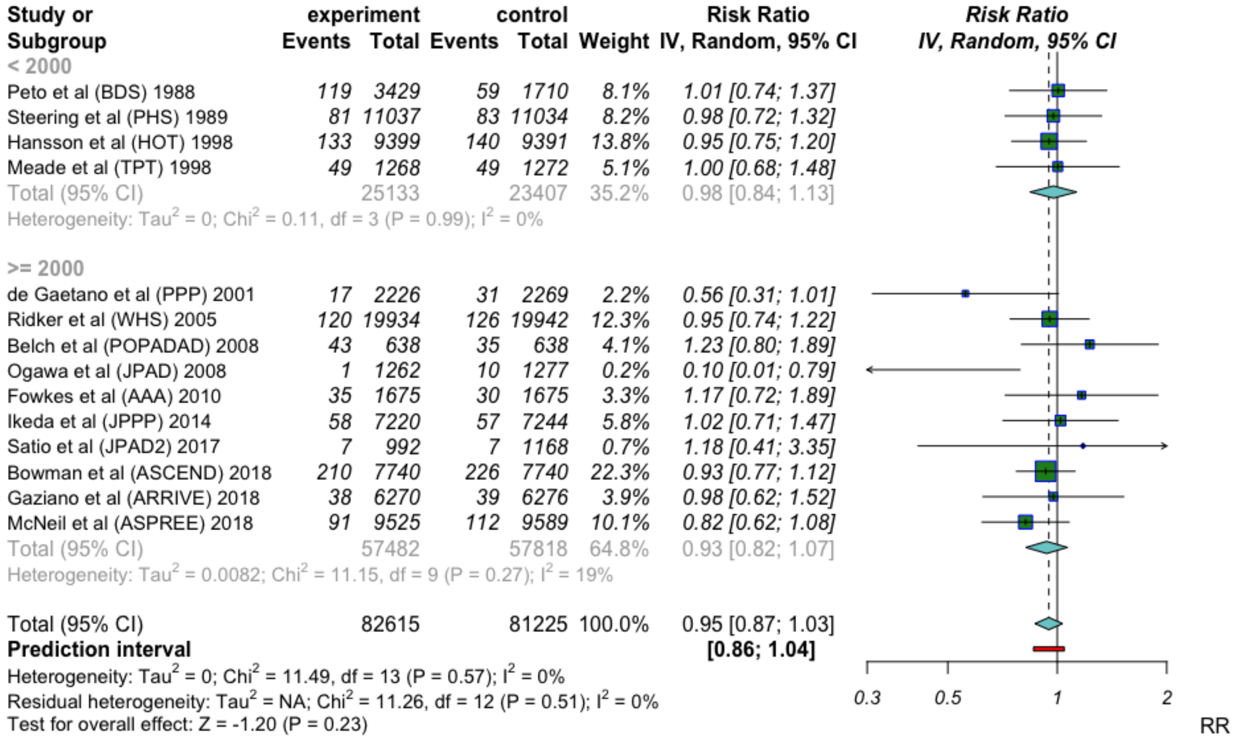

Figure B. Summary forest plot for sensitivity analysis on cardiovascular mortality by blinding (excluding open-label trials)

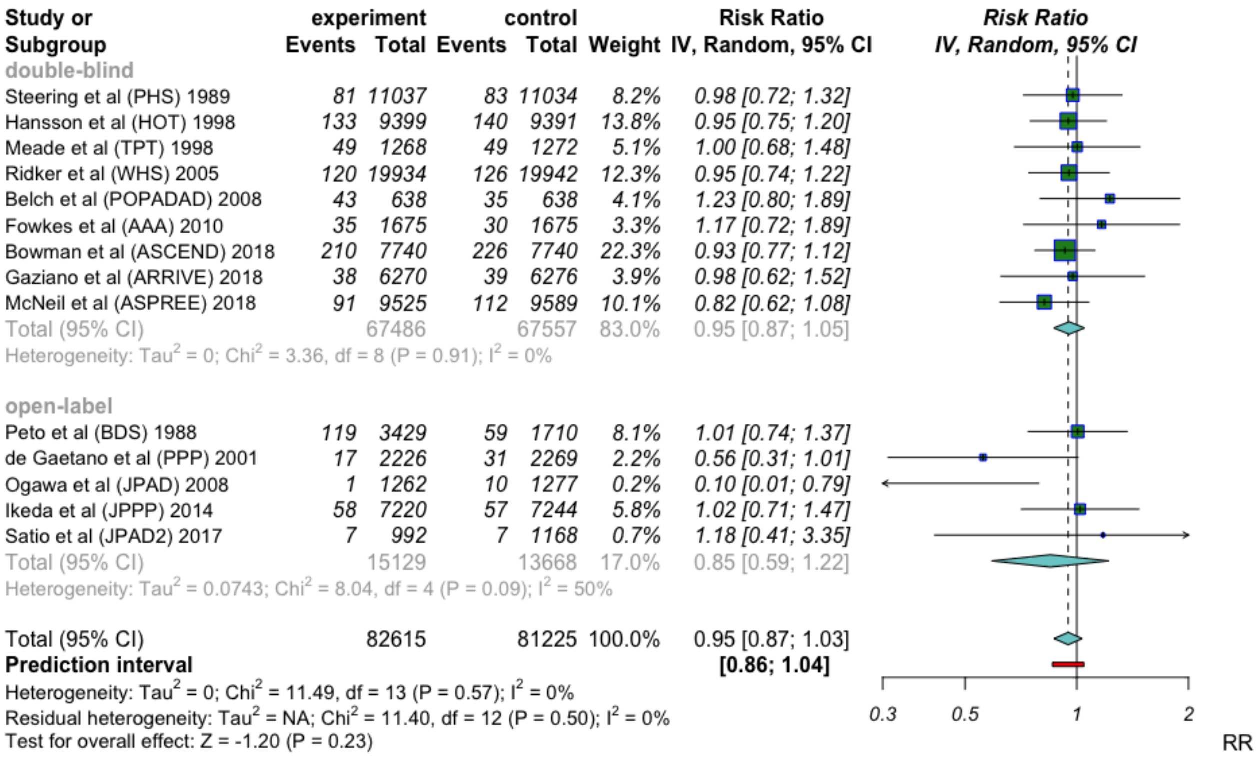

Figure C. Summary forest plot for sensitivity analysis on cardiovascular mortality by study quality (excluding high risk trials)

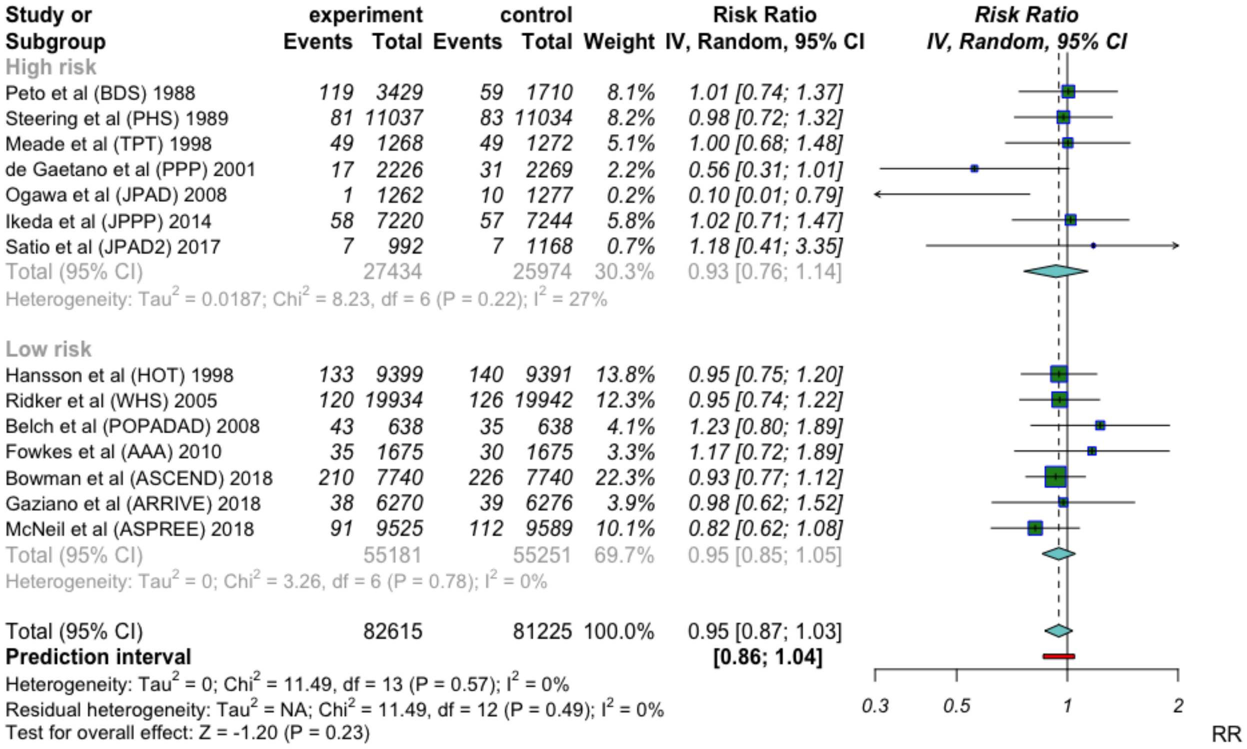

Figure D. Summary forest plot for sensitivity analysis on cardiovascular mortality by asymptomatic PAD (excluding asymptomatic PAD trials)

Study or Subgroup	experiment Events	experiment Total	control Events	control Total	Weight	Risk Ratio IV, Random, 95% CI
no						
Peto et al (BDS) 1988	*119*	*3429*	*59*	*1710*	*8.1%*	*1.01 [0.74; 1.37]*
Steering et al (PHS) 1989	*81*	*11037*	*83*	*11034*	*8.2%*	*0.98 [0.72; 1.32]*
Hansson et al (HOT) 1998	*133*	*9399*	*140*	*9391*	*13.8%*	*0.95 [0.75; 1.20]*
Meade et al (TPT) 1998	*49*	*1268*	*49*	*1272*	*5.1%*	*1.00 [0.68; 1.48]*
de Gaetano et al (PPP) 2001	*17*	*2226*	*31*	*2269*	*2.2%*	*0.56 [0.31; 1.01]*
Ridker et al (WHS) 2005	*120*	*19934*	*126*	*19942*	*12.3%*	*0.95 [0.74; 1.22]*
Ogawa et al (JPAD) 2008	*1*	*1262*	*10*	*1277*	*0.2%*	*0.10 [0.01; 0.79]*
Ikeda et al (JPPP) 2014	*58*	*7220*	*57*	*7244*	*5.8%*	*1.02 [0.71; 1.47]*
Satio et al (JPAD2) 2017	*7*	*992*	*7*	*1168*	*0.7%*	*1.18 [0.41; 3.35]*
Bowman et al (ASCEND) 2018	*210*	*7740*	*226*	*7740*	*22.3%*	*0.93 [0.77; 1.12]*
Gaziano et al (ARRIVE) 2018	*38*	*6270*	*39*	*6276*	*3.9%*	*0.98 [0.62; 1.52]*
McNeil et al (ASPREE) 2018	*91*	*9525*	*112*	*9589*	*10.1%*	*0.82 [0.62; 1.08]*
Total (95% CI)		80302		78912	92.6%	0.93 [0.85; 1.02]
Heterogeneity: $Tau^2 = 0$; $Chi^2 = 9.24$, df = 11 (P = 0.60); $I^2 = 0\%$						
yes						
Belch et al (POPADAD) 2008	*43*	*638*	*35*	*638*	*4.1%*	*1.23 [0.80; 1.89]*
Fowkes et al (AAA) 2010	*35*	*1675*	*30*	*1675*	*3.3%*	*1.17 [0.72; 1.89]*
Total (95% CI)		2313		2313	7.4%	1.20 [0.87; 1.66]
Heterogeneity: $Tau^2 = 0$; $Chi^2 = 0.02$, df = 1 (P = 0.88); $I^2 = 0\%$						
Total (95% CI)		82615		81225	100.0%	0.95 [0.87; 1.03]
**Prediction interval**						**[0.86; 1.04]**

Heterogeneity: $Tau^2 = 0$; $Chi^2 = 11.49$, df = 13 (P = 0.57); $I^2 = 0\%$
Residual heterogeneity: Tau^2 = NA; $Chi^2 = 9.26$, df = 12 (P = 0.68); $I^2 = 0\%$
Test for overall effect: Z = -1.20 (P = 0.23)

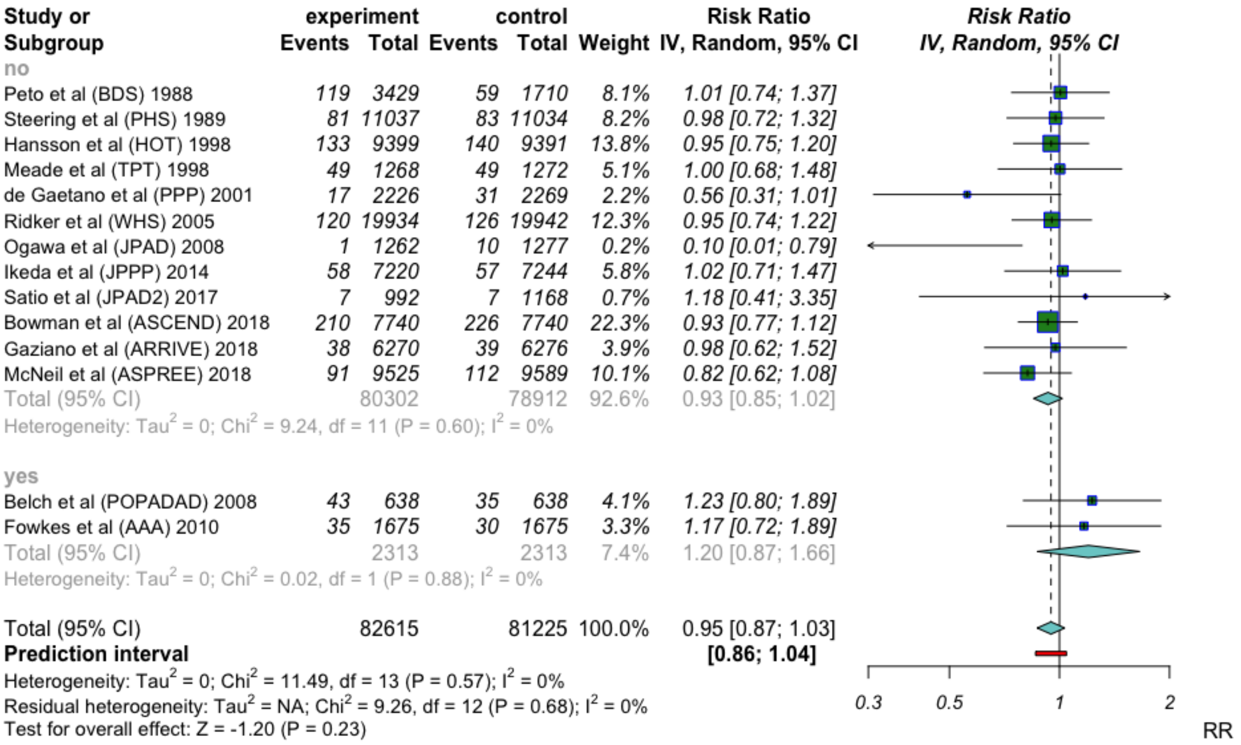

Figure E. Summary forest plot for sensitivity analysis on cardiovascular mortality by male populations (excluding 100% male individual trials)

Study or Subgroup	experiment Events	experiment Total	control Events	control Total	Weight	Risk Ratio IV, Random, 95% CI
male						
Peto et al (BDS) 1988	*119*	*3429*	*59*	*1710*	*8.1%*	*1.01 [0.74; 1.37]*
Steering et al (PHS) 1989	*81*	*11037*	*83*	*11034*	*8.2%*	*0.98 [0.72; 1.32]*
Meade et al (TPT) 1998	*49*	*1268*	*49*	*1272*	*5.1%*	*1.00 [0.68; 1.48]*
Total (95% CI)		15734		14016	21.4%	0.99 [0.82; 1.20]
Heterogeneity: $Tau^2 = 0$; $Chi^2 = 0.02$, df = 2 (P = 0.99); $I^2 = 0\%$						
non all male						
Hansson et al (HOT) 1998	*133*	*9399*	*140*	*9391*	*13.8%*	*0.95 [0.75; 1.20]*
de Gaetano et al (PPP) 2001	*17*	*2226*	*31*	*2269*	*2.2%*	*0.56 [0.31; 1.01]*
Ridker et al (WHS) 2005	*120*	*19934*	*126*	*19942*	*12.3%*	*0.95 [0.74; 1.22]*
Belch et al (POPADAD) 2008	*43*	*638*	*35*	*638*	*4.1%*	*1.23 [0.80; 1.89]*
Ogawa et al (JPAD) 2008	*1*	*1262*	*10*	*1277*	*0.2%*	*0.10 [0.01; 0.79]*
Fowkes et al (AAA) 2010	*35*	*1675*	*30*	*1675*	*3.3%*	*1.17 [0.72; 1.89]*
Ikeda et al (JPPP) 2014	*58*	*7220*	*57*	*7244*	*5.8%*	*1.02 [0.71; 1.47]*
Satio et al (JPAD2) 2017	*7*	*992*	*7*	*1168*	*0.7%*	*1.18 [0.41; 3.35]*
Bowman et al (ASCEND) 2018	*210*	*7740*	*226*	*7740*	*22.3%*	*0.93 [0.77; 1.12]*
Gaziano et al (ARRIVE) 2018	*38*	*6270*	*39*	*6276*	*3.9%*	*0.98 [0.62; 1.52]*
McNeil et al (ASPREE) 2018	*91*	*9525*	*112*	*9589*	*10.1%*	*0.82 [0.62; 1.08]*
Total (95% CI)		66881		67209	78.6%	0.94 [0.84; 1.04]
Heterogeneity: $Tau^2 = 0.0035$; $Chi^2 = 11.17$, df = 10 (P = 0.34); $I^2 = 10\%$						
Total (95% CI)		82615		81225	100.0%	0.95 [0.87; 1.03]
**Prediction interval**						**[0.86; 1.04]**

Heterogeneity: $Tau^2 = 0$; $Chi^2 = 11.49$, df = 13 (P = 0.57); $I^2 = 0\%$
Residual heterogeneity: $Tau^2 = NA$; $Chi^2 = 11.19$, df = 12 (P = 0.51); $I^2 = 0\%$
Test for overall effect: Z = -1.20 (P = 0.23)

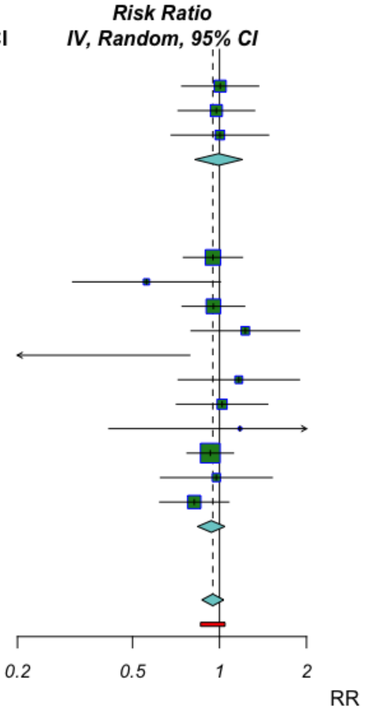

Figure F. Summary forest plot for sensitivity analysis on cardiovascular mortality by diabetes status (excluding 100% diabetic individual trials and restricted on 100% diabetic individual trials)

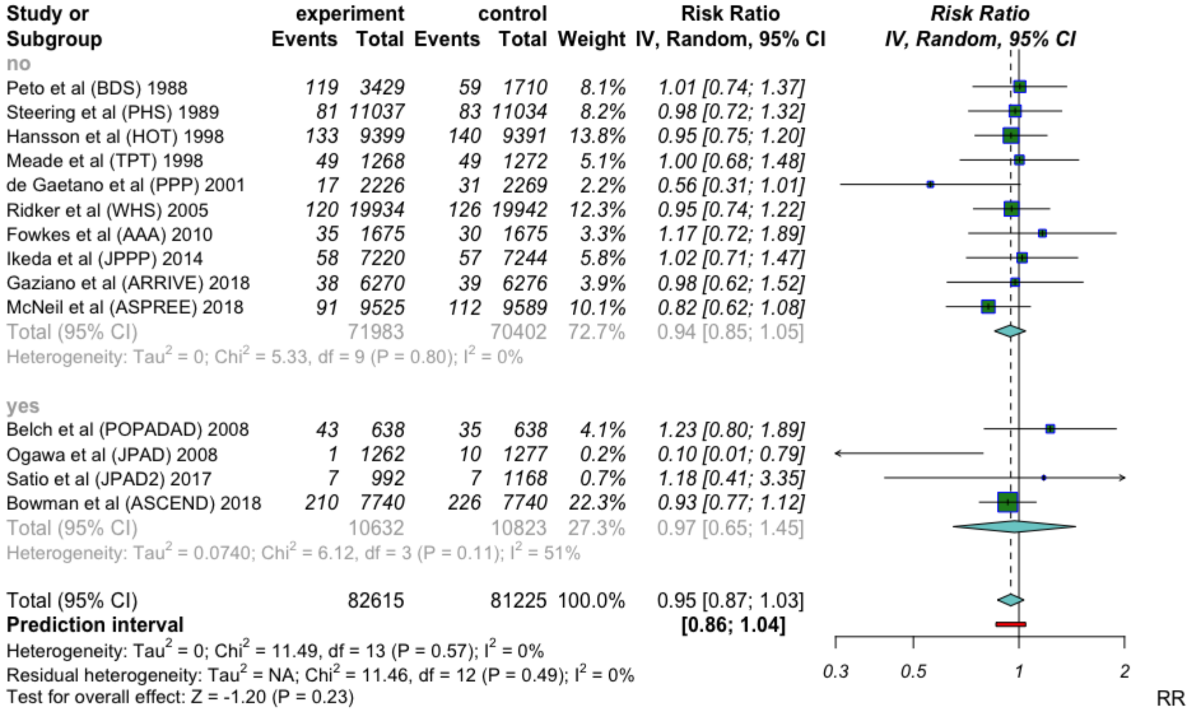

Figure G. Summary forest plot for sensitivity analysis on cardiovascular mortality by placebo use (excluding placebo use trials)

Study or Subgroup	experiment Events	experiment Total	control Events	control Total	Weight	Risk Ratio IV, Random, 95% CI
no						
Peto et al (BDS) 1988	119	3429	59	1710	8.1%	1.01 [0.74; 1.37]
de Gaetano et al (PPP) 2001	17	2226	31	2269	2.2%	0.56 [0.31; 1.01]
Ogawa et al (JPAD) 2008	1	1262	10	1277	0.2%	0.10 [0.01; 0.79]
Ikeda et al (JPPP) 2014	58	7220	57	7244	5.8%	1.02 [0.71; 1.47]
Satio et al (JPAD2) 2017	7	992	7	1168	0.7%	1.18 [0.41; 3.35]
Total (95% CI)		15129		13668	17.0%	0.85 [0.59; 1.22]
Heterogeneity: $Tau^2 = 0.0743$; $Chi^2 = 8.04$, df = 4 (P = 0.09); $I^2 = 50\%$						
yes						
Steering et al (PHS) 1989	81	11037	83	11034	8.2%	0.98 [0.72; 1.32]
Hansson et al (HOT) 1998	133	9399	140	9391	13.8%	0.95 [0.75; 1.20]
Meade et al (TPT) 1998	49	1268	49	1272	5.1%	1.00 [0.68; 1.48]
Ridker et al (WHS) 2005	120	19934	126	19942	12.3%	0.95 [0.74; 1.22]
Belch et al (POPADAD) 2008	43	638	35	638	4.1%	1.23 [0.80; 1.89]
Fowkes et al (AAA) 2010	35	1675	30	1675	3.3%	1.17 [0.72; 1.89]
Bowman et al (ASCEND) 2018	210	7740	226	7740	22.3%	0.93 [0.77; 1.12]
Gaziano et al (ARRIVE) 2018	38	6270	39	6276	3.9%	0.98 [0.62; 1.52]
McNeil et al (ASPREE) 2018	91	9525	112	9589	10.1%	0.82 [0.62; 1.08]
Total (95% CI)		67486		67557	83.0%	0.95 [0.87; 1.05]
Heterogeneity: $Tau^2 = 0$; $Chi^2 = 3.36$, df = 8 (P = 0.91); $I^2 = 0\%$						
Total (95% CI)		82615		81225	100.0%	0.95 [0.87; 1.03]
**Prediction interval**						**[0.86; 1.04]**

Heterogeneity: $Tau^2 = 0$; $Chi^2 = 11.49$, df = 13 (P = 0.57); $I^2 = 0\%$
Residual heterogeneity: Tau^2 = NA; $Chi^2 = 11.40$, df = 12 (P = 0.50); $I^2 = 0\%$
Test for overall effect: Z = -1.20 (P = 0.23)

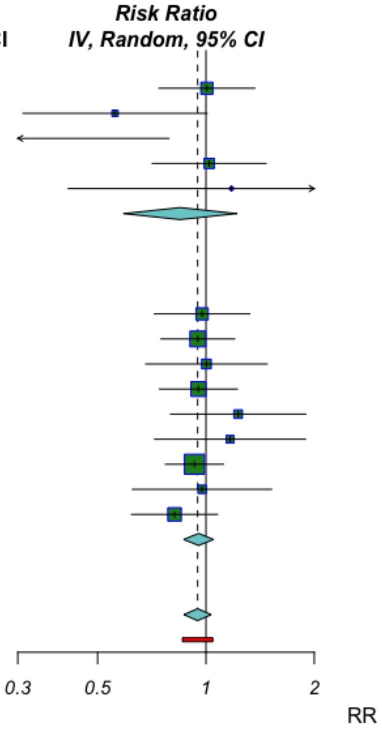

Figure H. Summary forest plot for sensitivity analysis on cardiovascular mortality by excluding TPT trial

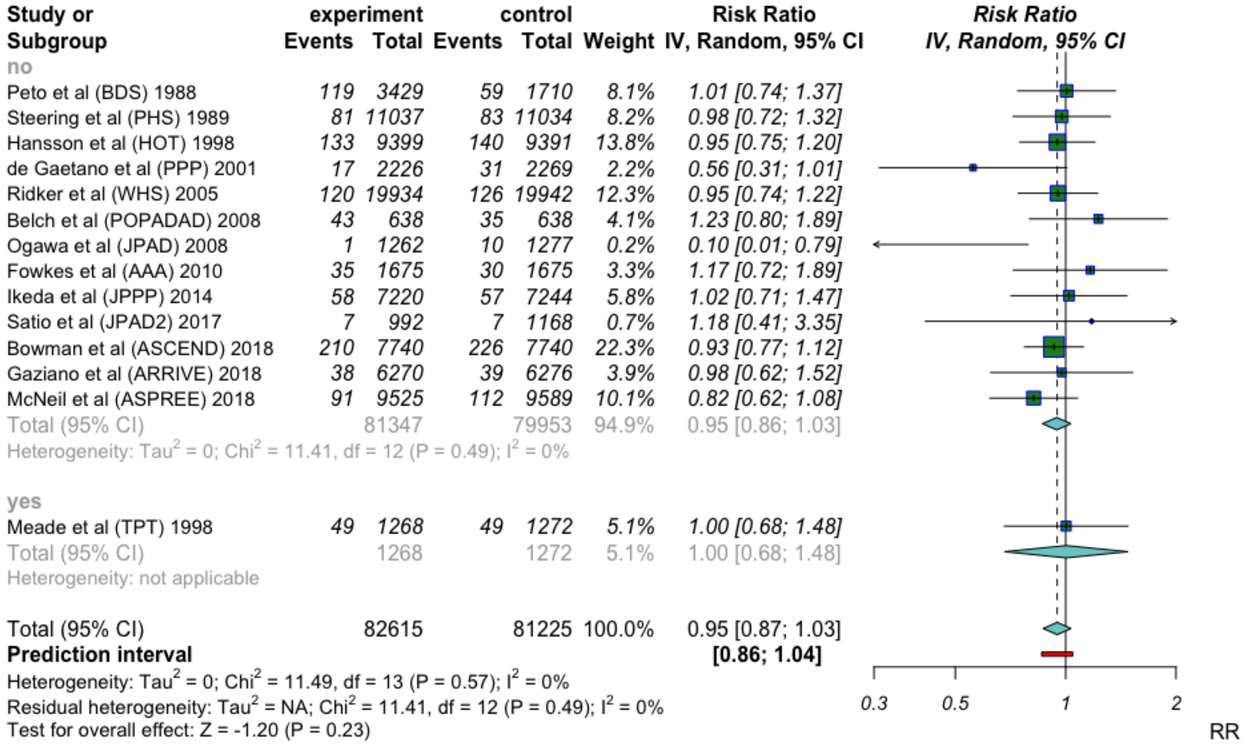

Supplement: Supplementary file 6 [file datasheet3.pdf]
