# Supplementary material for: Pros and Cons of Aspirin for the Primary Prevention of Cardiovascular Events: A Secondary Study of Trial Sequential Analysis
Source: Front Pharmacol. 2021 Jan 14;11:592116. doi: 10.3389/fphar.2020.592116 (PMC7845480; doi:10.3389/fphar.2020.592116)

Appendix File S5. Sensitivity analyses on all-MI.

Figure A. Summary forest plot for sensitivity analysis on all-MI by year (excluding trials published before 2000)

Study or Subgroup	experiment Events	experiment Total	control Events	control Total	Weight	Risk Ratio IV, Random, 95% CI
< 2000						
Peto et al (BDS) 1988	*181*	*3429*	*86*	*1710*	*8.3%*	*1.05 [0.82; 1.35]*
Steering et al (PHS) 1989	*139*	*11037*	*239*	*11034*	*9.4%*	*0.58 [0.47; 0.72]*
Hansson et al (HOT) 1998	*82*	*9399*	*127*	*9391*	*7.6%*	*0.65 [0.49; 0.85]*
Meade et al (TPT) 1998	*83*	*1268*	*107*	*1272*	*7.6%*	*0.78 [0.59; 1.03]*
Total (95% CI)		25133		23407	32.9%	0.74 [0.57; 0.97]
Heterogeneity: $Tau^2 = 0.0579$; $Chi^2 = 13.7$, df = 3 (P < 0.01); $I^2 = 78\%$						
>= 2000						
de Gaetano et al (PPP) 2001	*19*	*2226*	*28*	*2269*	*3.0%*	*0.69 [0.39; 1.23]*
Ridker et al (WHS) 2005	*198*	*19934*	*193*	*19942*	*9.7%*	*1.03 [0.84; 1.25]*
Belch et al (POPADAD) 2008	*76*	*638*	*69*	*638*	*6.9%*	*1.10 [0.81; 1.50]*
Ogawa et al (JPAD) 2008	*12*	*1262*	*14*	*1277*	*1.9%*	*0.87 [0.40; 1.87]*
Fowkes et al (AAA) 2010	*90*	*1675*	*86*	*1675*	*7.3%*	*1.05 [0.78; 1.40]*
Ikeda et al (JPPP) 2014	*27*	*7220*	*37*	*7244*	*3.9%*	*0.73 [0.45; 1.20]*
Satio et al (JPAD2) 2017	*57*	*992*	*68*	*1168*	*6.2%*	*0.99 [0.70; 1.39]*
Bowman et al (ASCEND) 2018	*296*	*7740*	*317*	*7740*	*10.9%*	*0.93 [0.80; 1.09]*
Gaziano et al (ARRIVE) 2018	*95*	*6270*	*112*	*6276*	*7.7%*	*0.85 [0.65; 1.11]*
McNeil et al (ASPREE) 2018	*171*	*9525*	*184*	*9589*	*9.5%*	*0.94 [0.76; 1.15]*
Total (95% CI)		57482		57818	67.1%	0.95 [0.88; 1.03]
Heterogeneity: $Tau^2 = 0$; $Chi^2 = 4.96$, df = 9 (P = 0.84); $I^2 = 0\%$						
Total (95% CI)		82615		81225	100.0%	0.87 [0.77; 0.97]
**Prediction interval**						**[0.60; 1.26]**

Heterogeneity: $Tau^2 = 0.0253$; $Chi^2 = 31.22$, df = 13 (P < 0.01); $I^2 = 58\%$
Residual heterogeneity: Tau^2 = NA; $Chi^2 = 18.65$, df = 12 (P = 0.10); $I^2 = 36\%$
Test for overall effect: Z = -2.39 (P = 0.02)

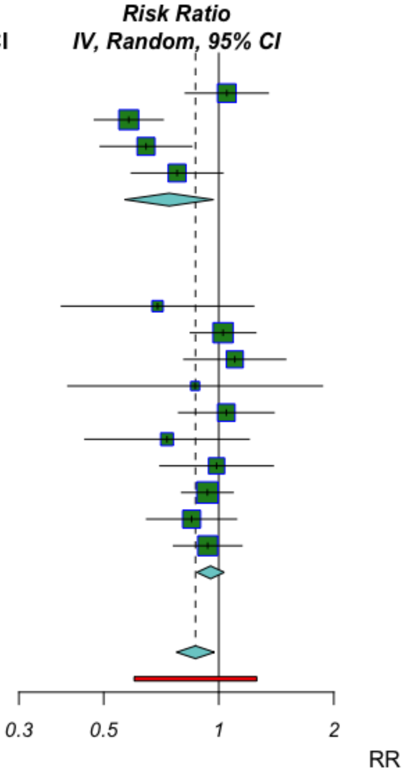

Figure B. Summary forest plot for sensitivity analysis on all-MI by blinding (excluding open-label trials)

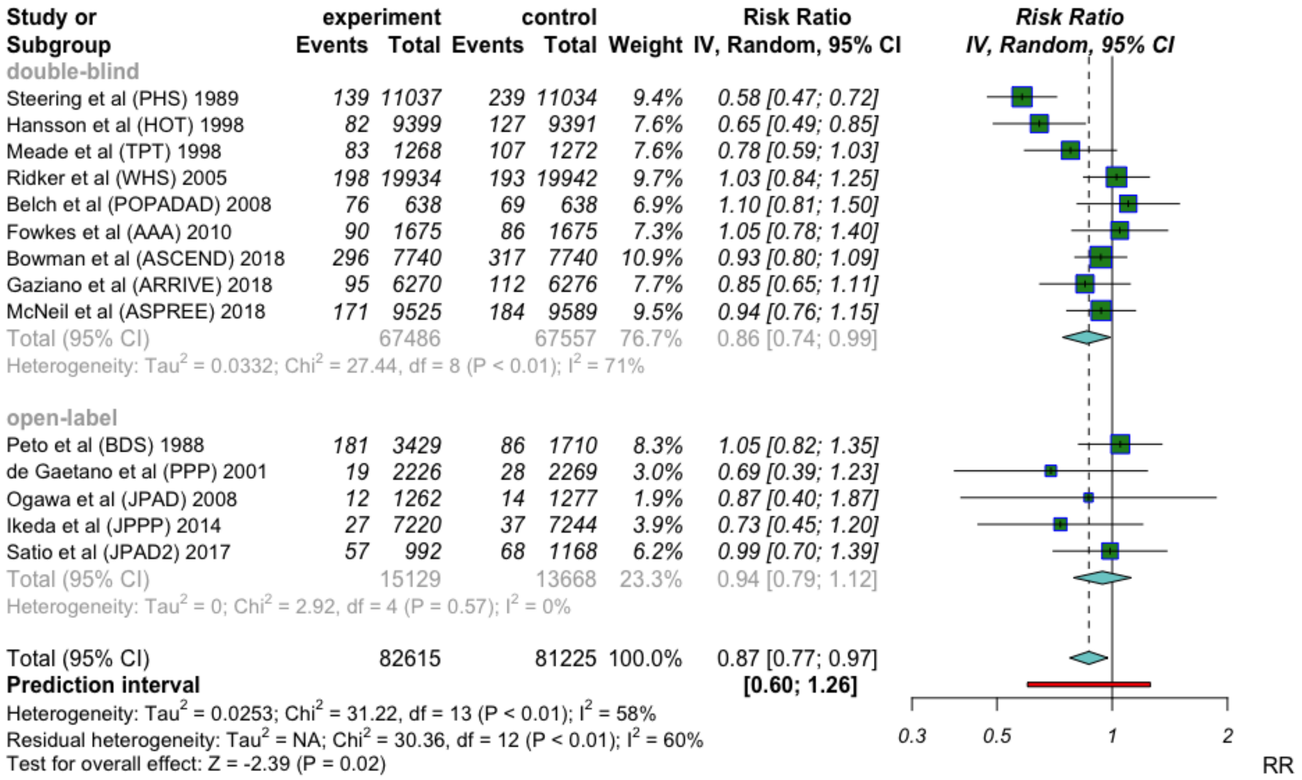

Figure C. Summary forest plot for sensitivity analysis on all-MI by study quality (excluding high risk trials)

Study or Subgroup	experiment Events	experiment Total	control Events	control Total	Weight	Risk Ratio IV, Random, 95% CI
**High-risk**						
Peto et al (BDS) 1988	181	3429	86	1710	8.3%	1.05 [0.82; 1.35]
Steering et al (PHS) 1989	139	11037	239	11034	9.4%	0.58 [0.47; 0.72]
Meade et al (TPT) 1998	83	1268	107	1272	7.6%	0.78 [0.59; 1.03]
de Gaetano et al (PPP) 2001	19	2226	28	2269	3.0%	0.69 [0.39; 1.23]
Ogawa et al (JPAD) 2008	12	1262	14	1277	1.9%	0.87 [0.40; 1.87]
Ikeda et al (JPPP) 2014	27	7220	37	7244	3.9%	0.73 [0.45; 1.20]
Satio et al (JPAD2) 2017	57	992	68	1168	6.2%	0.99 [0.70; 1.39]
Total (95% CI)		27434		25974	40.3%	0.80 [0.64; 0.98]
Heterogeneity: $Tau^2 = 0.0442$; $Chi^2 = 15.23$, df = 6 (P = 0.02); $I^2 = 61\%$						
**Low-risk**						
Hansson et al (HOT) 1998	82	9399	127	9391	7.6%	0.65 [0.49; 0.85]
Ridker et al (WHS) 2005	198	19934	193	19942	9.7%	1.03 [0.84; 1.25]
Belch et al (POPADAD) 2008	76	638	69	638	6.9%	1.10 [0.81; 1.50]
Fowkes et al (AAA) 2010	90	1675	86	1675	7.3%	1.05 [0.78; 1.40]
Bowman et al (ASCEND) 2018	296	7740	317	7740	10.9%	0.93 [0.80; 1.09]
Gaziano et al (ARRIVE) 2018	95	6270	112	6276	7.7%	0.85 [0.65; 1.11]
McNeil et al (ASPREE) 2018	171	9525	184	9589	9.5%	0.94 [0.76; 1.15]
Total (95% CI)		55181		55251	59.7%	0.93 [0.83; 1.04]
Heterogeneity: $Tau^2 = 0.0090$; $Chi^2 = 9.95$, df = 6 (P = 0.13); $I^2 = 40\%$						
Total (95% CI)		82615		81225	100.0%	0.87 [0.77; 0.97]
**Prediction interval**						**[0.60; 1.26]**

Heterogeneity: $Tau^2 = 0.0253$; $Chi^2 = 31.22$, df = 13 (P < 0.01); $I^2 = 58\%$
Residual heterogeneity: Tau^2 = NA; $Chi^2 = 25.18$, df = 12 (P = 0.01); $I^2 = 52\%$
Test for overall effect: Z = -2.39 (P = 0.02)

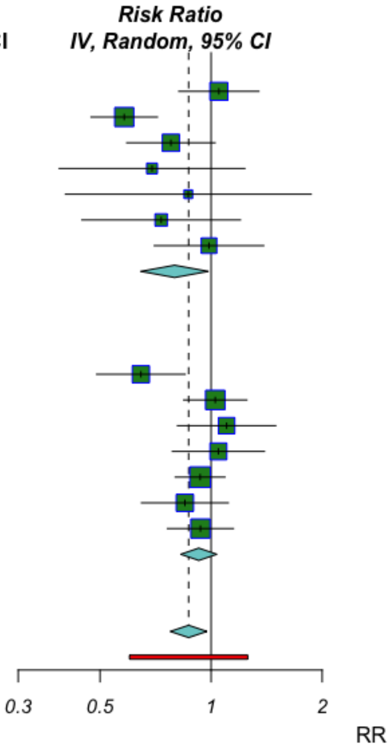

Figure D. Summary forest plot for sensitivity analysis on all-MI by asymptomatic PAD (excluding asymptomatic PAD trials)

Study or Subgroup	experiment Events	experiment Total	control Events	control Total	Weight	Risk Ratio IV, Random, 95% CI
no						
Peto et al (BDS) 1988	181	3429	86	1710	8.3%	1.05 [0.82; 1.35]
Steering et al (PHS) 1989	139	11037	239	11034	9.4%	0.58 [0.47; 0.72]
Hansson et al (HOT) 1998	82	9399	127	9391	7.6%	0.65 [0.49; 0.85]
Meade et al (TPT) 1998	83	1268	107	1272	7.6%	0.78 [0.59; 1.03]
de Gaetano et al (PPP) 2001	19	2226	28	2269	3.0%	0.69 [0.39; 1.23]
Ridker et al (WHS) 2005	198	19934	193	19942	9.7%	1.03 [0.84; 1.25]
Ogawa et al (JPAD) 2008	12	1262	14	1277	1.9%	0.87 [0.40; 1.87]
Ikeda et al (JPPP) 2014	27	7220	37	7244	3.9%	0.73 [0.45; 1.20]
Satio et al (JPAD2) 2017	57	992	68	1168	6.2%	0.99 [0.70; 1.39]
Bowman et al (ASCEND) 2018	296	7740	317	7740	10.9%	0.93 [0.80; 1.09]
Gaziano et al (ARRIVE) 2018	95	6270	112	6276	7.7%	0.85 [0.65; 1.11]
McNeil et al (ASPREE) 2018	171	9525	184	9589	9.5%	0.94 [0.76; 1.15]
Total (95% CI)		80302		78912	85.8%	0.84 [0.74; 0.95]
Heterogeneity: $Tau^2 = 0.0256$; $Chi^2 = 27.14$, df = 11 ($P < 0.01$); $I^2 = 59\%$						
yes						
Belch et al (POPADAD) 2008	76	638	69	638	6.9%	1.10 [0.81; 1.50]
Fowkes et al (AAA) 2010	90	1675	86	1675	7.3%	1.05 [0.78; 1.40]
Total (95% CI)		2313		2313	14.2%	1.07 [0.87; 1.32]
Heterogeneity: $Tau^2 = 0$; $Chi^2 = 0.06$, df = 1 ($P = 0.81$); $I^2 = 0\%$						
Total (95% CI)		82615		81225	100.0%	0.87 [0.77; 0.97]
**Prediction interval**						**[0.60; 1.26]**

Heterogeneity: $Tau^2 = 0.0253$; $Chi^2 = 31.22$, df = 13 ($P < 0.01$); $I^2 = 58\%$
Residual heterogeneity: Tau^2 = NA; $Chi^2 = 27.20$, df = 12 ($P < 0.01$); $I^2 = 56\%$
Test for overall effect: Z = -2.39 (P = 0.02)

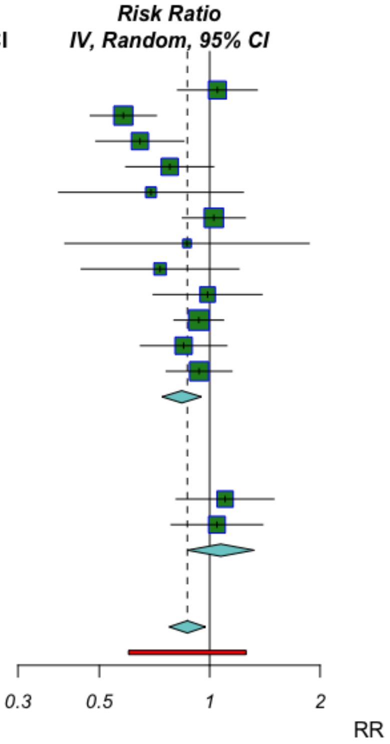

Figure E. Summary forest plot for sensitivity analysis on all-MI by male populations (excluding 100% male individual trials)

Study or Subgroup	experiment Events	experiment Total	control Events	control Total	Weight	Risk Ratio IV, Random, 95% CI
male						
Peto et al (BDS) 1988	181	3429	86	1710	8.3%	1.05 [0.82; 1.35]
Steering et al (PHS) 1989	139	11037	239	11034	9.4%	0.58 [0.47; 0.72]
Meade et al (TPT) 1998	83	1268	107	1272	7.6%	0.78 [0.59; 1.03]
Total (95% CI)		15734		14016	25.3%	0.78 [0.54; 1.11]
Heterogeneity: Tau^2 = 0.0829; Chi^2 = 12.79, df = 2 (P < 0.01); I^2 = 84%						
not all male						
Hansson et al (HOT) 1998	82	9399	127	9391	7.6%	0.65 [0.49; 0.85]
de Gaetano et al (PPP) 2001	19	2226	28	2269	3.0%	0.69 [0.39; 1.23]
Ridker et al (WHS) 2005	198	19934	193	19942	9.7%	1.03 [0.84; 1.25]
Belch et al (POPADAD) 2008	76	638	69	638	6.9%	1.10 [0.81; 1.50]
Ogawa et al (JPAD) 2008	12	1262	14	1277	1.9%	0.87 [0.40; 1.87]
Fowkes et al (AAA) 2010	90	1675	86	1675	7.3%	1.05 [0.78; 1.40]
Ikeda et al (JPPP) 2014	27	7220	37	7244	3.9%	0.73 [0.45; 1.20]
Satio et al (JPAD2) 2017	57	992	68	1168	6.2%	0.99 [0.70; 1.39]
Bowman et al (ASCEND) 2018	296	7740	317	7740	10.9%	0.93 [0.80; 1.09]
Gaziano et al (ARRIVE) 2018	95	6270	112	6276	7.7%	0.85 [0.65; 1.11]
McNeil et al (ASPREE) 2018	171	9525	184	9589	9.5%	0.94 [0.76; 1.15]
Total (95% CI)		66881		67209	74.7%	0.92 [0.84; 1.00]
Heterogeneity: Tau^2 = 0.0038; Chi^2 = 11.95, df = 10 (P = 0.29); I^2 = 16%						
**Total (95% CI)**		82615		81225	100.0%	0.87 [0.77; 0.97]
**Prediction interval**						**[0.60; 1.26]**

Heterogeneity: Tau^2 = 0.0253; Chi^2 = 31.22, df = 13 (P < 0.01); I^2 = 58%
Residual heterogeneity: Tau^2 = NA; Chi^2 = 24.74, df = 12 (P = 0.02); I^2 = 51%
Test for overall effect: Z = -2.39 (P = 0.02)

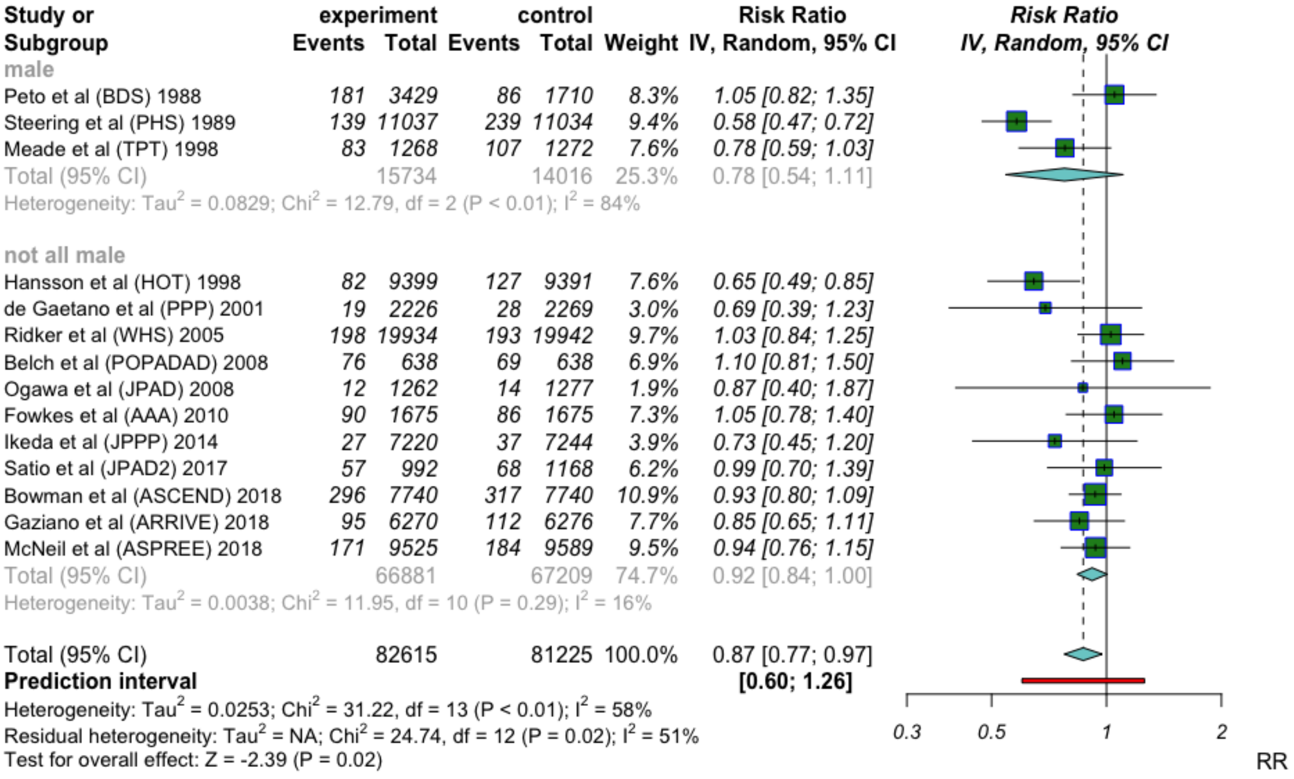

Figure F. Summary forest plot for sensitivity analysis on all-MI by diabetes status (excluding 100% diabetic individual trials and restricted on 100% diabetic individual trials)

Study or Subgroup	experiment Events	experiment Total	control Events	control Total	Weight	Risk Ratio IV, Random, 95% CI
no						
Peto et al (BDS) 1988	*181*	*3429*	*86*	*1710*	*8.3%*	*1.05 [0.82; 1.35]*
Steering et al (PHS) 1989	*139*	*11037*	*239*	*11034*	*9.4%*	*0.58 [0.47; 0.72]*
Hansson et al (HOT) 1998	*82*	*9399*	*127*	*9391*	*7.6%*	*0.65 [0.49; 0.85]*
Meade et al (TPT) 1998	*83*	*1268*	*107*	*1272*	*7.6%*	*0.78 [0.59; 1.03]*
de Gaetano et al (PPP) 2001	*19*	*2226*	*28*	*2269*	*3.0%*	*0.69 [0.39; 1.23]*
Ridker et al (WHS) 2005	*198*	*19934*	*193*	*19942*	*9.7%*	*1.03 [0.84; 1.25]*
Fowkes et al (AAA) 2010	*90*	*1675*	*86*	*1675*	*7.3%*	*1.05 [0.78; 1.40]*
Ikeda et al (JPPP) 2014	*27*	*7220*	*37*	*7244*	*3.9%*	*0.73 [0.45; 1.20]*
Gaziano et al (ARRIVE) 2018	*95*	*6270*	*112*	*6276*	*7.7%*	*0.85 [0.65; 1.11]*
McNeil et al (ASPREE) 2018	*171*	*9525*	*184*	*9589*	*9.5%*	*0.94 [0.76; 1.15]*
Total (95% CI)		71983		70402	74.1%	0.83 [0.72; 0.96]
Heterogeneity: $Tau^2 = 0.0362$; $Chi^2 = 26.93$, df = 9 ($P < 0.01$); $I^2 = 67\%$						
yes						
Belch et al (POPADAD) 2008	*76*	*638*	*69*	*638*	*6.9%*	*1.10 [0.81; 1.50]*
Ogawa et al (JPAD) 2008	*12*	*1262*	*14*	*1277*	*1.9%*	*0.87 [0.40; 1.87]*
Satio et al (JPAD2) 2017	*57*	*992*	*68*	*1168*	*6.2%*	*0.99 [0.70; 1.39]*
Bowman et al (ASCEND) 2018	*296*	*7740*	*317*	*7740*	*10.9%*	*0.93 [0.80; 1.09]*
Total (95% CI)		10632		10823	25.9%	0.97 [0.85; 1.10]
Heterogeneity: $Tau^2 = 0$; $Chi^2 = 0.98$, df = 3 ($P = 0.81$); $I^2 = 0\%$						
Total (95% CI)		82615		81225	100.0%	0.87 [0.77; 0.97]
**Prediction interval**						**[0.60; 1.26]**

Heterogeneity: $Tau^2 = 0.0253$; $Chi^2 = 31.22$, df = 13 ($P < 0.01$); $I^2 = 58\%$
Residual heterogeneity: Tau^2 = NA; $Chi^2 = 27.90$, df = 12 ($P < 0.01$); $I^2 = 57\%$
Test for overall effect: $Z = -2.39$ ($P = 0.02$)

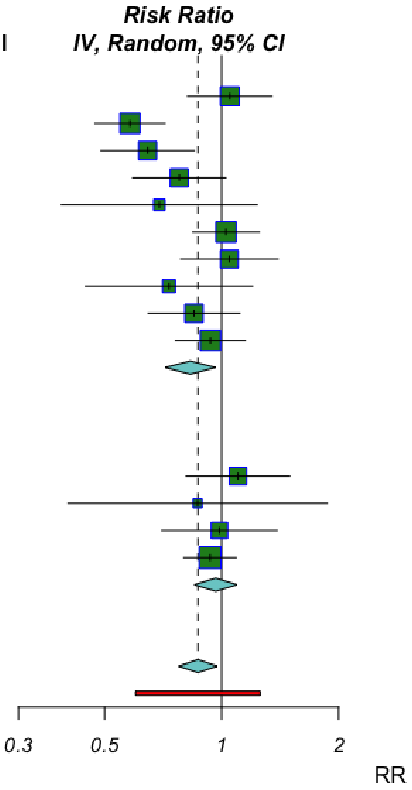

Figure G. Summary forest plot for sensitivity analysis on all-MI by placebo use (excluding placebo use trials)

Study or Subgroup	experiment Events	experiment Total	control Events	control Total	Weight	Risk Ratio IV, Random, 95% CI
no						
Peto et al (BDS) 1988	*181*	*3429*	*86*	*1710*	*8.3%*	*1.05 [0.82; 1.35]*
de Gaetano et al (PPP) 2001	*19*	*2226*	*28*	*2269*	*3.0%*	*0.69 [0.39; 1.23]*
Ogawa et al (JPAD) 2008	*12*	*1262*	*14*	*1277*	*1.9%*	*0.87 [0.40; 1.87]*
Ikeda et al (JPPP) 2014	*27*	*7220*	*37*	*7244*	*3.9%*	*0.73 [0.45; 1.20]*
Satio et al (JPAD2) 2017	*57*	*992*	*68*	*1168*	*6.2%*	*0.99 [0.70; 1.39]*
Total (95% CI)		15129		13668	23.3%	0.94 [0.79; 1.12]
Heterogeneity: $Tau^2 = 0$; $Chi^2 = 2.92$, df = 4 (P = 0.57); $I^2 = 0\%$						
yes						
Steering et al (PHS) 1989	*139*	*11037*	*239*	*11034*	*9.4%*	*0.58 [0.47; 0.72]*
Hansson et al (HOT) 1998	*82*	*9399*	*127*	*9391*	*7.6%*	*0.65 [0.49; 0.85]*
Meade et al (TPT) 1998	*83*	*1268*	*107*	*1272*	*7.6%*	*0.78 [0.59; 1.03]*
Ridker et al (WHS) 2005	*198*	*19934*	*193*	*19942*	*9.7%*	*1.03 [0.84; 1.25]*
Belch et al (POPADAD) 2008	*76*	*638*	*69*	*638*	*6.9%*	*1.10 [0.81; 1.50]*
Fowkes et al (AAA) 2010	*90*	*1675*	*86*	*1675*	*7.3%*	*1.05 [0.78; 1.40]*
Bowman et al (ASCEND) 2018	*296*	*7740*	*317*	*7740*	*10.9%*	*0.93 [0.80; 1.09]*
Gaziano et al (ARRIVE) 2018	*95*	*6270*	*112*	*6276*	*7.7%*	*0.85 [0.65; 1.11]*
McNeil et al (ASPREE) 2018	*171*	*9525*	*184*	*9589*	*9.5%*	*0.94 [0.76; 1.15]*
Total (95% CI)		67486		67557	76.7%	0.86 [0.74; 0.99]
Heterogeneity: $Tau^2 = 0.0332$; $Chi^2 = 27.44$, df = 8 (P < 0.01); $I^2 = 71\%$						
Total (95% CI)		82615		81225	100.0%	0.87 [0.77; 0.97]
**Prediction interval**						**[0.60; 1.26]**

Heterogeneity: $Tau^2 = 0.0253$; $Chi^2 = 31.22$, df = 13 (P < 0.01); $I^2 = 58\%$
Residual heterogeneity: $Tau^2 = NA$; $Chi^2 = 30.36$, df = 12 (P < 0.01); $I^2 = 60\%$
Test for overall effect: Z = -2.39 (P = 0.02)

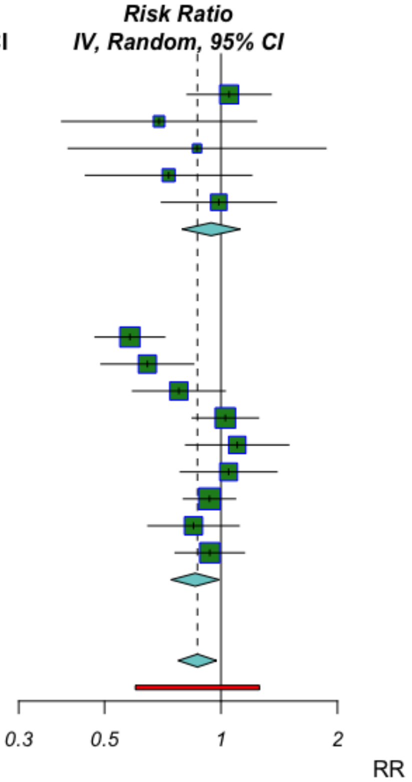

Figure H. Summary forest plot for sensitivity analysis on all-MI by excluding TPT trial

Study or Subgroup	experiment Events	experiment Total	control Events	control Total	Weight	Risk Ratio IV, Random, 95% CI
no						
Peto et al (BDS) 1988	*181*	*3429*	*86*	*1710*	*8.3%*	*1.05 [0.82; 1.35]*
Steering et al (PHS) 1989	*139*	*11037*	*239*	*11034*	*9.4%*	*0.58 [0.47; 0.72]*
Hansson et al (HOT) 1998	*82*	*9399*	*127*	*9391*	*7.6%*	*0.65 [0.49; 0.85]*
de Gaetano et al (PPP) 2001	*19*	*2226*	*28*	*2269*	*3.0%*	*0.69 [0.39; 1.23]*
Ridker et al (WHS) 2005	*198*	*19934*	*193*	*19942*	*9.7%*	*1.03 [0.84; 1.25]*
Belch et al (POPADAD) 2008	*76*	*638*	*69*	*638*	*6.9%*	*1.10 [0.81; 1.50]*
Ogawa et al (JPAD) 2008	*12*	*1262*	*14*	*1277*	*1.9%*	*0.87 [0.40; 1.87]*
Fowkes et al (AAA) 2010	*90*	*1675*	*86*	*1675*	*7.3%*	*1.05 [0.78; 1.40]*
Ikeda et al (JPPP) 2014	*27*	*7220*	*37*	*7244*	*3.9%*	*0.73 [0.45; 1.20]*
Satio et al (JPAD2) 2017	*57*	*992*	*68*	*1168*	*6.2%*	*0.99 [0.70; 1.39]*
Bowman et al (ASCEND) 2018	*296*	*7740*	*317*	*7740*	*10.9%*	*0.93 [0.80; 1.09]*
Gaziano et al (ARRIVE) 2018	*95*	*6270*	*112*	*6276*	*7.7%*	*0.85 [0.65; 1.11]*
McNeil et al (ASPREE) 2018	*171*	*9525*	*184*	*9589*	*9.5%*	*0.94 [0.76; 1.15]*
Total (95% CI)		81347		79953	92.4%	0.88 [0.78; 0.99]
Heterogeneity: $Tau^2 = 0.0276$; $Chi^2 = 30.48$, df = 12 ($P < 0.01$); $I^2 = 61\%$						
yes						
Meade et al (TPT) 1998	*83*	*1268*	*107*	*1272*	*7.6%*	*0.78 [0.59; 1.03]*
Total (95% CI)		1268		1272	7.6%	0.78 [0.59; 1.03]
Heterogeneity: not applicable						
Total (95% CI)		82615		81225	100.0%	0.87 [0.77; 0.97]
**Prediction interval**						**[0.60; 1.26]**

Heterogeneity: $Tau^2 = 0.0253$; $Chi^2 = 31.22$, df = 13 ($P < 0.01$); $I^2 = 58\%$
Residual heterogeneity: Tau^2 = NA; $Chi^2 = 30.48$, df = 12 ($P < 0.01$); $I^2 = 61\%$
Test for overall effect: $Z = -2.39$ ($P = 0.02$)

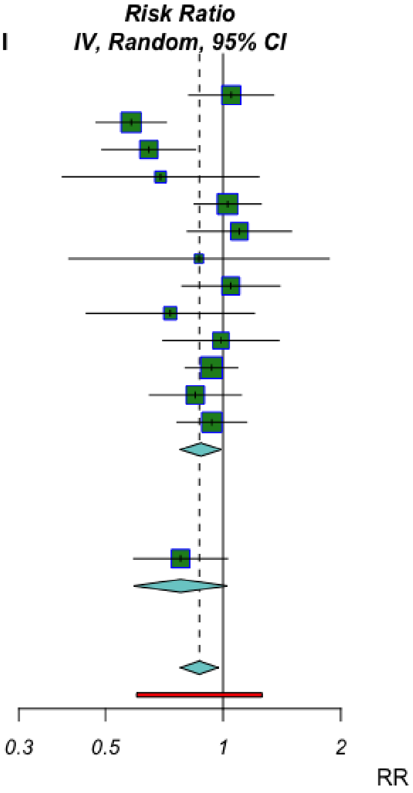

Supplement: Supplementary file 7 [file datasheet4.pdf]
