# Supplementary material for: Pros and Cons of Aspirin for the Primary Prevention of Cardiovascular Events: A Secondary Study of Trial Sequential Analysis
Source: Front Pharmacol. 2021 Jan 14;11:592116. doi: 10.3389/fphar.2020.592116 (PMC7845480; doi:10.3389/fphar.2020.592116)

Appendix File S6. Sensitivity analyses on total stroke.

Figure A. Summary forest plot for sensitivity analysis on total stroke by year (excluding trials published before 2000)

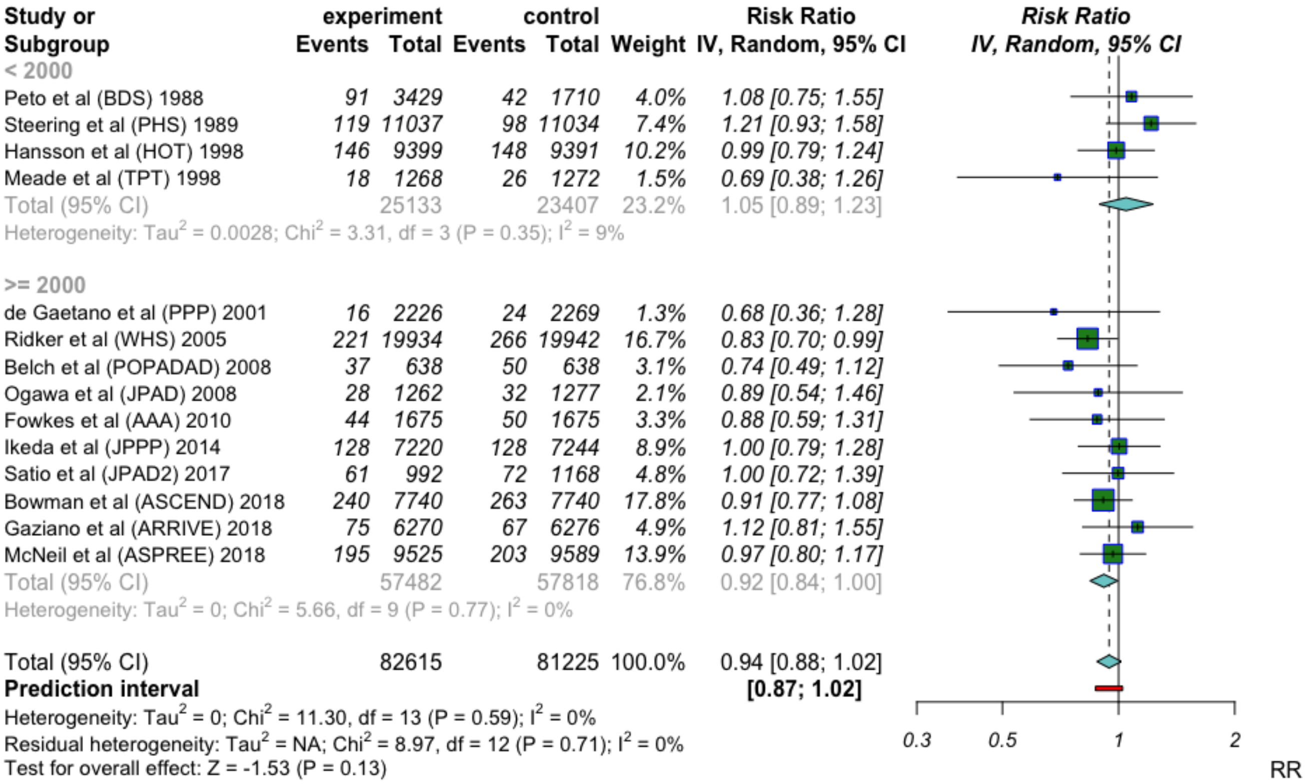

Figure B. Summary forest plot for sensitivity analysis on total stroke by blinding (excluding open-label trials)

Study or Subgroup	experiment Events	experiment Total	control Events	control Total	Weight	Risk Ratio IV, Random, 95% CI
**double-blind**						
Steering et al (PHS) 1989	*119*	*11037*	*98*	*11034*	*7.4%*	*1.21 [0.93; 1.58]*
Hansson et al (HOT) 1998	*146*	*9399*	*148*	*9391*	*10.2%*	*0.99 [0.79; 1.24]*
Meade et al (TPT) 1998	*18*	*1268*	*26*	*1272*	*1.5%*	*0.69 [0.38; 1.26]*
Ridker et al (WHS) 2005	*221*	*19934*	*266*	*19942*	*16.7%*	*0.83 [0.70; 0.99]*
Belch et al (POPADAD) 2008	*37*	*638*	*50*	*638*	*3.1%*	*0.74 [0.49; 1.12]*
Fowkes et al (AAA) 2010	*44*	*1675*	*50*	*1675*	*3.3%*	*0.88 [0.59; 1.31]*
Bowman et al (ASCEND) 2018	*240*	*7740*	*263*	*7740*	*17.8%*	*0.91 [0.77; 1.08]*
Gaziano et al (ARRIVE) 2018	*75*	*6270*	*67*	*6276*	*4.9%*	*1.12 [0.81; 1.55]*
McNeil et al (ASPREE) 2018	*195*	*9525*	*203*	*9589*	*13.9%*	*0.97 [0.80; 1.17]*
Total (95% CI)		67486		67557	78.8%	0.94 [0.86; 1.03]
Heterogeneity: $Tau^2 = 0.0026$; $Chi^2 = 9.26$, df = 8 (P = 0.32); $I^2 = 14\%$						
**open-label**						
Peto et al (BDS) 1988	*91*	*3429*	*42*	*1710*	*4.0%*	*1.08 [0.75; 1.55]*
de Gaetano et al (PPP) 2001	*16*	*2226*	*24*	*2269*	*1.3%*	*0.68 [0.36; 1.28]*
Ogawa et al (JPAD) 2008	*28*	*1262*	*32*	*1277*	*2.1%*	*0.89 [0.54; 1.46]*
Ikeda et al (JPPP) 2014	*128*	*7220*	*128*	*7244*	*8.9%*	*1.00 [0.79; 1.28]*
Satio et al (JPAD2) 2017	*61*	*992*	*72*	*1168*	*4.8%*	*1.00 [0.72; 1.39]*
Total (95% CI)		15129		13668	21.2%	0.98 [0.84; 1.15]
Heterogeneity: $Tau^2 = 0$; $Chi^2 = 1.78$, df = 4 (P = 0.78); $I^2 = 0\%$						
Total (95% CI)		82615		81225	100.0%	0.94 [0.88; 1.02]
**Prediction interval**						**[0.87; 1.02]**

Heterogeneity: $Tau^2 = 0$; $Chi^2 = 11.30$, df = 13 (P = 0.59); $I^2 = 0\%$
Residual heterogeneity: Tau^2 = NA; $Chi^2 = 11.04$, df = 12 (P = 0.53); $I^2 = 0\%$
Test for overall effect: Z = -1.53 (P = 0.13)

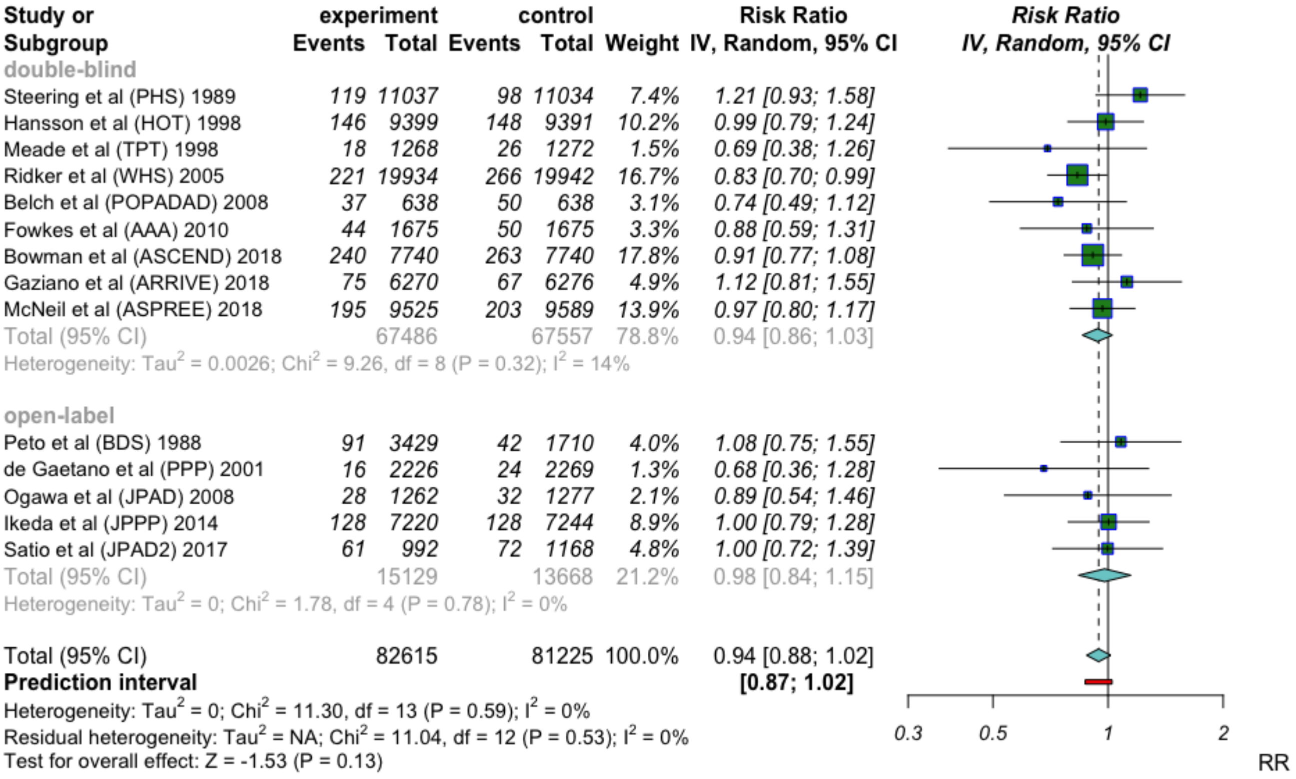

Figure C. Summary forest plot for sensitivity analysis on total stroke by study quality (excluding high risk trials)

Study or Subgroup	experiment Events	experiment Total	control Events	control Total	Weight	Risk Ratio IV, Random, 95% CI
**High risk**						
Peto et al (BDS) 1988	91	3429	42	1710	4.0%	1.08 [0.75; 1.55]
Steering et al (PHS) 1989	119	11037	98	11034	7.4%	1.21 [0.93; 1.58]
Meade et al (TPT) 1998	18	1268	26	1272	1.5%	0.69 [0.38; 1.26]
de Gaetano et al (PPP) 2001	16	2226	24	2269	1.3%	0.68 [0.36; 1.28]
Ogawa et al (JPAD) 2008	28	1262	32	1277	2.1%	0.89 [0.54; 1.46]
Ikeda et al (JPPP) 2014	128	7220	128	7244	8.9%	1.00 [0.79; 1.28]
Satio et al (JPAD2) 2017	61	992	72	1168	4.8%	1.00 [0.72; 1.39]
Total (95% CI)		27434		25974	30.1%	1.02 [0.89; 1.16]
Heterogeneity: $Tau^2 = 0$; $Chi^2 = 5.28$, df = 6 (P = 0.51); $I^2 = 0\%$						
**Low-risk**						
Hansson et al (HOT) 1998	146	9399	148	9391	10.2%	0.99 [0.79; 1.24]
Ridker et al (WHS) 2005	221	19934	266	19942	16.7%	0.83 [0.70; 0.99]
Belch et al (POPADAD) 2008	37	638	50	638	3.1%	0.74 [0.49; 1.12]
Fowkes et al (AAA) 2010	44	1675	50	1675	3.3%	0.88 [0.59; 1.31]
Bowman et al (ASCEND) 2018	240	7740	263	7740	17.8%	0.91 [0.77; 1.08]
Gaziano et al (ARRIVE) 2018	75	6270	67	6276	4.9%	1.12 [0.81; 1.55]
McNeil et al (ASPREE) 2018	195	9525	203	9589	13.9%	0.97 [0.80; 1.17]
Total (95% CI)		55181		55251	69.9%	0.92 [0.84; 1.00]
Heterogeneity: $Tau^2 = 0$; $Chi^2 = 4.38$, df = 6 (P = 0.62); $I^2 = 0\%$						
Total (95% CI)		82615		81225	100.0%	0.94 [0.88; 1.02]
**Prediction interval**						**[0.87; 1.02]**

Heterogeneity: $Tau^2 = 0$; $Chi^2 = 11.30$, df = 13 (P = 0.59); $I^2 = 0\%$
Residual heterogeneity: Tau^2 = NA; $Chi^2 = 9.66$, df = 12 (P = 0.65); $I^2 = 0\%$
Test for overall effect: Z = -1.53 (P = 0.13)

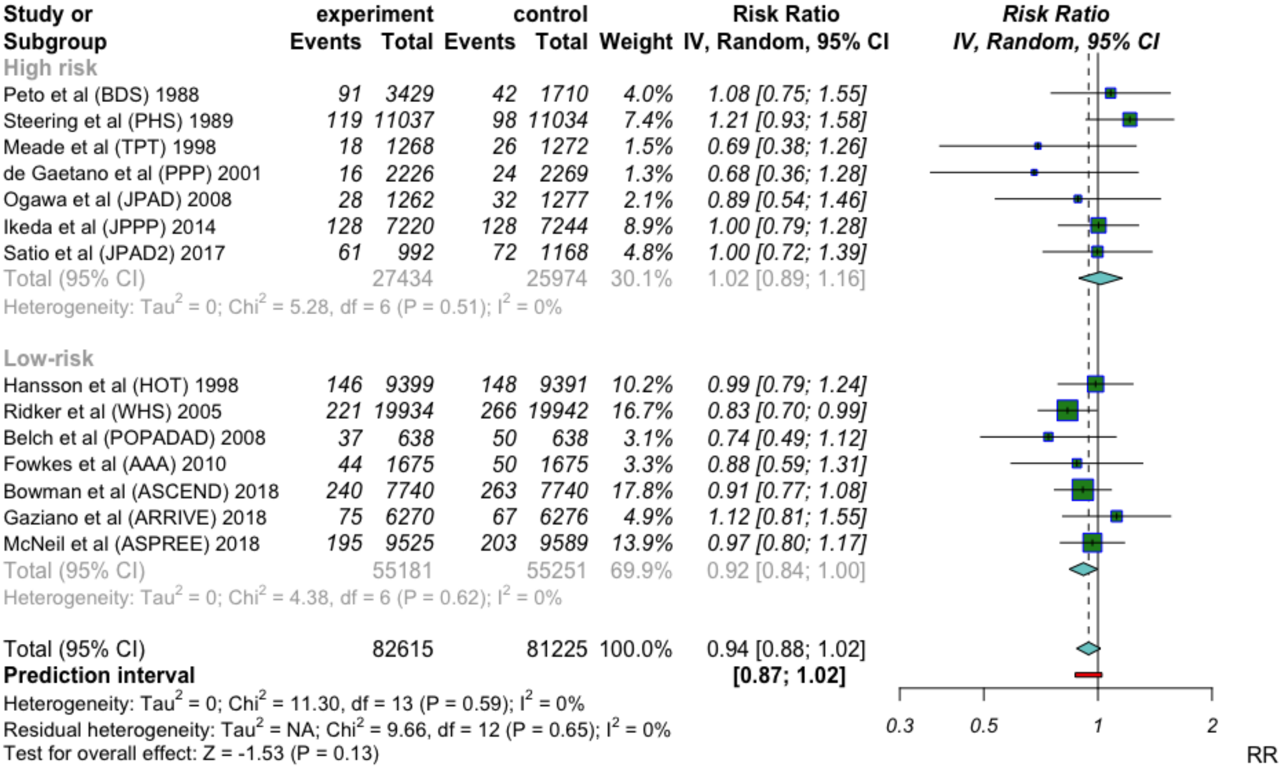

Figure D. Summary forest plot for sensitivity analysis on total stroke by asymptomatic PAD (excluding asymptomatic PAD trials)

Study or Subgroup	experiment Events	experiment Total	control Events	control Total	Weight	Risk Ratio IV, Random, 95% CI
no						
Peto et al (BDS) 1988	91	3429	42	1710	4.0%	1.08 [0.75; 1.55]
Steering et al (PHS) 1989	119	11037	98	11034	7.4%	1.21 [0.93; 1.58]
Hansson et al (HOT) 1998	146	9399	148	9391	10.2%	0.99 [0.79; 1.24]
Meade et al (TPT) 1998	18	1268	26	1272	1.5%	0.69 [0.38; 1.26]
de Gaetano et al (PPP) 2001	16	2226	24	2269	1.3%	0.68 [0.36; 1.28]
Ridker et al (WHS) 2005	221	19934	266	19942	16.7%	0.83 [0.70; 0.99]
Ogawa et al (JPAD) 2008	28	1262	32	1277	2.1%	0.89 [0.54; 1.46]
Ikeda et al (JPPP) 2014	128	7220	128	7244	8.9%	1.00 [0.79; 1.28]
Satio et al (JPAD2) 2017	61	992	72	1168	4.8%	1.00 [0.72; 1.39]
Bowman et al (ASCEND) 2018	240	7740	263	7740	17.8%	0.91 [0.77; 1.08]
Gaziano et al (ARRIVE) 2018	75	6270	67	6276	4.9%	1.12 [0.81; 1.55]
McNeil et al (ASPREE) 2018	195	9525	203	9589	13.9%	0.97 [0.80; 1.17]
Total (95% CI)		80302		78912	93.6%	0.95 [0.89; 1.03]
Heterogeneity: $Tau^2 = 0$; $Chi^2 = 9.73$, df = 11 (P = 0.55); $I^2 = 0\%$						
yes						
Belch et al (POPADAD) 2008	37	638	50	638	3.1%	0.74 [0.49; 1.12]
Fowkes et al (AAA) 2010	44	1675	50	1675	3.3%	0.88 [0.59; 1.31]
Total (95% CI)		2313		2313	6.4%	0.81 [0.61; 1.08]
Heterogeneity: $Tau^2 = 0$; $Chi^2 = 0.35$, df = 1 (P = 0.55); $I^2 = 0\%$						
**Total (95% CI)**		82615		81225	100.0%	0.94 [0.88; 1.02]
**Prediction interval**						**[0.87; 1.02]**

Heterogeneity: $Tau^2 = 0$; $Chi^2 = 11.30$, df = 13 (P = 0.59); $I^2 = 0\%$
Residual heterogeneity: Tau^2 = NA; $Chi^2 = 10.09$, df = 12 (P = 0.61); $I^2 = 0\%$
Test for overall effect: Z = -1.53 (P = 0.13)

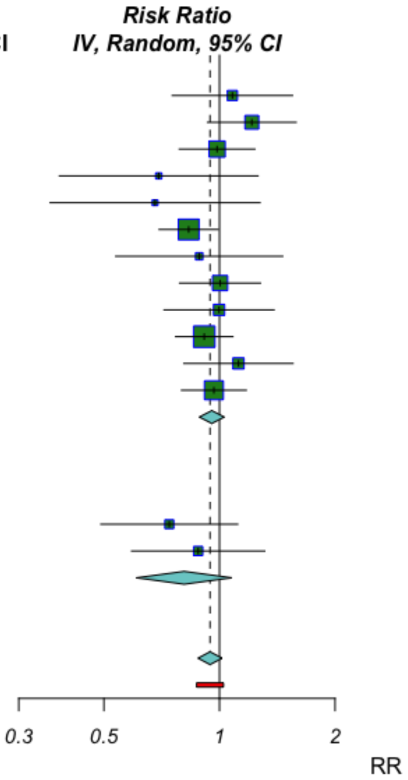

Figure E. Summary forest plot for sensitivity analysis on total stroke by male populations (excluding 100% male individual trials)

Study or Subgroup	experiment Events	experiment Total	control Events	control Total	Weight	Risk Ratio IV, Random, 95% CI
**male**						
Peto et al (BDS) 1988	91	3429	42	1710	4.0%	1.08 [0.75; 1.55]
Steering et al (PHS) 1989	119	11037	98	11034	7.4%	1.21 [0.93; 1.58]
Meade et al (TPT) 1998	18	1268	26	1272	1.5%	0.69 [0.38; 1.26]
Total (95% CI)		15734		14016	12.9%	1.07 [0.83; 1.38]
Heterogeneity: $Tau^2 = 0.0156$; $Chi^2 = 2.82$, df = 2 (P = 0.24); $I^2 = 29\%$						
**not all male**						
Hansson et al (HOT) 1998	146	9399	148	9391	10.2%	0.99 [0.79; 1.24]
de Gaetano et al (PPP) 2001	16	2226	24	2269	1.3%	0.68 [0.36; 1.28]
Ridker et al (WHS) 2005	221	19934	266	19942	16.7%	0.83 [0.70; 0.99]
Belch et al (POPADAD) 2008	37	638	50	638	3.1%	0.74 [0.49; 1.12]
Ogawa et al (JPAD) 2008	28	1262	32	1277	2.1%	0.89 [0.54; 1.46]
Fowkes et al (AAA) 2010	44	1675	50	1675	3.3%	0.88 [0.59; 1.31]
Ikeda et al (JPPP) 2014	128	7220	128	7244	8.9%	1.00 [0.79; 1.28]
Satio et al (JPAD2) 2017	61	992	72	1168	4.8%	1.00 [0.72; 1.39]
Bowman et al (ASCEND) 2018	240	7740	263	7740	17.8%	0.91 [0.77; 1.08]
Gaziano et al (ARRIVE) 2018	75	6270	67	6276	4.9%	1.12 [0.81; 1.55]
McNeil et al (ASPREE) 2018	195	9525	203	9589	13.9%	0.97 [0.80; 1.17]
Total (95% CI)		66881		67209	87.1%	0.92 [0.85; 1.00]
Heterogeneity: $Tau^2 = 0$; $Chi^2 = 6.01$, df = 10 (P = 0.81); $I^2 = 0\%$						
Total (95% CI)		82615		81225	100.0%	0.94 [0.88; 1.02]
**Prediction interval**						**[0.87; 1.02]**

Heterogeneity: $Tau^2 = 0$; $Chi^2 = 11.30$, df = 13 (P = 0.59); $I^2 = 0\%$
Residual heterogeneity: Tau^2 = NA; $Chi^2 = 8.84$, df = 12 (P = 0.72); $I^2 = 0\%$
Test for overall effect: Z = -1.53 (P = 0.13)

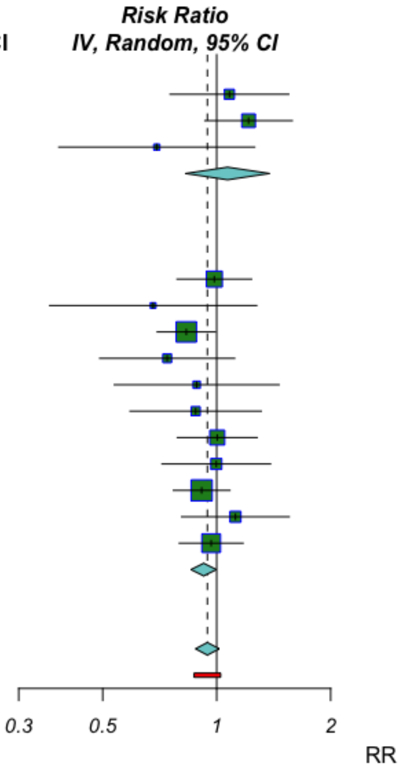

Figure F. Summary forest plot for sensitivity analysis on total stroke by diabetes status (excluding 100% diabetic individual trials and restricted on 100% diabetic individual trials)

Study or Subgroup	experiment Events	experiment Total	control Events	control Total	Weight	Risk Ratio IV, Random, 95% CI
no						
Peto et al (BDS) 1988	91	3429	42	1710	4.0%	1.08 [0.75; 1.55]
Steering et al (PHS) 1989	119	11037	98	11034	7.4%	1.21 [0.93; 1.58]
Hansson et al (HOT) 1998	146	9399	148	9391	10.2%	0.99 [0.79; 1.24]
Meade et al (TPT) 1998	18	1268	26	1272	1.5%	0.69 [0.38; 1.26]
de Gaetano et al (PPP) 2001	16	2226	24	2269	1.3%	0.68 [0.36; 1.28]
Ridker et al (WHS) 2005	221	19934	266	19942	16.7%	0.83 [0.70; 0.99]
Fowkes et al (AAA) 2010	44	1675	50	1675	3.3%	0.88 [0.59; 1.31]
Ikeda et al (JPPP) 2014	128	7220	128	7244	8.9%	1.00 [0.79; 1.28]
Gaziano et al (ARRIVE) 2018	75	6270	67	6276	4.9%	1.12 [0.81; 1.55]
McNeil et al (ASPREE) 2018	195	9525	203	9589	13.9%	0.97 [0.80; 1.17]
Total (95% CI)		71983		70402	72.2%	0.96 [0.88; 1.05]
Heterogeneity: $Tau^2 = 0.0010$; $Chi^2 = 9.45$, df = 9 (P = 0.40); $I^2 = 5\%$						
yes						
Belch et al (POPADAD) 2008	37	638	50	638	3.1%	0.74 [0.49; 1.12]
Ogawa et al (JPAD) 2008	28	1262	32	1277	2.1%	0.89 [0.54; 1.46]
Satio et al (JPAD2) 2017	61	992	72	1168	4.8%	1.00 [0.72; 1.39]
Bowman et al (ASCEND) 2018	240	7740	263	7740	17.8%	0.91 [0.77; 1.08]
Total (95% CI)		10632		10823	27.8%	0.90 [0.79; 1.04]
Heterogeneity: $Tau^2 = 0$; $Chi^2 = 1.27$, df = 3 (P = 0.74); $I^2 = 0\%$						
Total (95% CI)		82615		81225	100.0%	0.94 [0.88; 1.02]
**Prediction interval**						**[0.87; 1.02]**

Heterogeneity: $Tau^2 = 0$; $Chi^2 = 11.30$, df = 13 (P = 0.59); $I^2 = 0\%$
Residual heterogeneity: Tau^2 = NA; $Chi^2 = 10.72$, df = 12 (P = 0.55); $I^2 = 0\%$
Test for overall effect: Z = -1.53 (P = 0.13)

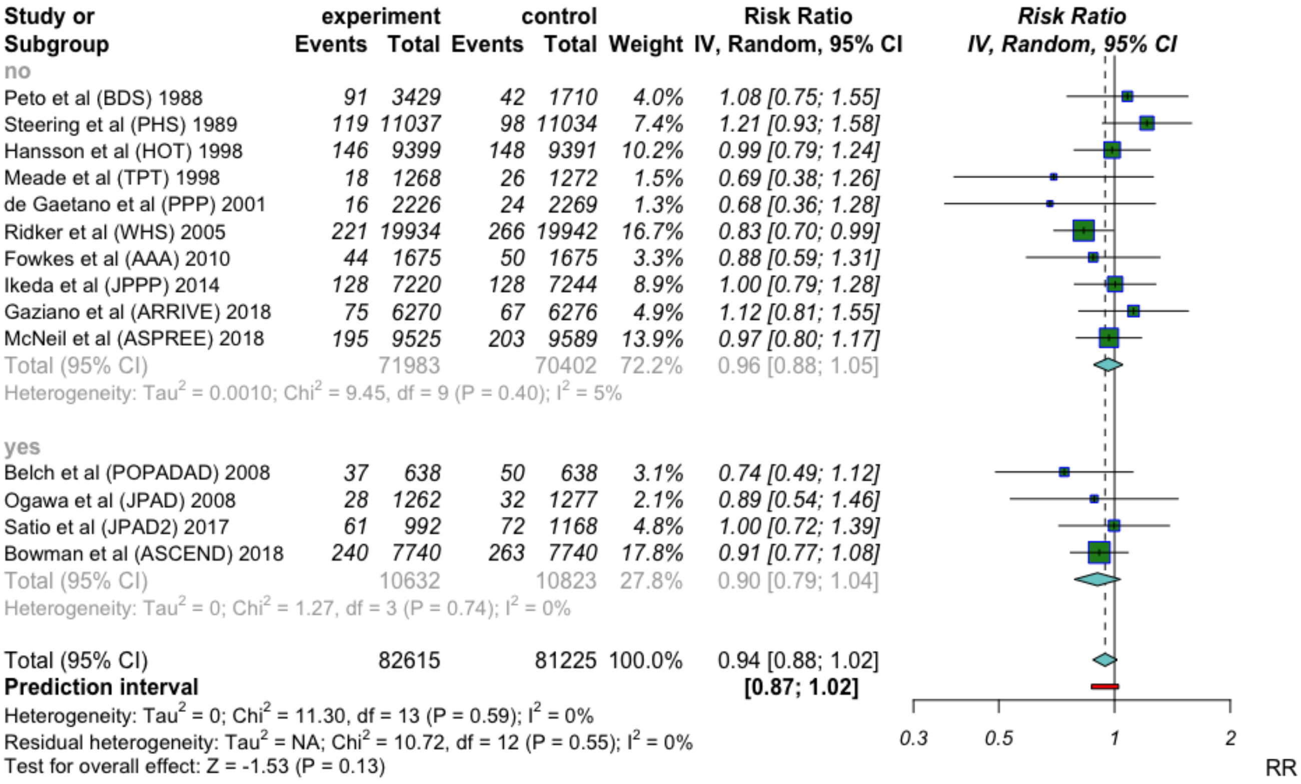

Figure G. Summary forest plot for sensitivity analysis on total stroke by placebo use (excluding placebo use trials)

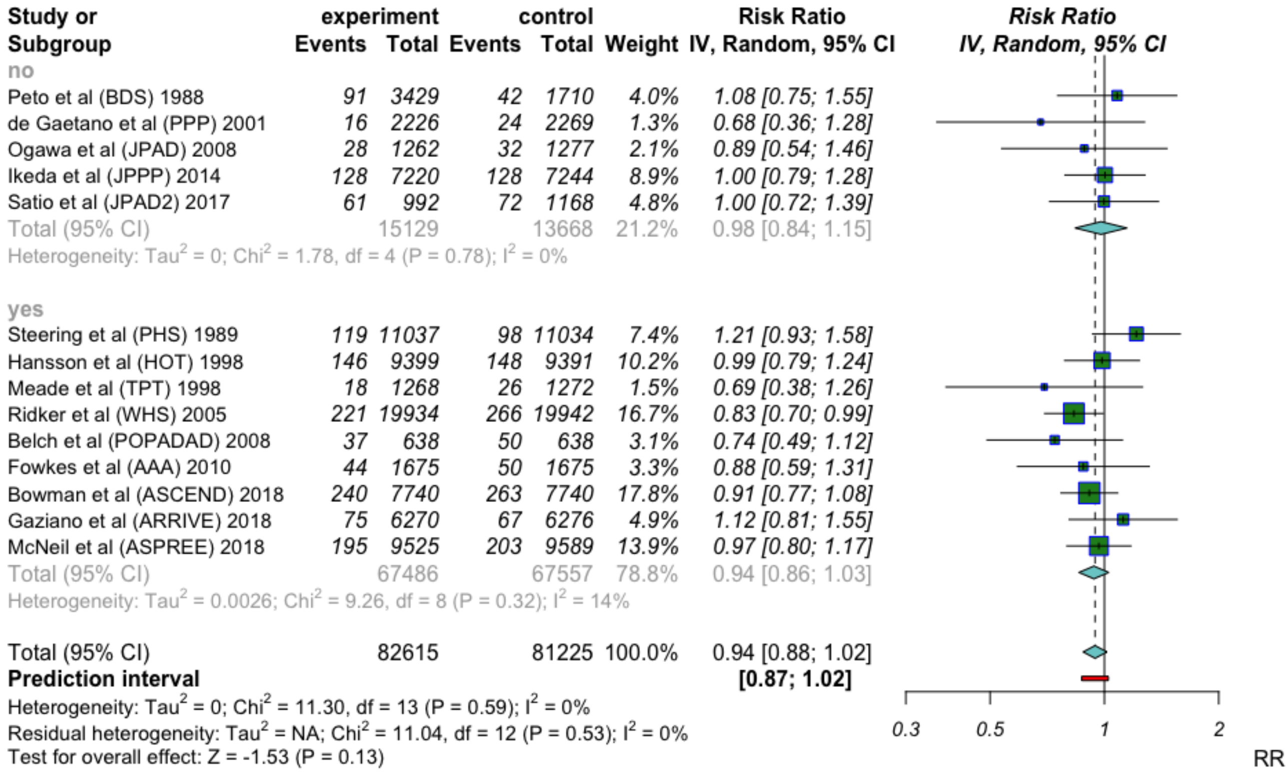

Figure H. Summary forest plot for sensitivity analysis on total stroke by excluding TPT trial

Study or Subgroup	experiment Events	experiment Total	control Events	control Total	Weight	Risk Ratio IV, Random, 95% CI
no						
Peto et al (BDS) 1988	91	3429	42	1710	4.0%	1.08 [0.75; 1.55]
Steering et al (PHS) 1989	119	11037	98	11034	7.4%	1.21 [0.93; 1.58]
Hansson et al (HOT) 1998	146	9399	148	9391	10.2%	0.99 [0.79; 1.24]
de Gaetano et al (PPP) 2001	16	2226	24	2269	1.3%	0.68 [0.36; 1.28]
Ridker et al (WHS) 2005	221	19934	266	19942	16.7%	0.83 [0.70; 0.99]
Belch et al (POPADAD) 2008	37	638	50	638	3.1%	0.74 [0.49; 1.12]
Ogawa et al (JPAD) 2008	28	1262	32	1277	2.1%	0.89 [0.54; 1.46]
Fowkes et al (AAA) 2010	44	1675	50	1675	3.3%	0.88 [0.59; 1.31]
Ikeda et al (JPPP) 2014	128	7220	128	7244	8.9%	1.00 [0.79; 1.28]
Satio et al (JPAD2) 2017	61	992	72	1168	4.8%	1.00 [0.72; 1.39]
Bowman et al (ASCEND) 2018	240	7740	263	7740	17.8%	0.91 [0.77; 1.08]
Gaziano et al (ARRIVE) 2018	75	6270	67	6276	4.9%	1.12 [0.81; 1.55]
McNeil et al (ASPREE) 2018	195	9525	203	9589	13.9%	0.97 [0.80; 1.17]
Total (95% CI)		81347		79953	98.5%	0.95 [0.88; 1.02]
Heterogeneity: $Tau^2 = 0$; $Chi^2 = 10.25$, df = 12 (P = 0.59); $I^2 = 0\%$						
yes						
Meade et al (TPT) 1998	18	1268	26	1272	1.5%	0.69 [0.38; 1.26]
Total (95% CI)		1268		1272	1.5%	0.69 [0.38; 1.26]
Heterogeneity: not applicable						
Total (95% CI)		82615		81225	100.0%	0.94 [0.88; 1.02]
**Prediction interval**						**[0.87; 1.02]**

Heterogeneity: $Tau^2 = 0$; $Chi^2 = 11.30$, df = 13 (P = 0.59); $I^2 = 0\%$
Residual heterogeneity: $Tau^2 = NA$; $Chi^2 = 10.25$, df = 12 (P = 0.59); $I^2 = 0\%$
Test for overall effect: Z = -1.53 (P = 0.13)

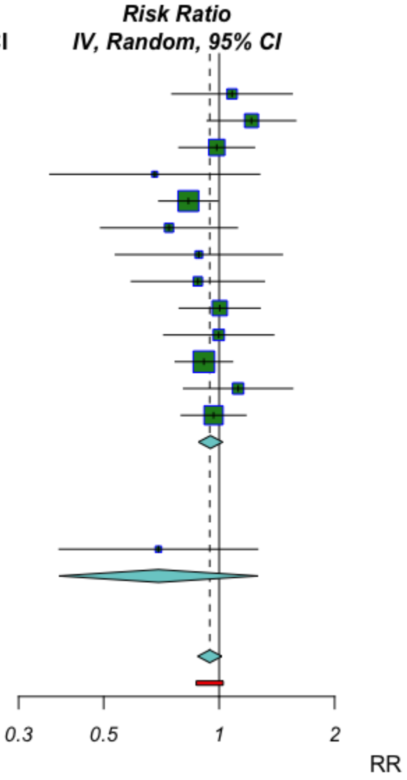

Supplement: Supplementary file 8 [file datasheet5.pdf]
