# Supplementary material for: Pros and Cons of Aspirin for the Primary Prevention of Cardiovascular Events: A Secondary Study of Trial Sequential Analysis
Source: Front Pharmacol. 2021 Jan 14;11:592116. doi: 10.3389/fphar.2020.592116 (PMC7845480; doi:10.3389/fphar.2020.592116)

Appendix File S7. Sensitivity analyses on ischaemic stroke.

Figure A. Summary forest plot for sensitivity analysis on ischemic stroke by year (excluding trials published before 2000)

Study or Subgroup	experiment Events	experiment Total	control Events	control Total	Weight	Risk Ratio IV, Random, 95% CI
< 2000						
Peto et al (BDS) 1988	21	3429	7	1710	1.1%	1.50 [0.64; 3.51]
Steering et al (PHS) 1989	91	11037	82	11034	9.3%	1.11 [0.82; 1.49]
Meade et al (TPT) 1998	10	1268	18	1272	1.4%	0.56 [0.26; 1.20]
Total (95% CI)		15734		14016	11.8%	1.00 [0.64; 1.57]
Heterogeneity: $Tau^2 = 0.0708$; $Chi^2 = 3.4$, df = 2 (P = 0.18); $I^2 = 41\%$						
>= 2000						
de Gaetano et al (PPP) 2001	14	2226	21	2269	1.8%	0.68 [0.35; 1.33]
Ridker et al (WHS) 2005	170	19934	221	19942	20.8%	0.77 [0.63; 0.94]
Ogawa et al (JPAD) 2008	22	1262	25	1277	2.6%	0.89 [0.50; 1.57]
Fowkes et al (AAA) 2010	30	1675	37	1675	3.6%	0.81 [0.50; 1.31]
Ikeda et al (JPPP) 2014	85	7220	101	7244	10.0%	0.84 [0.63; 1.12]
Satio et al (JPAD2) 2017	37	992	45	1168	4.5%	0.97 [0.63; 1.48]
Bowman et al (ASCEEND) 2018	240	7740	263	7740	27.8%	0.91 [0.77; 1.08]
McNeil et al (ASPREE) 2018	148	9525	167	9589	17.1%	0.89 [0.72; 1.11]
Total (95% CI)		50574		50904	88.2%	0.86 [0.78; 0.94]
Heterogeneity: $Tau^2 = 0$; $Chi^2 = 2.61$, df = 7 (P = 0.92); $I^2 = 0\%$						
Total (95% CI)		66308		64920	100.0%	0.88 [0.80; 0.96]
**Prediction interval**						**[0.79; 0.98]**

Heterogeneity: $Tau^2 = 0$; $Chi^2 = 8.06$, df = 10 (P = 0.62); $I^2 = 0\%$
Residual heterogeneity: $Tau^2 = NA$; $Chi^2 = 6.01$, df = 9 (P = 0.74); $I^2 = 0\%$
Test for overall effect: Z = -2.79 (P < 0.01)

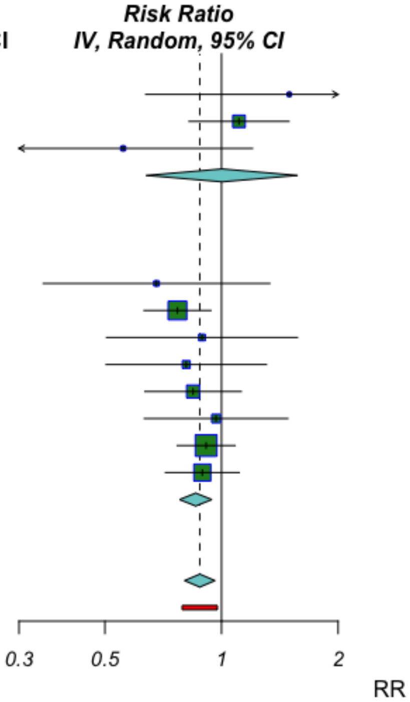

Figure B. Summary forest plot for sensitivity analysis on ischemic stroke by blinding (excluding open-label trials)

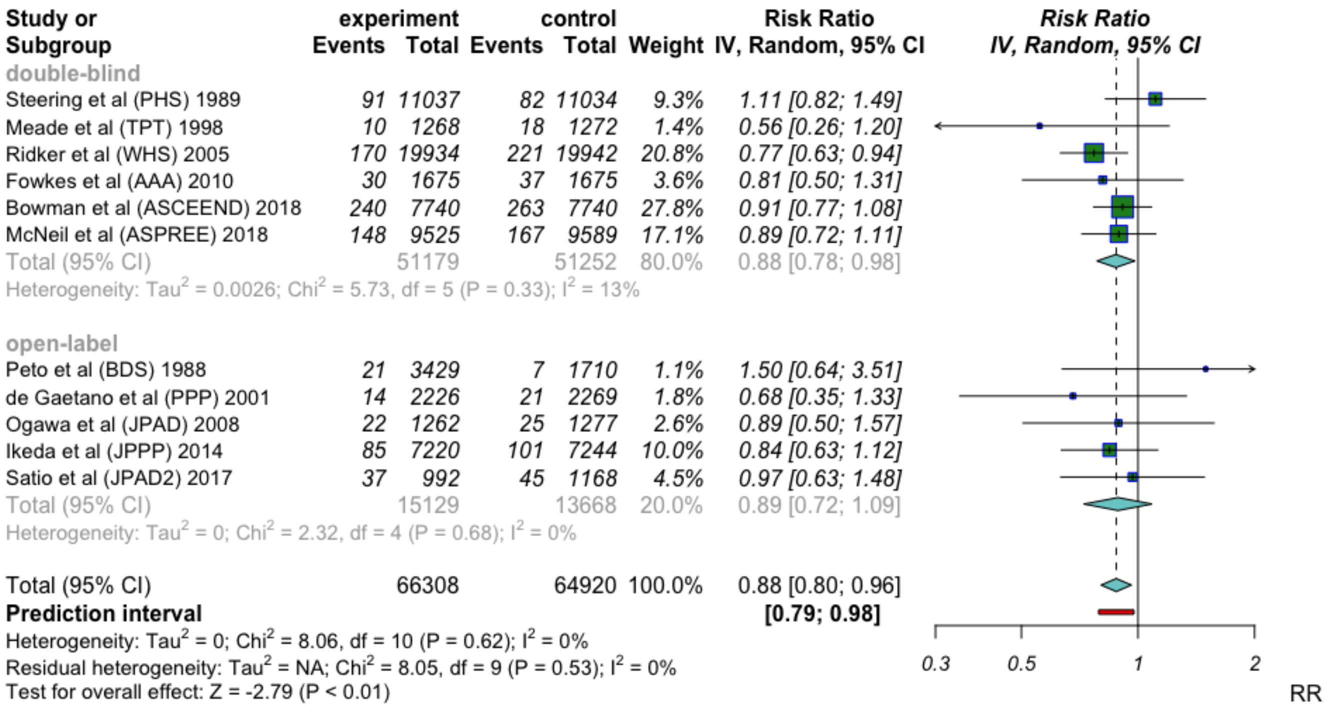

Figure C. Summary forest plot for sensitivity analysis on ischemic stroke by study quality (excluding high risk trials)

Study or Subgroup	experiment Events	experiment Total	control Events	control Total	Weight	Risk Ratio IV, Random, 95% CI
**High risk**						
Peto et al (BDS) 1988	*21*	*3429*	*7*	*1710*	*1.1%*	*1.50 [0.64; 3.51]*
Steering et al (PHS) 1989	*91*	*11037*	*82*	*11034*	*9.3%*	*1.11 [0.82; 1.49]*
Meade et al (TPT) 1998	*10*	*1268*	*18*	*1272*	*1.4%*	*0.56 [0.26; 1.20]*
Ogawa et al (JPAD) 2008	*22*	*1262*	*25*	*1277*	*2.6%*	*0.89 [0.50; 1.57]*
Ikeda et al (JPPP) 2014	*85*	*7220*	*101*	*7244*	*10.0%*	*0.84 [0.63; 1.12]*
Satio et al (JPAD2) 2017	*37*	*992*	*45*	*1168*	*4.5%*	*0.97 [0.63; 1.48]*
Total (95% CI)		25208		23705	28.9%	0.95 [0.80; 1.12]
Heterogeneity: $Tau^2 = 0$; $Chi^2 = 4.69$, df = 5 (P = 0.46); $I^2 = 0\%$						
**Low risk**						
de Gaetano et al (PPP) 2001	*14*	*2226*	*21*	*2269*	*1.8%*	*0.68 [0.35; 1.33]*
Ridker et al (WHS) 2005	*170*	*19934*	*221*	*19942*	*20.8%*	*0.77 [0.63; 0.94]*
Fowkes et al (AAA) 2010	*30*	*1675*	*37*	*1675*	*3.6%*	*0.81 [0.50; 1.31]*
Bowman et al (ASCEEND) 2018	*240*	*7740*	*263*	*7740*	*27.8%*	*0.91 [0.77; 1.08]*
McNeil et al (ASPREE) 2018	*148*	*9525*	*167*	*9589*	*17.1%*	*0.89 [0.72; 1.11]*
Total (95% CI)		41100		41215	71.1%	0.85 [0.76; 0.95]
Heterogeneity: $Tau^2 = 0$; $Chi^2 = 2.26$, df = 4 (P = 0.69); $I^2 = 0\%$						
Total (95% CI)		66308		64920	100.0%	0.88 [0.80; 0.96]
**Prediction interval**						**[0.79; 0.98]**

Heterogeneity: $Tau^2 = 0$; $Chi^2 = 8.06$, df = 10 (P = 0.62); $I^2 = 0\%$
Residual heterogeneity: $Tau^2 = NA$; $Chi^2 = 6.95$, df = 9 (P = 0.64); $I^2 = 0\%$
Test for overall effect: Z = -2.79 (P < 0.01)

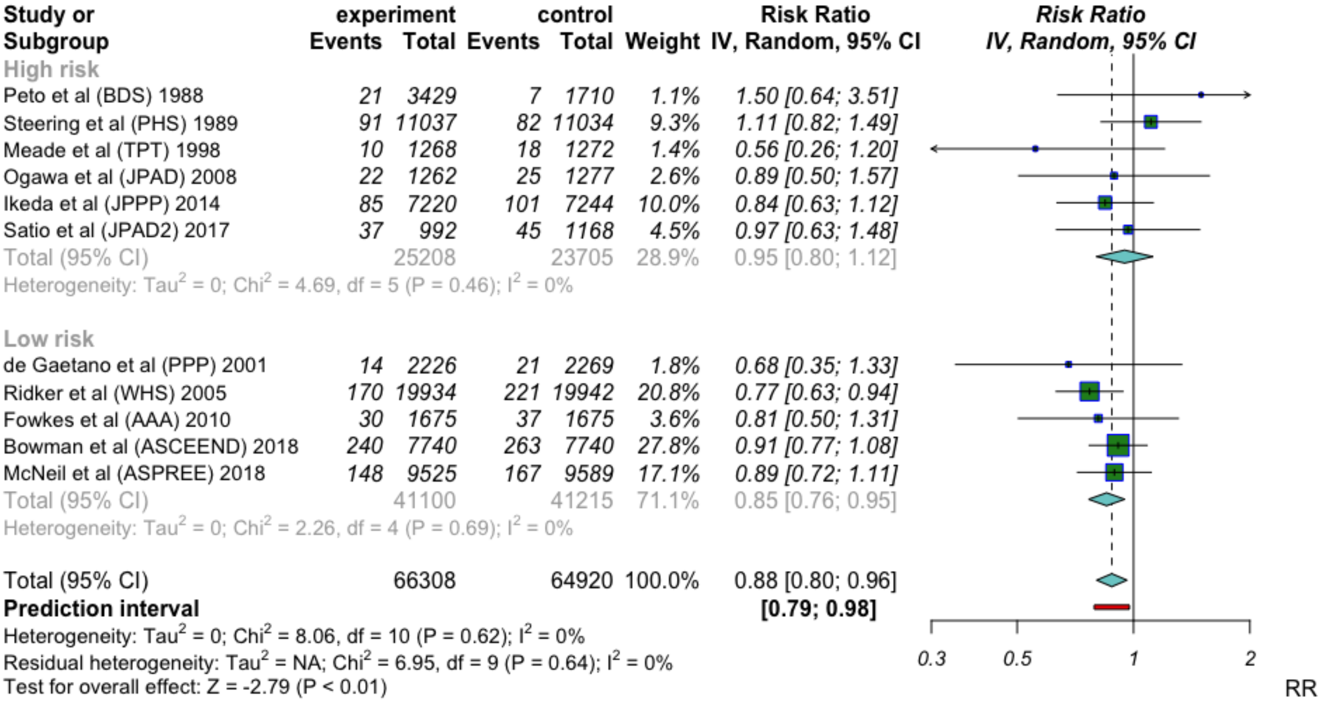

Figure D. Summary forest plot for sensitivity analysis on ischemic stroke by asymptomatic PAD (excluding asymptomatic PAD trials)

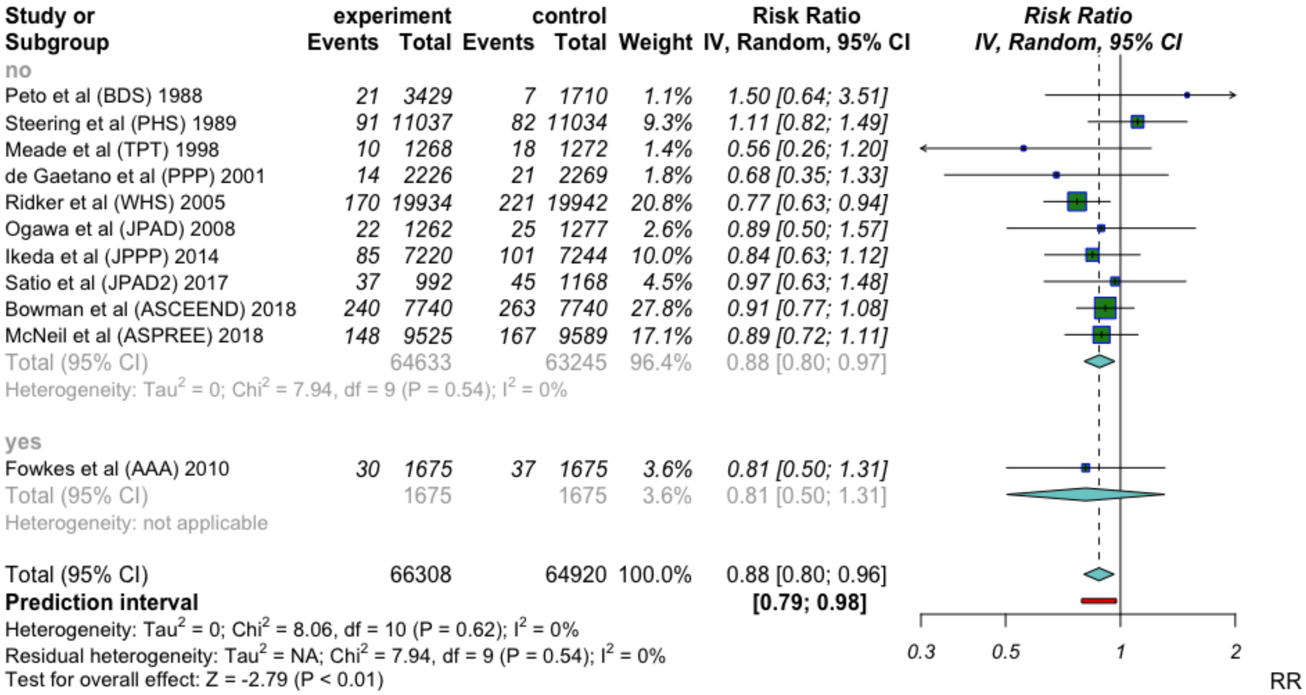

Figure E. Summary forest plot for sensitivity analysis on ischemic stroke by male populations (excluding 100% male individual trials)

Study or Subgroup	experiment Events	experiment Total	control Events	control Total	Weight	Risk Ratio IV, Random, 95% CI
male						
Peto et al (BDS) 1988	*21*	*3429*	*7*	*1710*	*1.1%*	*1.50 [0.64; 3.51]*
Steering et al (PHS) 1989	*91*	*11037*	*82*	*11034*	*9.3%*	*1.11 [0.82; 1.49]*
Meade et al (TPT) 1998	*10*	*1268*	*18*	*1272*	*1.4%*	*0.56 [0.26; 1.20]*
Total (95% CI)		15734		14016	11.8%	1.00 [0.64; 1.57]
Heterogeneity: $Tau^2 = 0.0708$; $Chi^2 = 3.4$, df = 2 (P = 0.18); $I^2 = 41\%$						
not all male						
de Gaetano et al (PPP) 2001	*14*	*2226*	*21*	*2269*	*1.8%*	*0.68 [0.35; 1.33]*
Ridker et al (WHS) 2005	*170*	*19934*	*221*	*19942*	*20.8%*	*0.77 [0.63; 0.94]*
Ogawa et al (JPAD) 2008	*22*	*1262*	*25*	*1277*	*2.6%*	*0.89 [0.50; 1.57]*
Fowkes et al (AAA) 2010	*30*	*1675*	*37*	*1675*	*3.6%*	*0.81 [0.50; 1.31]*
Ikeda et al (JPPP) 2014	*85*	*7220*	*101*	*7244*	*10.0%*	*0.84 [0.63; 1.12]*
Satio et al (JPAD2) 2017	*37*	*992*	*45*	*1168*	*4.5%*	*0.97 [0.63; 1.48]*
Bowman et al (ASCEEND) 2018	*240*	*7740*	*263*	*7740*	*27.8%*	*0.91 [0.77; 1.08]*
McNeil et al (ASPREE) 2018	*148*	*9525*	*167*	*9589*	*17.1%*	*0.89 [0.72; 1.11]*
Total (95% CI)		50574		50904	88.2%	0.86 [0.78; 0.94]
Heterogeneity: $Tau^2 = 0$; $Chi^2 = 2.61$, df = 7 (P = 0.92); $I^2 = 0\%$						
Total (95% CI)		66308		64920	100.0%	0.88 [0.80; 0.96]
**Prediction interval**						**[0.79; 0.98]**

Heterogeneity: $Tau^2 = 0$; $Chi^2 = 8.06$, df = 10 (P = 0.62); $I^2 = 0\%$
Residual heterogeneity: $Tau^2 = NA$; $Chi^2 = 6.01$, df = 9 (P = 0.74); $I^2 = 0\%$
Test for overall effect: Z = -2.79 (P < 0.01)

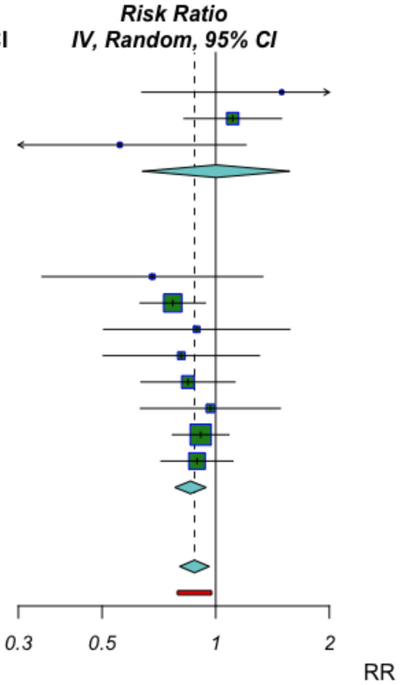

Figure F. Summary forest plot for sensitivity analysis on ischemic stroke by diabetes status (excluding 100% diabetic individual trials and restricted on 100% diabetic individual trials)

Study or Subgroup	experiment Events	experiment Total	control Events	control Total	Weight	Risk Ratio IV, Random, 95% CI
no						
Peto et al (BDS) 1988	21	3429	7	1710	1.1%	1.50 [0.64; 3.51]
Steering et al (PHS) 1989	91	11037	82	11034	9.3%	1.11 [0.82; 1.49]
Meade et al (TPT) 1998	10	1268	18	1272	1.4%	0.56 [0.26; 1.20]
de Gaetano et al (PPP) 2001	14	2226	21	2269	1.8%	0.68 [0.35; 1.33]
Ridker et al (WHS) 2005	170	19934	221	19942	20.8%	0.77 [0.63; 0.94]
Fowkes et al (AAA) 2010	30	1675	37	1675	3.6%	0.81 [0.50; 1.31]
Ikeda et al (JPPP) 2014	85	7220	101	7244	10.0%	0.84 [0.63; 1.12]
McNeil et al (ASPREE) 2018	148	9525	167	9589	17.1%	0.89 [0.72; 1.11]
Total (95% CI)		56314		54735	65.1%	0.86 [0.76; 0.97]
Heterogeneity: $Tau^2 = 0.0021$; $Chi^2 = 7.51$, df = 7 (P = 0.38); $I^2 = 7\%$						
yes						
Ogawa et al (JPAD) 2008	22	1262	25	1277	2.6%	0.89 [0.50; 1.57]
Satio et al (JPAD2) 2017	37	992	45	1168	4.5%	0.97 [0.63; 1.48]
Bowman et al (ASCEEND) 2018	240	7740	263	7740	27.8%	0.91 [0.77; 1.08]
Total (95% CI)		9994		10185	34.9%	0.92 [0.79; 1.07]
Heterogeneity: $Tau^2 = 0$; $Chi^2 = 0.08$, df = 2 (P = 0.96); $I^2 = 0\%$						
Total (95% CI)		66308		64920	100.0%	0.88 [0.80; 0.96]
**Prediction interval**						**[0.79; 0.98]**

Heterogeneity: $Tau^2 = 0$; $Chi^2 = 8.06$, df = 10 (P = 0.62); $I^2 = 0\%$
Residual heterogeneity: Tau^2 = NA; $Chi^2 = 7.58$, df = 9 (P = 0.58); $I^2 = 0\%$
Test for overall effect: Z = -2.79 (P < 0.01)

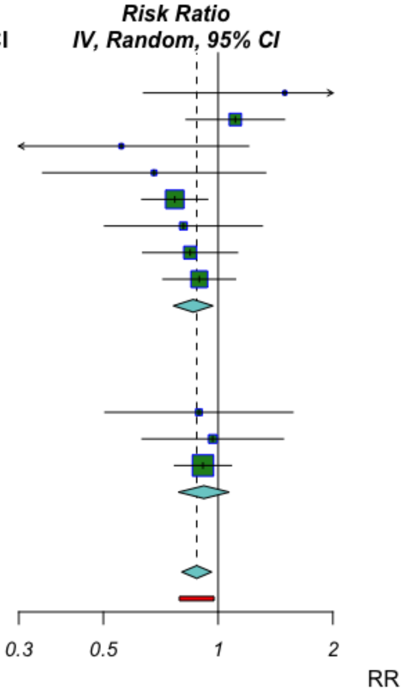

Figure G. Summary forest plot for sensitivity analysis on ischemic stroke by placebo use (excluding placebo use trials)

Study or Subgroup	experiment Events	experiment Total	control Events	control Total	Weight	Risk Ratio IV, Random, 95% CI
no						
Peto et al (BDS) 1988	21	3429	7	1710	1.1%	1.50 [0.64; 3.51]
de Gaetano et al (PPP) 2001	14	2226	21	2269	1.8%	0.68 [0.35; 1.33]
Ogawa et al (JPAD) 2008	22	1262	25	1277	2.6%	0.89 [0.50; 1.57]
Ikeda et al (JPPP) 2014	85	7220	101	7244	10.0%	0.84 [0.63; 1.12]
Satio et al (JPAD2) 2017	37	992	45	1168	4.5%	0.97 [0.63; 1.48]
Total (95% CI)		15129		13668	20.0%	0.89 [0.72; 1.09]
Heterogeneity: $Tau^2 = 0$; $Chi^2 = 2.32$, df = 4 (P = 0.68); $I^2 = 0\%$						
yes						
Steering et al (PHS) 1989	91	11037	82	11034	9.3%	1.11 [0.82; 1.49]
Meade et al (TPT) 1998	10	1268	18	1272	1.4%	0.56 [0.26; 1.20]
Ridker et al (WHS) 2005	170	19934	221	19942	20.8%	0.77 [0.63; 0.94]
Fowkes et al (AAA) 2010	30	1675	37	1675	3.6%	0.81 [0.50; 1.31]
Bowman et al (ASCEEND) 2018	240	7740	263	7740	27.8%	0.91 [0.77; 1.08]
McNeil et al (ASPREE) 2018	148	9525	167	9589	17.1%	0.89 [0.72; 1.11]
Total (95% CI)		51179		51252	80.0%	0.88 [0.78; 0.98]
Heterogeneity: $Tau^2 = 0.0026$; $Chi^2 = 5.73$, df = 5 (P = 0.33); $I^2 = 13\%$						
Total (95% CI)		66308		64920	100.0%	0.88 [0.80; 0.96]
**Prediction interval**						**[0.79; 0.98]**

Heterogeneity: $Tau^2 = 0$; $Chi^2 = 8.06$, df = 10 (P = 0.62); $I^2 = 0\%$
Residual heterogeneity: Tau^2 = NA; $Chi^2 = 8.05$, df = 9 (P = 0.53); $I^2 = 0\%$
Test for overall effect: Z = -2.79 (P < 0.01)

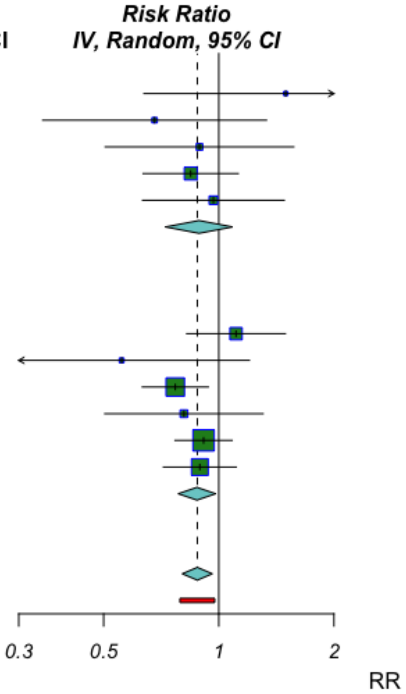

Figure H. Summary forest plot for sensitivity analysis on total stroke by excluding TPT trial

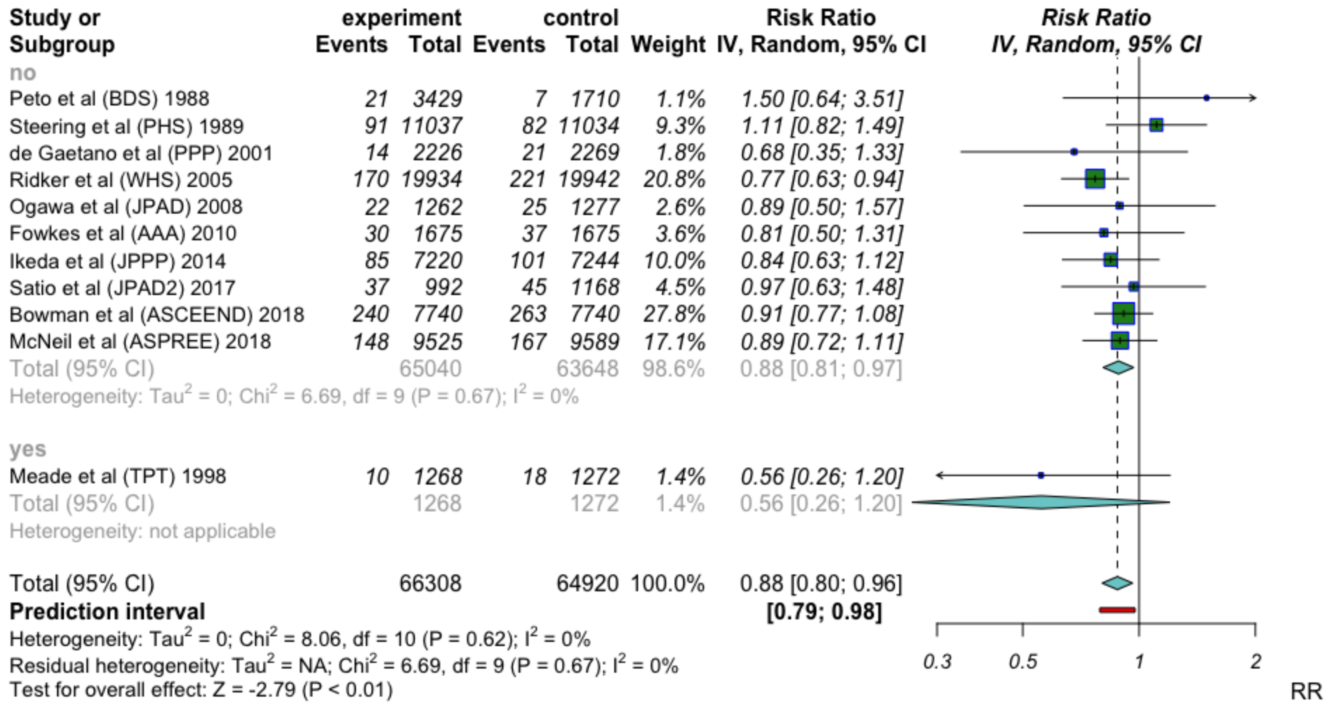

Supplement: Supplementary file 9 [file datasheet6.pdf]
