# Supplementary material for: Pros and Cons of Aspirin for the Primary Prevention of Cardiovascular Events: A Secondary Study of Trial Sequential Analysis
Source: Front Pharmacol. 2021 Jan 14;11:592116. doi: 10.3389/fphar.2020.592116 (PMC7845480; doi:10.3389/fphar.2020.592116)

Appendix File S8. Sensitivity analyses on cancer incidence.

Figure A. Summary forest plot for sensitivity analysis on incident cancer by year (excluding trials published before 2000)

Study or Subgroup	experiment Events	experiment Total	control Events	control Total	Weight	Risk Ratio IV, Random, 95% CI
**< 2000**						
Peto et al (BDS) 1988	*119*	*3429*	*58*	*1710*	*3.1%*	*1.02 [0.75; 1.39]*
Hansson et al (HOT) 1998	*294*	*9399*	*311*	*9391*	*9.5%*	*0.94 [0.81; 1.10]*
Total (95% CI)		12828		11101	12.6%	0.96 [0.83; 1.10]
Heterogeneity: Tau^2 = 0; Chi^2 = 0.21, df = 1 (P = 0.65); I^2 = 0%						
**>= 2000**						
de Gaetano et al (PPP) 2001	*86*	*2226*	*80*	*2269*	*3.3%*	*1.10 [0.81; 1.48]*
Ogawa et al (JPAD) 2008	*149*	*1262*	*169*	*1277*	*6.2%*	*0.89 [0.73; 1.10]*
Ikeda et al (JPPP) 2014	*332*	*7220*	*271*	*7244*	*9.4%*	*1.23 [1.05; 1.44]*
Ridker et al (WHS) 2005	*1438*	*19934*	*1427*	*19942*	*21.9%*	*1.01 [0.94; 1.08]*
Belch et al (POPADAD) 2008	*45*	*638*	*60*	*638*	*2.2%*	*0.75 [0.52; 1.09]*
Fowkes et al (AAA) 2010	*166*	*1675*	*194*	*1675*	*6.8%*	*0.86 [0.70; 1.04]*
Bowman et al (ASCEND) 2018	*897*	*7740*	*887*	*7740*	*18.6%*	*1.01 [0.93; 1.10]*
McNeil et al (ASPREE) 2018	*981*	*9525*	*952*	*9589*	*19.1%*	*1.04 [0.95; 1.13]*
Total (95% CI)		50220		50374	87.4%	1.01 [0.94; 1.08]
Heterogeneity: Tau^2 = 0.0037; Chi^2 = 13.24, df = 7 (P = 0.07); I^2 = 47%						
Total (95% CI)		63048		61475	100.0%	1.00 [0.95; 1.06]
**Prediction interval**						**[0.88; 1.15]**

Heterogeneity: Tau^2 = 0.0026; Chi^2 = 14.01, df = 9 (P = 0.12); I^2 = 36%
Residual heterogeneity: Tau^2 = NA; Chi^2 = 13.45, df = 8 (P = 0.10); I^2 = 41%
Test for overall effect: Z = 0.16 (P = 0.87)

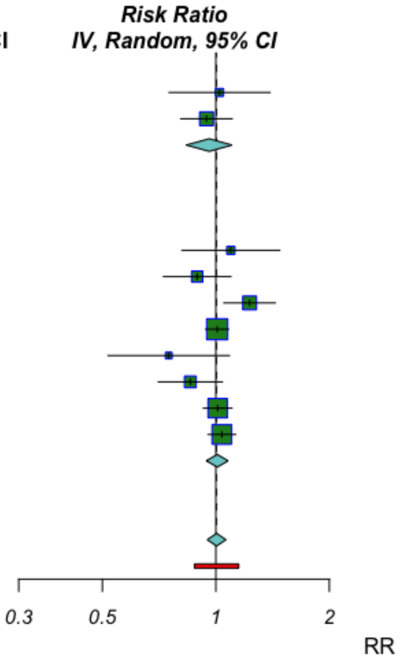

Figure B. Summary forest plot for sensitivity analysis on incident cancer by blinding (excluding open-label trials)

Study or Subgroup	experiment Events	experiment Total	control Events	control Total	Weight	Risk Ratio IV, Random, 95% CI
**double-blind**						
Hansson et al (HOT) 1998	*294*	*9399*	*311*	*9391*	*9.5%*	*0.94 [0.81; 1.10]*
Ridker et al (WHS) 2005	*1438*	*19934*	*1427*	*19942*	*21.9%*	*1.01 [0.94; 1.08]*
Belch et al (POPADAD) 2008	*45*	*638*	*60*	*638*	*2.2%*	*0.75 [0.52; 1.09]*
Fowkes et al (AAA) 2010	*166*	*1675*	*194*	*1675*	*6.8%*	*0.86 [0.70; 1.04]*
Bowman et al (ASCEND) 2018	*897*	*7740*	*887*	*7740*	*18.6%*	*1.01 [0.93; 1.10]*
McNeil et al (ASPREE) 2018	*981*	*9525*	*952*	*9589*	*19.1%*	*1.04 [0.95; 1.13]*
Total (95% CI)		48911		48975	77.9%	0.99 [0.95; 1.05]
Heterogeneity: Tau^2 = 0.0007; Chi^2 = 6.09, df = 5 (P = 0.30); I^2 = 18%						
**open-label**						
de Gaetano et al (PPP) 2001	*86*	*2226*	*80*	*2269*	*3.3%*	*1.10 [0.81; 1.48]*
Ogawa et al (JPAD) 2008	*149*	*1262*	*169*	*1277*	*6.2%*	*0.89 [0.73; 1.10]*
Ikeda et al (JPPP) 2014	*332*	*7220*	*271*	*7244*	*9.4%*	*1.23 [1.05; 1.44]*
Peto et al (BDS) 1988	*119*	*3429*	*58*	*1710*	*3.1%*	*1.02 [0.75; 1.39]*
Total (95% CI)		14137		12500	22.1%	1.06 [0.90; 1.25]
Heterogeneity: Tau^2 = 0.0137; Chi^2 = 6.03, df = 3 (P = 0.11); I^2 = 50%						
Total (95% CI)		63048		61475	100.0%	1.00 [0.95; 1.06]
**Prediction interval**						**[0.88; 1.15]**

Heterogeneity: Tau^2 = 0.0026; Chi^2 = 14.01, df = 9 (P = 0.12); I^2 = 36%
Residual heterogeneity: Tau^2 = NA; Chi^2 = 12.12, df = 8 (P = 0.15); I^2 = 34%
Test for overall effect: Z = 0.16 (P = 0.87)

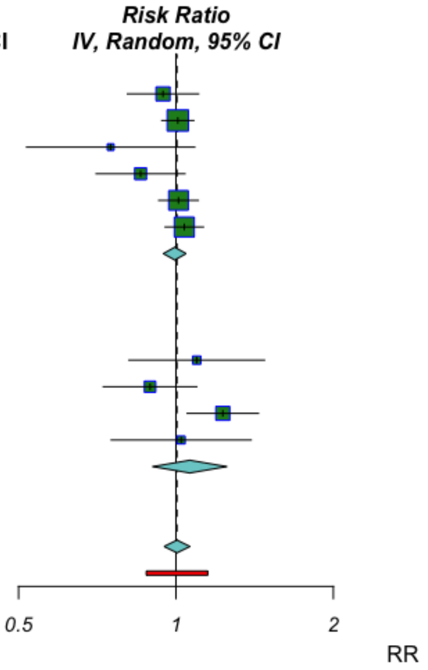

Figure C. Summary forest plot for sensitivity analysis on incident cancer by study quality (excluding high risk trials)

Study or Subgroup	experiment Events	experiment Total	control Events	control Total	Weight	Risk Ratio IV, Random, 95% CI
**High risk**						
de Gaetano et al (PPP) 2001	*86*	*2226*	*80*	*2269*	*3.3%*	*1.10 [0.81; 1.48]*
Ogawa et al (JPAD) 2008	*149*	*1262*	*169*	*1277*	*6.2%*	*0.89 [0.73; 1.10]*
Ikeda et al (JPPP) 2014	*332*	*7220*	*271*	*7244*	*9.4%*	*1.23 [1.05; 1.44]*
Peto et al (BDS) 1988	*119*	*3429*	*58*	*1710*	*3.1%*	*1.02 [0.75; 1.39]*
Total (95% CI)		14137		12500	22.1%	1.06 [0.90; 1.25]
Heterogeneity: $Tau^2 = 0.0137$; $Chi^2 = 6.03$, df = 3 (P = 0.11); $I^2 = 50\%$						
**Low risk**						
Hansson et al (HOT) 1998	*294*	*9399*	*311*	*9391*	*9.5%*	*0.94 [0.81; 1.10]*
Ridker et al (WHS) 2005	*1438*	*19934*	*1427*	*19942*	*21.9%*	*1.01 [0.94; 1.08]*
Belch et al (POPADAD) 2008	*45*	*638*	*60*	*638*	*2.2%*	*0.75 [0.52; 1.09]*
Fowkes et al (AAA) 2010	*166*	*1675*	*194*	*1675*	*6.8%*	*0.86 [0.70; 1.04]*
Bowman et al (ASCEND) 2018	*897*	*7740*	*887*	*7740*	*18.6%*	*1.01 [0.93; 1.10]*
McNeil et al (ASPREE) 2018	*981*	*9525*	*952*	*9589*	*19.1%*	*1.04 [0.95; 1.13]*
Total (95% CI)		48911		48975	77.9%	0.99 [0.95; 1.05]
Heterogeneity: $Tau^2 = 0.0007$; $Chi^2 = 6.09$, df = 5 (P = 0.30); $I^2 = 18\%$						
Total (95% CI)		63048		61475	100.0%	1.00 [0.95; 1.06]
**Prediction interval**						**[0.88; 1.15]**

Heterogeneity: $Tau^2 = 0.0026$; $Chi^2 = 14.01$, df = 9 (P = 0.12); $I^2 = 36\%$
Residual heterogeneity: Tau^2 = NA; $Chi^2 = 12.12$, df = 8 (P = 0.15); $I^2 = 34\%$
Test for overall effect: Z = 0.16 (P = 0.87)

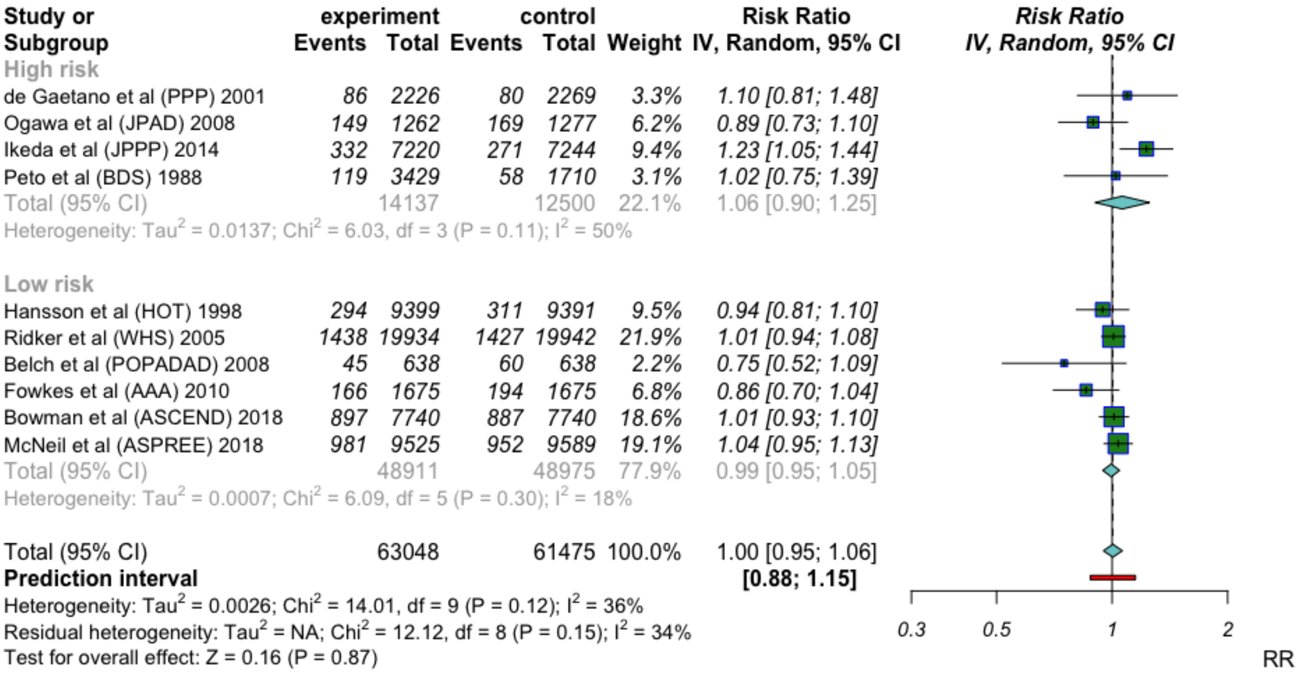

Figure D. Summary forest plot for sensitivity analysis on incident cancer by asymptomatic PAD (excluding asymptomatic PAD trials)

Study or Subgroup	experiment Events	experiment Total	control Events	control Total	Weight	Risk Ratio IV, Random, 95% CI
no						
de Gaetano et al (PPP) 2001	*86*	*2226*	*80*	*2269*	*3.3%*	*1.10 [0.81; 1.48]*
Ogawa et al (JPAD) 2008	*149*	*1262*	*169*	*1277*	*6.2%*	*0.89 [0.73; 1.10]*
Ikeda et al (JPPP) 2014	*332*	*7220*	*271*	*7244*	*9.4%*	*1.23 [1.05; 1.44]*
Peto et al (BDS) 1988	*119*	*3429*	*58*	*1710*	*3.1%*	*1.02 [0.75; 1.39]*
Hansson et al (HOT) 1998	*294*	*9399*	*311*	*9391*	*9.5%*	*0.94 [0.81; 1.10]*
Ridker et al (WHS) 2005	*1438*	*19934*	*1427*	*19942*	*21.9%*	*1.01 [0.94; 1.08]*
Bowman et al (ASCEND) 2018	*897*	*7740*	*887*	*7740*	*18.6%*	*1.01 [0.93; 1.10]*
McNeil et al (ASPREE) 2018	*981*	*9525*	*952*	*9589*	*19.1%*	*1.04 [0.95; 1.13]*
Total (95% CI)		60735		59162	91.0%	1.02 [0.97; 1.07]
Heterogeneity: $Tau^2 = 0.0008$; $Chi^2 = 8.47$, df = 7 (P = 0.29); $I^2 = 17\%$						
yes						
Belch et al (POPADAD) 2008	*45*	*638*	*60*	*638*	*2.2%*	*0.75 [0.52; 1.09]*
Fowkes et al (AAA) 2010	*166*	*1675*	*194*	*1675*	*6.8%*	*0.86 [0.70; 1.04]*
Total (95% CI)		2313		2313	9.0%	0.83 [0.70; 0.99]
Heterogeneity: $Tau^2 = 0$; $Chi^2 = 0.38$, df = 1 (P = 0.54); $I^2 = 0\%$						
Total (95% CI)		63048		61475	100.0%	1.00 [0.95; 1.06]
**Prediction interval**						**[0.88; 1.15]**

Heterogeneity: $Tau^2 = 0.0026$; $Chi^2 = 14.01$, df = 9 (P = 0.12); $I^2 = 36\%$
Residual heterogeneity: Tau^2 = NA; $Chi^2 = 8.85$, df = 8 (P = 0.35); $I^2 = 10\%$
Test for overall effect: Z = 0.16 (P = 0.87)

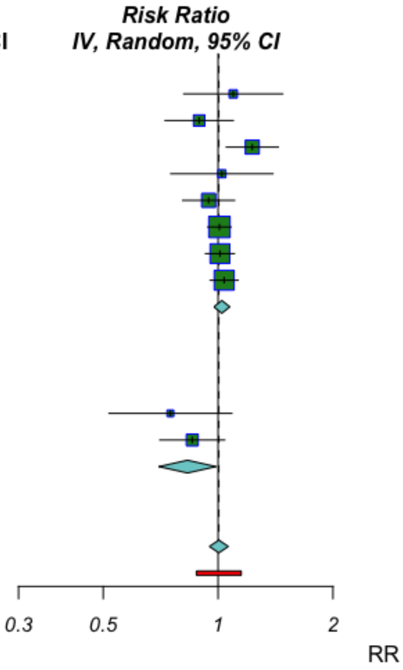

Figure E. Summary forest plot for sensitivity analysis on incident cancer by male populations (excluding 100% male individual trials)

Study or Subgroup	experiment Events	experiment Total	control Events	control Total	Weight	Risk Ratio IV, Random, 95% CI
**male**						
Peto et al (BDS) 1988	*119*	*3429*	*58*	*1710*	*3.1%*	*1.02 [0.75; 1.39]*
Total (95% CI)		3429		1710	3.1%	1.02 [0.75; 1.39]
Heterogeneity: not applicable						
**not all male**						
de Gaetano et al (PPP) 2001	*86*	*2226*	*80*	*2269*	*3.3%*	*1.10 [0.81; 1.48]*
Ogawa et al (JPAD) 2008	*149*	*1262*	*169*	*1277*	*6.2%*	*0.89 [0.73; 1.10]*
Ikeda et al (JPPP) 2014	*332*	*7220*	*271*	*7244*	*9.4%*	*1.23 [1.05; 1.44]*
Hansson et al (HOT) 1998	*294*	*9399*	*311*	*9391*	*9.5%*	*0.94 [0.81; 1.10]*
Ridker et al (WHS) 2005	*1438*	*19934*	*1427*	*19942*	*21.9%*	*1.01 [0.94; 1.08]*
Belch et al (POPADAD) 2008	*45*	*638*	*60*	*638*	*2.2%*	*0.75 [0.52; 1.09]*
Fowkes et al (AAA) 2010	*166*	*1675*	*194*	*1675*	*6.8%*	*0.86 [0.70; 1.04]*
Bowman et al (ASCEND) 2018	*897*	*7740*	*887*	*7740*	*18.6%*	*1.01 [0.93; 1.10]*
McNeil et al (ASPREE) 2018	*981*	*9525*	*952*	*9589*	*19.1%*	*1.04 [0.95; 1.13]*
Total (95% CI)		59619		59765	96.9%	1.00 [0.94; 1.07]
Heterogeneity: $Tau^2 = 0.0032$; $Chi^2 = 14$, df = 8 (P = 0.08); $I^2 = 43\%$						
Total (95% CI)		63048		61475	100.0%	1.00 [0.95; 1.06]
**Prediction interval**						**[0.88; 1.15]**

Heterogeneity: $Tau^2 = 0.0026$; $Chi^2 = 14.01$, df = 9 (P = 0.12); $I^2 = 36\%$
Residual heterogeneity: $Tau^2 = NA$; $Chi^2 = 14.00$, df = 8 (P = 0.08); $I^2 = 43\%$
Test for overall effect: Z = 0.16 (P = 0.87)

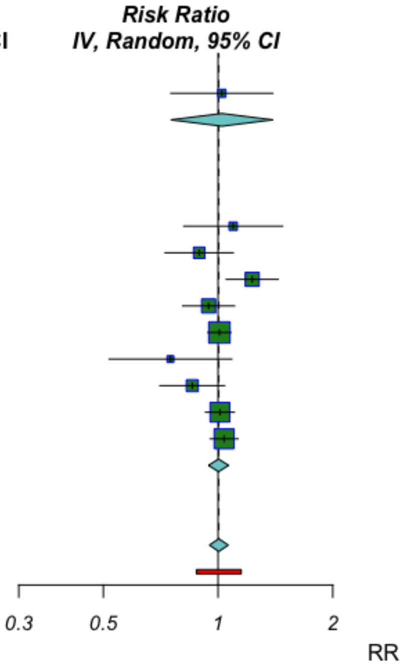

Figure F. Summary forest plot for sensitivity analysis on incident cancer by diabetes status (excluding 100% diabetic individual trials and restricted on 100% diabetic individual trials)

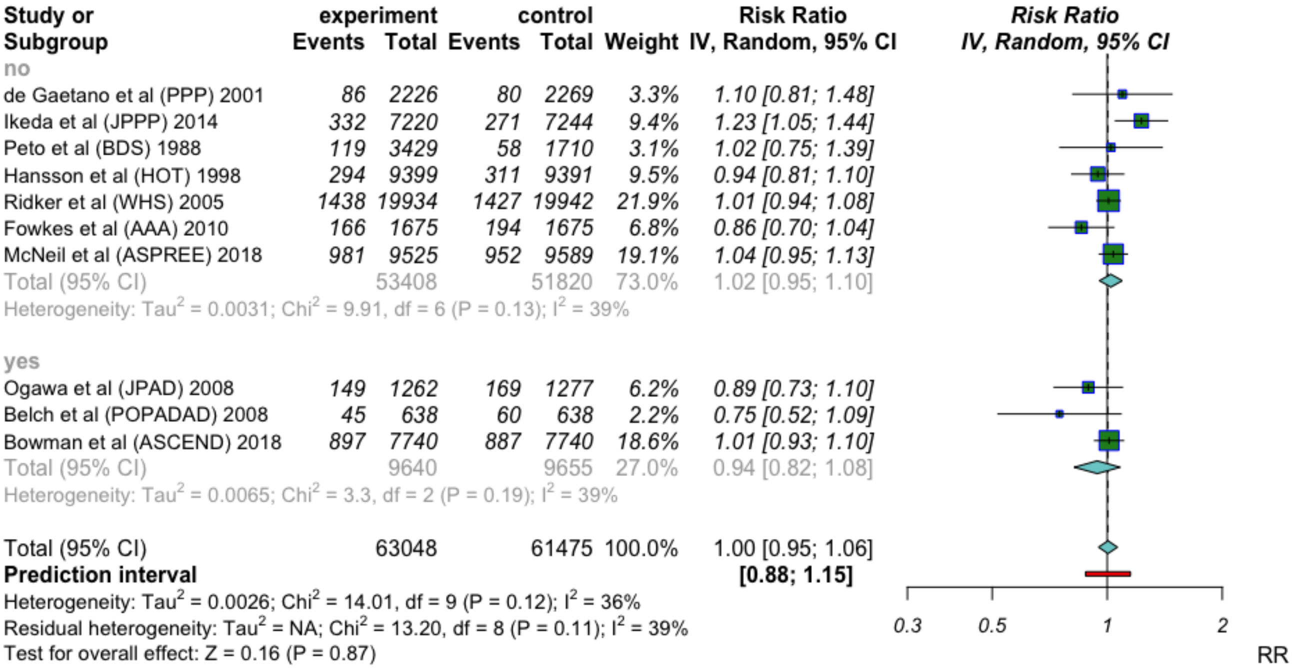

Figure G. Summary forest plot for sensitivity analysis on incident cancer by placebo use (excluding placebo use trials)

Study or Subgroup	experiment Events	experiment Total	control Events	control Total	Weight	Risk Ratio IV, Random, 95% CI
no						
de Gaetano et al (PPP) 2001	*86*	*2226*	*80*	*2269*	*3.3%*	*1.10 [0.81; 1.48]*
Ogawa et al (JPAD) 2008	*149*	*1262*	*169*	*1277*	*6.2%*	*0.89 [0.73; 1.10]*
Ikeda et al (JPPP) 2014	*332*	*7220*	*271*	*7244*	*9.4%*	*1.23 [1.05; 1.44]*
Peto et al (BDS) 1988	*119*	*3429*	*58*	*1710*	*3.1%*	*1.02 [0.75; 1.39]*
Total (95% CI)		14137		12500	22.1%	1.06 [0.90; 1.25]
Heterogeneity: $Tau^2 = 0.0137$; $Chi^2 = 6.03$, df = 3 (P = 0.11); $I^2 = 50\%$						
yes						
Hansson et al (HOT) 1998	*294*	*9399*	*311*	*9391*	*9.5%*	*0.94 [0.81; 1.10]*
Ridker et al (WHS) 2005	*1438*	*19934*	*1427*	*19942*	*21.9%*	*1.01 [0.94; 1.08]*
Belch et al (POPADAD) 2008	*45*	*638*	*60*	*638*	*2.2%*	*0.75 [0.52; 1.09]*
Fowkes et al (AAA) 2010	*166*	*1675*	*194*	*1675*	*6.8%*	*0.86 [0.70; 1.04]*
Bowman et al (ASCEND) 2018	*897*	*7740*	*887*	*7740*	*18.6%*	*1.01 [0.93; 1.10]*
McNeil et al (ASPREE) 2018	*981*	*9525*	*952*	*9589*	*19.1%*	*1.04 [0.95; 1.13]*
Total (95% CI)		48911		48975	77.9%	0.99 [0.95; 1.05]
Heterogeneity: $Tau^2 = 0.0007$; $Chi^2 = 6.09$, df = 5 (P = 0.30); $I^2 = 18\%$						
Total (95% CI)		63048		61475	100.0%	1.00 [0.95; 1.06]
**Prediction interval**						**[0.88; 1.15]**

Heterogeneity: $Tau^2 = 0.0026$; $Chi^2 = 14.01$, df = 9 (P = 0.12); $I^2 = 36\%$
Residual heterogeneity: $Tau^2 = NA$; $Chi^2 = 12.12$, df = 8 (P = 0.15); $I^2 = 34\%$
Test for overall effect: Z = 0.16 (P = 0.87)

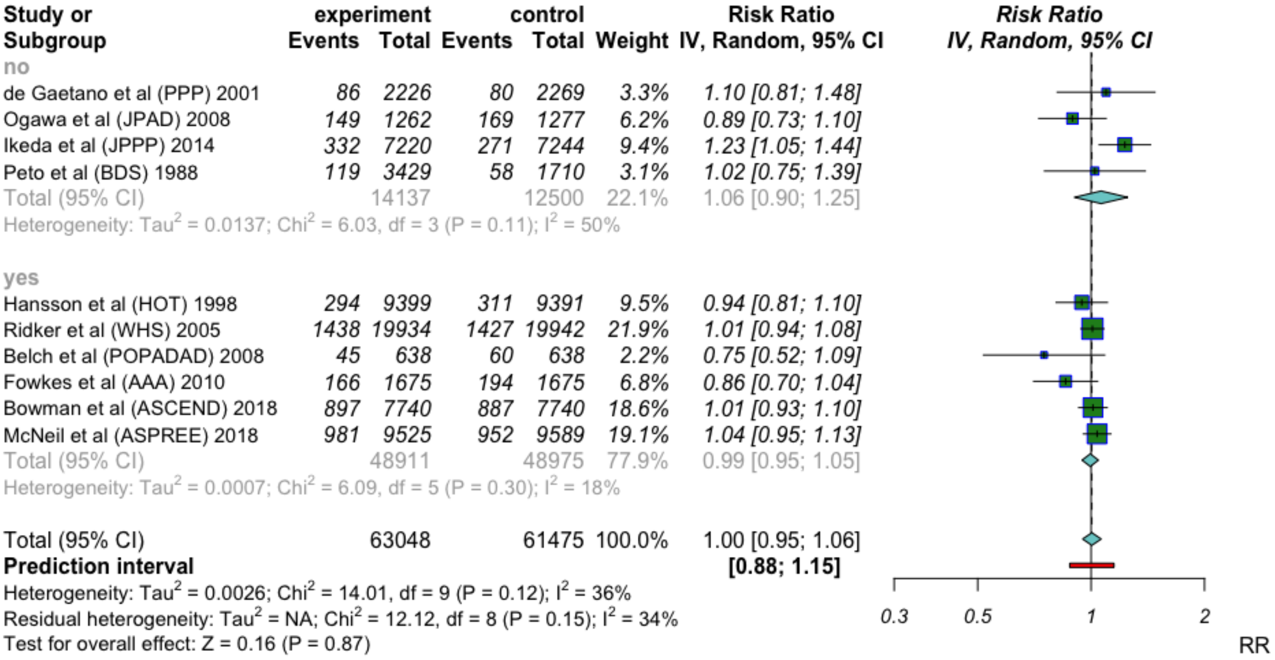

Supplement: Supplementary file 10 [file datasheet7.pdf]
