# Supplementary material for: Pros and Cons of Aspirin for the Primary Prevention of Cardiovascular Events: A Secondary Study of Trial Sequential Analysis
Source: Front Pharmacol. 2021 Jan 14;11:592116. doi: 10.3389/fphar.2020.592116 (PMC7845480; doi:10.3389/fphar.2020.592116)

Appendix File S9. Sensitivity analyses on cancer mortality.

Figure A. Summary forest plot for sensitivity analysis on incident cancer by year (excluding trials published before 2000)

Study or Subgroup	experiment Events	experiment Total	control Events	control Total	Weight	Risk Ratio IV, Random, 95% CI
**< 2000**						
Peto et al (BDS) 1988	*75*	*3429*	*47*	*1710*	*4.9%*	*0.80 [0.56; 1.14]*
Hansson et al (HOT) 1998	*108*	*9399*	*105*	*9391*	*8.1%*	*1.03 [0.79; 1.34]*
Steering et al (PHS) 1989	*79*	*11037*	*68*	*11034*	*5.9%*	*1.16 [0.84; 1.60]*
Meade et al (TPT) 1998	*49*	*1268*	*51*	*1272*	*4.4%*	*0.96 [0.66; 1.42]*
Total (95% CI)		25133		23407	23.3%	0.99 [0.85; 1.17]
Heterogeneity: $Tau^2 = 0$; $Chi^2 = 2.44$, df = 3 (P = 0.49); $I^2 = 0\%$						
**>= 2000**						
de Gaetano et al (PPP) 2001	*31*	*2226*	*29*	*2269*	*2.7%*	*1.09 [0.66; 1.80]*
Ogawa et al (JPAD) 2008	*63*	*1262*	*60*	*1277*	*5.3%*	*1.06 [0.75; 1.50]*
Ikeda et al (JPPP) 2014	*134*	*7220*	*125*	*7244*	*9.5%*	*1.08 [0.84; 1.37]*
Ridker et al (WHS) 2005	*284*	*19934*	*299*	*19942*	*16.7%*	*0.95 [0.81; 1.12]*
Belch et al (POPADAD) 2008	*25*	*638*	*31*	*638*	*2.5%*	*0.81 [0.48; 1.35]*
Fowkes et al (AAA) 2010	*78*	*1675*	*90*	*1675*	*6.9%*	*0.87 [0.64; 1.16]*
Bowman et al (ASCEND) 2018	*309*	*7740*	*315*	*7740*	*17.7%*	*0.98 [0.84; 1.14]*
McNeil et al (ASPREE) 2018	*295*	*9525*	*227*	*9589*	*15.5%*	*1.31 [1.10; 1.55]*
Total (95% CI)		50220		50374	76.7%	1.03 [0.93; 1.15]
Heterogeneity: $Tau^2 = 0.0085$; $Chi^2 = 11.22$, df = 7 (P = 0.13); $I^2 = 38\%$						
Total (95% CI)		75353		73781	100.0%	1.03 [0.94; 1.12]
**Prediction interval**						**[0.86; 1.22]**
Heterogeneity: $Tau^2 = 0.0044$; $Chi^2 = 13.89$, df = 11 (P = 0.24); $I^2 = 21\%$						
Residual heterogeneity: $Tau^2 = NA$; $Chi^2 = 13.67$, df = 10 (P = 0.19); $I^2 = 27\%$						
Test for overall effect: Z = 0.59 (P = 0.56)						

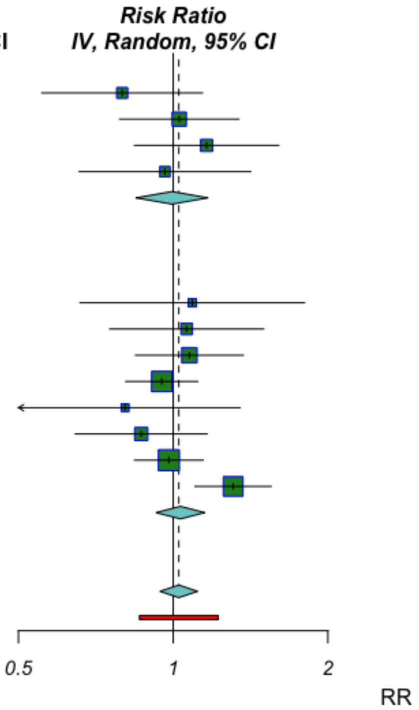

Figure B. Summary forest plot for sensitivity analysis on incident cancer by blinding (excluding open-label trials)

Study or Subgroup	experiment Events	experiment Total	control Events	control Total	Weight	Risk Ratio IV, Random, 95% CI
double-blind						
Hansson et al (HOT) 1998	*108*	*9399*	*105*	*9391*	*8.1%*	*1.03 [0.79; 1.34]*
Ridker et al (WHS) 2005	*284*	*19934*	*299*	*19942*	*16.7%*	*0.95 [0.81; 1.12]*
Belch et al (POPADAD) 2008	*25*	*638*	*31*	*638*	*2.5%*	*0.81 [0.48; 1.35]*
Fowkes et al (AAA) 2010	*78*	*1675*	*90*	*1675*	*6.9%*	*0.87 [0.64; 1.16]*
Bowman et al (ASCEND) 2018	*309*	*7740*	*315*	*7740*	*17.7%*	*0.98 [0.84; 1.14]*
McNeil et al (ASPREE) 2018	*295*	*9525*	*227*	*9589*	*15.5%*	*1.31 [1.10; 1.55]*
Steering et al (PHS) 1989	*79*	*11037*	*68*	*11034*	*5.9%*	*1.16 [0.84; 1.60]*
Meade et al (TPT) 1998	*49*	*1268*	*51*	*1272*	*4.4%*	*0.96 [0.66; 1.42]*
Total (95% CI)		61216		61281	77.7%	1.03 [0.92; 1.15]
Heterogeneity: $Tau^2 = 0.0094$; $Chi^2 = 11.7$, df = 7 (P = 0.11); $I^2 = 40\%$						
open-label						
de Gaetano et al (PPP) 2001	*31*	*2226*	*29*	*2269*	*2.7%*	*1.09 [0.66; 1.80]*
Ogawa et al (JPAD) 2008	*63*	*1262*	*60*	*1277*	*5.3%*	*1.06 [0.75; 1.50]*
Ikeda et al (JPPP) 2014	*134*	*7220*	*125*	*7244*	*9.5%*	*1.08 [0.84; 1.37]*
Peto et al (BDS) 1988	*75*	*3429*	*47*	*1710*	*4.9%*	*0.80 [0.56; 1.14]*
Total (95% CI)		14137		12500	22.3%	1.01 [0.86; 1.19]
Heterogeneity: $Tau^2 = 0$; $Chi^2 = 2.12$, df = 3 (P = 0.55); $I^2 = 0\%$						
Total (95% CI)		75353		73781	100.0%	1.03 [0.94; 1.12]
**Prediction interval**						**[0.86; 1.22]**

Heterogeneity: $Tau^2 = 0.0044$; $Chi^2 = 13.89$, df = 11 (P = 0.24); $I^2 = 21\%$
Residual heterogeneity: $Tau^2 = NA$; $Chi^2 = 13.81$, df = 10 (P = 0.18); $I^2 = 28\%$
Test for overall effect: Z = 0.59 (P = 0.56)

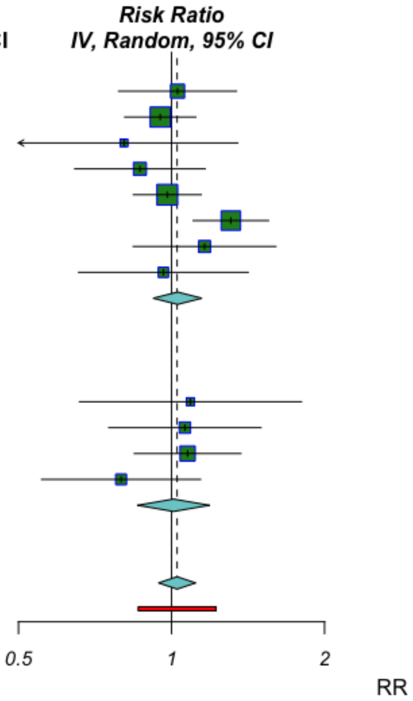

Figure C. Summary forest plot for sensitivity analysis on incident cancer by study quality (excluding high risk trials)

Study or Subgroup	experiment Events	experiment Total	control Events	control Total	Weight	Risk Ratio IV, Random, 95% CI
**High risk**						
de Gaetano et al (PPP) 2001	*31*	*2226*	*29*	*2269*	*2.7%*	*1.09 [0.66; 1.80]*
Ogawa et al (JPAD) 2008	*63*	*1262*	*60*	*1277*	*5.3%*	*1.06 [0.75; 1.50]*
Ikeda et al (JPPP) 2014	*134*	*7220*	*125*	*7244*	*9.5%*	*1.08 [0.84; 1.37]*
Peto et al (BDS) 1988	*75*	*3429*	*47*	*1710*	*4.9%*	*0.80 [0.56; 1.14]*
Steering et al (PHS) 1989	*79*	*11037*	*68*	*11034*	*5.9%*	*1.16 [0.84; 1.60]*
Meade et al (TPT) 1998	*49*	*1268*	*51*	*1272*	*4.4%*	*0.96 [0.66; 1.42]*
Total (95% CI)		26442		24806	32.6%	1.03 [0.90; 1.18]
Heterogeneity: $Tau^2 = 0$; $Chi^2 = 2.82$, df = 5 (P = 0.73); $I^2 = 0\%$						
**Low risk**						
Hansson et al (HOT) 1998	*108*	*9399*	*105*	*9391*	*8.1%*	*1.03 [0.79; 1.34]*
Ridker et al (WHS) 2005	*284*	*19934*	*299*	*19942*	*16.7%*	*0.95 [0.81; 1.12]*
Belch et al (POPADAD) 2008	*25*	*638*	*31*	*638*	*2.5%*	*0.81 [0.48; 1.35]*
Fowkes et al (AAA) 2010	*78*	*1675*	*90*	*1675*	*6.9%*	*0.87 [0.64; 1.16]*
Bowman et al (ASCEND) 2018	*309*	*7740*	*315*	*7740*	*17.7%*	*0.98 [0.84; 1.14]*
McNeil et al (ASPREE) 2018	*295*	*9525*	*227*	*9589*	*15.5%*	*1.31 [1.10; 1.55]*
Total (95% CI)		48911		48975	67.4%	1.02 [0.89; 1.16]
Heterogeneity: $Tau^2 = 0.0142$; $Chi^2 = 11.07$, df = 5 (P = 0.05); $I^2 = 55\%$						
Total (95% CI)		75353		73781	100.0%	1.03 [0.94; 1.12]
**Prediction interval**						**[0.86; 1.22]**

Heterogeneity: $Tau^2 = 0.0044$; $Chi^2 = 13.89$, df = 11 (P = 0.24); $I^2 = 21\%$
Residual heterogeneity: Tau^2 = NA; $Chi^2 = 13.89$, df = 10 (P = 0.18); $I^2 = 28\%$
Test for overall effect: Z = 0.59 (P = 0.56)

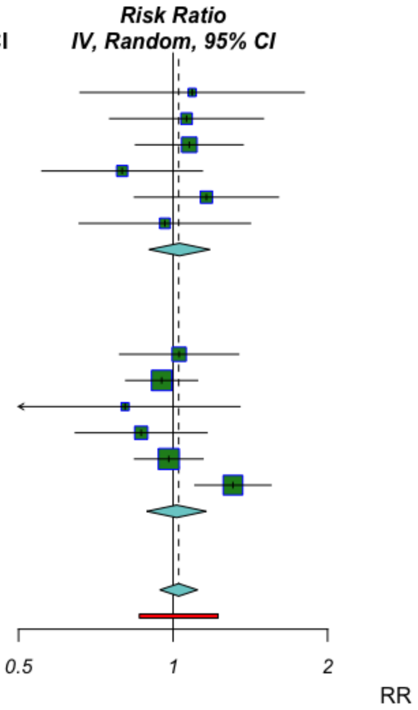

Figure D. Summary forest plot for sensitivity analysis on incident cancer by asymptomatic PAD (excluding asymptomatic PAD trials)

Study or Subgroup	experiment Events	experiment Total	control Events	control Total	Weight	Risk Ratio IV, Random, 95% CI
no						
de Gaetano et al (PPP) 2001	31	2226	29	2269	2.7%	1.09 [0.66; 1.80]
Ogawa et al (JPAD) 2008	63	1262	60	1277	5.3%	1.06 [0.75; 1.50]
Ikeda et al (JPPP) 2014	134	7220	125	7244	9.5%	1.08 [0.84; 1.37]
Peto et al (BDS) 1988	75	3429	47	1710	4.9%	0.80 [0.56; 1.14]
Hansson et al (HOT) 1998	108	9399	105	9391	8.1%	1.03 [0.79; 1.34]
Ridker et al (WHS) 2005	284	19934	299	19942	16.7%	0.95 [0.81; 1.12]
Bowman et al (ASCEND) 2018	309	7740	315	7740	17.7%	0.98 [0.84; 1.14]
McNeil et al (ASPREE) 2018	295	9525	227	9589	15.5%	1.31 [1.10; 1.55]
Steering et al (PHS) 1989	79	11037	68	11034	5.9%	1.16 [0.84; 1.60]
Meade et al (TPT) 1998	49	1268	51	1272	4.4%	0.96 [0.66; 1.42]
Total (95% CI)		73040		71468	90.6%	1.05 [0.96; 1.14]
Heterogeneity: $Tau^2 = 0.0043$; $Chi^2 = 11.53$, df = 9 (P = 0.24); $I^2 = 22\%$						
yes						
Belch et al (POPADAD) 2008	25	638	31	638	2.5%	0.81 [0.48; 1.35]
Fowkes et al (AAA) 2010	78	1675	90	1675	6.9%	0.87 [0.64; 1.16]
Total (95% CI)		2313		2313	9.4%	0.85 [0.66; 1.10]
Heterogeneity: $Tau^2 = 0$; $Chi^2 = 0.06$, df = 1 (P = 0.81); $I^2 = 0\%$						
**Total (95% CI)**		75353		73781	100.0%	1.03 [0.94; 1.12]
**Prediction interval**						**[0.86; 1.22]**

Heterogeneity: $Tau^2 = 0.0044$; $Chi^2 = 13.89$, df = 11 (P = 0.24); $I^2 = 21\%$
Residual heterogeneity: $Tau^2 = NA$; $Chi^2 = 11.58$, df = 10 (P = 0.31); $I^2 = 14\%$
Test for overall effect: Z = 0.59 (P = 0.56)

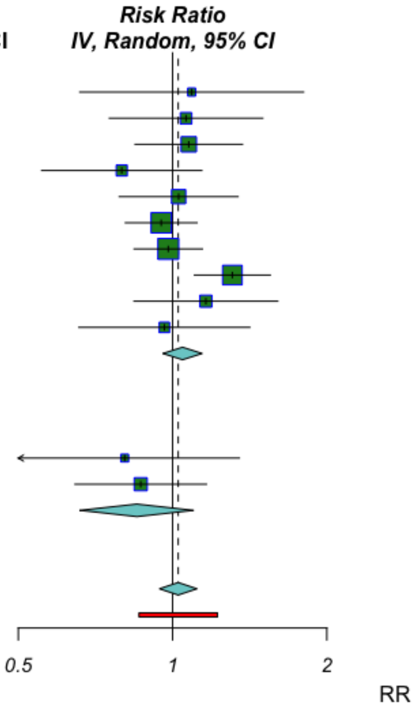

Figure E. Summary forest plot for sensitivity analysis on incident cancer by male populations (excluding 100% male individual trials)

Study or Subgroup	experiment Events	experiment Total	control Events	control Total	Weight	Risk Ratio IV, Random, 95% CI
male						
Peto et al (BDS) 1988	75	3429	47	1710	4.9%	0.80 [0.56; 1.14]
Steering et al (PHS) 1989	79	11037	68	11034	5.9%	1.16 [0.84; 1.60]
Meade et al (TPT) 1998	49	1268	51	1272	4.4%	0.96 [0.66; 1.42]
Total (95% CI)		15734		14016	15.1%	0.97 [0.78; 1.22]
Heterogeneity: $Tau^2 = 0.0058$; $Chi^2 = 2.35$, df = 2 (P = 0.31); $I^2 = 15\%$						
not all male						
de Gaetano et al (PPP) 2001	31	2226	29	2269	2.7%	1.09 [0.66; 1.80]
Ogawa et al (JPAD) 2008	63	1262	60	1277	5.3%	1.06 [0.75; 1.50]
Ikeda et al (JPPP) 2014	134	7220	125	7244	9.5%	1.08 [0.84; 1.37]
Hansson et al (HOT) 1998	108	9399	105	9391	8.1%	1.03 [0.79; 1.34]
Ridker et al (WHS) 2005	284	19934	299	19942	16.7%	0.95 [0.81; 1.12]
Belch et al (POPADAD) 2008	25	638	31	638	2.5%	0.81 [0.48; 1.35]
Fowkes et al (AAA) 2010	78	1675	90	1675	6.9%	0.87 [0.64; 1.16]
Bowman et al (ASCEND) 2018	309	7740	315	7740	17.7%	0.98 [0.84; 1.14]
McNeil et al (ASPREE) 2018	295	9525	227	9589	15.5%	1.31 [1.10; 1.55]
Total (95% CI)		59619		59765	84.9%	1.03 [0.94; 1.14]
Heterogeneity: $Tau^2 = 0.0058$; $Chi^2 = 11.23$, df = 8 (P = 0.19); $I^2 = 29\%$						
Total (95% CI)		75353		73781	100.0%	1.03 [0.94; 1.12]
**Prediction interval**						**[0.86; 1.22]**

Heterogeneity: $Tau^2 = 0.0044$; $Chi^2 = 13.89$, df = 11 (P = 0.24); $I^2 = 21\%$
Residual heterogeneity: Tau^2 = NA; $Chi^2 = 13.58$, df = 10 (P = 0.19); $I^2 = 26\%$
Test for overall effect: Z = 0.59 (P = 0.56)

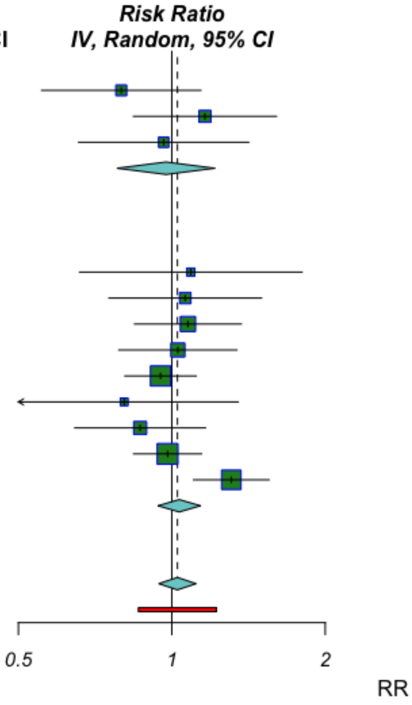

Figure F. Summary forest plot for sensitivity analysis on incident cancer by diabetes status (excluding 100% diabetic individual trials and restricted on 100% diabetic individual trials)

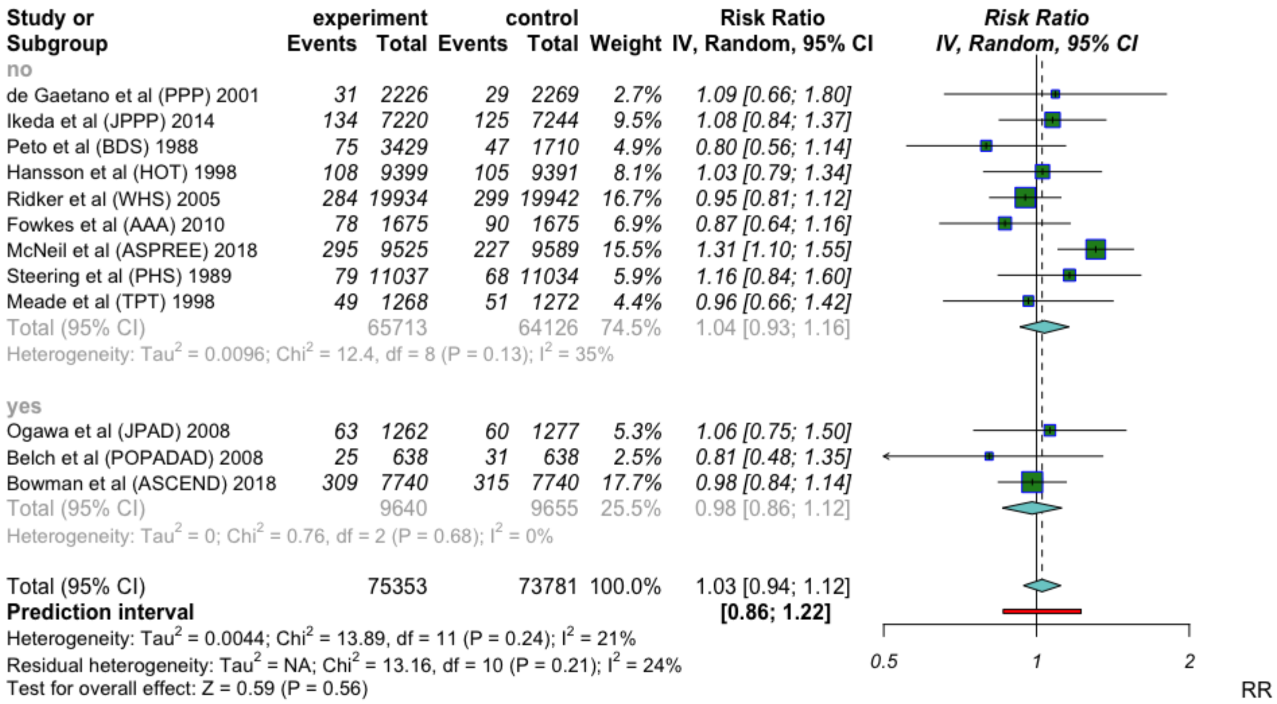

Figure G. Summary forest plot for sensitivity analysis on incident cancer by placebo use (excluding placebo use trials)

Study or Subgroup	experiment Events	experiment Total	control Events	control Total	Weight	Risk Ratio IV, Random, 95% CI
no						
de Gaetano et al (PPP) 2001	31	2226	29	2269	2.7%	1.09 [0.66; 1.80]
Ogawa et al (JPAD) 2008	63	1262	60	1277	5.3%	1.06 [0.75; 1.50]
Ikeda et al (JPPP) 2014	134	7220	125	7244	9.5%	1.08 [0.84; 1.37]
Peto et al (BDS) 1988	75	3429	47	1710	4.9%	0.80 [0.56; 1.14]
Total (95% CI)		14137		12500	22.3%	1.01 [0.86; 1.19]
Heterogeneity: $Tau^2 = 0$; $Chi^2 = 2.12$, df = 3 (P = 0.55); $I^2 = 0\%$						
yes						
Hansson et al (HOT) 1998	108	9399	105	9391	8.1%	1.03 [0.79; 1.34]
Ridker et al (WHS) 2005	284	19934	299	19942	16.7%	0.95 [0.81; 1.12]
Belch et al (POPADAD) 2008	25	638	31	638	2.5%	0.81 [0.48; 1.35]
Fowkes et al (AAA) 2010	78	1675	90	1675	6.9%	0.87 [0.64; 1.16]
Bowman et al (ASCEND) 2018	309	7740	315	7740	17.7%	0.98 [0.84; 1.14]
McNeil et al (ASPREE) 2018	295	9525	227	9589	15.5%	1.31 [1.10; 1.55]
Steering et al (PHS) 1989	79	11037	68	11034	5.9%	1.16 [0.84; 1.60]
Meade et al (TPT) 1998	49	1268	51	1272	4.4%	0.96 [0.66; 1.42]
Total (95% CI)		61216		61281	77.7%	1.03 [0.92; 1.15]
Heterogeneity: $Tau^2 = 0.0094$; $Chi^2 = 11.7$, df = 7 (P = 0.11); $I^2 = 40\%$						
Total (95% CI)		75353		73781	100.0%	1.03 [0.94; 1.12]
**Prediction interval**						**[0.86; 1.22]**

Heterogeneity: $Tau^2 = 0.0044$; $Chi^2 = 13.89$, df = 11 (P = 0.24); $I^2 = 21\%$
Residual heterogeneity: Tau^2 = NA; $Chi^2 = 13.81$, df = 10 (P = 0.18); $I^2 = 28\%$
Test for overall effect: Z = 0.59 (P = 0.56)

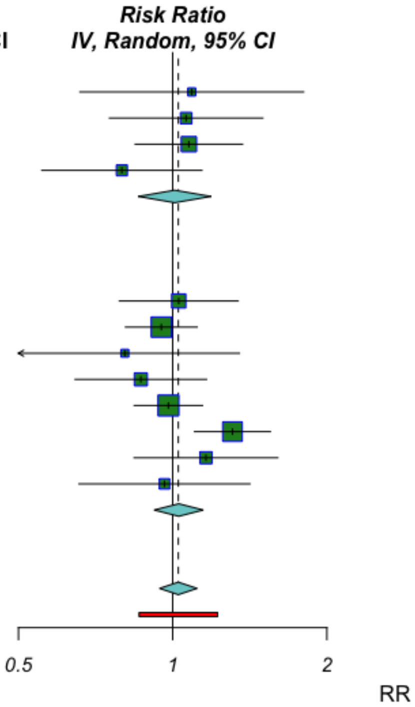

Figure H. Summary forest plot for sensitivity analysis on excluding TPT trial

Study or Subgroup	experiment Events	experiment Total	control Events	control Total	Weight	Risk Ratio IV, Random, 95% CI
no						
de Gaetano et al (PPP) 2001	*31*	*2226*	*29*	*2269*	*2.7%*	*1.09 [0.66; 1.80]*
Ogawa et al (JPAD) 2008	*63*	*1262*	*60*	*1277*	*5.3%*	*1.06 [0.75; 1.50]*
Ikeda et al (JPPP) 2014	*134*	*7220*	*125*	*7244*	*9.5%*	*1.08 [0.84; 1.37]*
Peto et al (BDS) 1988	*75*	*3429*	*47*	*1710*	*4.9%*	*0.80 [0.56; 1.14]*
Hansson et al (HOT) 1998	*108*	*9399*	*105*	*9391*	*8.1%*	*1.03 [0.79; 1.34]*
Ridker et al (WHS) 2005	*284*	*19934*	*299*	*19942*	*16.7%*	*0.95 [0.81; 1.12]*
Belch et al (POPADAD) 2008	*25*	*638*	*31*	*638*	*2.5%*	*0.81 [0.48; 1.35]*
Fowkes et al (AAA) 2010	*78*	*1675*	*90*	*1675*	*6.9%*	*0.87 [0.64; 1.16]*
Bowman et al (ASCEND) 2018	*309*	*7740*	*315*	*7740*	*17.7%*	*0.98 [0.84; 1.14]*
McNeil et al (ASPREE) 2018	*295*	*9525*	*227*	*9589*	*15.5%*	*1.31 [1.10; 1.55]*
Steering et al (PHS) 1989	*79*	*11037*	*68*	*11034*	*5.9%*	*1.16 [0.84; 1.60]*
Total (95% CI)		74085		72509	95.6%	1.03 [0.94; 1.12]
Heterogeneity: Tau^2 = 0.0060; Chi^2 = 13.77, df = 10 (P = 0.18); I^2 = 27%						
yes						
Meade et al (TPT) 1998	*49*	*1268*	*51*	*1272*	*4.4%*	*0.96 [0.66; 1.42]*
Total (95% CI)		1268		1272	4.4%	0.96 [0.66; 1.42]
Heterogeneity: not applicable						
Total (95% CI)		75353		73781	100.0%	1.03 [0.94; 1.12]
**Prediction interval**						**[0.86; 1.22]**

Heterogeneity: Tau^2 = 0.0044; Chi^2 = 13.89, df = 11 (P = 0.24); I^2 = 21%
Residual heterogeneity: Tau^2 = NA; Chi^2 = 13.77, df = 10 (P = 0.18); I^2 = 27%
Test for overall effect: Z = 0.59 (P = 0.56)

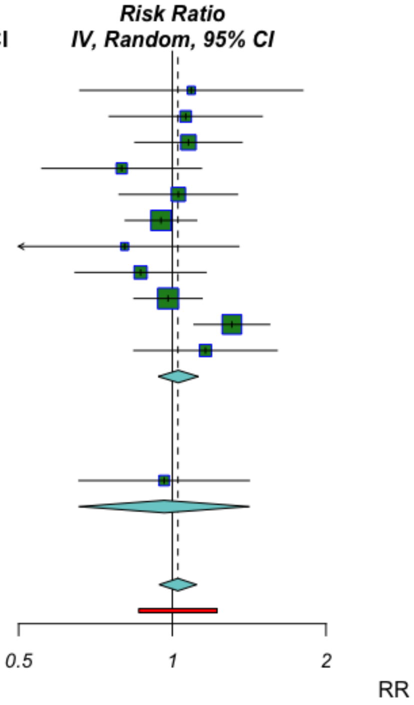

Supplement: Supplementary file 11 [file datasheet8.pdf]
