# Supplementary material for: Pros and Cons of Aspirin for the Primary Prevention of Cardiovascular Events: A Secondary Study of Trial Sequential Analysis
Source: Front Pharmacol. 2021 Jan 14;11:592116. doi: 10.3389/fphar.2020.592116 (PMC7845480; doi:10.3389/fphar.2020.592116)

Appendix File S10. Sensitivity analyses on major bleeding.

Figure A. Summary forest plot for sensitivity analysis on major bleeding by year (excluding trials published before 2000)

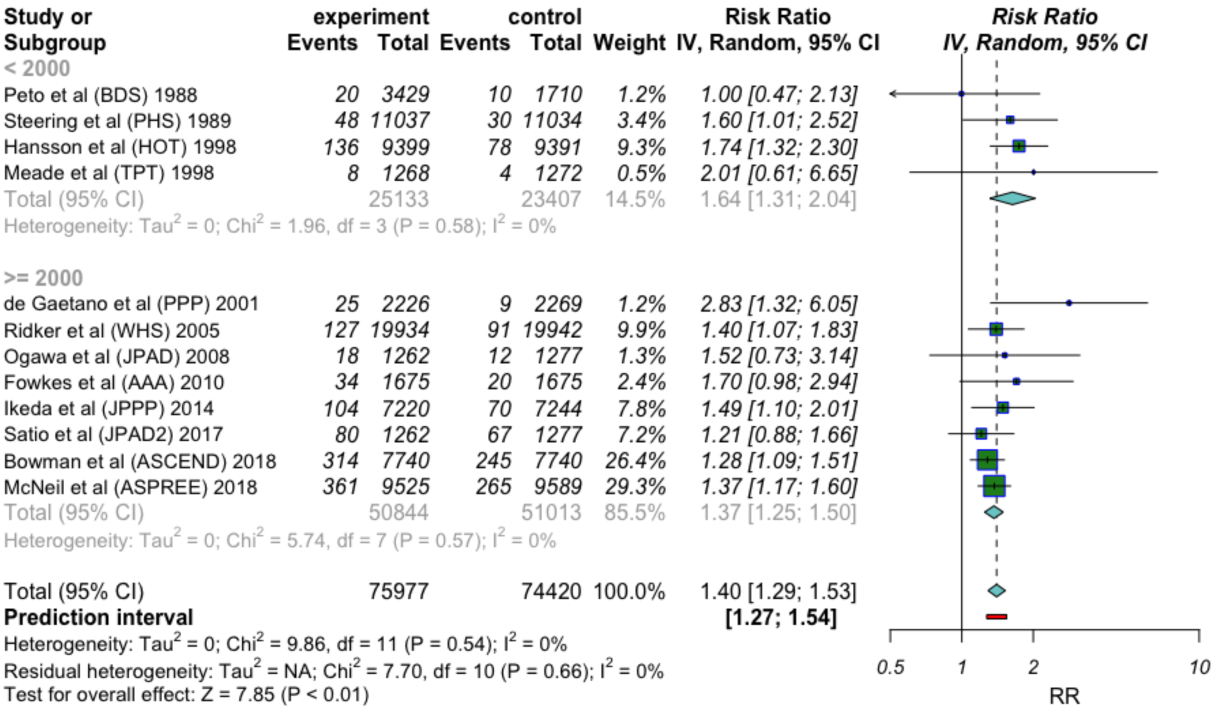

Figure B. Summary forest plot for sensitivity analysis on major bleeding by blinding (excluding open-label trials)

Study or Subgroup	experiment Events	experiment Total	control Events	control Total	Weight	Risk Ratio IV, Random, 95% CI
**double-blind**						
Steering et al (PHS) 1989	*48*	*11037*	*30*	*11034*	*3.4%*	*1.60 [1.01; 2.52]*
Hansson et al (HOT) 1998	*136*	*9399*	*78*	*9391*	*9.3%*	*1.74 [1.32; 2.30]*
Meade et al (TPT) 1998	*8*	*1268*	*4*	*1272*	*0.5%*	*2.01 [0.61; 6.65]*
Ridker et al (WHS) 2005	*127*	*19934*	*91*	*19942*	*9.9%*	*1.40 [1.07; 1.83]*
Fowkes et al (AAA) 2010	*34*	*1675*	*20*	*1675*	*2.4%*	*1.70 [0.98; 2.94]*
Bowman et al (ASCEND) 2018	*314*	*7740*	*245*	*7740*	*26.4%*	*1.28 [1.09; 1.51]*
McNeil et al (ASPREE) 2018	*361*	*9525*	*265*	*9589*	*29.3%*	*1.37 [1.17; 1.60]*
Total (95% CI)		60578		60643	81.2%	1.40 [1.28; 1.54]
Heterogeneity: $Tau^2 = 0$; $Chi^2 = 4.73$, df = 6 (P = 0.58); $I^2 = 0\%$						
**open-label**						
Peto et al (BDS) 1988	*20*	*3429*	*10*	*1710*	*1.2%*	*1.00 [0.47; 2.13]*
de Gaetano et al (PPP) 2001	*25*	*2226*	*9*	*2269*	*1.2%*	*2.83 [1.32; 6.05]*
Ogawa et al (JPAD) 2008	*18*	*1262*	*12*	*1277*	*1.3%*	*1.52 [0.73; 3.14]*
Ikeda et al (JPPP) 2014	*104*	*7220*	*70*	*7244*	*7.8%*	*1.49 [1.10; 2.01]*
Satio et al (JPAD2) 2017	*80*	*1262*	*67*	*1277*	*7.2%*	*1.21 [0.88; 1.66]*
Total (95% CI)		15399		13777	18.8%	1.42 [1.11; 1.80]
Heterogeneity: $Tau^2 = 0.0166$; $Chi^2 = 5.13$, df = 4 (P = 0.27); $I^2 = 22\%$						
Total (95% CI)		75977		74420	100.0%	1.40 [1.29; 1.53]
**Prediction interval**						**[1.27; 1.54]**

Heterogeneity: $Tau^2 = 0$; $Chi^2 = 9.86$, df = 11 (P = 0.54); $I^2 = 0\%$
Residual heterogeneity: Tau^2 = NA; $Chi^2 = 9.86$, df = 10 (P = 0.45); $I^2 = 0\%$
Test for overall effect: Z = 7.85 (P < 0.01)

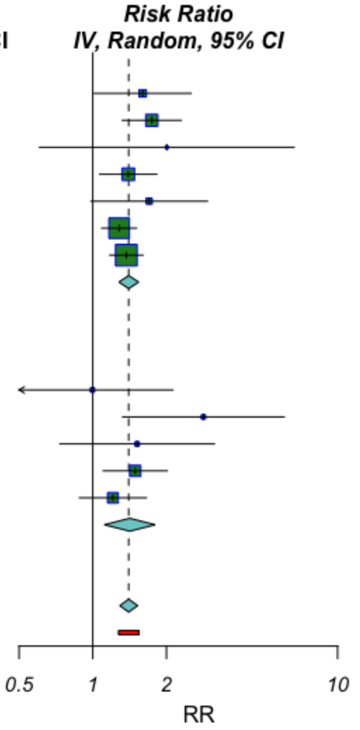

Figure C. Summary forest plot for sensitivity analysis on major bleeding by study quality (excluding high risk trials)

Study or Subgroup	experiment Events	experiment Total	control Events	control Total	Weight	Risk Ratio IV, Random, 95% CI
**High risk**						
Peto et al (BDS) 1988	*20*	*3429*	*10*	*1710*	*1.2%*	*1.00 [0.47; 2.13]*
Steering et al (PHS) 1989	*48*	*11037*	*30*	*11034*	*3.4%*	*1.60 [1.01; 2.52]*
Meade et al (TPT) 1998	*8*	*1268*	*4*	*1272*	*0.5%*	*2.01 [0.61; 6.65]*
de Gaetano et al (PPP) 2001	*25*	*2226*	*9*	*2269*	*1.2%*	*2.83 [1.32; 6.05]*
Ogawa et al (JPAD) 2008	*18*	*1262*	*12*	*1277*	*1.3%*	*1.52 [0.73; 3.14]*
Ikeda et al (JPPP) 2014	*104*	*7220*	*70*	*7244*	*7.8%*	*1.49 [1.10; 2.01]*
Satio et al (JPAD2) 2017	*80*	*1262*	*67*	*1277*	*7.2%*	*1.21 [0.88; 1.66]*
Total (95% CI)		27704		26083	22.8%	1.44 [1.21; 1.72]
Heterogeneity: $Tau^2 = 0$; $Chi^2 = 5.71$, df = 6 (P = 0.46); $I^2 = 0\%$						
**Low risk**						
Hansson et al (HOT) 1998	*136*	*9399*	*78*	*9391*	*9.3%*	*1.74 [1.32; 2.30]*
Ridker et al (WHS) 2005	*127*	*19934*	*91*	*19942*	*9.9%*	*1.40 [1.07; 1.83]*
Fowkes et al (AAA) 2010	*34*	*1675*	*20*	*1675*	*2.4%*	*1.70 [0.98; 2.94]*
Bowman et al (ASCEND) 2018	*314*	*7740*	*245*	*7740*	*26.4%*	*1.28 [1.09; 1.51]*
McNeil et al (ASPREE) 2018	*361*	*9525*	*265*	*9589*	*29.3%*	*1.37 [1.17; 1.60]*
Total (95% CI)		48273		48337	77.2%	1.39 [1.26; 1.53]
Heterogeneity: $Tau^2 = 0.0001$; $Chi^2 = 4.04$, df = 4 (P = 0.40); $I^2 = 1\%$						
Total (95% CI)		75977		74420	100.0%	1.40 [1.29; 1.53]
**Prediction interval**						**[1.27; 1.54]**

Heterogeneity: $Tau^2 = 0$; $Chi^2 = 9.86$, df = 11 (P = 0.54); $I^2 = 0\%$
Residual heterogeneity: Tau^2 = NA; $Chi^2 = 9.75$, df = 10 (P = 0.46); $I^2 = 0\%$
Test for overall effect: Z = 7.85 (P < 0.01)

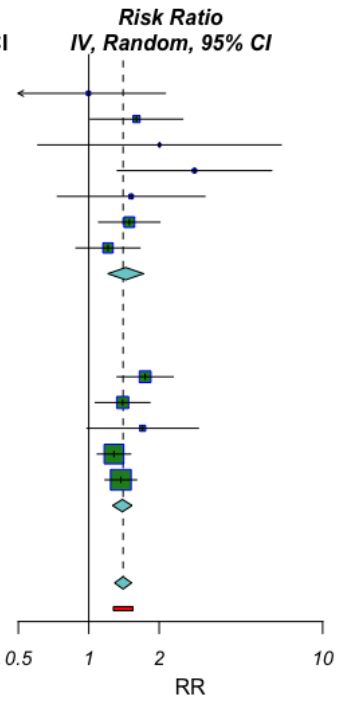

Figure D. Summary forest plot for sensitivity analysis on major bleeding by asymptomatic PAD (excluding asymptomatic PAD trials)

Study or Subgroup	experiment Events	experiment Total	control Events	control Total	Weight	Risk Ratio IV, Random, 95% CI
no						
Peto et al (BDS) 1988	20	3429	10	1710	1.2%	1.00 [0.47; 2.13]
Steering et al (PHS) 1989	48	11037	30	11034	3.4%	1.60 [1.01; 2.52]
Hansson et al (HOT) 1998	136	9399	78	9391	9.3%	1.74 [1.32; 2.30]
Meade et al (TPT) 1998	8	1268	4	1272	0.5%	2.01 [0.61; 6.65]
de Gaetano et al (PPP) 2001	25	2226	9	2269	1.2%	2.83 [1.32; 6.05]
Ridker et al (WHS) 2005	127	19934	91	19942	9.9%	1.40 [1.07; 1.83]
Ogawa et al (JPAD) 2008	18	1262	12	1277	1.3%	1.52 [0.73; 3.14]
Ikeda et al (JPPP) 2014	104	7220	70	7244	7.8%	1.49 [1.10; 2.01]
Satio et al (JPAD2) 2017	80	1262	67	1277	7.2%	1.21 [0.88; 1.66]
Bowman et al (ASCEND) 2018	314	7740	245	7740	26.4%	1.28 [1.09; 1.51]
McNeil et al (ASPREE) 2018	361	9525	265	9589	29.3%	1.37 [1.17; 1.60]
Total (95% CI)		74302		72745	97.6%	1.40 [1.28; 1.52]
Heterogeneity: $Tau^2 = 0$; $Chi^2 = 9.37$, df = 10 (P = 0.50); $I^2 = 0\%$						
yes						
Fowkes et al (AAA) 2010	34	1675	20	1675	2.4%	1.70 [0.98; 2.94]
Total (95% CI)		1675		1675	2.4%	1.70 [0.98; 2.94]
Heterogeneity: not applicable						
Total (95% CI)		75977		74420	100.0%	1.40 [1.29; 1.53]
**Prediction interval**						**[1.27; 1.54]**

Heterogeneity: $Tau^2 = 0$; $Chi^2 = 9.86$, df = 11 (P = 0.54); $I^2 = 0\%$
Residual heterogeneity: $Tau^2 = NA$; $Chi^2 = 9.37$, df = 10 (P = 0.50); $I^2 = 0\%$
Test for overall effect: Z = 7.85 (P < 0.01)

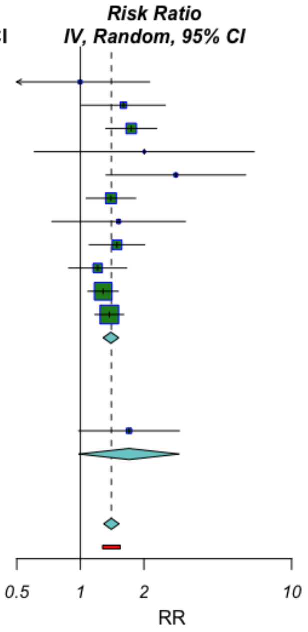

Figure E. Summary forest plot for sensitivity analysis on major bleeding by male populations (excluding 100% male individual trials)

Study or Subgroup	experiment Events	experiment Total	control Events	control Total	Weight	Risk Ratio IV, Random, 95% CI
male						
Peto et al (BDS) 1988	20	3429	10	1710	1.2%	1.00 [0.47; 2.13]
Steering et al (PHS) 1989	48	11037	30	11034	3.4%	1.60 [1.01; 2.52]
Meade et al (TPT) 1998	8	1268	4	1272	0.5%	2.01 [0.61; 6.65]
Total (95% CI)		15734		14016	5.2%	1.46 [1.01; 2.11]
Heterogeneity: $Tau^2 = 0$; $Chi^2 = 1.4$, df = 2 (P = 0.50); $I^2 = 0\%$						
not all male						
Hansson et al (HOT) 1998	136	9399	78	9391	9.3%	1.74 [1.32; 2.30]
de Gaetano et al (PPP) 2001	25	2226	9	2269	1.2%	2.83 [1.32; 6.05]
Ridker et al (WHS) 2005	127	19934	91	19942	9.9%	1.40 [1.07; 1.83]
Ogawa et al (JPAD) 2008	18	1262	12	1277	1.3%	1.52 [0.73; 3.14]
Fowkes et al (AAA) 2010	34	1675	20	1675	2.4%	1.70 [0.98; 2.94]
Ikeda et al (JPPP) 2014	104	7220	70	7244	7.8%	1.49 [1.10; 2.01]
Satio et al (JPAD2) 2017	80	1262	67	1277	7.2%	1.21 [0.88; 1.66]
Bowman et al (ASCEND) 2018	314	7740	245	7740	26.4%	1.28 [1.09; 1.51]
McNeil et al (ASPREE) 2018	361	9525	265	9589	29.3%	1.37 [1.17; 1.60]
Total (95% CI)		60243		60404	94.8%	1.40 [1.28; 1.54]
Heterogeneity: $Tau^2 = 0.0010$; $Chi^2 = 8.41$, df = 8 (P = 0.39); $I^2 = 5\%$						
**Total (95% CI)**		75977		74420	100.0%	1.40 [1.29; 1.53]
**Prediction interval**						**[1.27; 1.54]**

Heterogeneity: $Tau^2 = 0$; $Chi^2 = 9.86$, df = 11 (P = 0.54); $I^2 = 0\%$
Residual heterogeneity: Tau^2 = NA; $Chi^2 = 9.81$, df = 10 (P = 0.46); $I^2 = 0\%$
Test for overall effect: Z = 7.85 (P < 0.01)

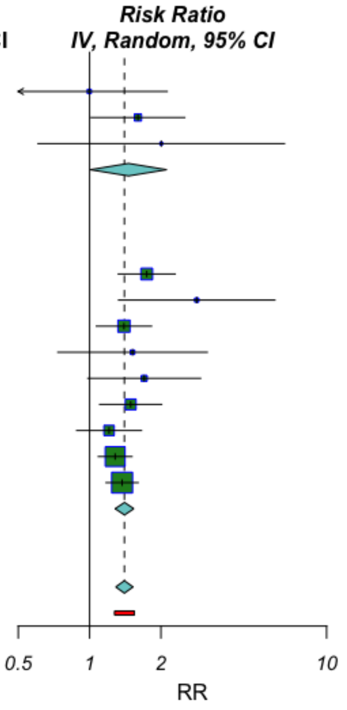

Figure F. Summary forest plot for sensitivity analysis on major bleeding by diabetes status (excluding 100% diabetic individual trials and restricted on 100% diabetic individual trials)

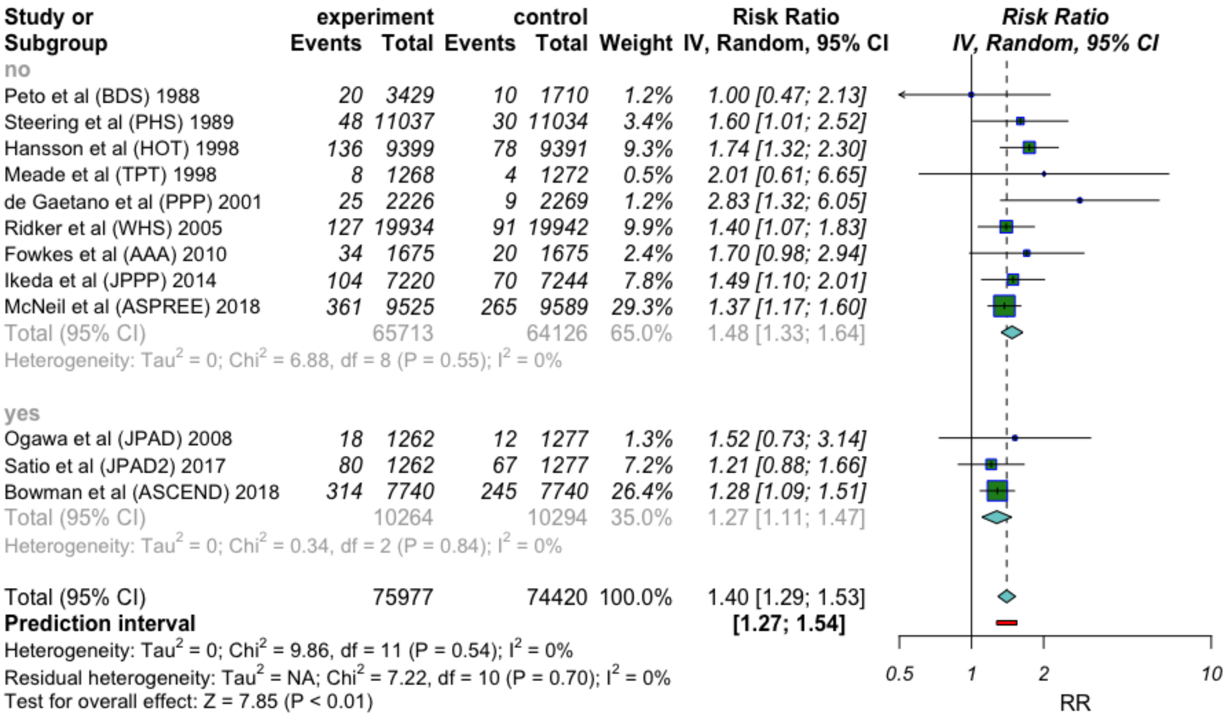

Figure G. Summary forest plot for sensitivity analysis on major bleeding by placebo use (excluding placebo use trials)

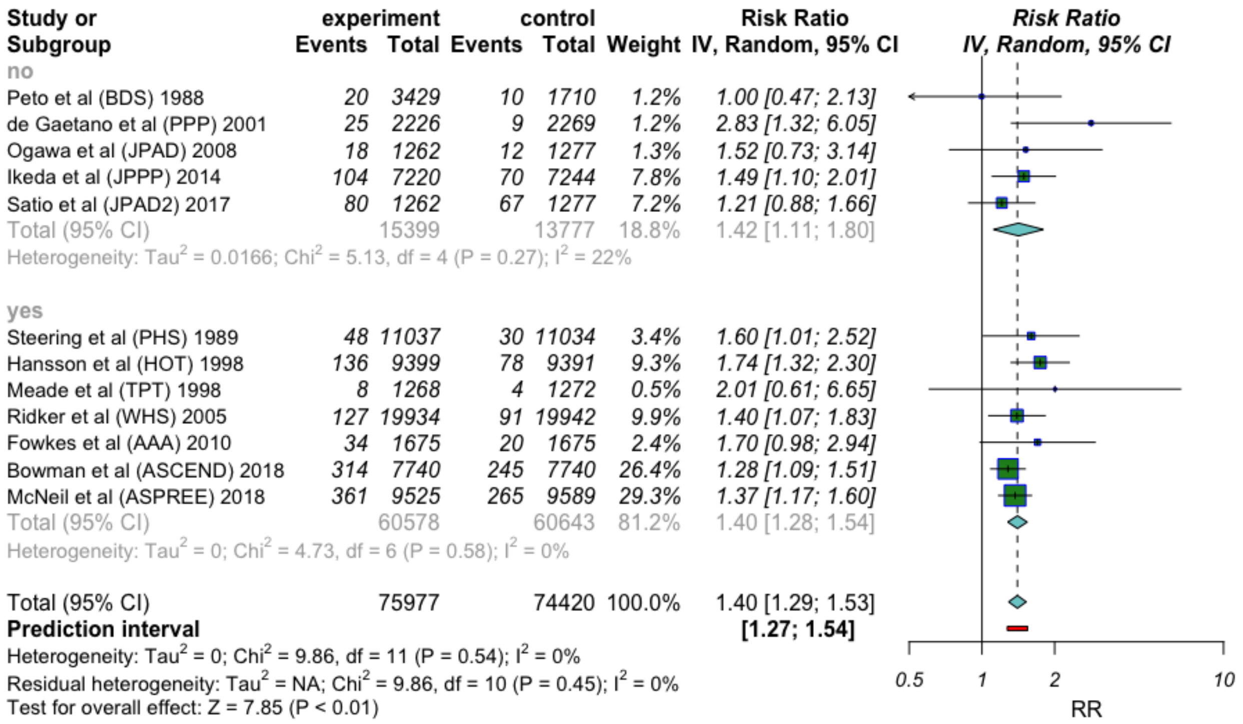

Figure H. Summary forest plot for sensitivity analysis on major bleeding excluding TPT trial

Study or Subgroup	experiment Events	experiment Total	control Events	control Total	Weight	Risk Ratio IV, Random, 95% CI
no						
Peto et al (BDS) 1988	20	3429	10	1710	1.2%	1.00 [0.47; 2.13]
Steering et al (PHS) 1989	48	11037	30	11034	3.4%	1.60 [1.01; 2.52]
Hansson et al (HOT) 1998	136	9399	78	9391	9.3%	1.74 [1.32; 2.30]
de Gaetano et al (PPP) 2001	25	2226	9	2269	1.2%	2.83 [1.32; 6.05]
Ridker et al (WHS) 2005	127	19934	91	19942	9.9%	1.40 [1.07; 1.83]
Ogawa et al (JPAD) 2008	18	1262	12	1277	1.3%	1.52 [0.73; 3.14]
Fowkes et al (AAA) 2010	34	1675	20	1675	2.4%	1.70 [0.98; 2.94]
Ikeda et al (JPPP) 2014	104	7220	70	7244	7.8%	1.49 [1.10; 2.01]
Satio et al (JPAD2) 2017	80	1262	67	1277	7.2%	1.21 [0.88; 1.66]
Bowman et al (ASCEND) 2018	314	7740	245	7740	26.4%	1.28 [1.09; 1.51]
McNeil et al (ASPREE) 2018	361	9525	265	9589	29.3%	1.37 [1.17; 1.60]
Total (95% CI)		74709		73148	99.5%	1.40 [1.29; 1.52]
Heterogeneity: $Tau^2 = 0$; $Chi^2 = 9.51$, df = 10 (P = 0.48); $I^2 = 0\%$						
yes						
Meade et al (TPT) 1998	8	1268	4	1272	0.5%	2.01 [0.61; 6.65]
Total (95% CI)		1268		1272	0.5%	2.01 [0.61; 6.65]
Heterogeneity: not applicable						
Total (95% CI)		75977		74420	100.0%	1.40 [1.29; 1.53]
**Prediction interval**						**[1.27; 1.54]**

Heterogeneity: $Tau^2 = 0$; $Chi^2 = 9.86$, df = 11 (P = 0.54); $I^2 = 0\%$
Residual heterogeneity: $Tau^2 = NA$; $Chi^2 = 9.51$, df = 10 (P = 0.48); $I^2 = 0\%$
Test for overall effect: Z = 7.85 (P < 0.01)

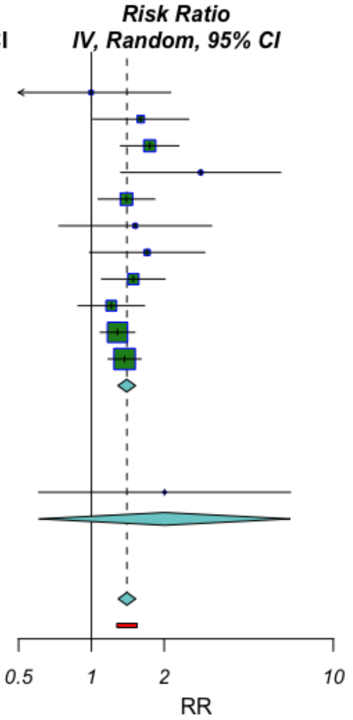

Supplement: Supplementary file 12 [file datasheet9.pdf]
