# Supplementary material for: Pros and Cons of Aspirin for the Primary Prevention of Cardiovascular Events: A Secondary Study of Trial Sequential Analysis
Source: Front Pharmacol. 2021 Jan 14;11:592116. doi: 10.3389/fphar.2020.592116 (PMC7845480; doi:10.3389/fphar.2020.592116)

# Appendix File S11. Sensitivity analyses on intracranial bleeding.

**Figure A**. Summary forest plot for sensitivity analysis on intracranial bleeding by year (excluding trials published before 2000)

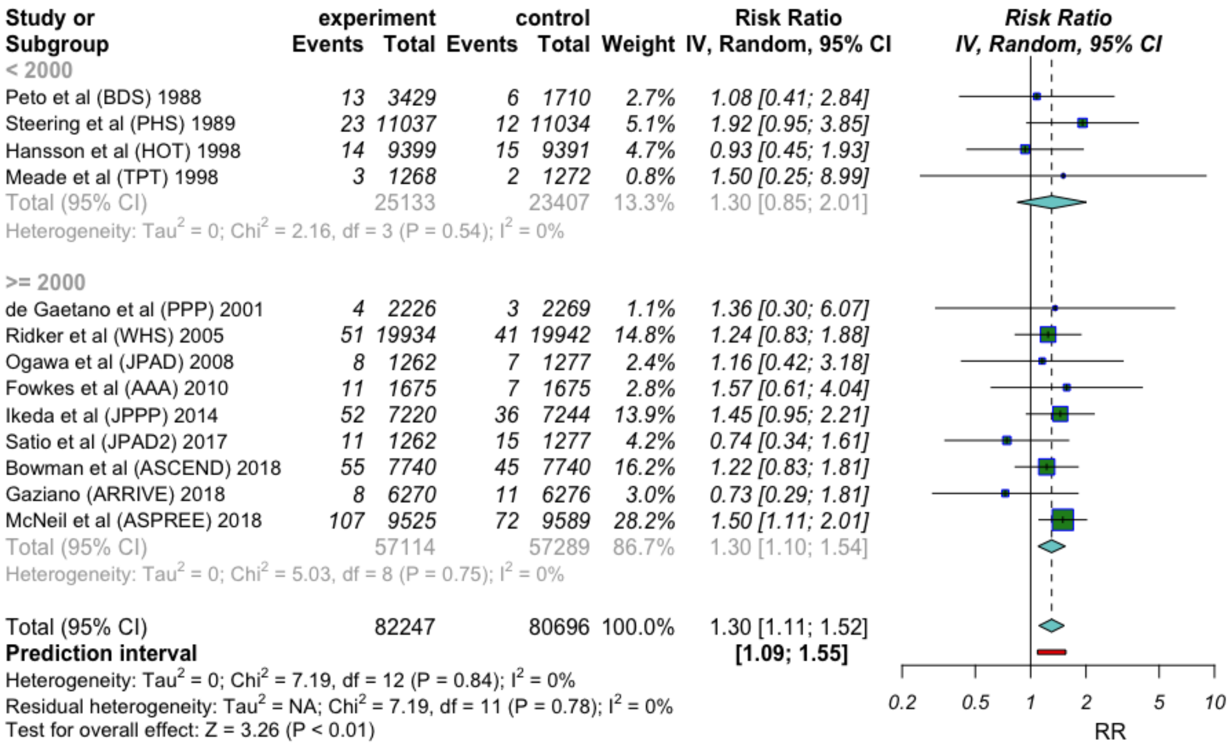

**Figure B**. Summary forest plot for sensitivity analysis on intracranial bleeding by blinding (excluding open-label trials)

| Study or Subgroup | experiment Events | experiment Total | control Events | control Total | Weight | Risk Ratio IV, Random, 95% CI |
|---|---|---|---|---|---|---|
| **double-blind** | | | | | | |
| Steering et al (PHS) 1989 | 23 | 11037 | 12 | 11034 | 5.1% | 1.92 [0.95; 3.85] |
| Hansson et al (HOT) 1998 | 14 | 9399 | 15 | 9391 | 4.7% | 0.93 [0.45; 1.93] |
| Meade et al (TPT) 1998 | 3 | 1268 | 2 | 1272 | 0.8% | 1.50 [0.25; 8.99] |
| Ridker et al (WHS) 2005 | 51 | 19934 | 41 | 19942 | 14.8% | 1.24 [0.83; 1.88] |
| Fowkes et al (AAA) 2010 | 11 | 1675 | 7 | 1675 | 2.8% | 1.57 [0.61; 4.04] |
| Bowman et al (ASCEND) 2018 | 55 | 7740 | 45 | 7740 | 16.2% | 1.22 [0.83; 1.81] |
| Gaziano (ARRIVE) 2018 | 8 | 6270 | 11 | 6276 | 3.0% | 0.73 [0.29; 1.81] |
| McNeil et al (ASPREE) 2018 | 107 | 9525 | 72 | 9589 | 28.2% | 1.50 [1.11; 2.01] |
| Total (95% CI) | | 66848 | | 66919 | 75.7% | 1.33 [1.11; 1.59] |
| Heterogeneity: $Tau^2 = 0$; $Chi^2 = 4.67$, df = 7 (P = 0.70); $I^2 = 0\%$ | | | | | | |
| **open-label** | | | | | | |
| Peto et al (BDS) 1988 | 13 | 3429 | 6 | 1710 | 2.7% | 1.08 [0.41; 2.84] |
| de Gaetano et al (PPP) 2001 | 4 | 2226 | 3 | 2269 | 1.1% | 1.36 [0.30; 6.07] |
| Ogawa et al (JPAD) 2008 | 8 | 1262 | 7 | 1277 | 2.4% | 1.16 [0.42; 3.18] |
| Ikeda et al (JPPP) 2014 | 52 | 7220 | 36 | 7244 | 13.9% | 1.45 [0.95; 2.21] |
| Satio et al (JPAD2) 2017 | 11 | 1262 | 15 | 1277 | 4.2% | 0.74 [0.34; 1.61] |
| Total (95% CI) | | 15399 | | 13777 | 24.3% | 1.22 [0.89; 1.68] |
| Heterogeneity: $Tau^2 = 0$; $Chi^2 = 2.31$, df = 4 (P = 0.68); $I^2 = 0\%$ | | | | | | |
| Total (95% CI) | | 82247 | | 80696 | 100.0% | 1.30 [1.11; 1.52] |
| **Prediction interval** | | | | | | **[1.09; 1.55]** |

Heterogeneity: $Tau^2 = 0$; $Chi^2 = 7.19$, df = 12 (P = 0.84); $I^2 = 0\%$
Residual heterogeneity: $Tau^2$ = NA; $Chi^2 = 6.98$, df = 11 (P = 0.80); $I^2 = 0\%$
Test for overall effect: Z = 3.26 (P < 0.01)

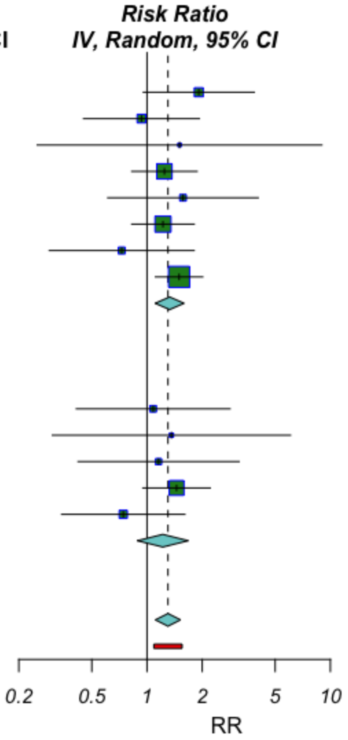

**Figure C**. Summary forest plot for sensitivity analysis on intracranial bleeding by study quality (excluding high risk trials)

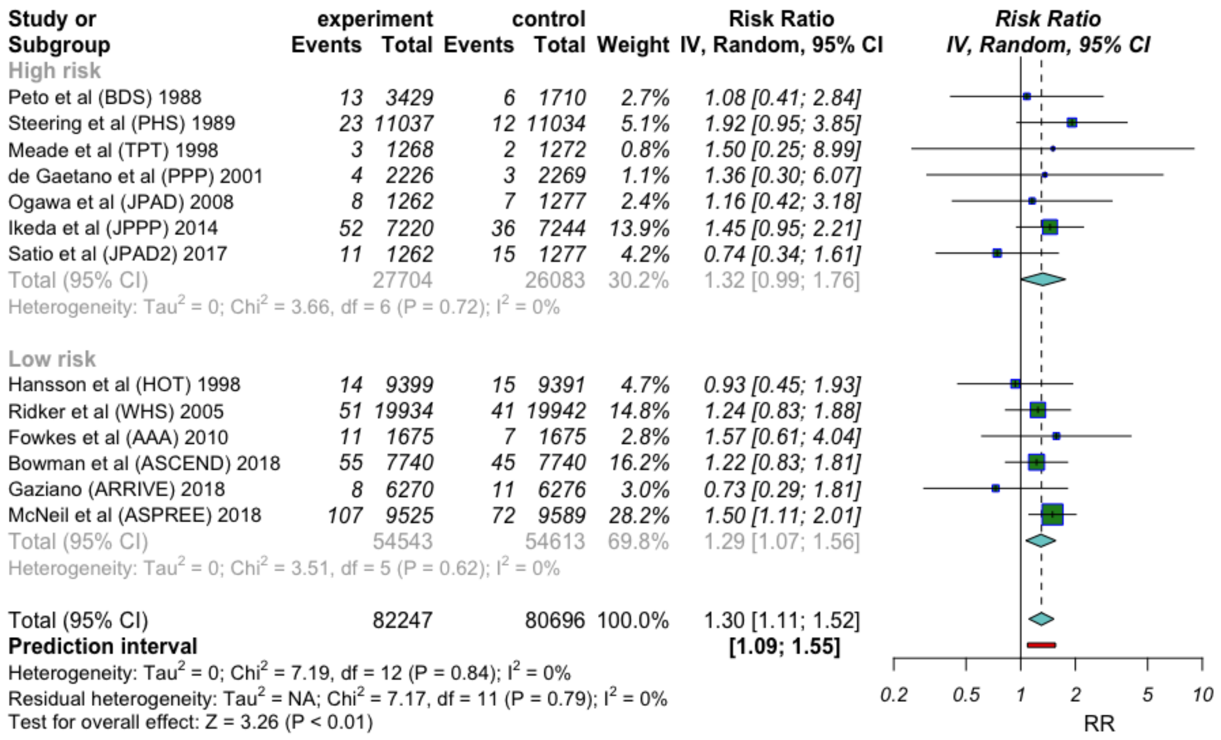

**Figure D**. Summary forest plot for sensitivity analysis on intracranial bleeding by asymptomatic PAD (excluding asymptomatic PAD trials)

| Study or Subgroup | experiment Events | experiment Total | control Events | control Total | Weight | Risk Ratio IV, Random, 95% CI |
|---|---|---|---|---|---|---|
| no | | | | | | |
| Peto et al (BDS) 1988 | 13 | 3429 | 6 | 1710 | 2.7% | 1.08 [0.41; 2.84] |
| Steering et al (PHS) 1989 | 23 | 11037 | 12 | 11034 | 5.1% | 1.92 [0.95; 3.85] |
| Hansson et al (HOT) 1998 | 14 | 9399 | 15 | 9391 | 4.7% | 0.93 [0.45; 1.93] |
| Meade et al (TPT) 1998 | 3 | 1268 | 2 | 1272 | 0.8% | 1.50 [0.25; 8.99] |
| de Gaetano et al (PPP) 2001 | 4 | 2226 | 3 | 2269 | 1.1% | 1.36 [0.30; 6.07] |
| Ridker et al (WHS) 2005 | 51 | 19934 | 41 | 19942 | 14.8% | 1.24 [0.83; 1.88] |
| Ogawa et al (JPAD) 2008 | 8 | 1262 | 7 | 1277 | 2.4% | 1.16 [0.42; 3.18] |
| Ikeda et al (JPPP) 2014 | 52 | 7220 | 36 | 7244 | 13.9% | 1.45 [0.95; 2.21] |
| Satio et al (JPAD2) 2017 | 11 | 1262 | 15 | 1277 | 4.2% | 0.74 [0.34; 1.61] |
| Bowman et al (ASCEND) 2018 | 55 | 7740 | 45 | 7740 | 16.2% | 1.22 [0.83; 1.81] |
| Gaziano (ARRIVE) 2018 | 8 | 6270 | 11 | 6276 | 3.0% | 0.73 [0.29; 1.81] |
| McNeil et al (ASPREE) 2018 | 107 | 9525 | 72 | 9589 | 28.2% | 1.50 [1.11; 2.01] |
| Total (95% CI) | | 80572 | | 79021 | 97.2% | 1.29 [1.10; 1.52] |
| Heterogeneity: $Tau^2 = 0$; $Chi^2 = 7.03$, df = 11 (P = 0.80); $I^2 = 0\%$ | | | | | | |
| yes | | | | | | |
| Fowkes et al (AAA) 2010 | 11 | 1675 | 7 | 1675 | 2.8% | 1.57 [0.61; 4.04] |
| Total (95% CI) | | 1675 | | 1675 | 2.8% | 1.57 [0.61; 4.04] |
| Heterogeneity: not applicable | | | | | | |
| **Total (95% CI)** | | 82247 | | 80696 | 100.0% | 1.30 [1.11; 1.52] |
| **Prediction interval** | | | | | | **[1.09; 1.55]** |

Heterogeneity: $Tau^2 = 0$; $Chi^2 = 7.19$, df = 12 (P = 0.84); $I^2 = 0\%$
Residual heterogeneity: $Tau^2$ = NA; $Chi^2 = 7.03$, df = 11 (P = 0.80); $I^2 = 0\%$
Test for overall effect: Z = 3.26 (P < 0.01)

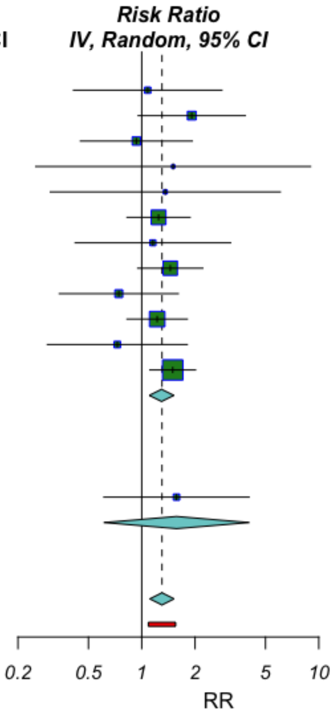

**Figure E**. Summary forest plot for sensitivity analysis on intracranial bleeding by male populations (excluding 100% male individual trials)

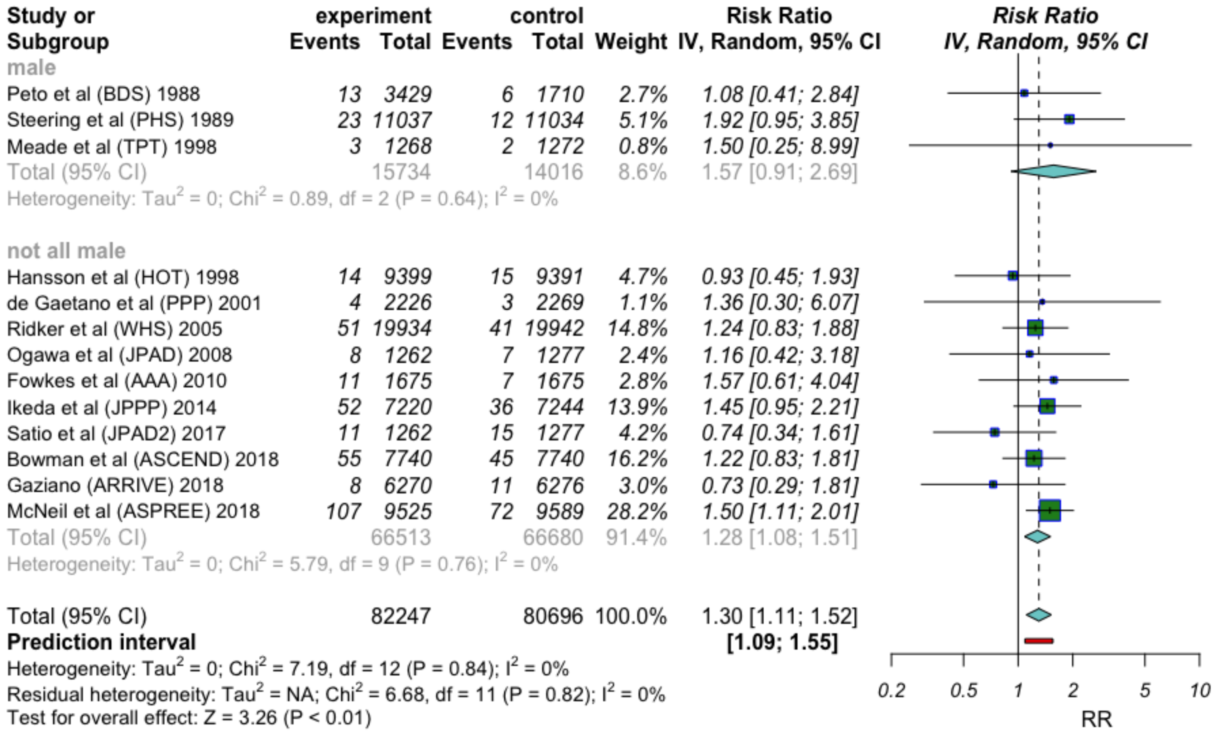

**Figure F**. Summary forest plot for sensitivity analysis on intracranial bleeding by diabetes status (excluding 100% diabetic individual trials and restricted on 100% diabetic individual trials)

| Study or Subgroup | experiment Events | experiment Total | control Events | control Total | Weight | Risk Ratio IV, Random, 95% CI |
|---|---|---|---|---|---|---|
| no | | | | | | |
| Peto et al (BDS) 1988 | 13 | 3429 | 6 | 1710 | 2.7% | 1.08 [0.41; 2.84] |
| Steering et al (PHS) 1989 | 23 | 11037 | 12 | 11034 | 5.1% | 1.92 [0.95; 3.85] |
| Hansson et al (HOT) 1998 | 14 | 9399 | 15 | 9391 | 4.7% | 0.93 [0.45; 1.93] |
| Meade et al (TPT) 1998 | 3 | 1268 | 2 | 1272 | 0.8% | 1.50 [0.25; 8.99] |
| de Gaetano et al (PPP) 2001 | 4 | 2226 | 3 | 2269 | 1.1% | 1.36 [0.30; 6.07] |
| Ridker et al (WHS) 2005 | 51 | 19934 | 41 | 19942 | 14.8% | 1.24 [0.83; 1.88] |
| Fowkes et al (AAA) 2010 | 11 | 1675 | 7 | 1675 | 2.8% | 1.57 [0.61; 4.04] |
| Ikeda et al (JPPP) 2014 | 52 | 7220 | 36 | 7244 | 13.9% | 1.45 [0.95; 2.21] |
| Gaziano (ARRIVE) 2018 | 8 | 6270 | 11 | 6276 | 3.0% | 0.73 [0.29; 1.81] |
| McNeil et al (ASPREE) 2018 | 107 | 9525 | 72 | 9589 | 28.2% | 1.50 [1.11; 2.01] |
| Total (95% CI) | | 71983 | | 70402 | 77.2% | 1.36 [1.14; 1.63] |
| Heterogeneity: $Tau^2 = 0$; $Chi^2 = 4.75$, df = 9 (P = 0.86); $I^2 = 0\%$ | | | | | | |
| yes | | | | | | |
| Ogawa et al (JPAD) 2008 | 8 | 1262 | 7 | 1277 | 2.4% | 1.16 [0.42; 3.18] |
| Satio et al (JPAD2) 2017 | 11 | 1262 | 15 | 1277 | 4.2% | 0.74 [0.34; 1.61] |
| Bowman et al (ASCEND) 2018 | 55 | 7740 | 45 | 7740 | 16.2% | 1.22 [0.83; 1.81] |
| Total (95% CI) | | 10264 | | 10294 | 22.8% | 1.11 [0.80; 1.54] |
| Heterogeneity: $Tau^2 = 0$; $Chi^2 = 1.28$, df = 2 (P = 0.53); $I^2 = 0\%$ | | | | | | |
| Total (95% CI) | | 82247 | | 80696 | 100.0% | 1.30 [1.11; 1.52] |
| **Prediction interval** | | | | | | **[1.09; 1.55]** |

Heterogeneity: $Tau^2 = 0$; $Chi^2 = 7.19$, df = 12 (P = 0.84); $I^2 = 0\%$
Residual heterogeneity: $Tau^2 = NA$; $Chi^2 = 6.03$, df = 11 (P = 0.87); $I^2 = 0\%$
Test for overall effect: Z = 3.26 (P < 0.01)

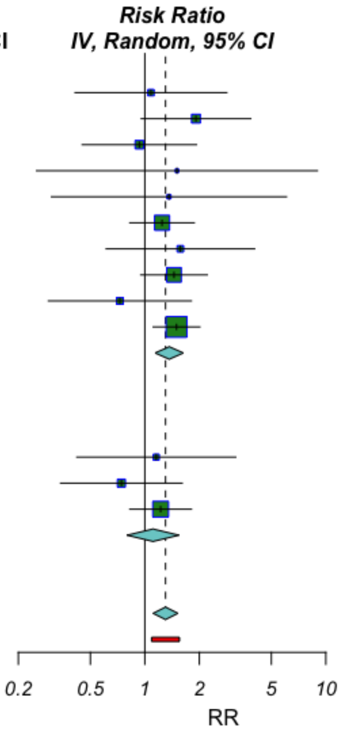

**Figure G**. Summary forest plot for sensitivity analysis on intracranial bleeding by placebo use (excluding placebo use trials)

| Study or Subgroup | experiment Events | experiment Total | control Events | control Total | Weight | Risk Ratio IV, Random, 95% CI |
|---|---|---|---|---|---|---|
| **no** | | | | | | |
| Peto et al (BDS) 1988 | *13* | *3429* | *6* | *1710* | *2.7%* | *1.08 [0.41; 2.84]* |
| de Gaetano et al (PPP) 2001 | *4* | *2226* | *3* | *2269* | *1.1%* | *1.36 [0.30; 6.07]* |
| Ogawa et al (JPAD) 2008 | *8* | *1262* | *7* | *1277* | *2.4%* | *1.16 [0.42; 3.18]* |
| Ikeda et al (JPPP) 2014 | *52* | *7220* | *36* | *7244* | *13.9%* | *1.45 [0.95; 2.21]* |
| Satio et al (JPAD2) 2017 | *11* | *1262* | *15* | *1277* | *4.2%* | *0.74 [0.34; 1.61]* |
| Total (95% CI) | | 15399 | | 13777 | 24.3% | 1.22 [0.89; 1.68] |
| Heterogeneity: $Tau^2 = 0$; $Chi^2 = 2.31$, df = 4 (P = 0.68); $I^2 = 0\%$ | | | | | | |
| **yes** | | | | | | |
| Steering et al (PHS) 1989 | *23* | *11037* | *12* | *11034* | *5.1%* | *1.92 [0.95; 3.85]* |
| Hansson et al (HOT) 1998 | *14* | *9399* | *15* | *9391* | *4.7%* | *0.93 [0.45; 1.93]* |
| Meade et al (TPT) 1998 | *3* | *1268* | *2* | *1272* | *0.8%* | *1.50 [0.25; 8.99]* |
| Ridker et al (WHS) 2005 | *51* | *19934* | *41* | *19942* | *14.8%* | *1.24 [0.83; 1.88]* |
| Fowkes et al (AAA) 2010 | *11* | *1675* | *7* | *1675* | *2.8%* | *1.57 [0.61; 4.04]* |
| Bowman et al (ASCEND) 2018 | *55* | *7740* | *45* | *7740* | *16.2%* | *1.22 [0.83; 1.81]* |
| Gaziano (ARRIVE) 2018 | *8* | *6270* | *11* | *6276* | *3.0%* | *0.73 [0.29; 1.81]* |
| McNeil et al (ASPREE) 2018 | *107* | *9525* | *72* | *9589* | *28.2%* | *1.50 [1.11; 2.01]* |
| Total (95% CI) | | 66848 | | 66919 | 75.7% | 1.33 [1.11; 1.59] |
| Heterogeneity: $Tau^2 = 0$; $Chi^2 = 4.67$, df = 7 (P = 0.70); $I^2 = 0\%$ | | | | | | |
| Total (95% CI) | | 82247 | | 80696 | 100.0% | 1.30 [1.11; 1.52] |
| **Prediction interval** | | | | | | **[1.09; 1.55]** |

Heterogeneity: $Tau^2 = 0$; $Chi^2 = 7.19$, df = 12 (P = 0.84); $I^2 = 0\%$
Residual heterogeneity: $Tau^2 = NA$; $Chi^2 = 6.98$, df = 11 (P = 0.80); $I^2 = 0\%$
Test for overall effect: Z = 3.26 (P < 0.01)

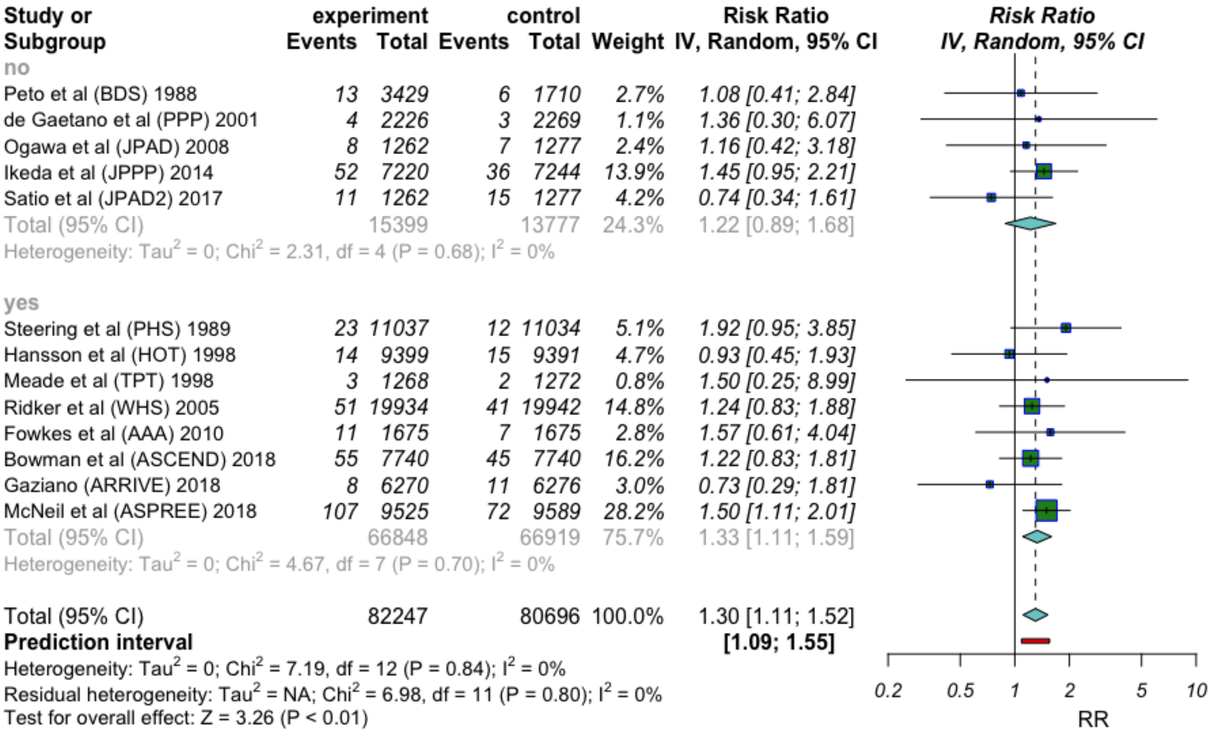

**Figure H**. Summary forest plot for sensitivity analysis on intracranial bleeding excluding TPT trial

| Study or Subgroup | experiment Events | experiment Total | control Events | control Total | Weight | Risk Ratio IV, Random, 95% CI |
|---|---|---|---|---|---|---|
| no | | | | | | |
| Peto et al (BDS) 1988 | *13* | *3429* | *6* | *1710* | *2.7%* | *1.08 [0.41; 2.84]* |
| Steering et al (PHS) 1989 | *23* | *11037* | *12* | *11034* | *5.1%* | *1.92 [0.95; 3.85]* |
| Hansson et al (HOT) 1998 | *14* | *9399* | *15* | *9391* | *4.7%* | *0.93 [0.45; 1.93]* |
| de Gaetano et al (PPP) 2001 | *4* | *2226* | *3* | *2269* | *1.1%* | *1.36 [0.30; 6.07]* |
| Ridker et al (WHS) 2005 | *51* | *19934* | *41* | *19942* | *14.8%* | *1.24 [0.83; 1.88]* |
| Ogawa et al (JPAD) 2008 | *8* | *1262* | *7* | *1277* | *2.4%* | *1.16 [0.42; 3.18]* |
| Fowkes et al (AAA) 2010 | *11* | *1675* | *7* | *1675* | *2.8%* | *1.57 [0.61; 4.04]* |
| Ikeda et al (JPPP) 2014 | *52* | *7220* | *36* | *7244* | *13.9%* | *1.45 [0.95; 2.21]* |
| Satio et al (JPAD2) 2017 | *11* | *1262* | *15* | *1277* | *4.2%* | *0.74 [0.34; 1.61]* |
| Bowman et al (ASCEND) 2018 | *55* | *7740* | *45* | *7740* | *16.2%* | *1.22 [0.83; 1.81]* |
| Gaziano (ARRIVE) 2018 | *8* | *6270* | *11* | *6276* | *3.0%* | *0.73 [0.29; 1.81]* |
| McNeil et al (ASPREE) 2018 | *107* | *9525* | *72* | *9589* | *28.2%* | *1.50 [1.11; 2.01]* |
| Total (95% CI) | | 80979 | | 79424 | 99.2% | 1.30 [1.11; 1.52] |
| Heterogeneity: $Tau^2 = 0$; $Chi^2 = 7.16$, df = 11 (P = 0.79); $I^2 = 0\%$ | | | | | | |
| yes | | | | | | |
| Meade et al (TPT) 1998 | *3* | *1268* | *2* | *1272* | *0.8%* | *1.50 [0.25; 8.99]* |
| Total (95% CI) | | 1268 | | 1272 | 0.8% | 1.50 [0.25; 8.99] |
| Heterogeneity: not applicable | | | | | | |
| Total (95% CI) | | 82247 | | 80696 | 100.0% | 1.30 [1.11; 1.52] |
| **Prediction interval** | | | | | | **[1.09; 1.55]** |

Heterogeneity: $Tau^2 = 0$; $Chi^2 = 7.19$, df = 12 (P = 0.84); $I^2 = 0\%$
Residual heterogeneity: $Tau^2 = NA$; $Chi^2 = 7.16$, df = 11 (P = 0.79); $I^2 = 0\%$
Test for overall effect: Z = 3.26 (P < 0.01)

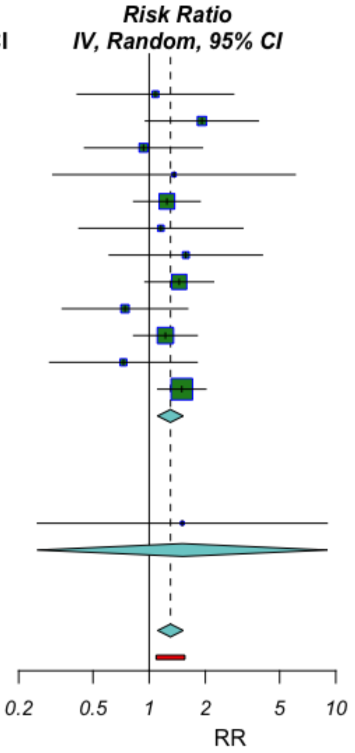

Supplement: Supplementary file 13 [file datasheet10.pdf]
