# Supplementary material for: Pros and Cons of Aspirin for the Primary Prevention of Cardiovascular Events: A Secondary Study of Trial Sequential Analysis
Source: Front Pharmacol. 2021 Jan 14;11:592116. doi: 10.3389/fphar.2020.592116 (PMC7845480; doi:10.3389/fphar.2020.592116)

## Appendix File S12. Sensitivity analyses on major gastrointestinal bleeding.

**Figure A**. Summary forest plot for sensitivity analysis on major gastrointestinal bleeding by year (excluding trials published before 2000)

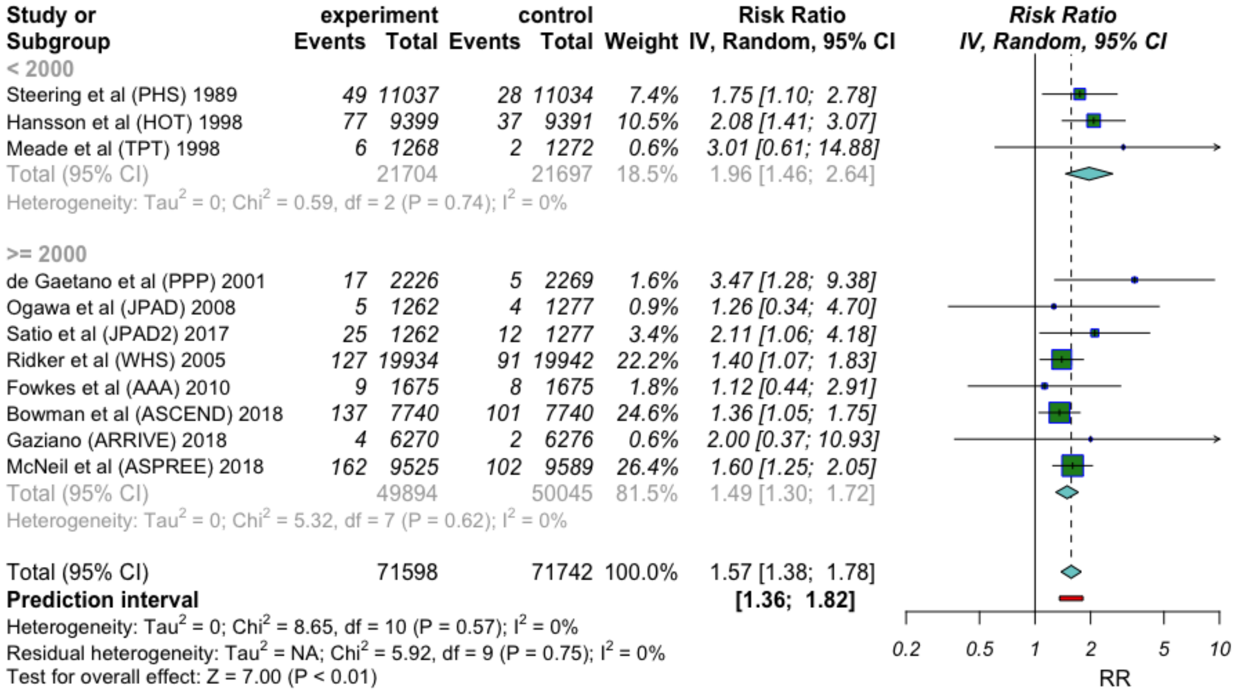

**Figure B**. Summary forest plot for sensitivity analysis on major gastrointestinal bleeding by blinding (excluding open-label trials)

| Study or Subgroup | experiment Events | experiment Total | control Events | control Total | Weight | Risk Ratio IV, Random, 95% CI |
|---|---|---|---|---|---|---|
| **double-blind** | | | | | | |
| Hansson et al (HOT) 1998 | 77 | 9399 | 37 | 9391 | 10.5% | 2.08 [1.41; 3.07] |
| Ridker et al (WHS) 2005 | 127 | 19934 | 91 | 19942 | 22.2% | 1.40 [1.07; 1.83] |
| Fowkes et al (AAA) 2010 | 9 | 1675 | 8 | 1675 | 1.8% | 1.12 [0.44; 2.91] |
| Bowman et al (ASCEND) 2018 | 137 | 7740 | 101 | 7740 | 24.6% | 1.36 [1.05; 1.75] |
| Gaziano (ARRIVE) 2018 | 4 | 6270 | 2 | 6276 | 0.6% | 2.00 [0.37; 10.93] |
| McNeil et al (ASPREE) 2018 | 162 | 9525 | 102 | 9589 | 26.4% | 1.60 [1.25; 2.05] |
| Meade et al (TPT) 1998 | 6 | 1268 | 2 | 1272 | 0.6% | 3.01 [0.61; 14.88] |
| Total (95% CI) | | 55811 | | 55885 | 86.6% | 1.52 [1.33; 1.74] |
| Heterogeneity: $Tau^2 = 0$; $Chi^2 = 4.97$, df = 6 ($P = 0.55$); $I^2 = 0\%$ | | | | | | |
| **open-label** | | | | | | |
| de Gaetano et al (PPP) 2001 | 17 | 2226 | 5 | 2269 | 1.6% | 3.47 [1.28; 9.38] |
| Ogawa et al (JPAD) 2008 | 5 | 1262 | 4 | 1277 | 0.9% | 1.26 [0.34; 4.70] |
| Satio et al (JPAD2) 2017 | 25 | 1262 | 12 | 1277 | 3.4% | 2.11 [1.06; 4.18] |
| Steering et al (PHS) 1989 | 49 | 11037 | 28 | 11034 | 7.4% | 1.75 [1.10; 2.78] |
| Total (95% CI) | | 15787 | | 15857 | 13.4% | 1.95 [1.38; 2.75] |
| Heterogeneity: $Tau^2 = 0$; $Chi^2 = 1.96$, df = 3 ($P = 0.58$); $I^2 = 0\%$ | | | | | | |
| Total (95% CI) | | 71598 | | 71742 | 100.0% | 1.57 [1.38; 1.78] |
| **Prediction interval** | | | | | | **[1.36; 1.82]** |

Heterogeneity: $Tau^2 = 0$; $Chi^2 = 8.65$, df = 10 ($P = 0.57$); $I^2 = 0\%$
Residual heterogeneity: $Tau^2 = NA$; $Chi^2 = 6.93$, df = 9 ($P = 0.64$); $I^2 = 0\%$
Test for overall effect: $Z = 7.00$ ($P < 0.01$)

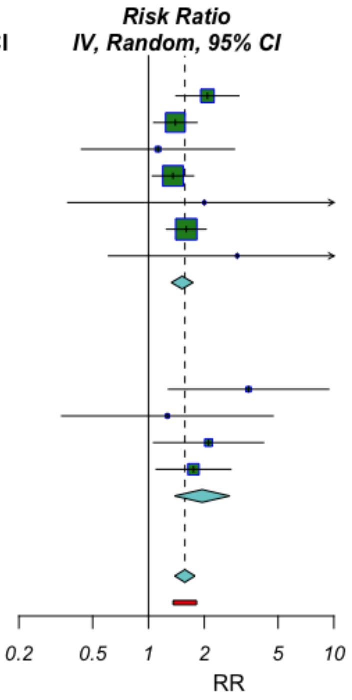

**Figure C**. Summary forest plot for sensitivity analysis on major gastrointestinal bleeding by study quality (excluding high risk trials)

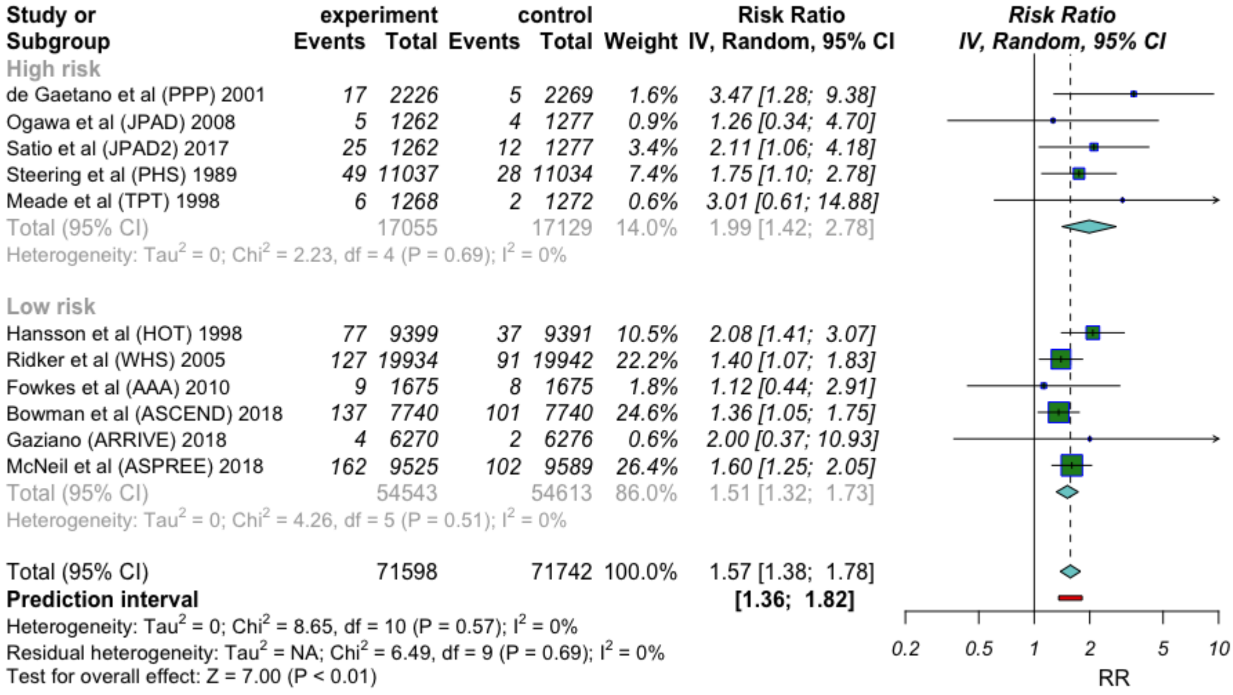

**Figure D**. Summary forest plot for sensitivity analysis on major gastrointestinal bleeding by asymptomatic PAD (excluding asymptomatic PAD trials)

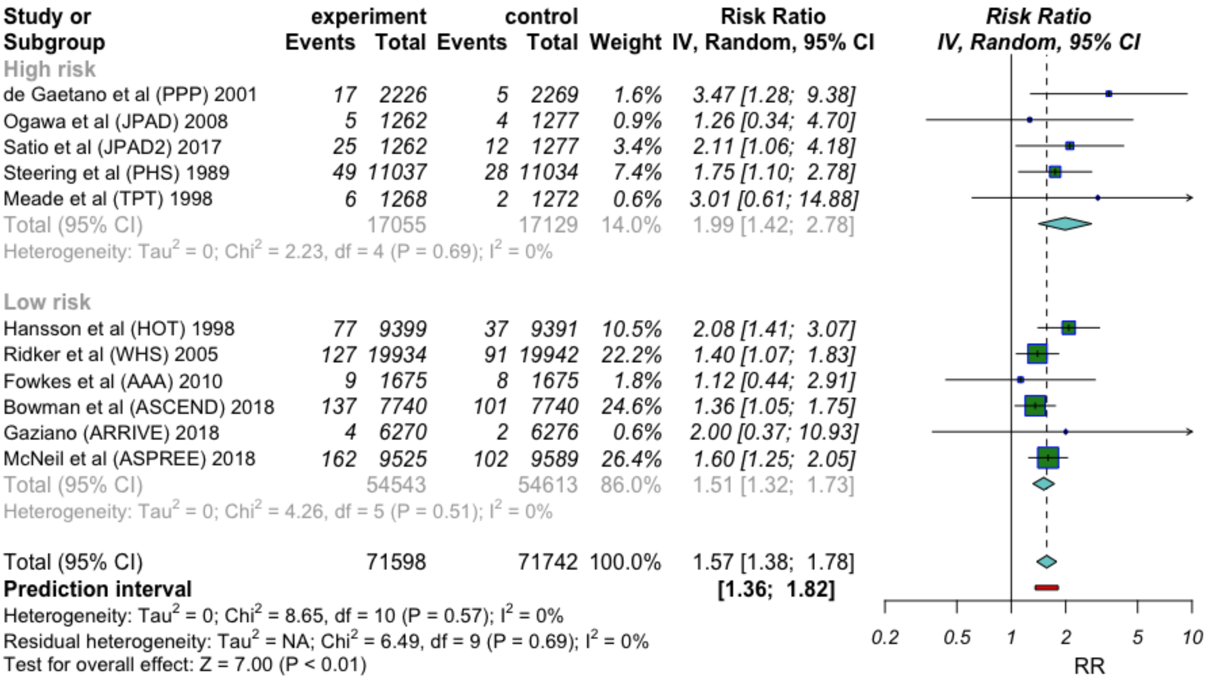

**Figure E**. Summary forest plot for sensitivity analysis on major gastrointestinal bleeding by male populations (excluding 100% male individual trials)

| Study or Subgroup | experiment Events | experiment Total | control Events | control Total | Weight | Risk Ratio IV, Random, 95% CI |
|---|---|---|---|---|---|---|
| male | | | | | | |
| Steering et al (PHS) 1989 | 49 | 11037 | 28 | 11034 | 7.4% | 1.75 [1.10; 2.78] |
| Meade et al (TPT) 1998 | 6 | 1268 | 2 | 1272 | 0.6% | 3.01 [0.61; 14.88] |
| Total (95% CI) | | 12305 | | 12306 | 8.1% | 1.82 [1.17; 2.85] |
| Heterogeneity: $Tau^2 = 0$; $Chi^2 = 0.41$, df = 1 (P = 0.52); $I^2 = 0\%$ | | | | | | |
| not all male | | | | | | |
| de Gaetano et al (PPP) 2001 | 17 | 2226 | 5 | 2269 | 1.6% | 3.47 [1.28; 9.38] |
| Ogawa et al (JPAD) 2008 | 5 | 1262 | 4 | 1277 | 0.9% | 1.26 [0.34; 4.70] |
| Satio et al (JPAD2) 2017 | 25 | 1262 | 12 | 1277 | 3.4% | 2.11 [1.06; 4.18] |
| Hansson et al (HOT) 1998 | 77 | 9399 | 37 | 9391 | 10.5% | 2.08 [1.41; 3.07] |
| Ridker et al (WHS) 2005 | 127 | 19934 | 91 | 19942 | 22.2% | 1.40 [1.07; 1.83] |
| Fowkes et al (AAA) 2010 | 9 | 1675 | 8 | 1675 | 1.8% | 1.12 [0.44; 2.91] |
| Bowman et al (ASCEND) 2018 | 137 | 7740 | 101 | 7740 | 24.6% | 1.36 [1.05; 1.75] |
| Gaziano (ARRIVE) 2018 | 4 | 6270 | 2 | 6276 | 0.6% | 2.00 [0.37; 10.93] |
| McNeil et al (ASPREE) 2018 | 162 | 9525 | 102 | 9589 | 26.4% | 1.60 [1.25; 2.05] |
| Total (95% CI) | | 59293 | | 59436 | 91.9% | 1.55 [1.36; 1.77] |
| Heterogeneity: $Tau^2 = 0$; $Chi^2 = 7.77$, df = 8 (P = 0.46); $I^2 = 0\%$ | | | | | | |
| Total (95% CI) | | 71598 | | 71742 | 100.0% | 1.57 [1.38; 1.78] |
| **Prediction interval** | | | | | | **[1.36; 1.82]** |

Heterogeneity: $Tau^2 = 0$; $Chi^2 = 8.65$, df = 10 (P = 0.57); $I^2 = 0\%$
Residual heterogeneity: $Tau^2 = NA$; $Chi^2 = 8.17$, df = 9 (P = 0.52); $I^2 = 0\%$
Test for overall effect: Z = 7.00 (P < 0.01)

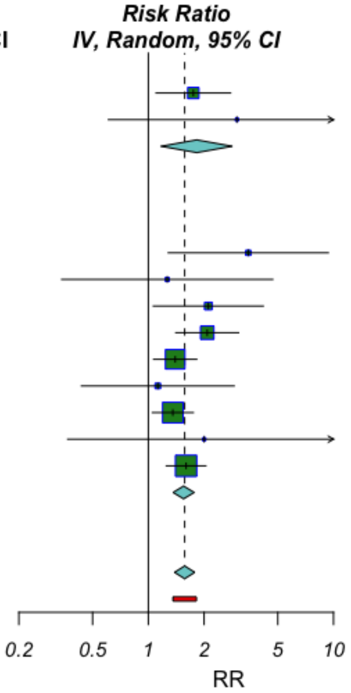

**Figure F**. Summary forest plot for sensitivity analysis on major gastrointestinal bleeding by diabetes status (excluding 100% diabetic individual trials and restricted on 100% diabetic individual trials)

| Study or Subgroup | experiment Events | experiment Total | control Events | control Total | Weight | Risk Ratio IV, Random, 95% CI |
|---|---|---|---|---|---|---|
| no | | | | | | |
| de Gaetano et al (PPP) 2001 | *17* | *2226* | *5* | *2269* | *1.6%* | *3.47 [1.28; 9.38]* |
| Steering et al (PHS) 1989 | *49* | *11037* | *28* | *11034* | *7.4%* | *1.75 [1.10; 2.78]* |
| Hansson et al (HOT) 1998 | *77* | *9399* | *37* | *9391* | *10.5%* | *2.08 [1.41; 3.07]* |
| Ridker et al (WHS) 2005 | *127* | *19934* | *91* | *19942* | *22.2%* | *1.40 [1.07; 1.83]* |
| Fowkes et al (AAA) 2010 | *9* | *1675* | *8* | *1675* | *1.8%* | *1.12 [0.44; 2.91]* |
| Gaziano (ARRIVE) 2018 | *4* | *6270* | *2* | *6276* | *0.6%* | *2.00 [0.37; 10.93]* |
| McNeil et al (ASPREE) 2018 | *162* | *9525* | *102* | *9589* | *26.4%* | *1.60 [1.25; 2.05]* |
| Meade et al (TPT) 1998 | *6* | *1268* | *2* | *1272* | *0.6%* | *3.01 [0.61; 14.88]* |
| Total (95% CI) | | 61334 | | 61448 | 71.1% | 1.63 [1.41; 1.90] |
| Heterogeneity: $Tau^2 = 0$; $Chi^2 = 6.29$, df = 7 (P = 0.51); $I^2 = 0\%$ | | | | | | |
| yes | | | | | | |
| Ogawa et al (JPAD) 2008 | *5* | *1262* | *4* | *1277* | *0.9%* | *1.26 [0.34; 4.70]* |
| Satio et al (JPAD2) 2017 | *25* | *1262* | *12* | *1277* | *3.4%* | *2.11 [1.06; 4.18]* |
| Bowman et al (ASCEND) 2018 | *137* | *7740* | *101* | *7740* | *24.6%* | *1.36 [1.05; 1.75]* |
| Total (95% CI) | | 10264 | | 10294 | 28.9% | 1.43 [1.13; 1.80] |
| Heterogeneity: $Tau^2 = 0$; $Chi^2 = 1.43$, df = 2 (P = 0.49); $I^2 = 0\%$ | | | | | | |
| Total (95% CI) | | 71598 | | 71742 | 100.0% | 1.57 [1.38; 1.78] |
| **Prediction interval** | | | | | | **[1.36; 1.82]** |

Heterogeneity: $Tau^2 = 0$; $Chi^2 = 8.65$, df = 10 (P = 0.57); $I^2 = 0\%$
Residual heterogeneity: $Tau^2 = NA$; $Chi^2 = 7.73$, df = 9 (P = 0.56); $I^2 = 0\%$
Test for overall effect: Z = 7.00 (P < 0.01)

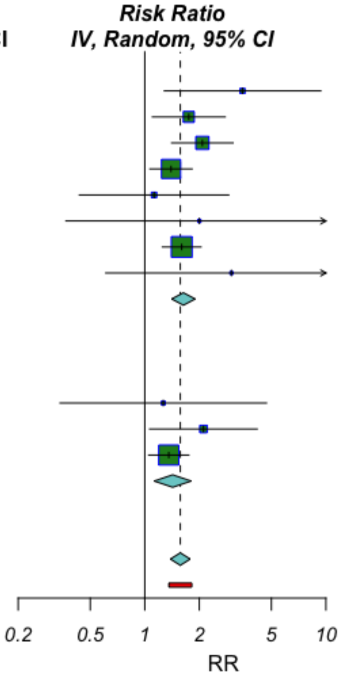

**Figure G**. Summary forest plot for sensitivity analysis on major gastrointestinal bleeding by placebo use (excluding placebo use trials)

| Study or Subgroup | experiment Events | experiment Total | control Events | control Total | Weight | Risk Ratio IV, Random, 95% CI |
|---|---|---|---|---|---|---|
| no | | | | | | |
| de Gaetano et al (PPP) 2001 | *17* | *2226* | *5* | *2269* | *1.6%* | *3.47 [1.28; 9.38]* |
| Ogawa et al (JPAD) 2008 | *5* | *1262* | *4* | *1277* | *0.9%* | *1.26 [0.34; 4.70]* |
| Satio et al (JPAD2) 2017 | *25* | *1262* | *12* | *1277* | *3.4%* | *2.11 [1.06; 4.18]* |
| Total (95% CI) | | 4750 | | 4823 | 6.0% | 2.23 [1.33; 3.74] |
| Heterogeneity: $Tau^2 = 0$; $Chi^2 = 1.5$, df = 2 (P = 0.47); $I^2 = 0\%$ | | | | | | |
| yes | | | | | | |
| Steering et al (PHS) 1989 | *49* | *11037* | *28* | *11034* | *7.4%* | *1.75 [1.10; 2.78]* |
| Hansson et al (HOT) 1998 | *77* | *9399* | *37* | *9391* | *10.5%* | *2.08 [1.41; 3.07]* |
| Ridker et al (WHS) 2005 | *127* | *19934* | *91* | *19942* | *22.2%* | *1.40 [1.07; 1.83]* |
| Fowkes et al (AAA) 2010 | *9* | *1675* | *8* | *1675* | *1.8%* | *1.12 [0.44; 2.91]* |
| Bowman et al (ASCEND) 2018 | *137* | *7740* | *101* | *7740* | *24.6%* | *1.36 [1.05; 1.75]* |
| Gaziano (ARRIVE) 2018 | *4* | *6270* | *2* | *6276* | *0.6%* | *2.00 [0.37; 10.93]* |
| McNeil et al (ASPREE) 2018 | *162* | *9525* | *102* | *9589* | *26.4%* | *1.60 [1.25; 2.05]* |
| Meade et al (TPT) 1998 | *6* | *1268* | *2* | *1272* | *0.6%* | *3.01 [0.61; 14.88]* |
| Total (95% CI) | | 66848 | | 66919 | 94.0% | 1.54 [1.35; 1.75] |
| Heterogeneity: $Tau^2 = 0$; $Chi^2 = 5.29$, df = 7 (P = 0.62); $I^2 = 0\%$ | | | | | | |
| Total (95% CI) | | 71598 | | 71742 | 100.0% | 1.57 [1.38; 1.78] |
| **Prediction interval** | | | | | | **[1.36; 1.82]** |

Heterogeneity: $Tau^2 = 0$; $Chi^2 = 8.65$, df = 10 (P = 0.57); $I^2 = 0\%$
Residual heterogeneity: $Tau^2 = NA$; $Chi^2 = 6.79$, df = 9 (P = 0.66); $I^2 = 0\%$
Test for overall effect: Z = 7.00 (P < 0.01)

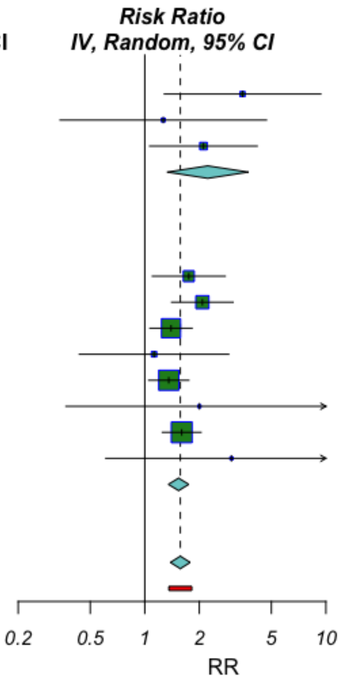

**Figure H**. Summary forest plot for sensitivity analysis on major gastrointestinal bleeding excluding TPT trial

| Study or Subgroup | experiment Events | experiment Total | control Events | control Total | Weight | Risk Ratio IV, Random, 95% CI |
|---|---|---|---|---|---|---|
| no | | | | | | |
| de Gaetano et al (PPP) 2001 | *17* | *2226* | *5* | *2269* | *1.6%* | *3.47 [1.28; 9.38]* |
| Ogawa et al (JPAD) 2008 | *5* | *1262* | *4* | *1277* | *0.9%* | *1.26 [0.34; 4.70]* |
| Satio et al (JPAD2) 2017 | *25* | *1262* | *12* | *1277* | *3.4%* | *2.11 [1.06; 4.18]* |
| Steering et al (PHS) 1989 | *49* | *11037* | *28* | *11034* | *7.4%* | *1.75 [1.10; 2.78]* |
| Hansson et al (HOT) 1998 | *77* | *9399* | *37* | *9391* | *10.5%* | *2.08 [1.41; 3.07]* |
| Ridker et al (WHS) 2005 | *127* | *19934* | *91* | *19942* | *22.2%* | *1.40 [1.07; 1.83]* |
| Fowkes et al (AAA) 2010 | *9* | *1675* | *8* | *1675* | *1.8%* | *1.12 [0.44; 2.91]* |
| Bowman et al (ASCEND) 2018 | *137* | *7740* | *101* | *7740* | *24.6%* | *1.36 [1.05; 1.75]* |
| Gaziano (ARRIVE) 2018 | *4* | *6270* | *2* | *6276* | *0.6%* | *2.00 [0.37; 10.93]* |
| McNeil et al (ASPREE) 2018 | *162* | *9525* | *102* | *9589* | *26.4%* | *1.60 [1.25; 2.05]* |
| Total (95% CI) | | 70330 | | 70470 | 99.4% | 1.56 [1.38; 1.78] |
| Heterogeneity: $Tau^2 = 0$; $Chi^2 = 8.01$, df = 9 ($P = 0.53$); $I^2 = 0\%$ | | | | | | |
| yes | | | | | | |
| Meade et al (TPT) 1998 | *6* | *1268* | *2* | *1272* | *0.6%* | *3.01 [0.61; 14.88]* |
| Total (95% CI) | | 1268 | | 1272 | 0.6% | 3.01 [0.61; 14.88] |
| Heterogeneity: not applicable | | | | | | |
| Total (95% CI) | | 71598 | | 71742 | 100.0% | 1.57 [1.38; 1.78] |
| **Prediction interval** | | | | | | **[1.36; 1.82]** |

Heterogeneity: $Tau^2 = 0$; $Chi^2 = 8.65$, df = 10 ($P = 0.57$); $I^2 = 0\%$
Residual heterogeneity: $Tau^2 = NA$; $Chi^2 = 8.01$, df = 9 ($P = 0.53$); $I^2 = 0\%$
Test for overall effect: $Z = 7.00$ ($P < 0.01$)

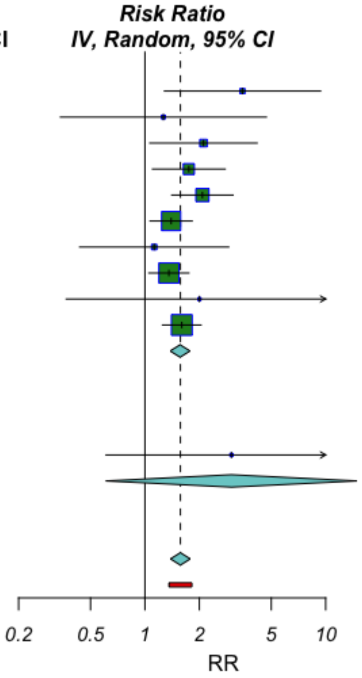

Supplement: Supplementary file 14 [file datasheet11.pdf]
