# Supplementary figures and images for: Pros and Cons of Aspirin for the Primary Prevention of Cardiovascular Events: A Secondary Study of Trial Sequential Analysis
Source: Front Pharmacol. 2021 Jan 14;11:592116. doi: 10.3389/fphar.2020.592116 (PMC7845480; doi:10.3389/fphar.2020.592116)

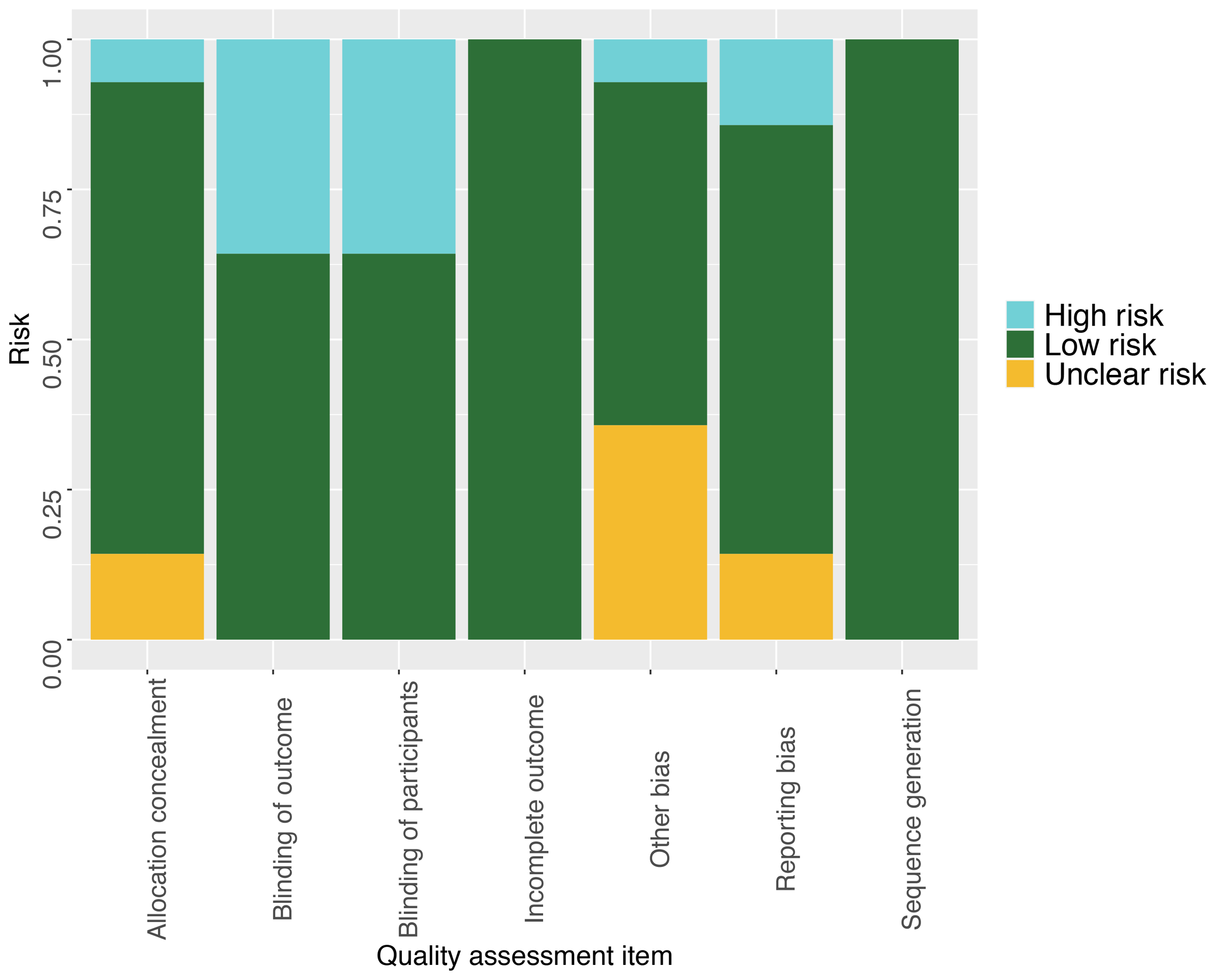

Supplement: Supplementary file 15 [file image1.tif]

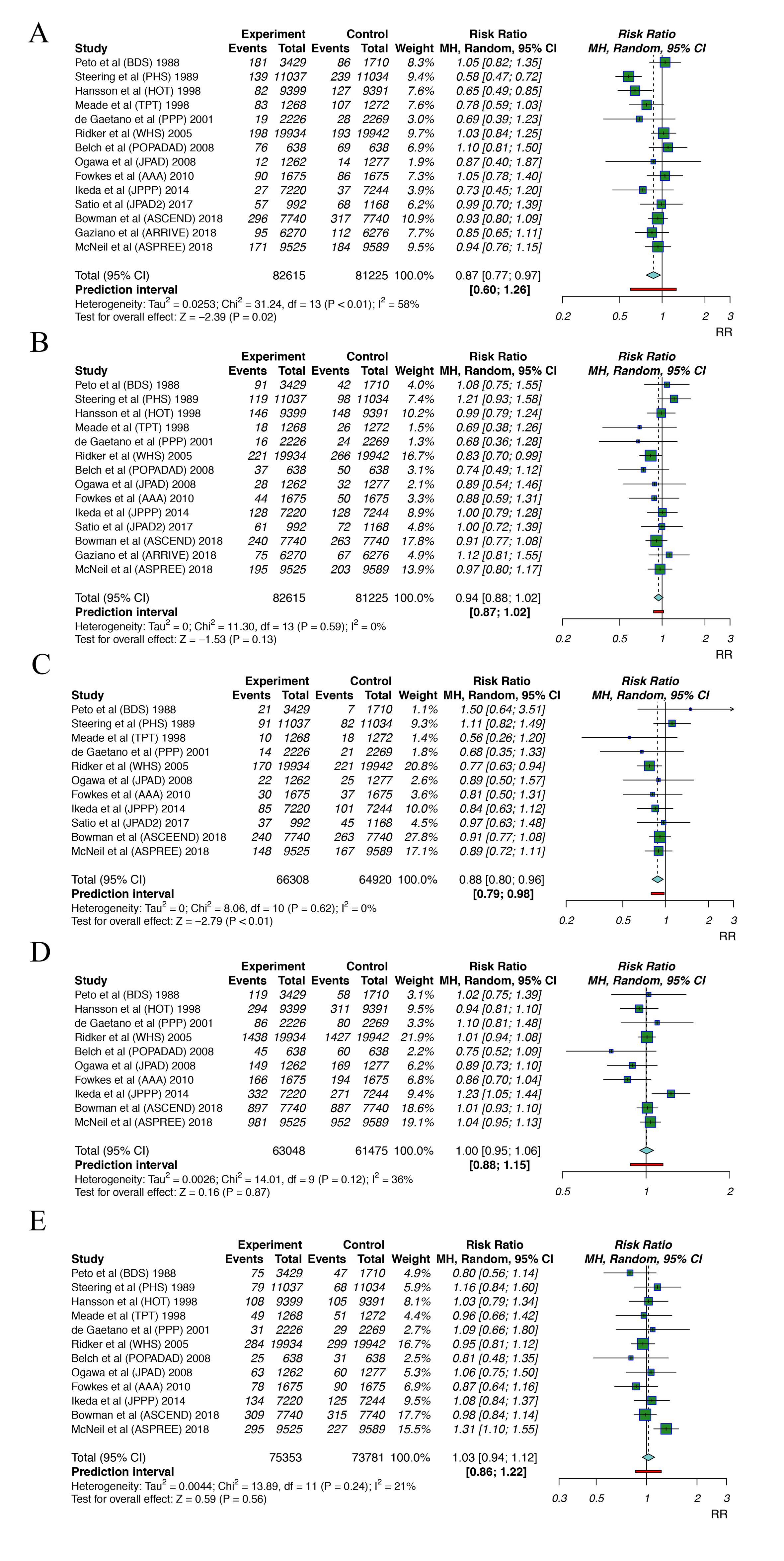

Supplement: Supplementary file 16 [file image2.tif]

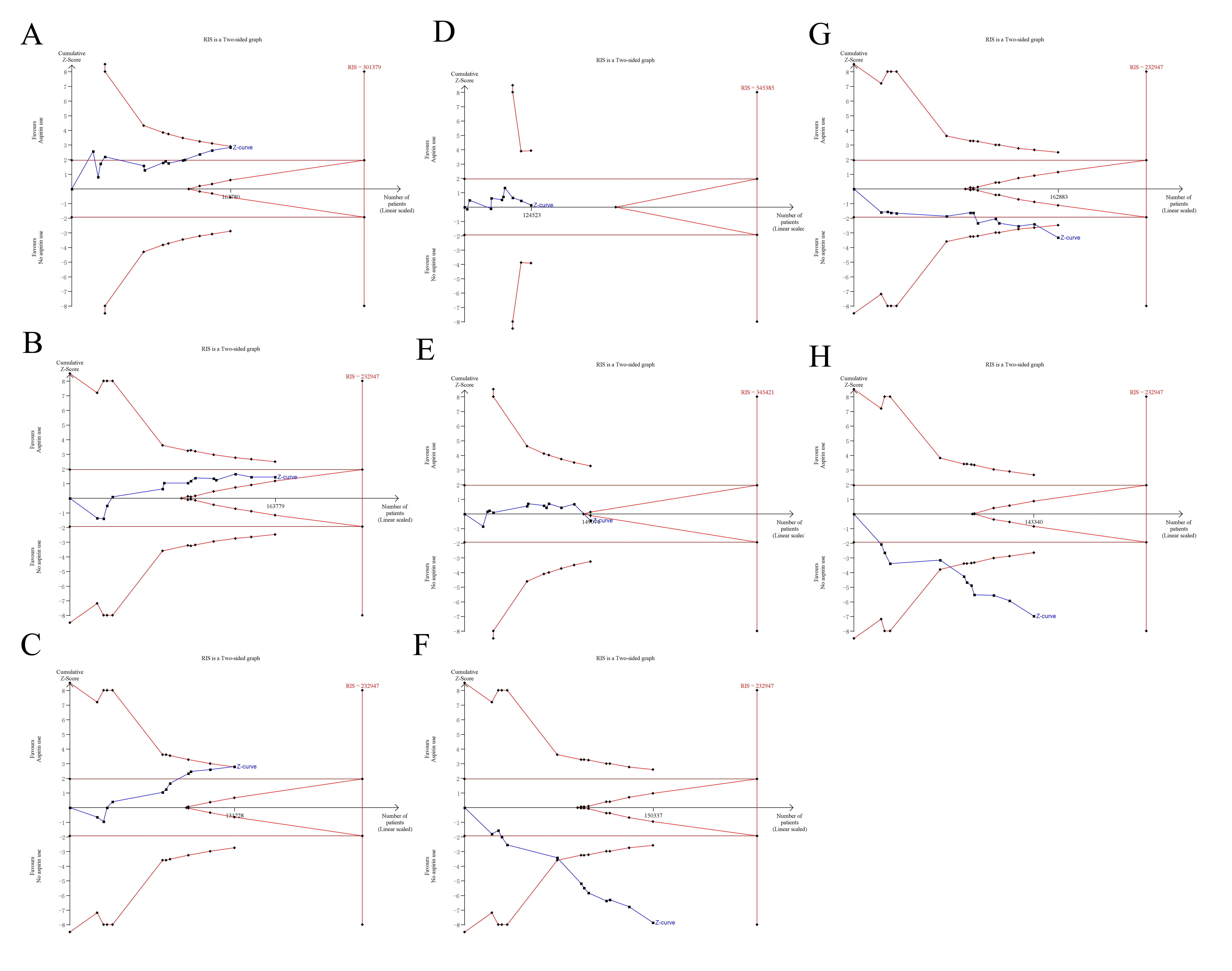

Supplement: Supplementary file 17 [file image3.tif]

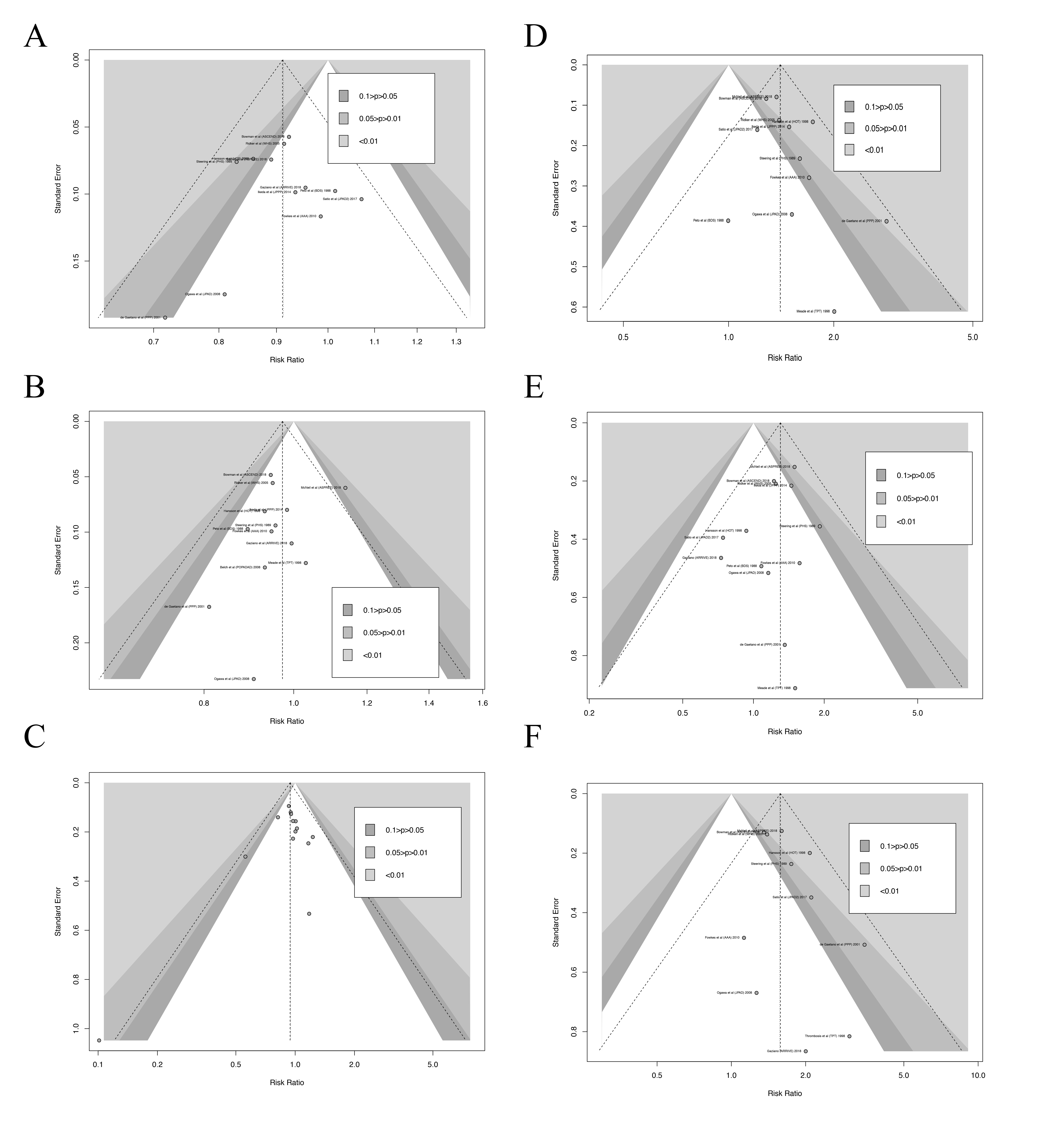

Supplement: Supplementary file 18 [file image4.tif]
